# Combining triazole ligation and enzymatic glycosylation on solid phase simplifies the synthesis of very long glycoprotein analogues†
†Electronic supplementary information (ESI) available: Detailed synthetic procedures, characterization and optimization. See DOI: 10.1039/c5sc00773a


**DOI:** 10.1039/c5sc00773a

**Published:** 2015-04-14

**Authors:** Mathieu Galibert, Véronique Piller, Friedrich Piller, Vincent Aucagne, Agnès F. Delmas

**Affiliations:** a Centre de Biophysique Moléculaire , CNRS UPR 4301 , Rue Charles Sadron , 45071 Orléans Cedex 2 , France . Email: aucagne@cnrs-orleans.fr

## Abstract

The solid-phase chemical assembly of a protein through iterative chemoselective ligation of unprotected peptide segments can be followed with chemical and/or enzymatic transformations of the resulting immobilized protein, the latter steps thus benefitting from the advantages provided by the solid support. We demonstrate here the usefulness of this strategy for the chemo-enzymatic synthesis of glycoprotein analogues. A linker was specifically designed for application to the synthesis of *O*-glycoproteins: this new linker is readily cleaved under mild aqueous conditions compatible with very sensitive glycosidic bonds, but is remarkably stable under a wide range of chemical and biochemical conditions. It was utilized for solid-supported N-to-C peptidomimetic triazole ligation followed by enzymatic glycosylation, ultimately leading to a very large MUC1-derived glycoprotein containing 160 amino acid residues, 24 α-GalNAc moieties linked to Ser and Thr, and 3 triazoles as peptide bond mimetics.

## Abstract 2

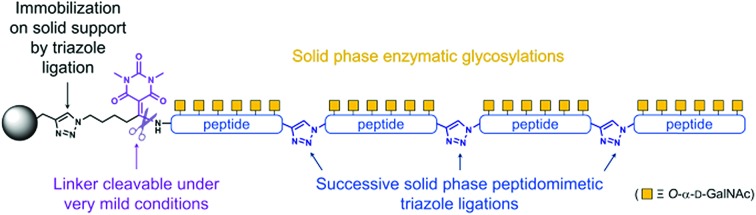
Solid phase chemical ligation followed by enzymatic glycosylation exploits the advantages of a solid support to minimize the purification steps, constituting a promising approach for the synthesis of complex glycoproteins.

## Introduction

Solid-supported chemoselective ligations of unprotected biomolecule segments^1^,^2^ have opened a route towards high-yielding and rapid synthesis of proteins and protein analogues,^1^ peptide–PNA conjugates,2*a* and more recently very long oligonucleotides.2*b* The main benefit of such an iterative strategy relies on the use of simple draining processes as alternatives for the laborious intermediate chromatographic purification and lyophilization steps. In addition, post-ligation synthetic transformations of the resulting immobilized biomacromolecule can also benefit from the advantages provided by the solid support. This has been demonstrated for disulfide formation1*d* or cysteine desulfurization,1i,^3^ but the extension to other reactions like enzyme-mediated transformations or chemical conjugation with probes remains to be explored.

The chemo-enzymatic synthesis of glycoproteins is a fast growing area,^4^ and we sought to combine solid phase ligations of peptide fragments with enzymatic glycosylation^5^,^6^ to provide a simplified access to complex glycoproteins. We herein focus on *O*-glycoproteins, identified as particularly demanding targets considering the narrow chemical compatibilities of *O*-glycans, which are sensitive to base-mediated β-elimination but also to acid hydrolysis, particularly the biologically-relevant sialylated compounds.

**Fig. 1:**
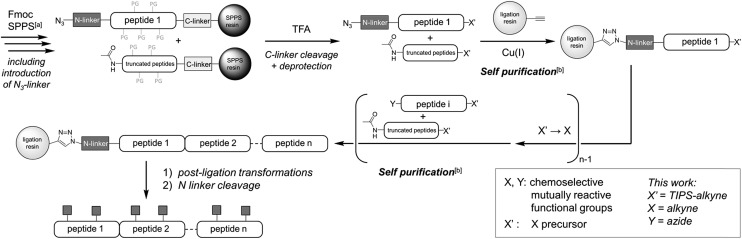
Overall description of the strategy, highlighting the self-purification nature1*e* of the solid-supported N-to-C assembly. ^a^ The SPPS process includes an acetylation-mediated capping step after each amino acid coupling step. ^b^ Subsequent click-chemistry can discriminate the target peptide having an azido group at the N-terminus from the undesired truncated acetylated byproducts.

**Fig. 2:**
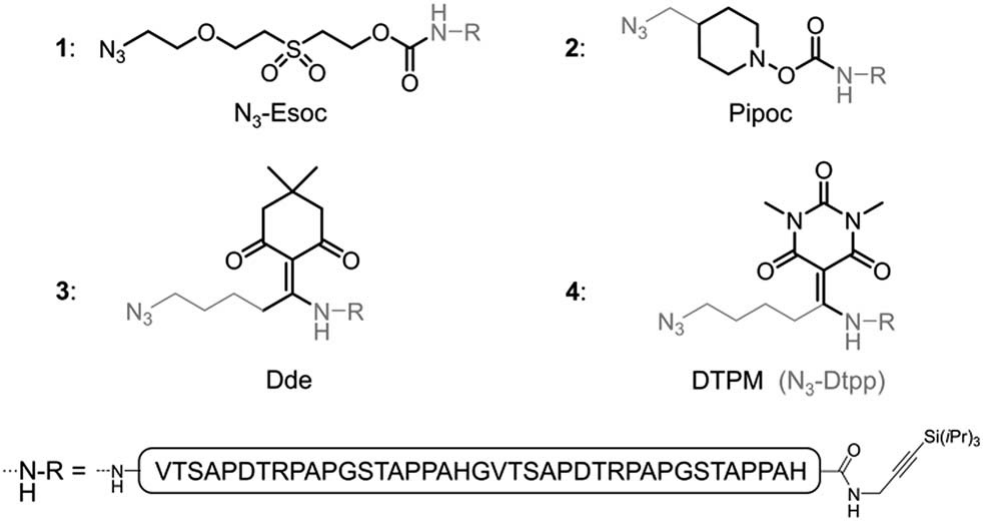
Linker-containing peptides evaluated in this work.

Besides protein elongation from the C-terminus to the N-terminus (C-to-N),1a–d,g–i *i.e.* in the same direction as solid-phase peptide synthesis (SPPS), “reversed” N-to-C ligation methodologies1a,e,f benefit from a self-purification effect (Fig. 1):^7^ provided that SPPS elongation includes an acetylation step before the removal of each Fmoc group, the truncated *N*-acetylated contaminants of the peptide segments can be eliminated by a simple draining after each ligation step. Paramount to such solid-phase ligation strategies are linkers to tether the starting unprotected (glyco)peptide segment to a water-compatible solid support (ligation resin, Fig. 1). For N-to-C protein assembly, a hetero-bifunctional N-terminal linker is introduced during SPPS as the Nα-protection group of the last amino acid. After cleavage from the SPPS resin and concomitant removal of the side-chain protective groups, the linker is used for chemoselective immobilization of the starting unprotected peptide segment on the ligation resin. After the ligation-based assembly, the linker must allow for a traceless and quantitative release of the protein, while being stable to a wide range of conditions. We recently introduced an azide-functionalized N_3_-Esoc1e,f linker (**1**, Fig. 2) that conveniently enables immobilization of the starting peptide segment by either Cu^I^-catalyzed azide/alkyne cycloaddition1*e* (CuAAC) or strain-promoted azide/alkyne cycloaddition1*f* (SPAAC), forming a bio- and chemically-stable triazole linkage. However, the base treatment necessary for its cleavage is not compatible with *O*-glycopeptides (ESI, Fig. S31†). Other currently available linkers also call for harsh release procedures, either under basic,1a,e,f or acidic1b,g–i conditions. New linkers cleavable under milder conditions and thus compatible with a wider range of post-translational or other modifications are still seriously needed to further push back the limits of (glyco)protein chemical synthesis.

We report here on the development of a promising N-terminal linker cleavable under mild neutral conditions and compatible with both NCL and peptidomimetic triazole ligation (PTL) as well as a wide range of chemical and biochemical treatments. Its usefulness is illustrated here by the synthesis of high molecular weight (up to ∼20 kDa)^8^ triazole-containing glycoprotein analogues, using a unprecedented combination of successive solid phase ligations and enzymatic glycosylations, ending with the release of glycoprotein analogues. Only one final HPLC purification step leads to well defined compounds mainly composed of single glycoforms.

## Results and discussion

We chose as a model the human mucin MUC1, which has been widely used as a benchmark *O*-glycoprotein for chemical ligation-based methodological developments.1e,h,^9^ The extracellular domain of MUC1 is mainly constituted of identical tandem repeats of 20 amino acids (aa), VT^2^S^3^APDT^7^ RPAPGS^13^T^14^APPAHG, with 5 potential Ser/Thr *O*-glycosylation sites. This glycoprotein, and its glycopeptide derivatives, represents a major target for the development of anti-tumour vaccines,^10^ as it is overexpressed in most epithelial cancers, decorated with short tumour-specific *O*-glycans (α-GalNAc, β-Gal-1,3-α-GalNAc, or sialyl α-GalNAc).

### Optimization of an N-terminal linker adapted to the synthesis of *O*-glycopeptides and *O*-glycoproteins

Inspired by existing amine protecting groups, we selected three scaffolds (Fig. 2) that could be compatible with *O*-glycoproteins: Pipoc,^11^ cleavable under mild reducing conditions, and enamine-based Dde^12^ and DTPM,^13^ cleavable upon treatment with nitrogen nucleophiles. We prepared azide-functionalized derivatives suitable for immobilization on a solid support through CuAAC or SPAAC. The resulting hetero-bifunctional linkers were installed during SPPS at the N-terminus of a protected MUC1 double tandem repeat sequence. Peptides were also equipped at their C-termini with a silyl-protected alkyne^14^ for further solid phase triazole ligations.

The stabilities of the new azido-linkers (peptides **2–4**) were systematically screened in solution under a representative set of conditions (ESI, Table S1†), and compared to N_3_-Esoc (peptide **1**). Unexpectedly, the Pipoc-based linker was not stable in the TFA cocktail used to cleave and deprotect peptide **2** after SPPS, making it unfit for our purpose. Surprisingly, we also observed slow cleavage of the Dde-derived linker (peptide **3**) under mildly acidic and even neutral aqueous conditions.^15^ In contrast, the DTPM-based linker (peptide **4**) was perfectly stable under acidic conditions as well as to a wide range of chemical treatments, including particularly harsh sodium methoxide-based deacetylation of chemically-introduced glycans, NCL and PTL conditions, and TBAF- or Ag-mediated alkyne desilylation. We thus concentrated our efforts on this promising 1-azido-5-[1,3-dimethyl-2,4,6(1*H*,3*H*,5*H*)-trioxopyrimidine-5-ylidene]pentyl linker, hereafter referred as N_3_-Dtpp.

Though perfectly stable towards nucleophiles such as thiolates (NCL conditions and cysteine), a large excess of sodium methoxide or primary amines (Tris buffer), Dtpp was quantitatively cleaved by 1 M aqueous hydrazine (pH 9.5). However, along with linker cleavage we observed the concomitant formation of two by-products showing an increase in mass of +2 Da and +4 Da. We suspected a diimide-based reduction of the silylalkyne into the corresponding *Z*-alkene and alkane,^16^ which was confirmed using a simple model alkyne (see ESI p S37†). Optimized, milder cleavage conditions using a 1 M aqueous hydroxylamine solution containing 100 mM sodium ascorbate,^17^ either buffered at neutral pH or without buffer (pH 8.5), cleanly cleaved the linker within a few hours.

### Application to the synthesis of *O*-glycopeptides through solid-phase enzymatic glycosylation

**Fig. 3:**
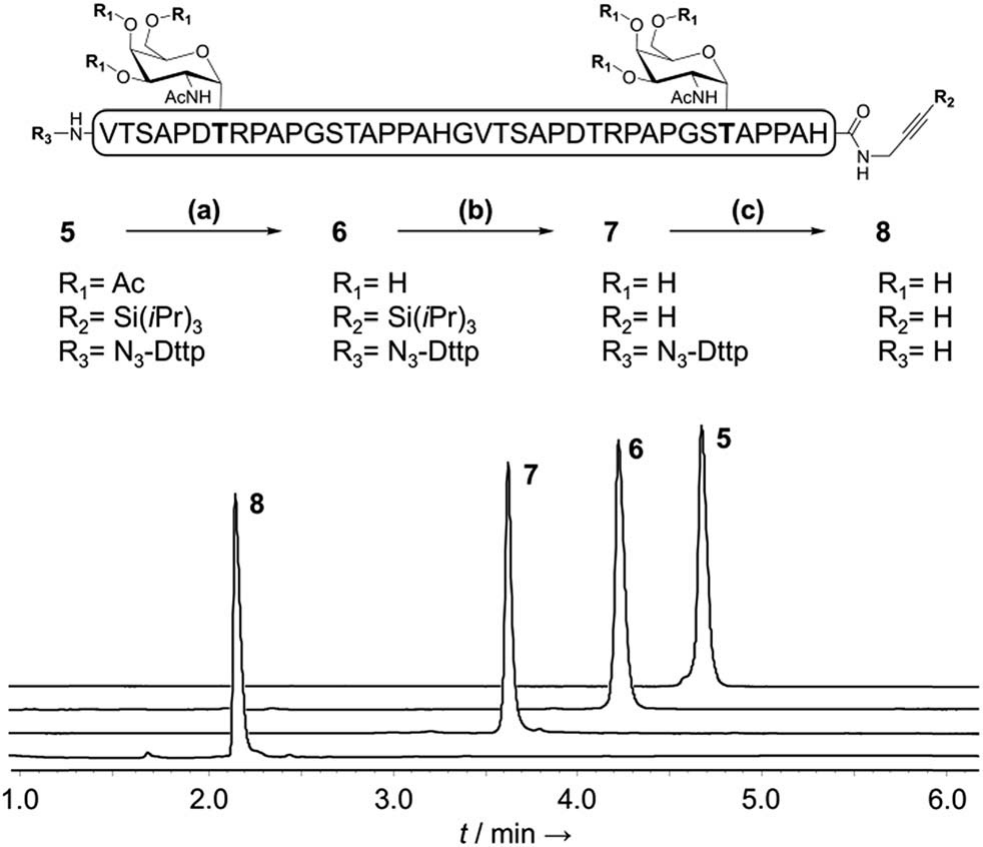
Sequential chemoselective deprotections of purified glycopeptide **5** to give **8**. (a) MeONa/MeOH (b) TBAF/DMF (c) aqueous NH_2_OH. The RP-HPLC traces (*λ* = 214 nm) correspond to the successive crude deprotection mixtures.

With a reliable linker cleavage procedure established, we wanted to test N_3_-Dtpp with a more challenging example, such as glycopeptide **5** bearing a silylalkyne at its C-terminus and two acetate-protected Thr(Ac_3_-α-d-GalNAc) groups chemically introduced as building blocks during SPPS (Fig. 3). **5** was treated with excess sodium methoxide in methanol for 1 h to effect the chemoselective deacetylation of the glycan moieties, cleanly leading to glycopeptide **6** without affecting the Dtpp. Crude **6** was subsequently treated with TBAF to remove the C-terminal tri-isopropylsilyl (TIPS) alkyne protecting group, without showing any trace of sugar release by β-elimination or linker cleavage. Finally, N_3_-Dtpp was easily cleaved by aqueous hydroxylamine. No trace of degradation of the resulting unprotected glycopeptide **8** was observed, even under prolonged cleavage conditions.

**Fig. 4:**
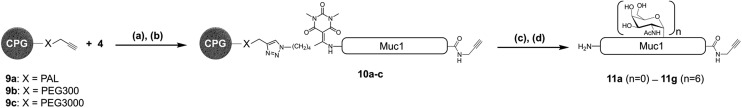
Immobilization and enzymatic glycosylation of a 39 aa solid supported MUC1 peptide. (a) CuBr·Me_2_S, THPTA, HEPES (pH 7.5)/NMP; (b) TBAF, DMF; (c) UDP-GalNAc, GalNAc-T1; (d) 1 M aqueous NH_2_OH. Muc1 refers to the same 39 aa sequence as in Fig. 1 and 2. PAL: 5-[3,5-dimethoxy-4-(aminomethyl)phenoxy]pentanoic acid.

Having demonstrated the chemical robustness of the Dtpp linker we next applied it to the challenge^5^ of solid-supported enzymatic glycosylation by the recombinant polypeptide α-*N*-acetyl-galactosaminyl transferase 1 (GalNAc-T1). This widely-used enzyme catalyzes the specific transfer of α-*N*-acetyl-d-galactosamine (α-GalNAc) from UDP-GalNAc to the hydroxyl groups on the side chains of Ser or Thr. To ensure a complete accessibility to enzymes with a relatively high molecular weight such as GalNAc-T1 (>70 kDa, ∼3 nm diameter), commercially available controlled pore glass (CPG) beads with pore sizes around 100 nm were used as the solid support.^18^ Crude unglycosylated peptide **4** (∼70% purity) was efficiently immobilized onto alkyne-functionalized CPG **9a** through CuAAC then desialylated with TBAF to give supported peptide **10a** (Fig. 4). An aliquot of **10a** was subsequently treated with hydroxylamine to release peptide **11a** into solution. Gratifyingly, **11a** was obtained in a much greater purity than substrate **4** due to the self-purification nature of the capture-and-release process, which eliminates truncated acetylated SPPS co-products (22 different truncated peptide impurities identified in crude **4**).1*e*

In this test, an additional acid-labile PAL linker was inserted between the CPG beads and the alkyne moiety. This double-linker strategy^19^ was employed to check for any peptide still present on the beads after cleavage of Dtpp. Subsequent TFA treatment did not release any additional peptide, confirming the high efficiency of the hydroxylamine-mediated Dtpp cleavage.^20^ As a consequence, we did not introduce a PAL linker and used only Dttp cleavage for quality control or final release in all further experiments.^21^

Although our first assay gave encouraging results, we nevertheless found enzymatic glycosylation of supported peptide **10a** to be much less efficient than a solution phase control experiment. However, satisfactory results were obtained by introducing a 3000 Da PEG spacer between the peptide and the solid support^22^ (**10c**): after peptide release, LC-MS analysis showed a mixture mainly composed of pentaglycosylated (**11f**) and hexaglycosylated (**11g**) peptides, with a product distribution comparable to that obtained in solution (ESI, Tables S6–S8†).^23^ Only very low amounts (<2%) of compounds containing more than six α-GalNAc could be detected in the mixture. The major product, HPLC-purified hexaglycosylated **11g**, was shown to be a single glycoform, its homogeneity being assessed by top-down analysis using electron-transfer dissociation ESI-MS/MS.^24^,^25^ The glycosylated amino acid residues were unequivocally mapped to Thr2, Ser13 and Thr14 in the first tandem repeat, and to their counterparts in the second, Thr22, Ser33 and Thr34. The four remaining possible *O*-glycosylation sites, Ser3, Thr7, Ser23 and Thr27, were clearly shown to be unmodified. These results are in perfect accordance with the sequence specificity of GalNAc-T1: this enzyme is known to be unable to glycosylate these Ser and Thr sites within MUC1 tandem repeats.^26^ However, the traces of heptaglycosylated products we observed probably correspond to glycosylation, with a very slow kinetic rate, at either one of these four positions. Importantly, this also demonstrates that the GalNAc-T1 tolerates the presence of the Dtpp linker at the N-terminus in close proximity to Thr2.

Considering that the glycosylation of Thr7 and Thr27 could be important for an immune response,^10^ chemically-synthesized glycopeptide **6** containing two GalNAc units at these positions was immobilized through CuAAC on the optimized support **9c**, then desialylated and subjected to enzymatic glycosylation with GalNAc-T1. About eighty percent of the peptide was converted to the octaglycosylated peptide (**S21′**, ESI Fig. S54†)^27^: the chemical pre-introduction of GalNAc units significantly increased the enzymatic glycosylation rate. This interesting result may be rationalized by the interaction of the lectin domain of the enzyme^28^ with the glycopeptide and/or by a conformational change of the peptide induced by the introduction of the two GalNAc residues.^29^ HPLC purification furnished pure **18** in a 48% yield.

**Fig. 5:**
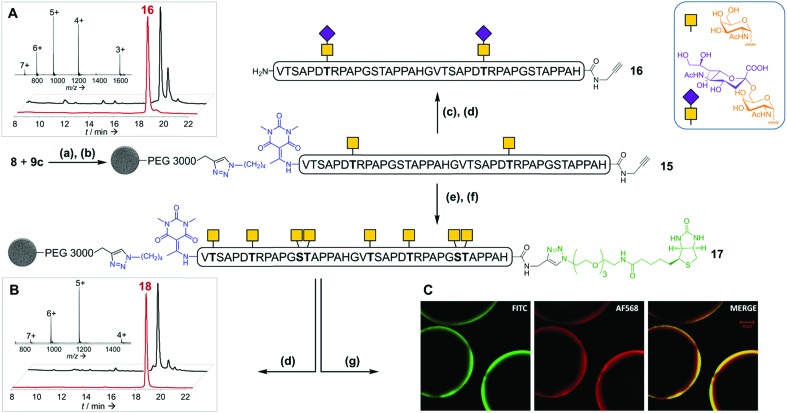
Solid phase glycosylation and conjugation to a biotin probe of peptide **15**. (a) CuBr·Me_2_S, THPTA, HEPES (pH 7.5)/NMP; (b) TBAF, DMF; (c) CMP-NeuAc, ST6GalNAc I; (d) 1 M NH_2_OH; (e) UDP-GalNAc, GalNAc-T1; (f) N_3_-PEG_3_-biotin, CuBr·Me_2_S, THPTA, HEPES (pH 7.5)/NMP; (g) FITC-conjugated *Vicia villosa* lectin, Alexa-fluor 568-labelled streptavidin. (A): RP-HPLC profile (*λ* = 214 nm) of crude (black) and purified (red) glycopeptide **16**. Inset: ESI-HRMS spectrum of purified **16**. (B): RP-HPLC profile (*λ* = 214 nm) of crude (black) and purified (red) glycopeptide **18**. Inset: ESI-HRMS spectrum of purified **18**. (C): Confocal fluorescence microscopy analysis of dually-labelled bead-supported biotinyl-glycopeptide **17**.

We then considered the possibility of extending the glycans towards more complex *O*-glycans using appropriate glycosyltransferases. We chose to introduce sialic acid (NeuAc) moieties, as a relevant example. They are found in both *O*- and *N*-glycoproteins. Sialylated antigens are of great interest and promise in the cancer vaccine field and are particularly difficult to prepare by purely chemical means.^30^ In addition, sialyl glycosides are unstable under aqueous acidic conditions. The introduction of sialic acid groups was performed by incubation of supported peptide **15** with the sialyltransferase ST6GalNAc I and CMP-NeuAc under unoptimized conditions. The linker cleavage with hydroxylamine was perfectly compatible with the sialyl moieties. HPLC purification provided di-sialylated glycopeptide **16** in a 38% yield (Fig. 5A). These results highlight the excellent flexibility and robustness of our strategy, which allows both enzymatic and chemical glycosylations to be readily combined.

### Application to the combination of solid phase glycosylation and chemoselective ligations

To further illustrate the versatility of our linker, we exploited the C-terminal alkyne for additional chemoselective ligation reactions. Firstly, solid phase CuAAC-based conjugation was used to label the synthetic glycopeptide with a biotin probe, giving compound **18** (Fig. 5B) in extraordinarily high purity considering its chemical complexity (45% isolated yield). We also demonstrated full compatibility of the solid support with imaging techniques typically used for living cells, using a variety of fluorescent antibodies, streptavidin and lectins (ESI Table S8† and Fig. 5C). The latter result suggests that solid-supported glycopeptide mixtures could be directly screened against biologically-relevant targets while still immobilized.

**Fig. 6:**
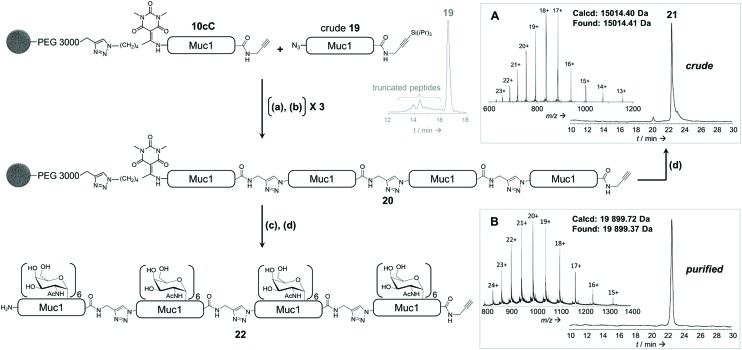
Combination of solid phase peptidomimetic triazole ligation and enzymatic glycosylation. (a) azidopeptide **19**, CuBr·Me_2_S, THPTA, HEPES (pH 7.5)/NMP; (b) TBAF, phenol, DMF; (c) UDP-GalNAc, GalNAc-T1; (d) 1 M NH_2_OH. (A): RP-HPLC profile (*λ* = 214 nm) and ESI-HRMS spectrum of crude unglycosylated 160 residue peptide **21**. (B): RP-HPLC profile (*λ* = 214 nm) and ESI-HRMS spectrum of HPLC-purified glycopeptide **22**.

Finally, we applied our linker to the synthesis of very long polypeptides through iterative solid-supported triazole ligations. Starting from immobilized peptide **10c**, three successive ligation/desilylation cycles, using crude peptide **19** (∼70% purity) featuring both an N-terminal azide group and a C-terminal silylalkyne, gave a very clean 160 residue supported compound **20**, as evidenced by LC-MS analysis of the crude released peptide **21** (Fig. 6A). This shows the efficiency of the N-to-C solid-phase chemical ligation strategy. It also illustrates the self-purification effect.1*e*

Subsequent solid-supported enzymatic glycosylation of **20** with GalNAc-T1, under the conditions optimized for 40-mer **10c**, successfully produced the corresponding triazolo-glycoproteins as a mixture of glycoforms, the major ones containing 22, 23 and 24 GalNAc moieties.

Most importantly, the presence of triazoles as amide surrogates did not alter in any way the glycosylation efficiency, as we also rigorously demonstrated with a model triazolopeptide (ESI p S101†). As expected, only low amounts (∼4%) of compounds containing more than 24 GalNAc moieties could be detected, supporting that the triazole-containing glycoprotein analogue **22** bearing 24 saccharide moieties is mainly composed of a single glycoform, glycosylated on the same three sites per tandem repeat that GalNAc-T1 can glycosylate. HPLC purification furnished triazolo-glycoprotein **22** (Fig. 6B) in an overall yield of 6%, taking into account the ligation-mediated assembly, enzymatic glycosylation and purification. Similar results were obtained with 80- and 120-mer compounds containing 12 and 18 α-GalNAc-Ser/Thr moieties and purified in 15% and 10% yields, respectively (ESI, p S115–S119†).

## Conclusions

We have designed a new solid phase synthetic strategy, based on multiple successive solid-supported ligations followed by enzymatic glycosylation, applicable to the synthesis of a wide range of glycopeptides and glycoproteins, and potentially to proteins with other post-translational modifications. This strategy is based on a heterobifunctional N-terminal azido linker, N_3_-Dtpp. Resistant to a wide set of chemical and biochemical transformations, this linker is cleavable under particularly mild neutral aqueous conditions. Its broad applicability was exemplified through the solid phase synthesis of complex mucin-type *O*-glycopeptides by combining enzymatic and chemical glycosylations, bioconjugation, and also direct fluorescence biochemical screening of the supported peptides with lectins and antibodies, which is promising for further biological applications. We also demonstrated that the linker is compatible with chemical ligation techniques such as peptidomimetic triazole ligations, to enable further solid phase elongation and the synthesis of longer and more complex glycoproteins. The latter point was demonstrated with the synthesis of a very large homogeneous 160 residue triazole-containing glycoprotein analogue through three successive ligations followed by enzymatic glycosylation, thus avoiding multiple solution-phase intermediate purification steps. Application of the linker to other ligation techniques such as NCL, to produce native glycoproteins not incorporating triazoles as backbone modifications, is currently underway and will be reported in due course.

## Supplementary Material

Supplementary information
