# Supplementary material for: Combining triazole ligation and enzymatic glycosylation on solid phase simplifies the synthesis of very long glycoprotein analogues
Source: Chem Sci. 2015 Apr 14;6(6):3617–23. doi: 10.1039/c5sc00773a (PMC6085731; doi:10.1039/c5sc00773a)
Supplement: Supplementary file 1 [file SC-006-C5SC00773A-s001.pdf]

Electronic Supplementary Material (ESI) for Chemical Science.
This journal is © The Royal Society of Chemistry 2015

Combining solid phase chemical ligations and enzymatic glycosylations: towards the simplification of glycoprotein synthesis

Mathieu Galibert, Véronique Piller, Friedrich Piller, Vincent Aucagne,* and Agnès F. Delmas

Supplementary information

Table of contents

A General information S3

B Preparation of the model linker-containing peptides **1-4** S19

C Stability study of the linker-containing peptides **1**, **2**, **3** and **4** under selected conditions S31

D Studies of the Dtpp linker cleavage S34

E Synthesis of **8** by successive deprotection of **5** S42

F Studies for improvement of solid supported enzymatic glycosylation S52

G Solid phase enzymatic glycosylation of chemically-glycosylated peptide **6** S72

H Chemical ligation to introduce a biotin probe S82

I Fluorescence labeling of CPG supported peptides S87

J Demonstration that presence of a triazole as amide surrogate does not alter enzymatic glycosylation efficiency S89

K Successive solid phase peptidomimetic triazole ligations S104

L Subsequent solid supported enzymatic glycosylation S115

<u>Abbreviations</u>:

AscNa: (+)-Sodium L-ascorbate
BSA: Bovine Serum albumin
CAPS: 3-(Cyclohexylamino)-1-propanesulfonic acid
CMP-NeuAc: Cytidine 5'-monophospho-*N*-acetylneuraminci acid
CPG: Controlled Pore Glass
DMAP: 4-(Dimethylamino)pyridine
DMF: *N*,*N*-Dimethylformamide
DTT: DL-Dithiothreitol
EDC: *N*-Ethyl-*N′*-(3-dimethylaminopropyl)carbodiimide
EDTA: Ethylenediaminetetraacetic acid
FITC: Fluorescein isothiocyanate isomer I
Fmoc-PAL-OH: 5-[3,5-Dimethoxy-4-(Fmoc-aminomethyl)phenoxy]pentanoic acid
Fmoc-TTDS-OH: *N*1-(9- Fmoc)-1,13-diamino-4,7,10-trioxatridecan-succinamic acid
GalNAc-T1: Polypeptide α-*N*-acetylgalactosaminyl transferase T1
HATU:1-[*Bis*(dimethylamino)methylene]-1*H*-1,2,3-triazolo[4,5-b]pyridinium 3-oxide hexafluorophosphate
HCTU: 1-[*Bis*(dimethylamino)methylen]-5-chlorobenzotriazolium 3-oxide hexafluorophosphate
HEPES: 4-(2-Hydroxyethyl)piperazine-1-ethanesulfonic acid
HOAt: 3*H*-[1,2,3]-Triazolo[4,5-*b*]pyridin-3-ol
***m*CPBA**: 3-Chloroperoxybenzoic acid
MES: 2-(*N*-Morpholino)ethanesulfonic acid
MPAA: 4-Mercaptophenylacetic acid
N_3Val-OH: (*S*)-2-Azido-3-methylbutanoic acid
NMP: 1-Methyl-2-pyrrolidone
PEG: Poly(ethylene glycol)
PBS: Phosphate buffered saline
ST6GalNAc I: GalNAc α -2,6-sialyltransferase 1
TBAF: Tetrabutylammonium fluoride
TCEP: *Tris*(2-carboxyethyl)phosphine
TFA: Trifluoroacetic acid
THPTA: *Tris*(3-hydroxypropyltriazolylmethyl)amine
TIPS: Triisopropylsilane
TRIS: Tris(hydroxymethyl)aminomethane
UDP-GalNAc: Uridine[5′]diphospho[1](2-acetamido-2-deoxy-α-D-galactopyranose)
VVL: *Vica villosa* lectin

A General information

Unless stated otherwise, all reagents and anhydrous solvents were used without further purification. Protected amino acids, Rink amide ChemMatrix® resin, HCTU were purchased from Merck Biosciences (Nottingham, UK). Controlled Pore Glass beads (TRISOPERL®, pore size: 100 nm, bead diameter: 100 -200 µm, lot number: PG L 14/04 AMINO 225) was obtained from VitraBio (Steinach, Germany). SPRINbeads AH130 was obtained from SPRIN technologies (Trieste, Italy). [N1-(9-Fluorenylmethoxycarbonyl)-1,13-diamino-4,7,10-trioxatridecan-succinamic acid (Fmoc-TTDS-OH, CAS Number: 172089-14-4) and Fmoc-NH-PEG-COOH (3000Da) were purchased from Iris Biotech GmbH (Marktredwitz, Germany). Peptide synthesis grade DMF and HATU were obtained from Applied Biosystems (Courtaboeuf, France). Ultrapure water was prepared using a Milli-Q water system from Millipore (Molsheim, France). All other chemicals were from Sigma Aldrich (St-Quentin-Fallavier, France) and solvents from SDS-Carlo Erba (Val de Reuil, France) and were used without any further purification. Polypropylene syringes fitted with polypropylene frits were obtained from Torviq (Niles, MI, USA) and were equipped with PTFE stopcock bought from Chromoptic (Courtaboeuf, France).

(S)-2-azido-3-methylbutanoic acid (N_3Val-OH)[1] was prepared using previously described protocols. Its ^{1}H NMR spectra matched the respective literature data.

All the CuAAC reactions were performed under a strict argon atmosphere, using solvents that had been freshly deoxygenated through ten successive vacuum (15 mbar)/argon cycles.

A.1 Characterization and purification of the building blocks

Flash column chromatography purifications were carried out using Kiesegel C60 (Merck, Germany) as the stationary phase, and thin layer chromatography analyses were performed on precoated silica gel plates (0.25 mm thick, 60 F_{254}, Merck, Germany) and observed under UV light at 254 nm then stained with a standard basic potassium permanganate solution.

Purifications by reverse phase column chromatography were carried out on the Interchim SPOT II Flash, equipped with CHROMABOND® Flash RS 40, 43 g C_{18} ec (flow rate: 40 mL/min)

^{1}H and ^{13}C NMR spectra were recorded on Bruker AV 500 and AV 600 instrument, at a constant temperature of 25 °C. Chemical shifts are reported in parts per million from low to high field. Coupling constants (*J*) are reported in hertz (Hz). Standard abbreviations indicating multiplicity were used as follows: s = singlet, d = doublet, t = triplet, m = multiplet, b = broad.

Specific rotation [α] are reported in (deg·mL)/(g·dm) and corresponding concentration *c* in g/100 mL.

[1] E. D. Goddard-Borger, R. V. Stick, *Org. Lett.* **2007**, *9*, 3797–3800.

A.2 Peptide characterization and purification

The peptides were analyzed by HPLC and High resolution ESI-MS mass spectrometry. HPLC analyses and semi-preparative purifications were carried out on LaChrom Elite system consisting of a Hitachi L-2130 pump, a Hitachi L-2455 diode array detector and a Hitachi L-2200 autosampler. The machines were equipped with Nucleosil C18 reversed-phase columns, 300 Å, 5 µm, 250 x 4.6 mm (flow rate: 1 mL/min) or Chromolith® High Resolution RP-18e, 150 Å, 10 x 4.6 mm (flow rate: 3 mL/min) for analysis and Nucleosil C18 reversed-phase columns 300 Å, 5 µm, 250 x 10 mm for purification (flow rate: 3 mL/min). Solvents A and B were 0.1% TFA in H_2O and 0.1% TFA in MeCN, respectively.

The Electron-transfer dissociation (ETD) experiments were performed on a HCTultra PTM Discovery System (Bruker Daltonics) ion trap mass spectrometer equipped with an electrospray ion source, at the concentration of 10 µM into a 45:54.8:0.2 CH_3CN/H_2O/formic acid solution. The acquisitions were carried out at standard/enhanced resolution (8100 *m*/*z* per second) from *m*/*z* 100 to 3000 for MS and MS/MS. Interpretation of the ETD spectra was performed with Biotools 3.2 software (Bruker Daltonics).

High resolution ESI-MS analyses and LC/MS were performed on a maXis ultra-high-resolution Q-TOF mass spectrometer (Bruker Daltonics, Bremen, Germany). LC was carried out on Ultimate® 3000 RSLC system (Dionex, Germering, Germany). The machine was equipped with Zorbax 300 SB-C18 RRHD, 1.8 µm, 2.1 x 100 mm (flow rate: 300 µL/min). Solvents A and B were 0.1% formic acid in H_2O and 0.8% formic acid in MeCN, respectively.

The observed *m/z* correspond to the monoisotopic ions except when stated otherwise.

A.3 General procedures for solid-phase peptide synthesis

Solid-phase peptide synthesis (SPPS) was run on an automated 433A synthesizer from Applied Biosystem using Fmoc/*t*Bu chemistry at a 0.1 mmol scale with HCTU as coupling reagent and Rink amide ChemMatrix® as solid support. The elongation was carried out automatically using a 10-fold excess of protected amino acids and HCTU; and 20-fold excess of $i\mathrm{Pr_2NEt}$. The side chain protecting groups used were Arg(Pbf), Asp(*t*Bu), His(Trt), Ser(*t*Bu), Thr(*t*Bu). Fmoc deprotection was performed using a 20% piperidine solution in DMF. The 0.1 mmol scale program purchased from the manufacturer was used, with a single coupling (unless stated otherwise) followed by capping with acetic anhydride. The crude peptide was released from the resin with TFA/$i\mathrm{Pr_3SiH}$/1,3 dimethoxybenzene (90:5:5) for 2 h, and the peptide was precipitated with ice-cold diethyl ether, recovered by centrifugation and washed 3 times with diethyl ether.

A.4 Preparation of the linkersSynthesis of the Pipoc-derived linker[2]

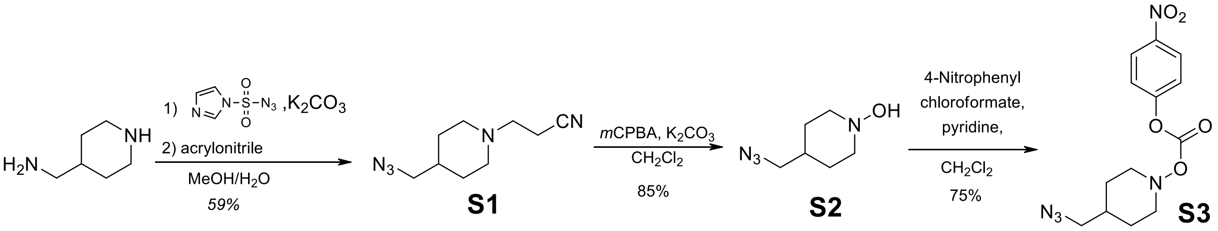

Supplementary scheme S1 - Synthesis of 4-(azidomethyl)piperidin-1-yl 4-nitrophenyl carbonate

• 3-[4-(Azidomethyl)piperidin-1-yl]propanenitrile (S1)

A solution of 4-(aminomethyl)piperidine (1.15 g, 10 mmol) and K_2CO_3 (2.76 g, 20 mmol, 2 equiv.) in MeOH/H_2O (1:1, 40 mL) was cooled in an ice bath. 3-Azidosulfonyl-3*H*-imidazol-1-ium hydrogen sulfate[3] (2.98 g, 11 mmol, 1.1 equiv.) was added portionwise and the reaction mixture was stirred overnight at room temperature. The reaction mixture was cooled down in an ice bath and acrylonitrile (1.31 mL, 20 mmol, 2 equiv.) was added dropwise over 30 min. The resulting mixture was stirred at room temperature for 4 h then volatiles were evaporated under reduced pressure. The resulting suspension was diluted with water (50 mL) and extracted with EtOAc (4 x 40 mL). The combined organic layers were dried over $MgSO_4$, filtrated then concentrated under reduced pressure. Purification by flash column chromatography (eluent: pet. ether/EtOAc 2:8) afforded compound **S1** (1.14 g, 59%) as a colorless liquid.

^{1}H NMR (500 MHz, $CDCl_3$): δ = 3.17 (d, *J* = 6.8 Hz, 2H), 2.94 – 2.86 (m, 2H), 2.68 (t, *J* = 7.1 Hz, 2H), 2.49 (t, *J* = 7.1 Hz, 2H), 2.06 (ddd, *J* = 11.7, 11.7, 2.5 Hz, 2H), 1.77 – 1.70 (m, 2H), 1.61 – 1.48 (m, 1H), 1.37 – 1.24 (m, 2H).

^{13}C NMR (125 MHz, $CDCl_3$): δ = 119.1, 57.2, 53.8, 53.0, 36.2, 29.9, 16.2.

ESI-HRMS (*m/z*): $[M+H]^+$ calcd for $C_9H_{16}N_5$: 194.1406, found: 194.1403.

---

[2] For examples of Pipoc-derived linkers used in solid supported organic synthesis see: Xiao, M. P. Nova, A. W. Czarnik, *J. Comb. Chem.* **1999**, *1*, 379–382.

[3] N. Fischer, E. D. Goddard-Borger, R. Greiner, T. M. Klapötke, B. W. Skelton, J. Stierstorfer, *J. Org. Chem.* **2012**, *77*, 1760–1764.

Copy of the ^{1}H NMR spectrum (500 MHz, $CDCl_3$):

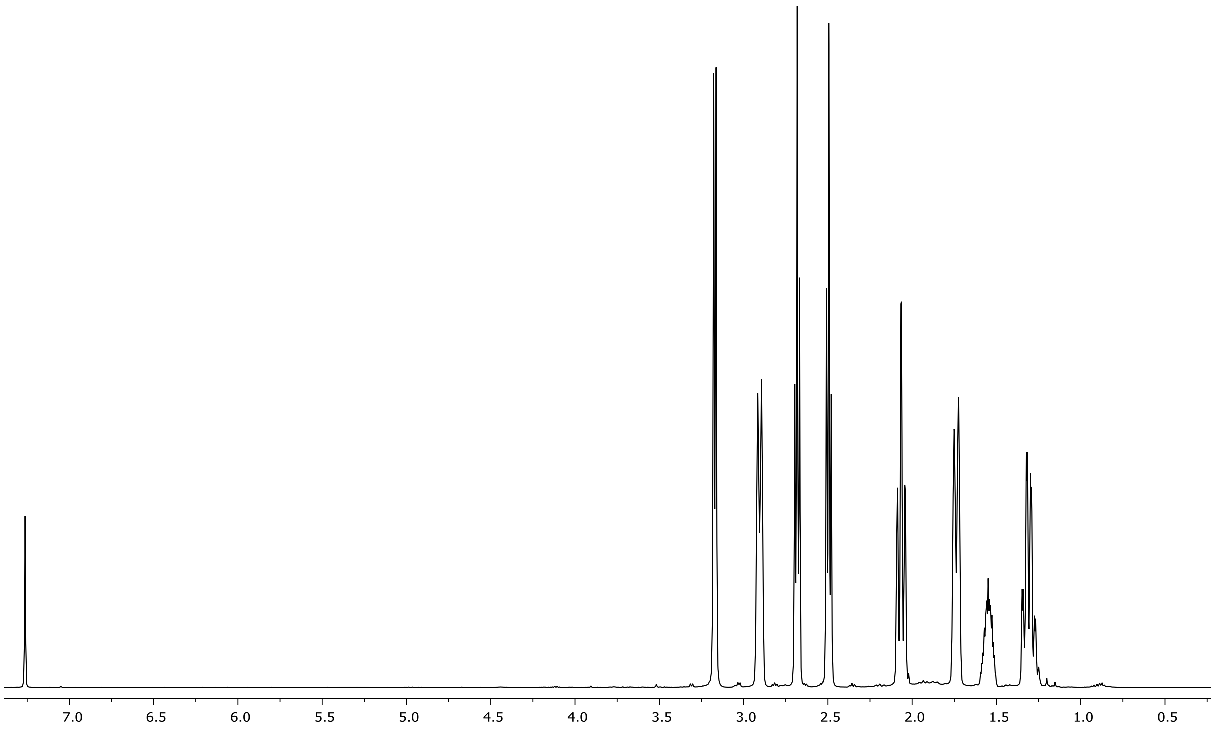

Copy of the ^{13}C NMR spectrum (125 MHz, $CDCl_3$):

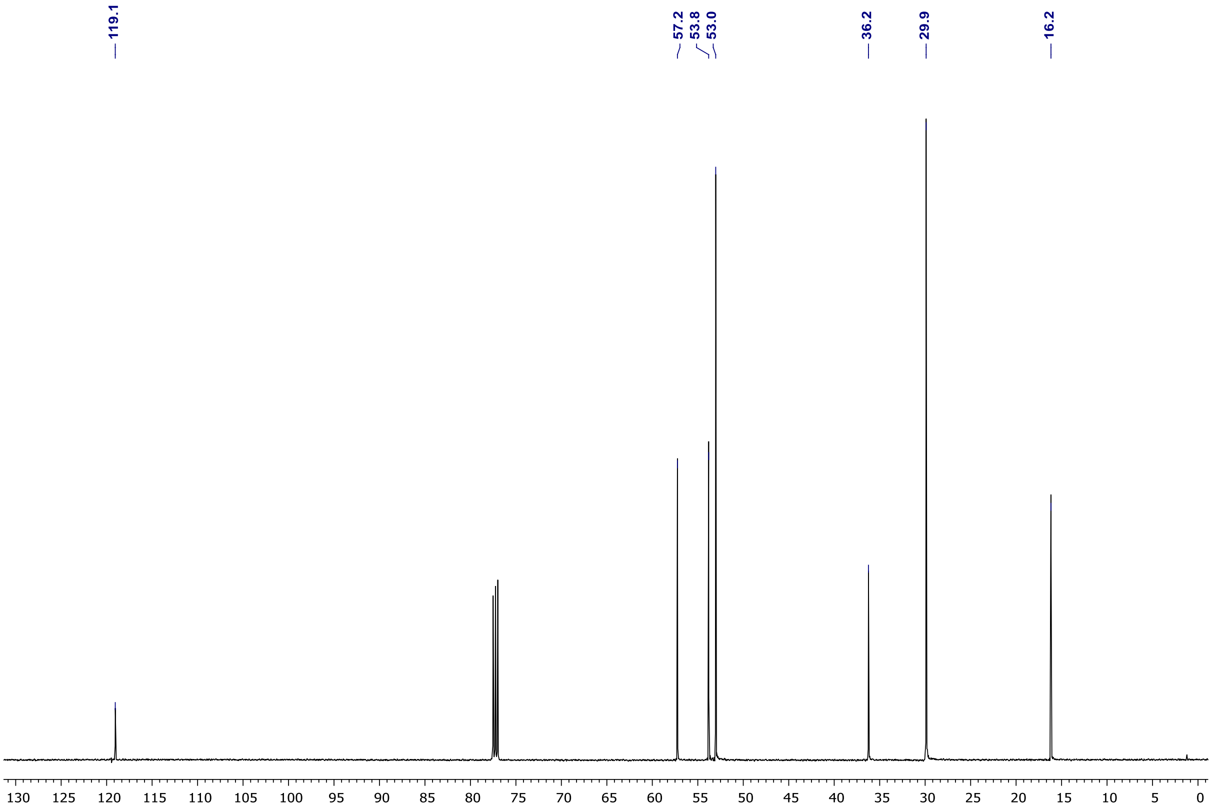

• **4-(Azidomethyl)piperidin-1-ol (S2)**

A solution of compound **S1** (1 g, 5.2 mmol) in CH_2Cl_2 (50 mL) was cooled to -78 °C. *m*CPBA (84% purity, 1.17 g, 5.7 mmol, 1.1 equiv.) and K_2CO_3 (1.08 g, 7.8 mmol, 1.5 equiv.) were added. The solution was stirred for 3 h then allowed to warm up to room temperature and stirred for an additional 16 h at room temperature. The resulting suspension was then filtrated through a pad of $MgSO_4$ and then concentrated under reduced pressure. Purification by flash column chromatography (eluent: EtOAc) afforded compound **S2** (690 mg, 85%) as a colorless liquid.

^{1}H NMR* (500 MHz, $CDCl_3$): δ = 3.36 – 3.29 (m, 2H), 3.16 (d, *J* = 6.7 Hz, 2H), 2.46 (ddd, *J* = 12.9, 10.2, 2.7 Hz, 2H), 1.86 – 1.78 (m, 2H), 1.60 – 1.51 (m, 1H), 1.41 – 1.29 (m, 2H).

^{13}C NMR (125 MHz, $CDCl_3$): δ = 57.2, 55.7, 34.3, 28.5.

ESI-HRMS (*m/z*): $[M+H]^+$ calcd for $C_6H_{13}N_4O$: 157.1089, found: 157.1089.

***NMR description of the major conformer, a minor one (~15%) being observed as broad peaks in the ^{1}H spectrum

Copy of the ^{1}H NMR spectrum (500 MHz, $CDCl_3$):

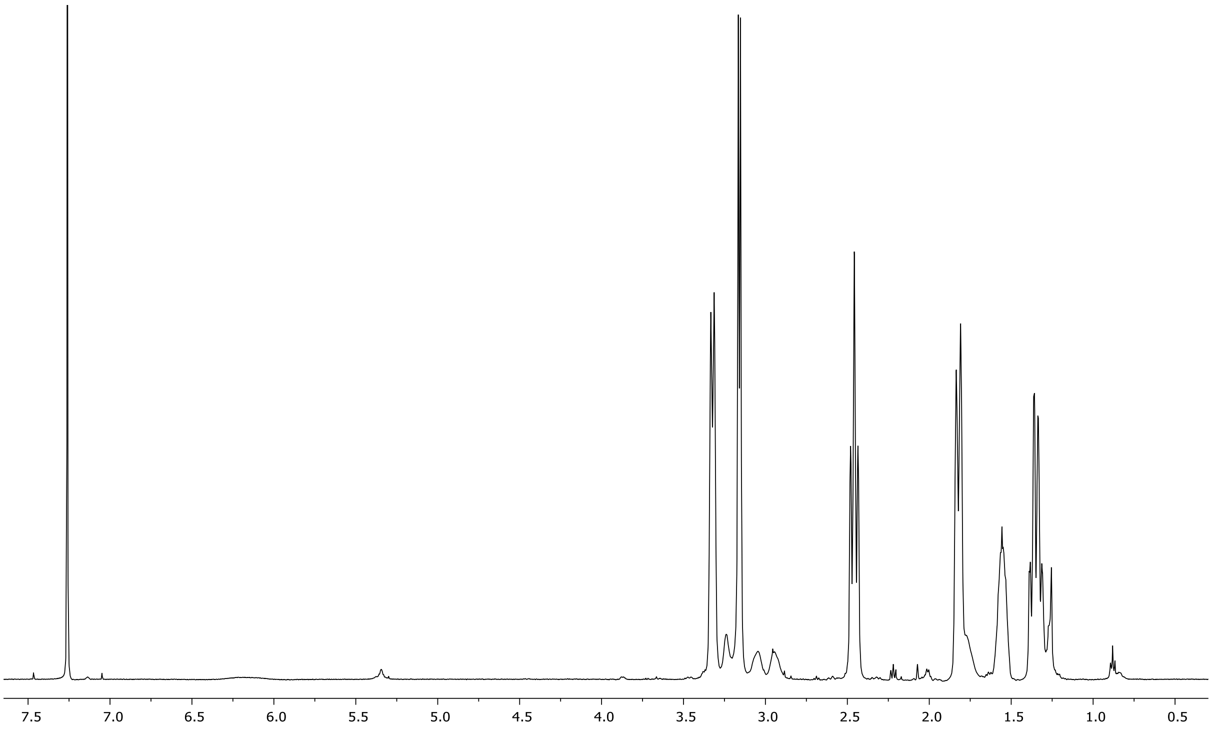

Copy of the ^{13}C NMR spectrum (125 MHz, $CDCl_3$):

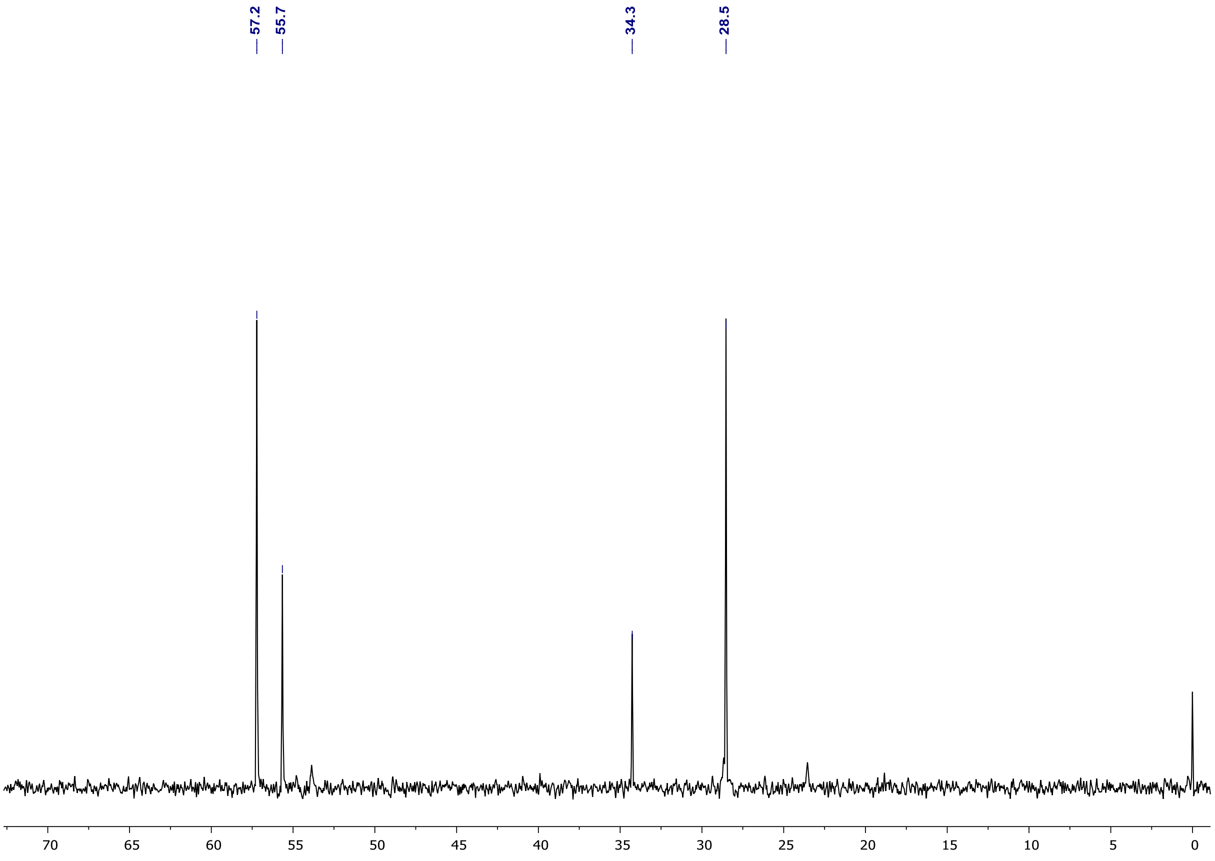

• **4-(Azidomethyl)piperidin-1-yl 4-nitrophenyl carbonate (S3)**

A solution of compound **S2** (690 mg, 4.4 mmol) and pyridine (3.5 mL, 44.2 mmol, 10 equiv.) in CH_2Cl_2 (20 mL) was cooled in an ice bath and *p*-nitrophenyl chloroformate (980 mg, 4.9 mmol, 1.1 equiv.) was added. The resulting solution was stirred at room temperature for 30 min then washed with a saturated aqueous $NaHCO_3$ solution (3 x 10 mL), dried over $MgSO_4$, filtrated then concentrated under reduced pressure. Purification by flash column chromatography (eluent: pet. ether/EtOAc 6:4) afforded compound **S3** (1.06 g, 75%) as a white amorphous solid.

^{1}H NMR* (500 MHz, $CDCl_3$): δ = 8.28 (d, *J* = 9.1 Hz, 2H), 7.40 (d, *J* = 8.6 Hz, 2H), 3.69 – 3.50 (m, 2H), 3.32 – 3.11 (m, 2H), 2.82 – 2.73 (m, 2H), 1.98 – 1.83 (m, 2H), 1.74 – 1.52 (m, 3H).

^{13}C NMR (125 MHz, $CDCl_3$): δ = 155.5, 152.0, 145.6, 125.5, 121.8, 56.9, 56.4, 35.3, 29.3.

ESI-HRMS (*m/z*): $[M+H]^+$ calcd for $C_{13}H_{16}N_5O_5$: 322.1151, found: 322.1147.

***NMR description of the major conformer, a minor one (~15%) being observed as broad peaks in the ^{1}H spectrum

Copy of the ^{1}H NMR spectrum (500 MHz, $CDCl_3$):

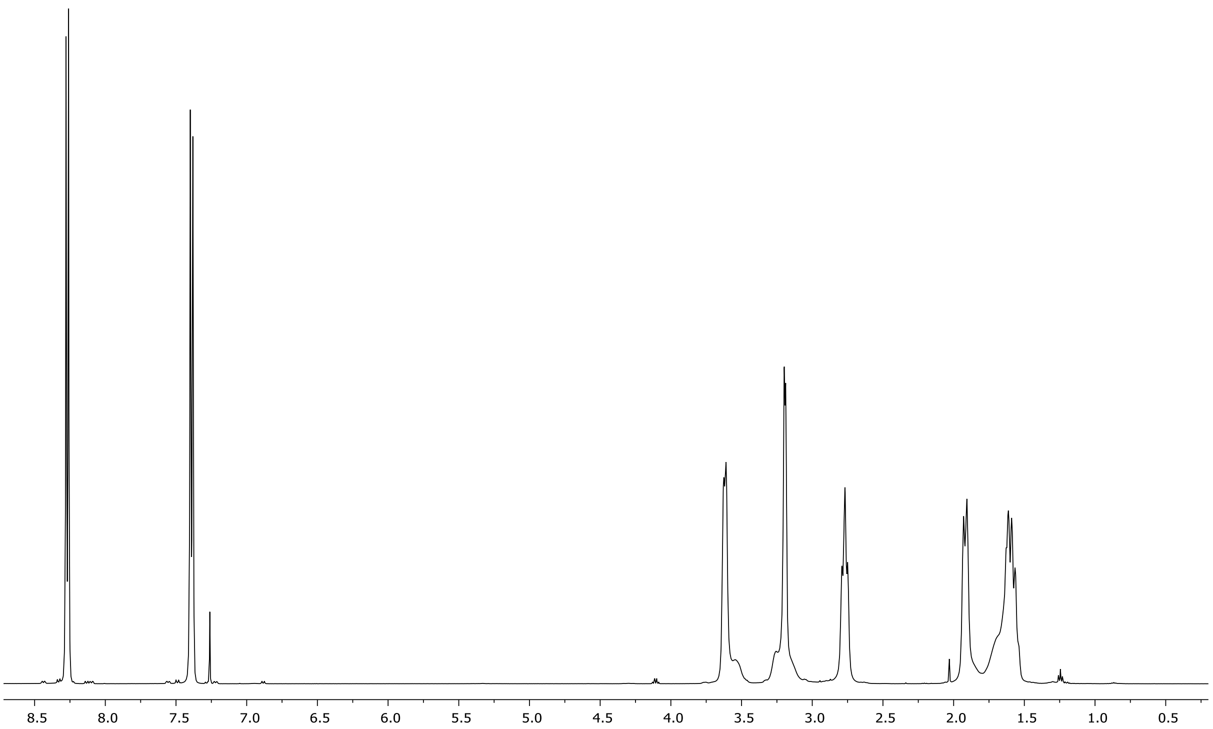

Copy of the ^{13}C NMR spectrum (125 MHz, $CDCl_3$):

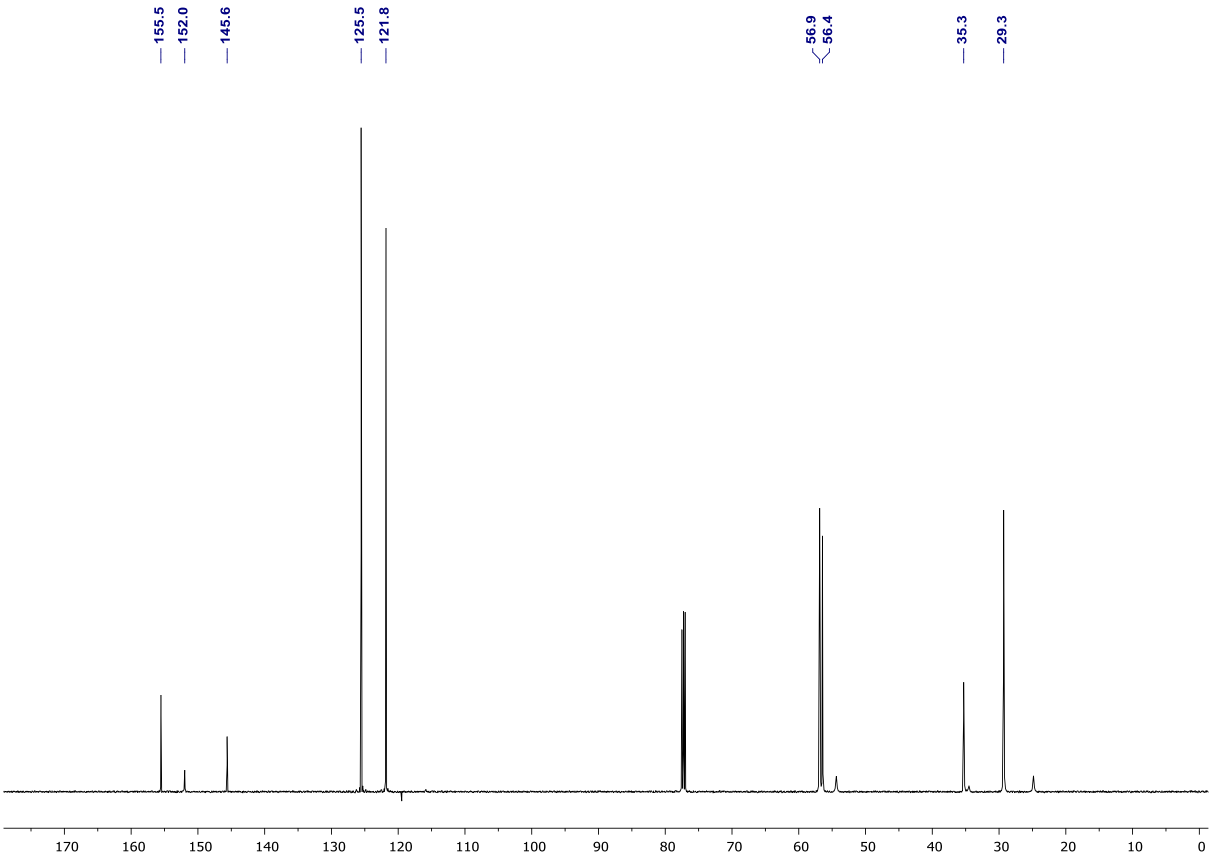

A.5 Synthesis of the Dde-derived linker[4]

• 2-(5-Azido-1-hydroxypentylidene)-5,5-dimethylcyclohexane-1,3-dione (S4)

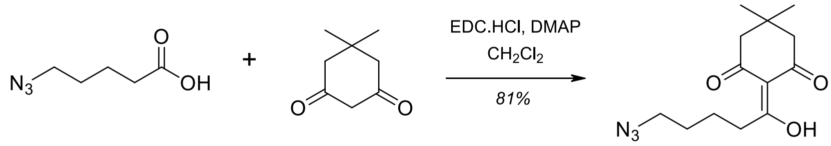

S4

Supplementary scheme S2 - Synthesis of 2-(5-azido-1-hydroxypentylidene)-5,5-dimethylcyclohexane-1,3-dione

A solution of 5-azidopentanoic acid (2.00 g, 14 mmol) and dimedone (2.55 g, 18 mmol, 1.3 equiv.) in CH_2Cl_2 (50 mL) was cooled in an ice bath. DMAP (2.22 g, 18 mmol, 1.3 equiv.) then EDC hydrochloride (2.82 g, 14.7 mmol, 1.05 equiv.) were added. The reaction mixture was stirred at room temperature for 16 h, then washed with a 1 M HCl aqueous solution (3 x 50 mL), dried over $MgSO_4$, filtrated then concentrated under reduced pressure. Purification by flash column chromatography (eluent: pet. ether/EtOAc 92:8) afforded compound **S4** (1.14 g, 75%) as a colorless oil.

^{1}H NMR (500 MHz, $CDCl_3$): δ = 3.31 (t, *J* = 6.4 Hz, 2H), 3.07 (t, *J* = 6.9 Hz, 2H), 2.54 (s, 2H), 2.35 (s, 2H), 1.77 – 1.62 (m, 4H), 1.08 (s, 6H).

^{13}C NMR (125 MHz, $CDCl_3$): δ = 204.9, 197.5, 195.2, 112.0, 52.6, 51.2, 46.7, 39.9, 30.7, 28.5, 28.2, 21.7.

ESI-HRMS (*m/z*): $[M+H]^+$ calcd for $C_{13}H_{20}N_3O_3$: 266.1505, found: 266.1503.

[4] For examples of Dde-derived linkers used in solid supported organic synthesis see: a) S. R. Chhabra, A. N. Khan, B. W. Bycroft, *Tetrahedron Lett.* **1998**, *39*, 3585–3588. b) P. J. Murray, C. Kay, J. J. Scicinski, S. C. McKeown, S. P. Watson, R. A. E. Carr, *Tetrahedron Lett.* **1999**, *40*, 5609–5612.c) S. R. Chhabra, H. Parekh, A. N. Khan, B. W. Bycroft, B. Kellam, *Tetrahedron Lett.* **2001**, *42*, 2189–2192.

Copy of the ^{1}H NMR spectrum (500 MHz, $CDCl_3$):

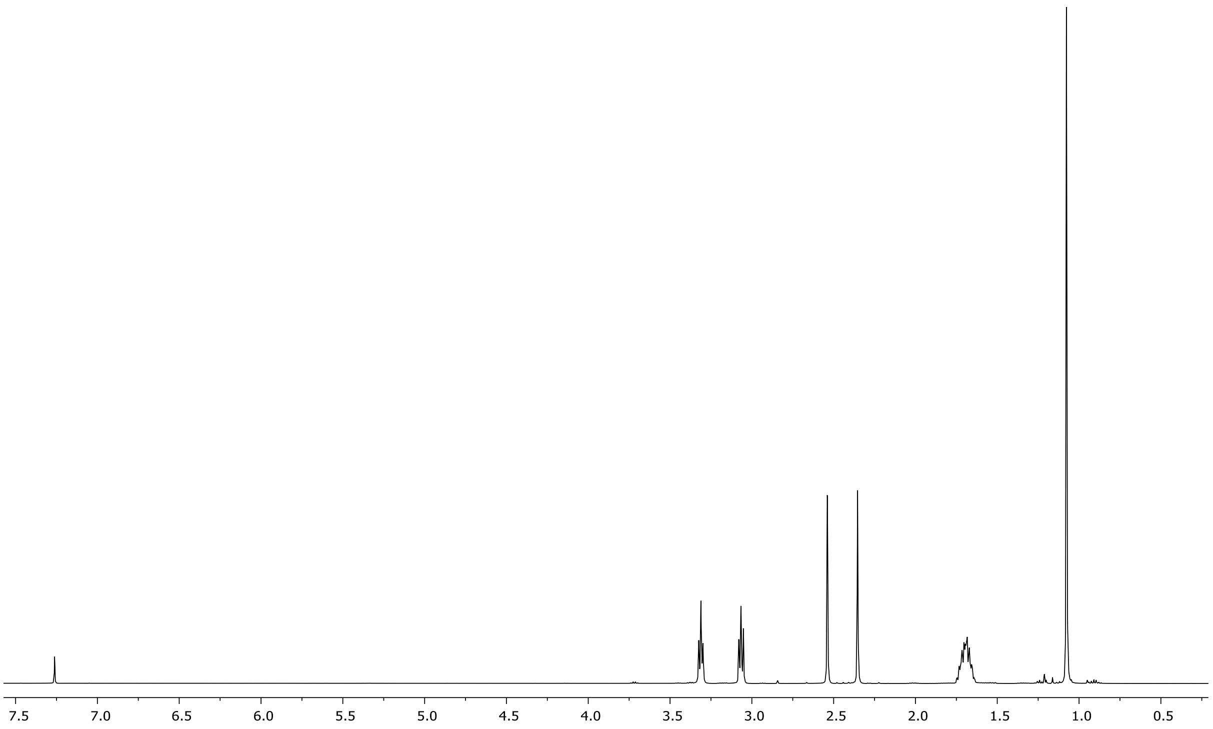

Copy of the ^{13}C NMR spectrum (125 MHz, $CDCl_3$):

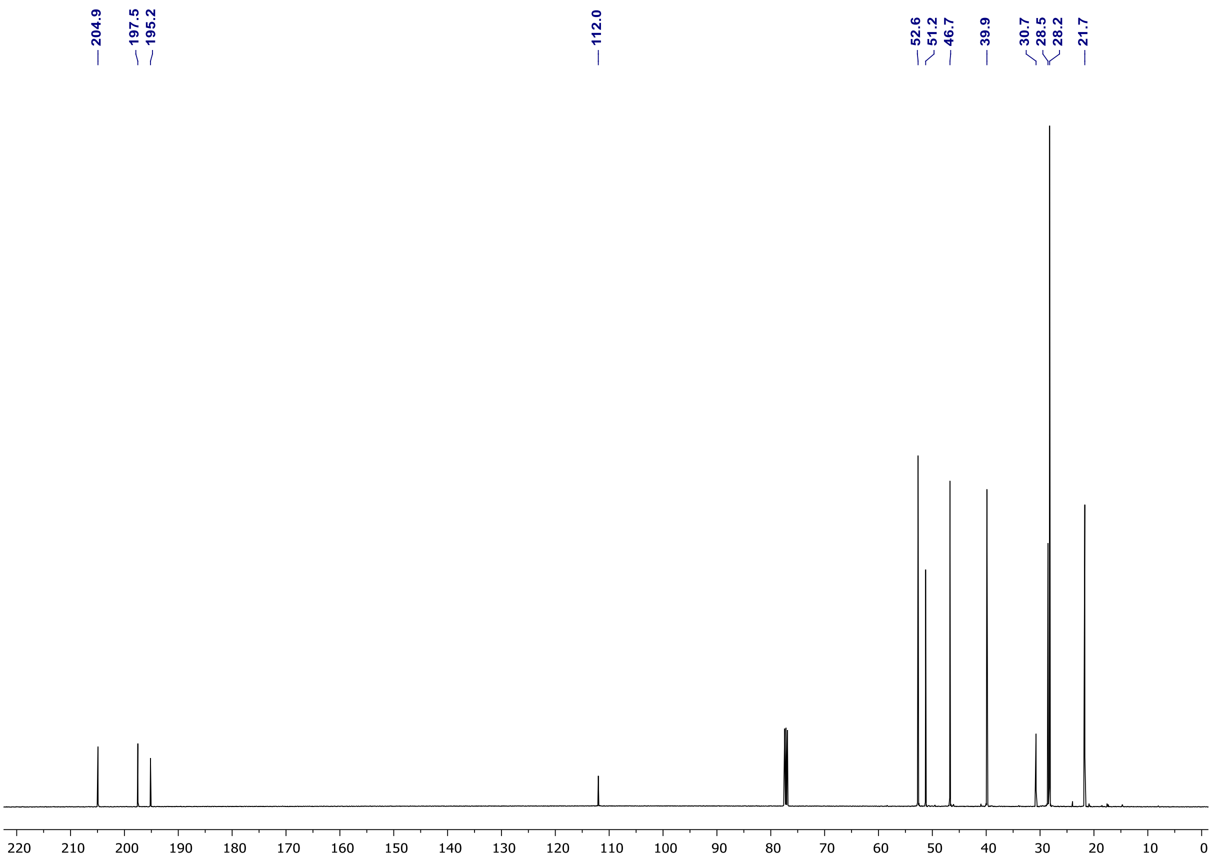

A.6 Synthesis of N_3-Dtpp linker[5]

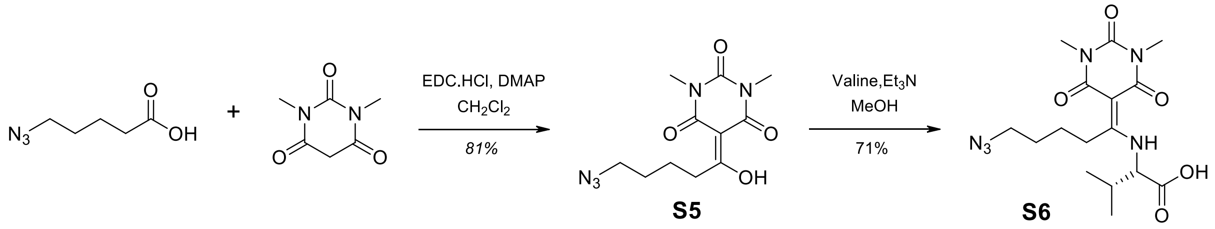

Supplementary scheme S3 - Synthesis of N_3-Dtpp-Val-OH (**S6**)

• N_3-Dtpp-OH (S5)

A solution of 1,3-dimethylbarbituric acid (3.12 g, 20 mmol) and 5-azidopentanoic acid (3.45 g, 24 mmol, 1.2 equiv.) in CH_2Cl_2 (100 mL) was cooled in an ice bath. DMAP (2.93 g, 24 mmol, 1.2 equiv.) then EDC hydrochloride (4.60 g, 24 mmol, 1.2 equiv.) were added. The reaction mixture was stirred at room temperature for 16 h, then washed with a 1 M HCl aqueous solution (3 x 50 mL), dried over $MgSO_4$, filtrated then concentrated under reduced pressure. Recrystallization from MeOH afforded the product **S5** (4.52 g, 81%) as a white solid.

^{1}H NMR (600 MHz, $CDCl_3$): δ = 17.60 (s, 1H), 3.38 (s, 3H), 3.36 – 3.32 (m, 5H), 3.21– 3.15 (m, 2H), 1.84 – 1.76 (m, 2H), 1.75 – 1.68 (m, 2H).

^{13}C NMR (151 MHz, $CDCl_3$): δ 198.8, 169.8, 160.9, 150.3, 95.3, 51.0, 36.1, 28.6, 28.1, 27.9, 22.8.

ESI-HRMS (*m/z*): $[M+H]^+$ calcd for $C_{11}H_{16}N_5O_4$: 282.1202, found: 282.1197.

M.p. 42 °C

[5] For examples of DTPM-derived linkers used in solid supported organic synthesis see: E. T. da Silva, R. A. S. S. Gil, S. Caldarelli, F. Ziarelli, E. L. S. Lima, *J. Braz. Chem. Soc.* **2011**, *22*, 86–91.

Copy of the ^{1}H NMR spectrum (600 MHz, $CDCl_3$):

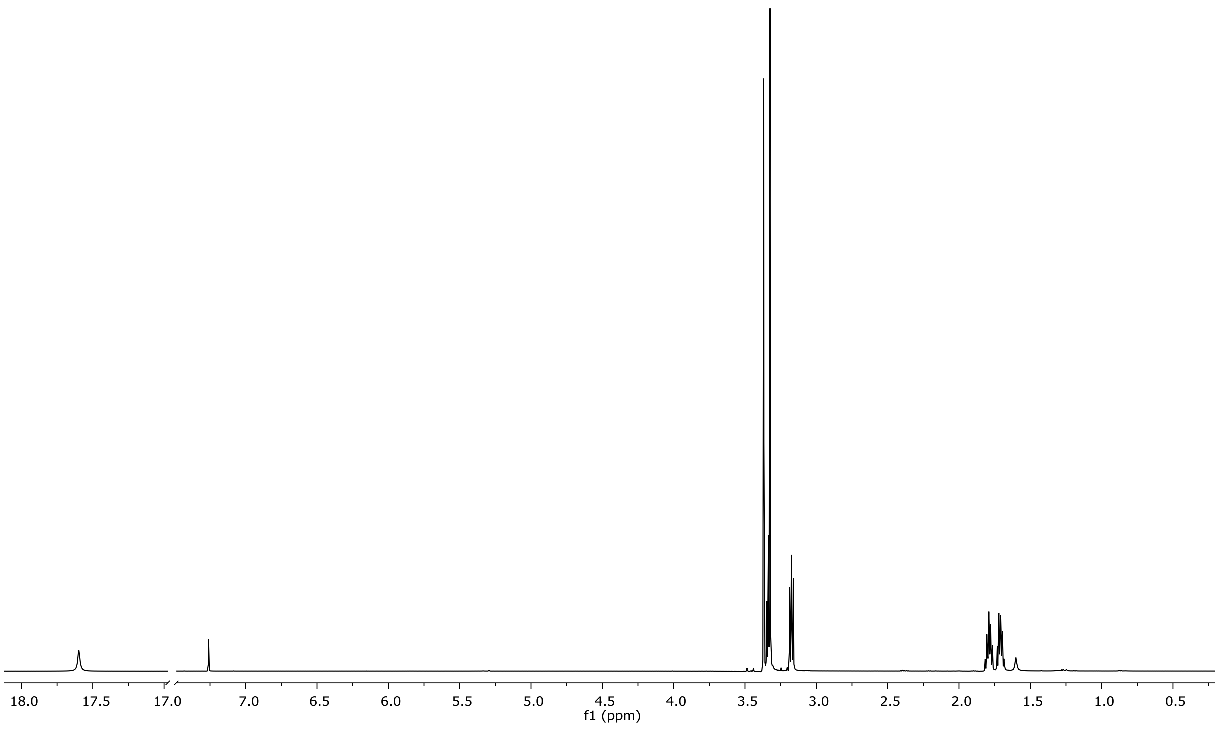

Copy of the ^{13}C NMR spectrum (151 MHz, $CDCl_3$):

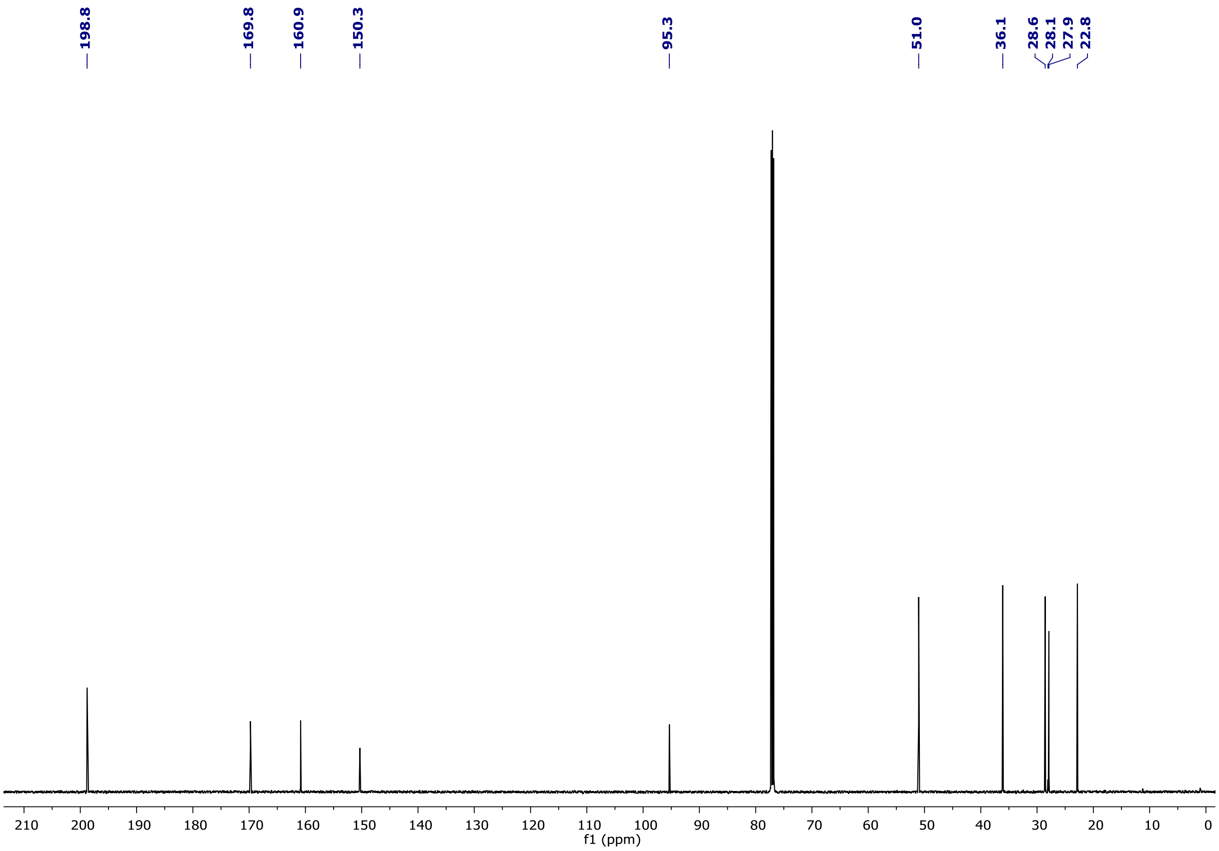

• **N_3-Dtpp-Val-OH (S6)**

A solution of compound **S5** (844 mg, 3 mmol), valine (703 mg, 6 mmol, 2 equiv.) and triethylamine (1.25 mL, 9 mmol, 3 equiv.) in MeOH (60 mL) was heated at reflux for 24 h. The reaction mixture was cooled down to room temperature then volatiles were evaporated under reduced pressure. The resulting suspension was diluted with CH_2Cl_2 (150 mL) and washed with a 1 M HCl aqueous solution (3 x 30 mL), dried over $MgSO_4$, filtrated then concentrated under reduced pressure. Purification by reverse phase column chromatography (eluent: H_2O/CH_3CN 8:2 then 6:7) afforded product **S6** (810 mg, 71%) as a white amorphous solid.

^{1}H NMR (600 MHz, $CDCl_3$): δ = 13.06 (d, J = 9.0 Hz, 1H), 4.32 (dd, J = 9.0, 4.9 Hz, 1H), 3.37 (t, J = 6.6 Hz, 2H), 3.34 (s, 3H), 3.31 (s, 3H), 3.21 - 3.09 (m, 1H), 3.08 – 2.95 (m, 1H), 2.49 – 2.36 (m, 1H), 1.84 – 1.71 (m, 2H), 1.71 – 1.55 (m, 2H), 1.13 (d, J = 6.9 Hz, 3H), 1.09 (d, J = 6.8 Hz, 3H).

^{13}C NMR (151 MHz, $CDCl_3$) δ 175.8, 172.9, 165.9, 161.4, 150.3, 89.5, 60.4, 49.7, 30.6, 28.2, 27.8, 27.1, 26.9, 23.9, 18.1, 16.7.

ESI-HRMS (*m/z*): $[M+H]^+$ calcd for $C_{16}H_{25}N_6O_5$: 381.1886, found: 381.1880.

UV (MeOH) λ_{max} = 303 nm, ε^{303} = 27000 M^{-1} cm^{-1}, ε^{280} = 8650 M^{-1} cm^{-1}

$[\alpha]_D^{20}$ +8.7 (*c* 1 in MeOH)

Copy of the ^{1}H NMR spectrum (600 MHz, $CDCl_3$):

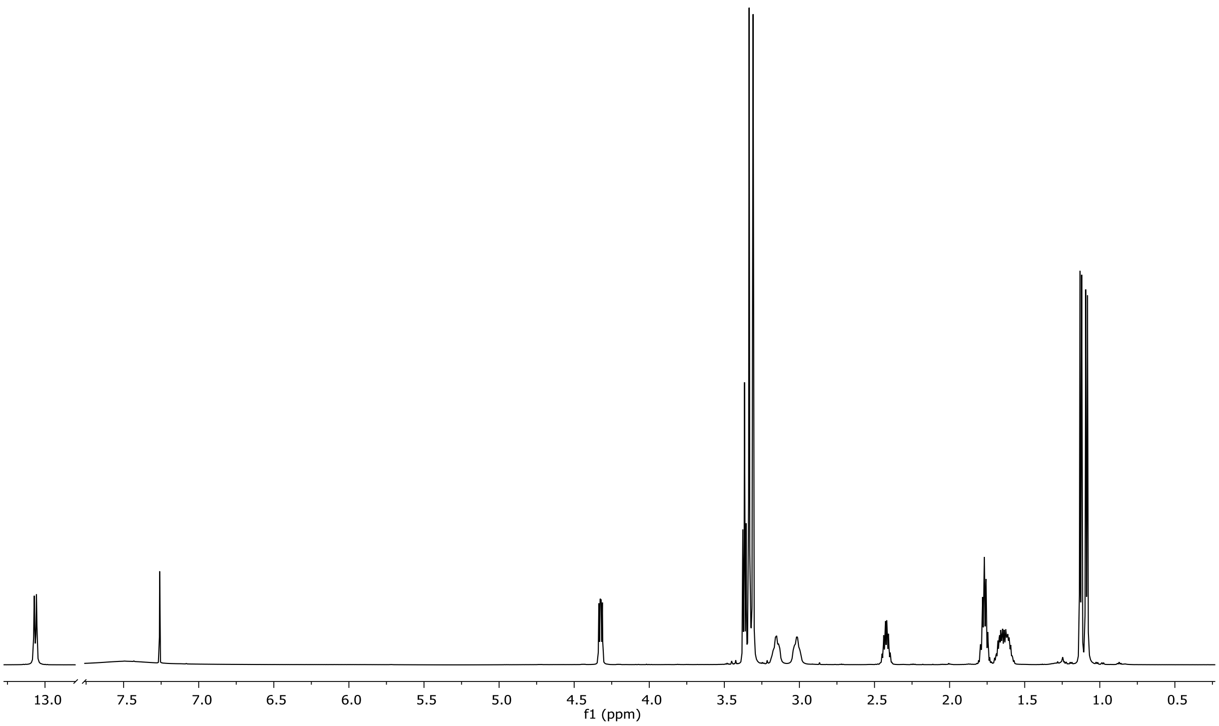

Copy of the ^{13}C NMR spectrum (151 MHz, $CDCl_3$):

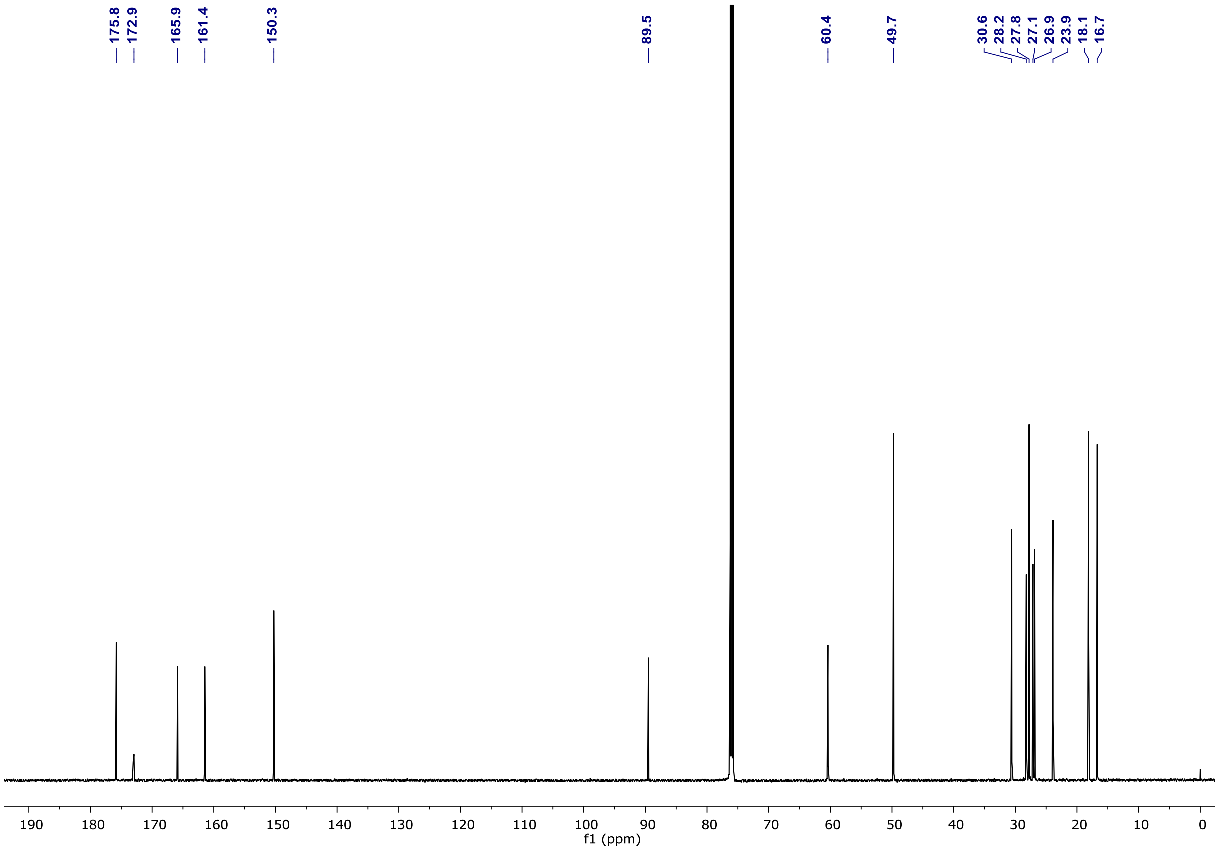
175.8
172.9
165.9
161.4
150.3
89.5
60.4
49.7
30.6
28.2
27.8
27.1
26.9
23.9
18.1
16.7
f1 (ppm)

B Preparation of the model linker-containing peptides 1-4

B.1 Solid-phase synthesis of solid-supported Muc1 peptide resin S7

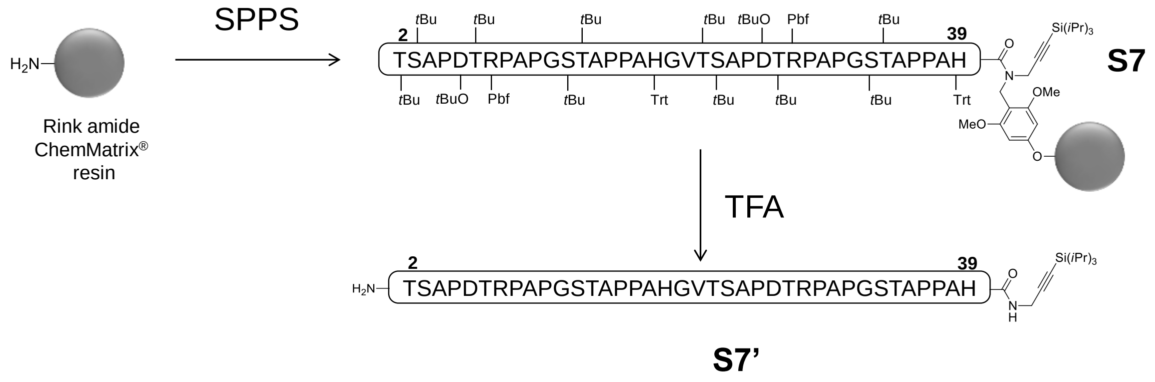

Supplementary scheme S4 - Solid-phase synthesis of solid-supported Muc1 peptide resin **S7**

The backbone amide linker 4-(4-formyl-3,5-dimethoxyphenoxy)butyric acid, (67 mg, 0.25 mmol, 2.5 equiv.) was loaded on Rink amide ChemMatrix® resin (192 mg, 0.52 mmol/g, 0.1 mmol) using HATU (95 mg, 0.25 mmol, 2.5 equiv.) and *i*Pr_2NEt (87 µL, 0.5 mmol, 5 equiv.) in DMF for 2 h at room temperature. The resin was then thoroughly washed with DMF. In case of incomplete reaction (positive Kaiser's test[6]), was repeated once.
3-(Triisopropylsilyl)prop-2-yn-1-amine (106 mg, 0.5 mmol, 5 equiv.) and $NaBH_3CN$ (32 mg, 0.5 mmol, 5 equiv.) dissolved in 5 mL of DMF/MeOH/AcOH (7:2:1) were added to the resin (1 equiv., swelled in DMF prior to the reaction), and the resulting suspension was mixed overnight at 50 °C with N_2 bubbling.
The resin was then thoroughly washed with DMF and CH_2Cl_2. Fmoc-His(Trt)-OH (620 mg, 1 mmol, 10 equiv.) was coupled onto the resulting resin with HATU (380 mg, 1 mmol, 10 equiv.) and *i*Pr_2NEt (348 µL, 2 mmol, 20 equiv.) in DMF overnight at room temperature.
The elongation of the peptide was performed by standard automated solid phase synthesis (p S4) up to ^{2}Thr. Prolines 4, 5, 24 and 25 were coupled twice following the automated program for double couplings. A 85% elongation yield was obtained, determined by the titration of the first (BAL) and last (^{2}Thr) Fmoc group deprotection (UV, 301 nm).
A few beads of the resulting resin **S7** were cleaved to give crude peptide **S7'** which was analyzed by LC/HRMS.

ESI-HRMS (*m/z*): $[M+H]^+$ calcd for $C_{165}H_{264}N_{49}O_{52}Si$: 3791.9289, found: 3791.9177.

[6] Kaiser, E.; Colescott, R. L.; Bossinger, C. D.; Cook, P. I. *Anal. Biochem.* **1970**, *34*, 595-598.

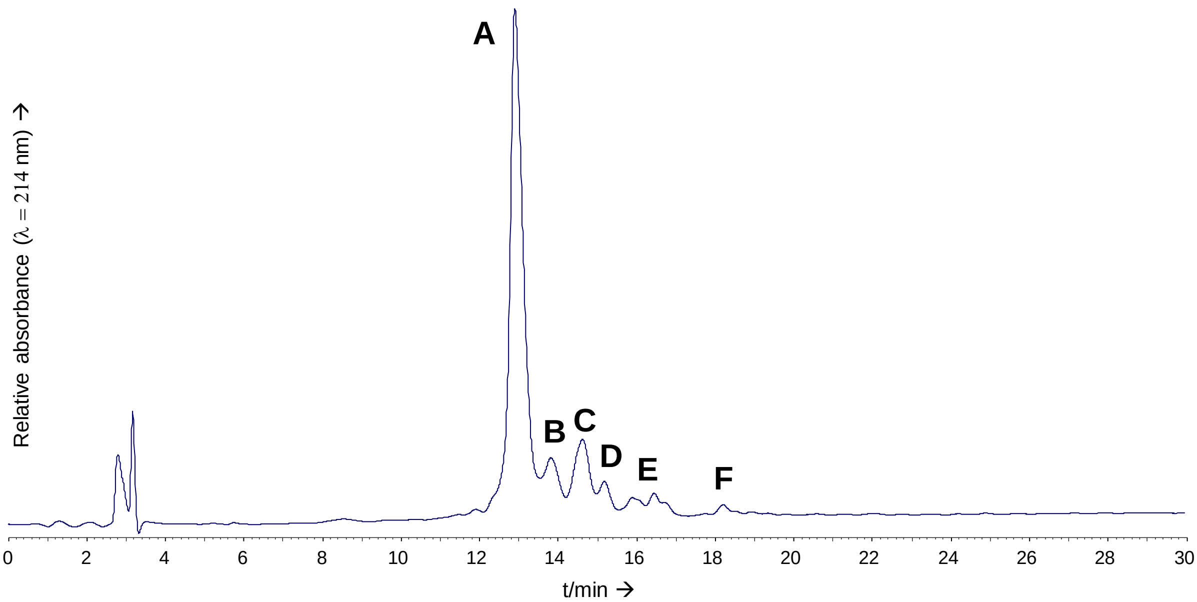

Supplementary figure S1 - Analytical HPLC profile of crude **S7'**. Column: Nucleosil®; gradient: from 30% to 60% B over 30 min.

Peak	Found *m/z*	Attributed to	Calculated $[M+H]^+$
A	3791.9177	H-[2-39]	3791.9289 ($C_{165}H_{264}N_{49}O_{52}Si$)
B	3574.8192	Ac-[5-39]	3574.8227 ($C_{157}H_{249}N_{46}O_{48}Si$)
C	3362.7356	Ac-[7-39]	3362.7430 ($C_{148}H_{237}N_{44}O_{44}Si$)
	3732.8789	Ac-[3-39]	3732.8918 ($C_{163}H_{259}N_{48}O_{51}Si$)
	3261.6876	Ac-[8-39]	3261.6953 ($C_{144}H_{230}N_{43}O_{42}Si$)
	3645.8594	Ac-[4-39]	3645.8598 ($C_{160}H_{254}N_{47}O_{49}Si$)
D	2840.4448	Ac-[12-39]	2840.4516 ($C_{125}H_{199}N_{36}O_{38}Si$)
	2783.4244	Ac-[13-39]	2783.4301 ($C_{123}H_{196}N_{35}O_{37}Si$)
	2937.4944	Ac-[11-39]	2937.5043 ($C_{130}H_{206}N_{37}O_{39}Si$)
	3105.5868	Ac-[9-39]	3105.5942 ($C_{138}H_{218}N_{39}O_{41}Si$)
	2696.3931	Ac-[14-39]	2696.3981 ($C_{120}H_{191}N_{34}O_{35}Si$)
	3008.5329	Ac-[10-39]	3008.5414 ($C_{133}H_{211}N_{38}O_{40}Si$)
E	1966.0157	Ac-[22-39]	1966.0218 ($C_{87}H_{141}N_{24}O_{26}Si$)
	1864.9684	Ac-[23-39]	1864.9742 ($C_{83}H_{134}N_{23}O_{24}Si$)
	2122.1073	Ac-[20-39]	2122.1117 ($C_{94}H_{153}N_{26}O_{28}Si$)
	1494.8202	Ac-[27-39]	1494.8253 ($C_{68}H_{112}N_{19}O_{17}Si$)
	1609.8478	Ac-[26-39]	1609.8523 ($C_{72}H_{117}N_{20}O_{20}Si$)
	1393.7730	Ac-[28-39]	1393.7776 ($C_{64}H_{105}N_{18}O_{15}Si$)
	1777.9367	Ac-[24-39]	1777.9421 ($C_{80}H_{129}N_{22}O_{22}Si$)
	1706.8987	Ac-[25-39]	1706.9050 ($C_{77}H_{124}N_{21}O_{21}Si$)
F	2065.0824	Ac-[21-39]	2065.0903 ($C_{92}H_{150}N_{25}O_{27}Si$)
	1237.6726	Ac-[29-39]	1237.6765 ($C_{58}H_{93}N_{14}O_{14}Si$)
	1140.6208	Ac-[30-39]	1140.6238 ($C_{53}H_{86}N_{13}O_{13}Si$)
	915.5092	Ac-[33-39]	915.5124 ($C_{43}H_{71}N_{10}O_{10}Si$)

B.2 Synthesis of peptide 1

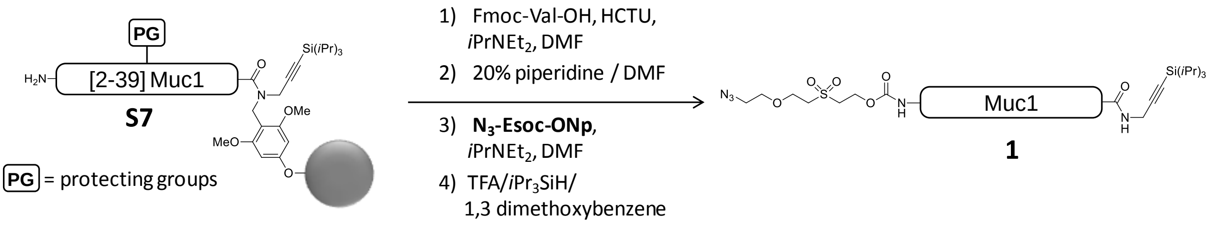

Supplementary scheme S5 - Solid-phase synthesis of peptide **1**

The coupling and Fmoc deprotection of the N-terminal Val residue were performed by automated SPPS on the peptide resin **S7** (5 µmol). The activated carbonate N_3-Esoc-ONp[7] (5 equiv., dissolved in DMF) and *i*Pr_2NEt (5 equiv.) were then added to the peptide resin (1 equiv., swelled in DMF prior to the reaction), and the resulting suspension was stirred overnight at room temperature. The resin was then thoroughly washed with DMF. The resin was further washed with CH_2Cl_2, and treated with TFA/*i*Pr_3SiH/1,3 dimethoxybenzene (90:5:5) for 2 h. Peptide **1** was precipitated by dilution with ice-cold diethyl ether, recovered by centrifugation, washed 3 times with diethyl ether then dried under reduced pressure. The crude mixture was purified by semi-preparative HPLC to yield peptide **1** as a white fluffy powder (7.4 mg, 1.6 µmol, 32%* isolated yield based on the original resin loading).

*: estimated considering a MW = 4595 g/mol taking into account 4 trifluoroacetate counter-anions, as predicted from the sequence: 2Arg, 2His = +4 overall charge. Water content is not taken into account.

ESI-HRMS (*m/z*): $[M+H]^+$ calcd for $C_{177}H_{284}N_{53}O_{58}SSi$: 4140.0393, found: 4140.0350.

[7] V. Aucagne, I. E. Valverde, P. Marceau, M. Galibert, N. Dendane, A. F. Delmas, *Angew.Chem. Int. Ed.* **2012**, *51*, 11320–11324.

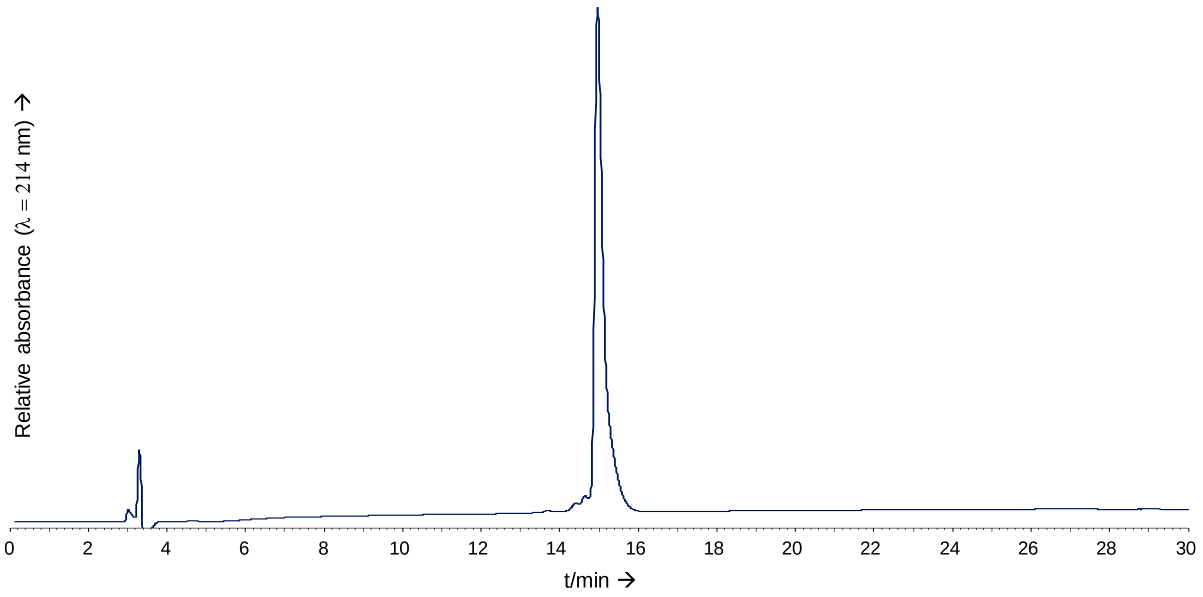

<u>Supplementary figure S2</u> - Analytical HPLC profile of purified peptide **1**. Column: Nucleosil®; gradient: from 30% to 60% B over 30 min.

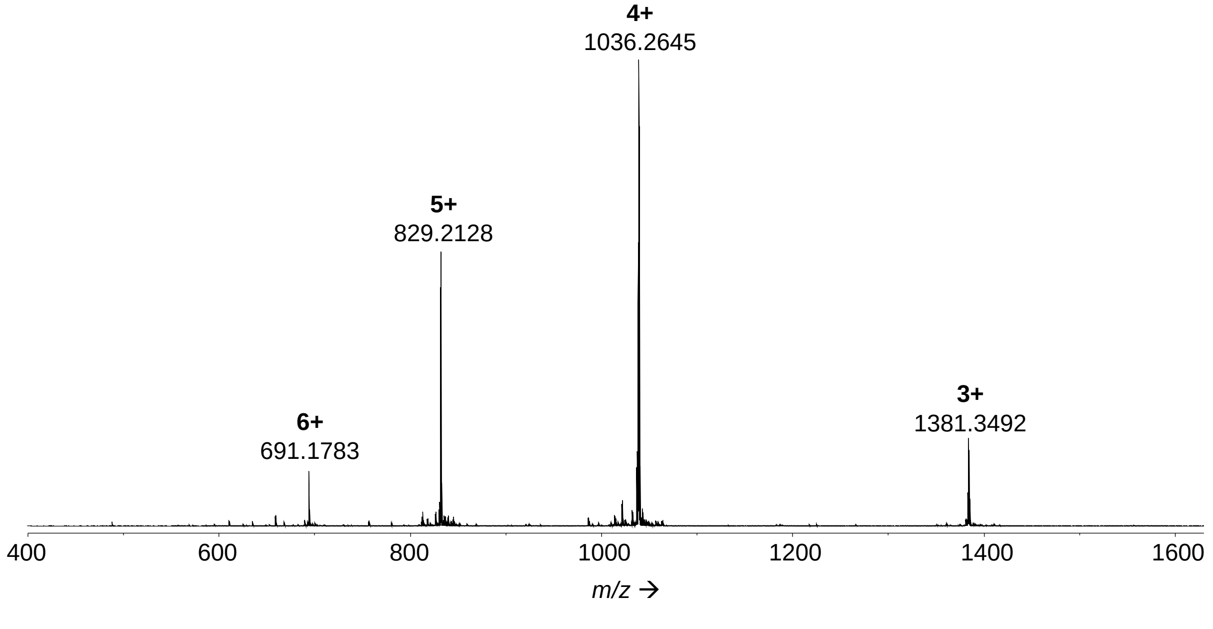

<u>Supplementary figure S3</u> - ESI-HRMS mass spectrum of peptide **1**

B.3 Synthesis of peptide 2

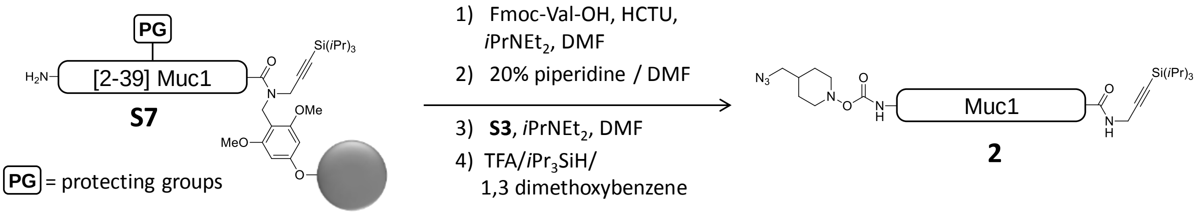

Supplementary scheme S6 - Solid-phase synthesis of peptide **2**

The coupling and Fmoc deprotection of the N-terminal Val residue were performed by automated SPPS on the peptide resin **S7** (5 µmol). The activated carbonate **S3** (5 equiv., dissolved in DMF) and *i*Pr_2NEt (5 equiv.) were then added to the peptide resin (1 equiv., swelled in DMF prior to the reaction), and the resulting suspension was stirred overnight at room temperature. The resin was then thoroughly washed with DMF. The resin was further washed with CH_2Cl_2, and treated with TFA/*i*Pr_3SiH/1,3 dimethoxybenzene (90:5:5) for 2 h. Peptide **2** was precipitated by dilution with ice-cold diethyl ether, recovered by centrifugation, washed 3 times with diethyl ether then dried under reduced pressure. The crude mixture was purified by semi-preparative HPLC to yield the peptide **2** as a white fluffy powder (3.1 mg, 0.7 µmol, 13%* isolated yield based on the original resin loading).

*: estimated considering a MW = 4642 g/mol taking into account 5 trifluoroacetate counter-anions, as predicted from the sequence: Linker, 2Arg, 2His = +5 overall charge. Water content is not taken into account.

ESI-HRMS (*m/z*): $[M+H]^+$ calcd for $C_{177}H_{283}N_{54}O_{55}Si$: 4073.0777, found: 4073.0768.

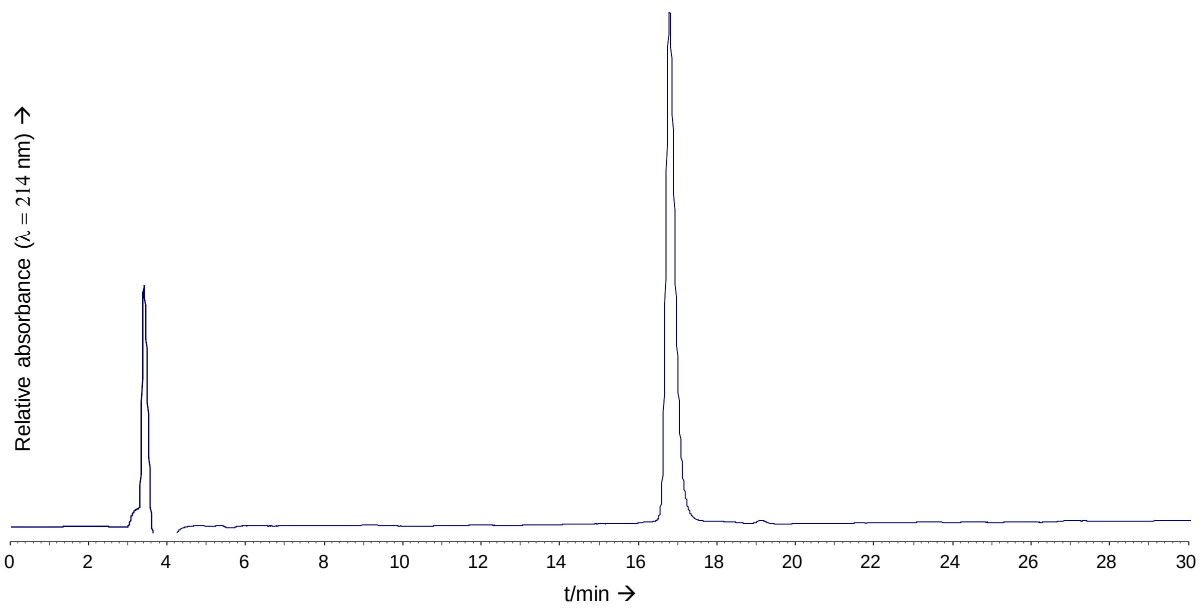

<u>Supplementary figure S4</u> - Analytical HPLC profile of purified peptide **2**. Column: Nucleosil®; gradient: from 30% to 60% B over 30 min.

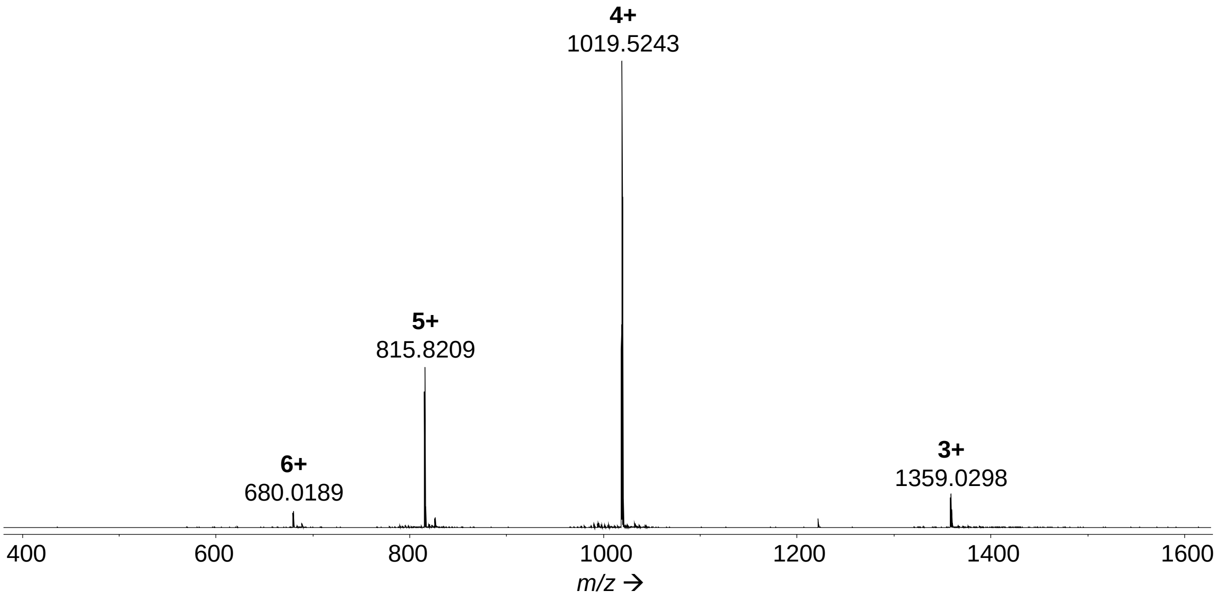

<u>Supplementary figure S5</u> - ESI-HRMS mass spectrum of peptide **2**

B.4 Synthesis of peptide 3

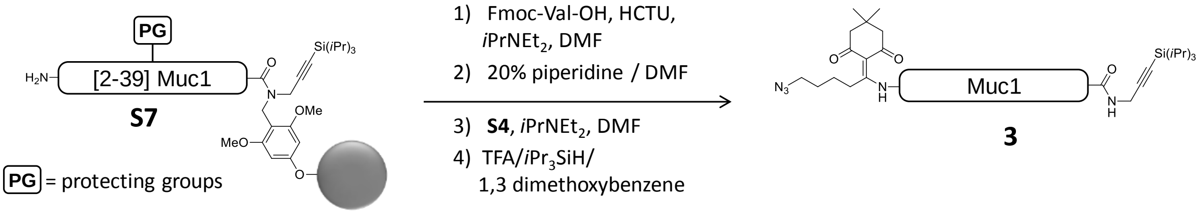

Supplementary scheme S7- Solid-phase synthesis of peptide **3**

The coupling and Fmoc deprotection of the N-terminal Val residue were performed by automated SPPS on the peptide resin **S7** (5 µmol). The compound **S4** (5 equiv., dissolved in DMF) was then added to the peptide resin (1 equiv., swelled in DMF prior to the reaction), and the resulting suspension was stirred overnight at room temperature. The resin was then thoroughly washed with DMF. The resin was further washed with CH_2Cl_2, and treated with TFA/iPr$_3$SiH/1,3 dimethoxybenzene (90:5:5) for 2 h. Peptide **3** was precipitated by dilution with ice-cold diethyl ether, recovered by centrifugation, washed 3 times with diethyl ether then dried under reduced pressure. The crude mixture was purified by semi-preparative HPLC to yield peptide **3** as a white fluffy powder (6.3 mg, 1.4 µmol, 27%* isolated yield based on the original resin loading).

*: estimated considering a MW = 4593 g/mol taking into account 4 trifluoroacetate counter-anions, as predicted from the sequence: 2Arg, 2His = +4 overall charge. Water content is not taken into account.

ESI-HRMS (*m/z*): $[M+H]^+$ calcd for $C_{183}H_{290}N_{53}O_{55}Si$: 4138.1294, found: 4138.1353.

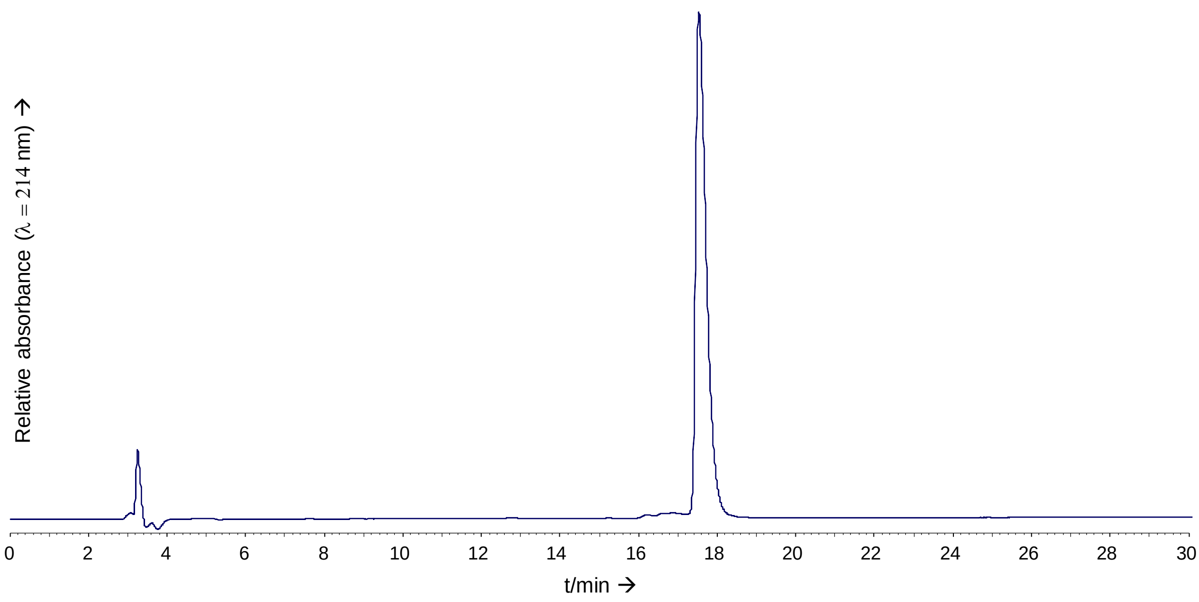

<u>Supplementary figure S6</u> - Analytical HPLC profile of purified peptide **3**. Column: Nucleosil®; gradient: from 30% to 60% B over 30 min.

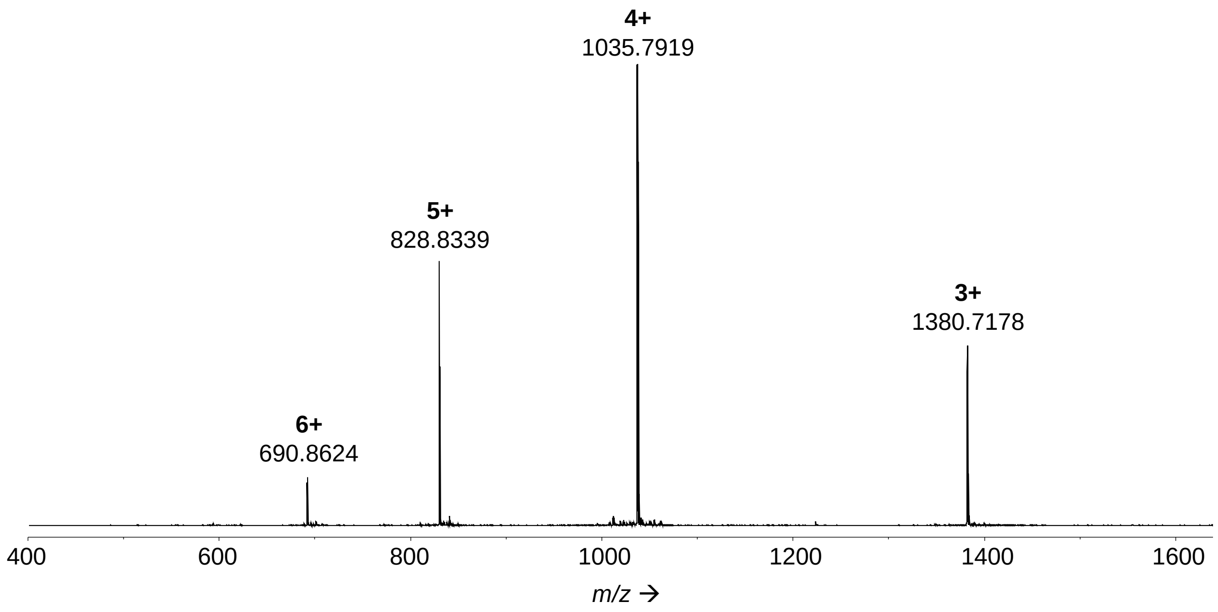

<u>Supplementary figure S7</u> - ESI-HRMS mass spectrum of peptide **3**

B.5 Synthesis of peptide 4

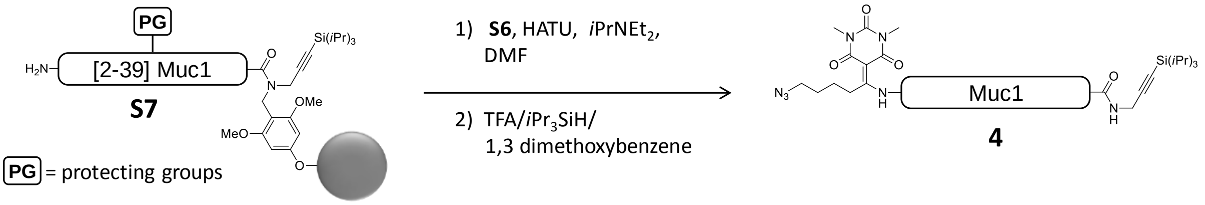

Supplementary scheme S8 - Solid-phase synthesis of peptide **4**

Compound **S6** (2 equiv., dissolved in DMF), HATU (2 equiv.) and *i*Pr_2NEt (3 equiv.) were added to the peptide resin **S7** (5 µmol, 1 equiv., swelled in DMF prior to the reaction), and the resulting suspension was stirred overnight at room temperature. The resin was thoroughly washed with DMF. The resin was further washed with CH_2Cl_2, and treated with TFA/*i*Pr_3SiH/1,3 dimethoxybenzene (90:5:5) for 2 h. Peptide **4** was precipitated by dilution with ice-cold diethyl ether, recovered by centrifugation, washed 3 times with diethyl ether then dried under reduced pressure. The crude mixture was purified by semi-preparative HPLC and lyophilized to yield the peptide **4** as a white fluffy powder (7.1 mg, 1.5 µmol, 31%* isolated yield based on the original resin loading).

*: estimated considering a MW = 4611 g/mol taking into account 4 trifluoroacetate counter-anions, as predicted from the sequence: 2Arg, 2His = +4 overall charge. Water content is not taken into account.

ESI-HRMS (*m/z*): $[M+H]^+$ calcd for $C_{181}H_{286}N_{55}O_{56}Si$: 4154.0992, found: 4154.0768.

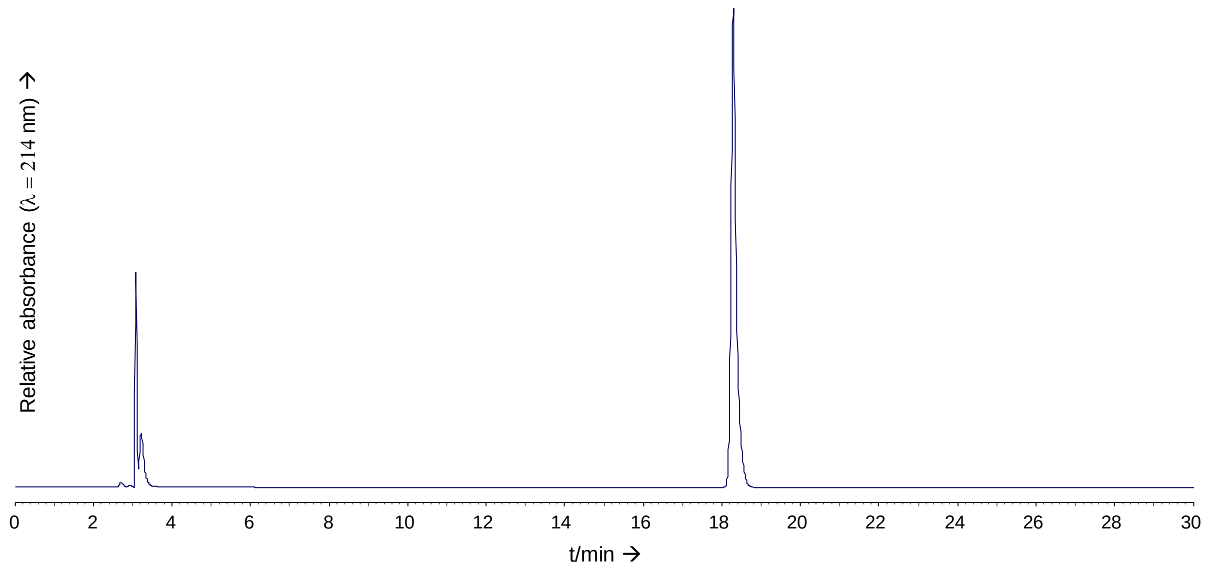

<u>Supplementary figure S8</u> - Analytical HPLC profile of purified peptide **4**. Column: Nucleoside®; gradient: from 30% to 60% B over 30 min.

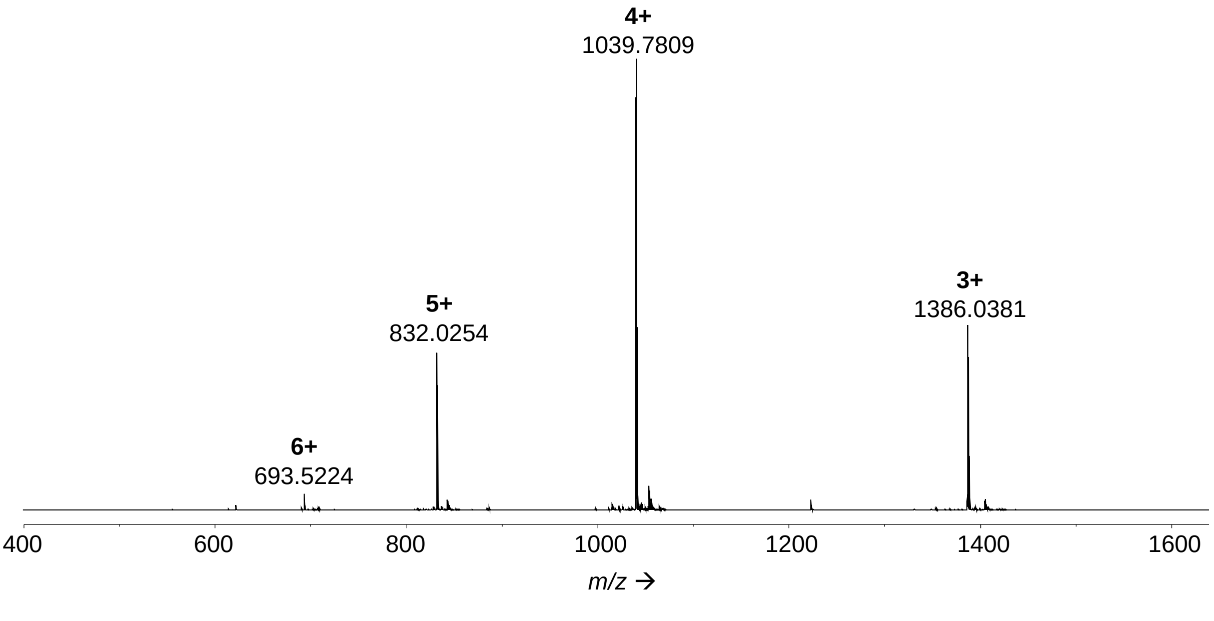

<u>Supplementary figure S9</u> - ESI-HRMS mass spectrum of peptide **4**

- **Synthesis of peptide 4’**

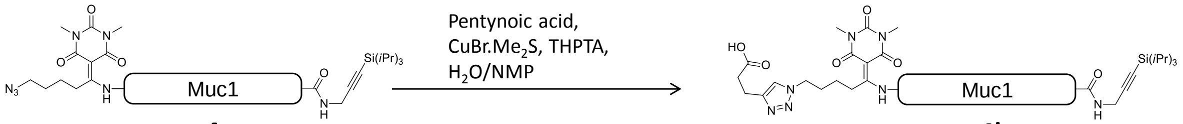

4 **4’**

__Supplementary scheme S9__ - Solid-phase synthesis of peptide **S8**

Peptide **4** (5 µmol) and 4-pentynoic acid (5 mg, 50 µmol, 10 equiv.) were dissolved in 400 µL of a 2:8 mixture of MeOH and 100 mM HEPES buffer pH 7.5. The reaction mixture was introduced in a 2 mL microcentrifuged tube sealed with a rubber septum and was deoxygenated through several successive vacuum (15 mbar) / argon cycles. Then, a mixture containing copper(I) bromide dimethyl sulfide complex (10 equiv.) and THPTA (15 equiv.) dissolved under argon in 50 µL of deoxygenated NMP was added, the resulting mixture was further deoxygenated and was stirred for 1 h at room temperature. Reaction mixture was analyzed by HPLC to check the total consumption of the peptide **4**. The crude mixture was purified by semi-preparative HPLC and lyophilized to yield the peptide **4’** as a white fluffy powder (6.1 mg, 1.3 µmol, 26%* isolated yield based on the original resin loading).

*: estimated considering a MW = 4707 g/mol taking into account 4 trifluoroacetate counter-anions, as predicted from the sequence: 2Arg, 2His = +4 overall charge. Water content is not taken into account.

ESI-HRMS (*m/z*): $[M+H]^+$ calcd for $C_{186}H_{292}N_{55}O_{58}Si$: 4252.1359, found: 4252.1389.

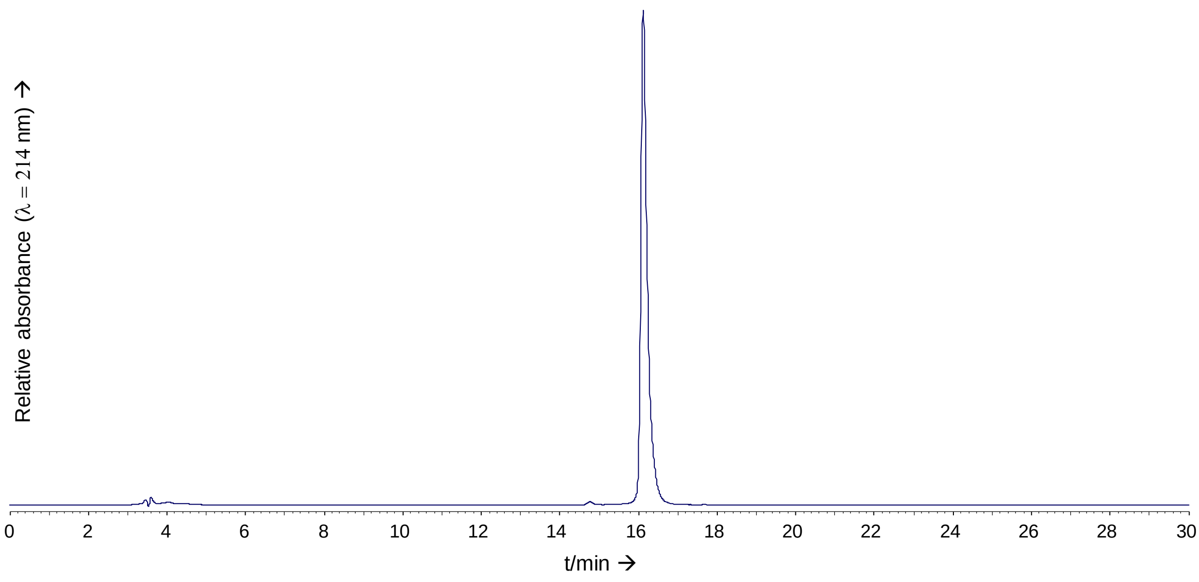

Supplementary figure S10 - Analytical HPLC profile of purified peptide **4'**. Column: Nucleosil®; gradient: from 30% to 60% B over 30 min.

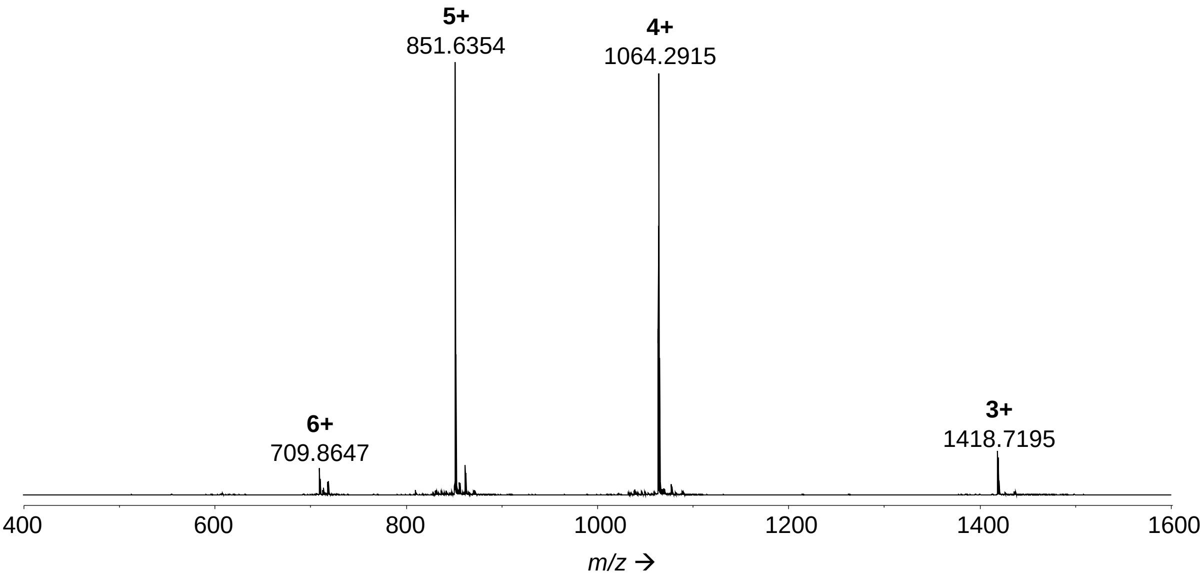

Supplementary figure S11 - ESI-HRMS mass spectrum of peptide **4'**

C Stability study of the linker-containing peptides 1, 2, 3 and 4 under selected conditions

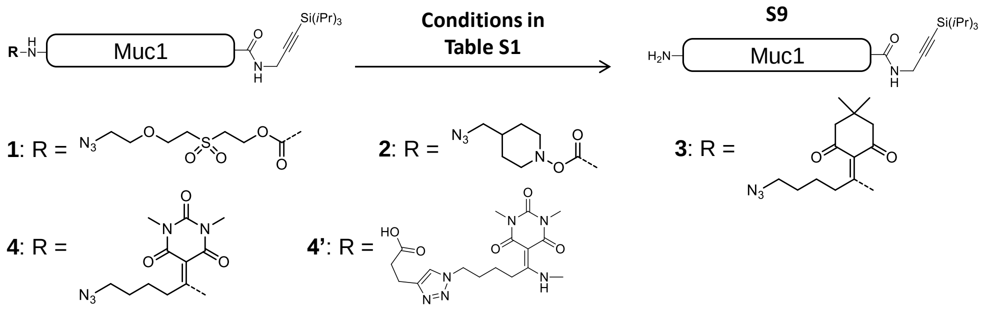

Supplementary scheme S10 - Stability study of the linker-containing peptides **1**, **2**, **3** and **4**

Peptides **1, 2, 3** and **4** (100 µM final peptide concentration) were subjected to a set of varied conditions to evaluate the stability of the four linkers in solution. Stabilities of each linker were followed by analytical HPLC. The amount of remaining intact peptide linker was measured by integration of the HPLC peaks at λ = 214 nm.

			Proportion of intact linker			
**Entry**	**Conditions**	**Time**	**1**	**2**	**3**	**4**
1	TFA cleavage cocktail[a]	2 h	100%	80%	100%	100%
2	0.1% TFA in H_2O (pH 2)	24 h	100%	83%	60%	100%
3	100 mM HEPES buffer (pH 7)	24 h	100%	n.d	79%	100%
3	100 mM HEPES buffer (pH 7)	72 h	100%	n.d	nd	100%
4	100 mM HEPES buffer (pH 8)	24 h	75%	n.d	n.d	98%
5	100 mM CAPS buffer (pH 9)	24 h	10%	n.d	n.d	89%
6	100 mM CAPS buffer (pH 10)	12 h	0%	n.d	n.d	57%
7	100 mM CAPS buffer (pH 12)	1 h	0%	n.d	n.d	0%
8	100 mM TBAF in DMF	2 h	0%	n.d	n.d	100%[b]
9	1% MeONa in MeOH	5 h	0%	n.d	n.d	100%
10	CuAAC[c]	16 h	100%	n.d	n.d	100%
11	Ag^{+}[d]	24 h	100% c	n.d	n.d	100%[b]
12	1 M cysteine, pH 7.5[e]	24h	n.d	n.d	n.d	100%
13	NCL conditions[f]	72 h	100%	n.d	n.d	100%[g]
14	1 M TRIS buffer (pH 7.5)	24h	n.d	n.d	n.d	100%
15	1 M aqueous hydrazine	1 h	95%	n.d	n.d	0%

Supplementary table S1 - Stability study of the linker-containing peptides **1**, **2**, **3** and **4**

Note

[a] TFA/iPr$_3$SiH/1,3 dimethoxybenzene (90:5:5)
[b] deprotection of the TIPS group
[c] 20 mM CuBr.Me_2S, THPTA 30 mM in H_2O/NMP (1:1)
[d] 2 M $AgNO_3$ in H_2O/NMP (1:9)
[e] in 0.1 M phosphate buffer
[f] 25 mM MPAA, 50 mM TCEP, 6 M Gnd.HCl, 200 mM Na_2HPO_4 in H_2O (pH 7)
[g] The experiment was realized with peptide **4'** in order to prevent TCEP reduction of the azide moiety of peptide **4**

C.1 Stability study of the Dde proecting group under aqueous conditions

- **Synthesis of peptide 3'**

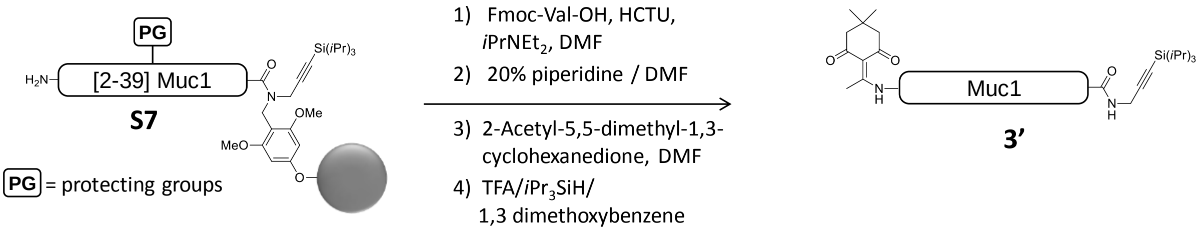

Supplementary scheme S11- Solid-phase synthesis of peptide **3'**

The coupling and Fmoc deprotection of the N-terminal Val residue were performed by automated SPPS on the peptide resin **S7** (1 µmol). 2-Acetyl-5,5-dimethyl-1,3-cyclohexanedione (5 equiv., dissolved in DMF) was then added to the peptide resin (1 equiv., swelled in DMF prior to the reaction), and the resulting suspension was stirred overnight at room temperature. The resin was then thoroughly washed with DMF. The resin was further washed with CH_2Cl_2, and treated with TFA/iPr$_3$SiH/1,3 dimethoxybenzene (90:5:5) for 2 h. Peptide **3'** was precipitated by dilution with ice-cold diethyl ether, recovered by centrifugation, washed 3 times with diethyl ether then dried under reduced pressure. The crude mixture was purified by semi-preparative HPLC to yield peptide **3'** as a white fluffy powder (1.5 mg, 0.33 µmol, 33%* isolated yield based on the original resin loading).

*: estimated considering a MW = 4512 g/mol taking into account 4 trifluoroacetate counter-anions, as predicted from the sequence: 2Arg, 2His = +4 overall charge. Water content is not taken into account.

ESI-HRMS (*m/z*): $[M+H]^+$ calcd for $C_{180}H_{285}N_{50}O_{55}Si$: 4055.0811, found: 4055.0876.

- Stability studies on an N-terminal Dde group under aqueous conditions

Peptide **3'** containing an N-terminal Dde protecting group was subjected to a set of aqueous conditions (100 µM final concentration). Cleavage of Dde was followed by analytical HPLC and measured by integration of the HPLC peaks at λ = 214 nm.

Entry	Conditions	Time	Dde cleavage
1	0.1% TFA in H_2O (pH 2)	24 h	10%
2	100 mM HEPES buffer (pH 7)	24 h	7%

Supplementary table S2 - Cleavage of Dde group under aqueous conditions

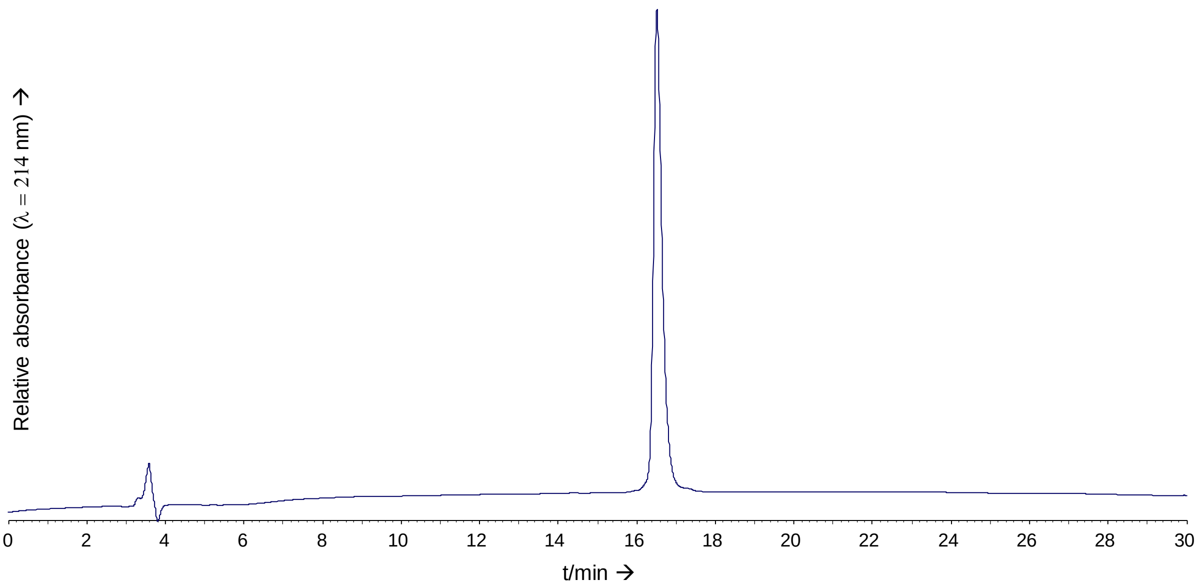

<u>Supplementary figure S12</u> - Analytical HPLC profile of purified peptide **3'**. Column: Nucleosil®; gradient: from 30% to 60% B over 30 min.

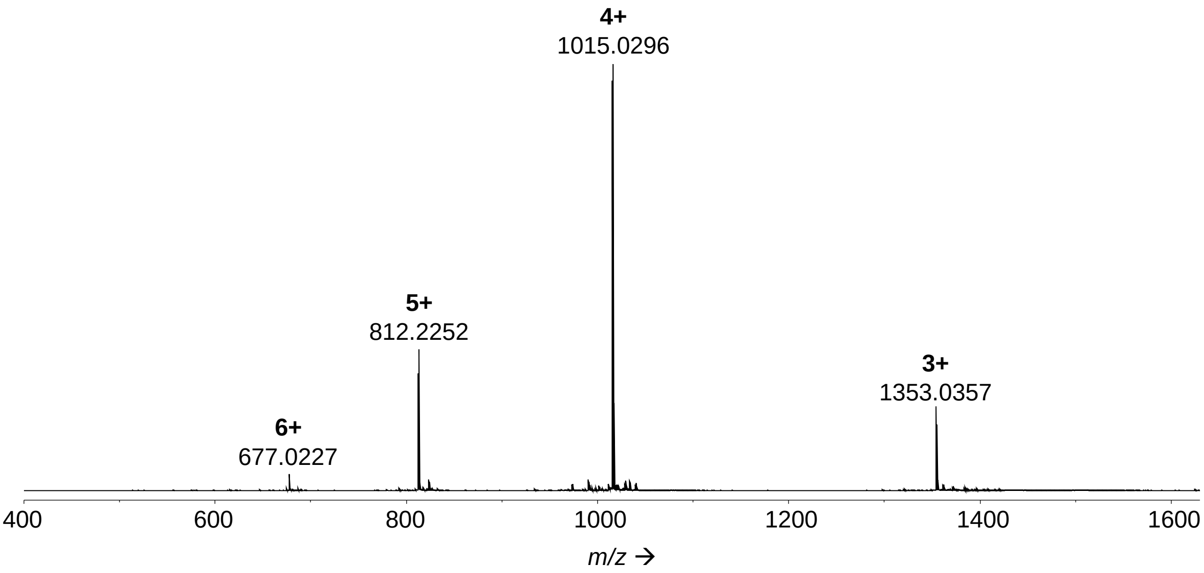

<u>Supplementary figure S13</u> - ESI-HRMS mass spectrum of peptide **3'**

D Studies of the Dtpp linker cleavage

D.1 First assay with hydrazine: co-products from the reduction of the alkyne

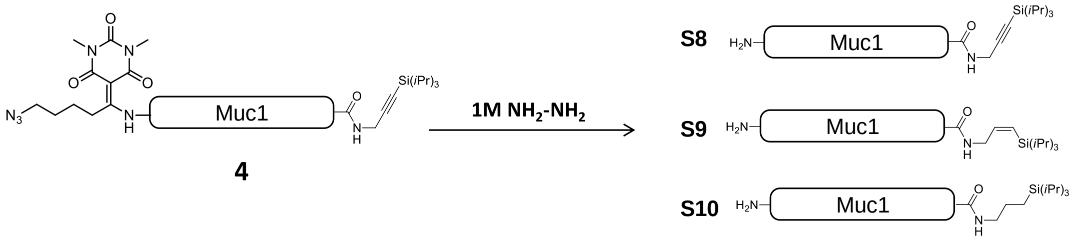

Supplementary scheme S12 – Reduction of protected alkyne by aqueous hydrazine solution

Peptide **4** was dissolved in a 1 M aqueous hydrazine solution (100 µM final peptide concentration, pH 9.5). Reaction was followed by analytical HPLC (column: Nucleosil®, gradient: from 25% to 55% B over 30 min). The amount of resulting products was measured by integration of the HPLC peaks at λ = 214 nm.

S8: ESI-HRMS (*m/z*): $[M+H]^+$ calcd for $C_{170}H_{273}N_{50}O_{53}Si$: 3890.9973, found: 3890.9951.
S9: ESI-HRMS (*m/z*): $[M+H]^+$ calcd for $C_{170}H_{275}N_{50}O_{53}Si$: 3893.0130, found: 3893.0131.
S10: ESI-HRMS (*m/z*): $[M+H]^+$ calcd for $C_{170}H_{277}N_{50}O_{53}Si$: 3895.0286, found: 3895.0285.

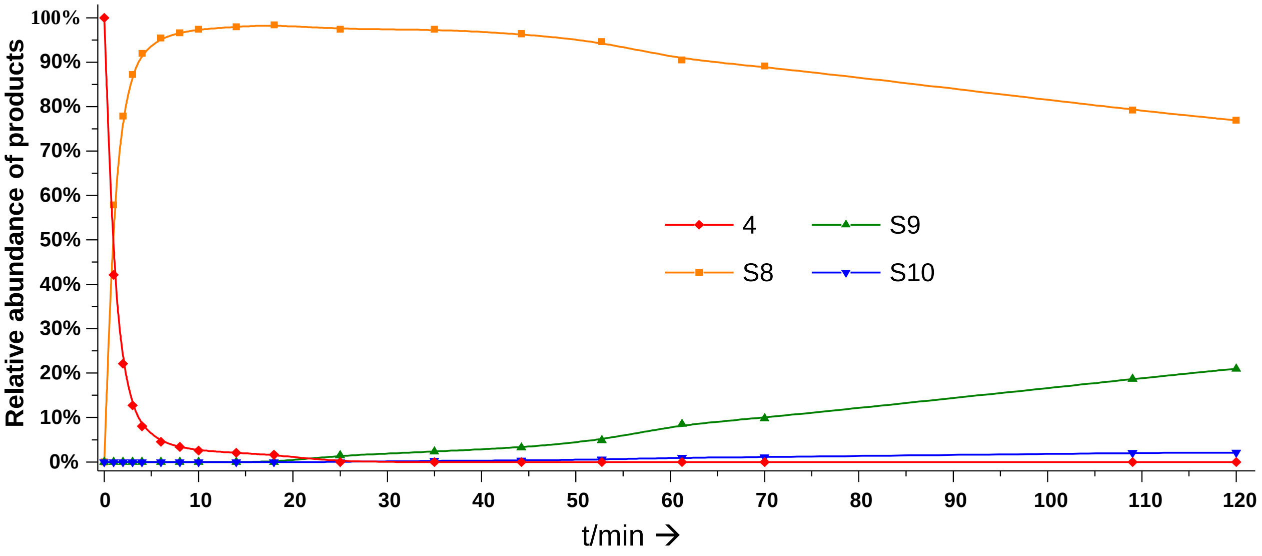

Supplementary figure S14 - Reaction of peptide **4** with a 1 M aqueous hydrazine solution

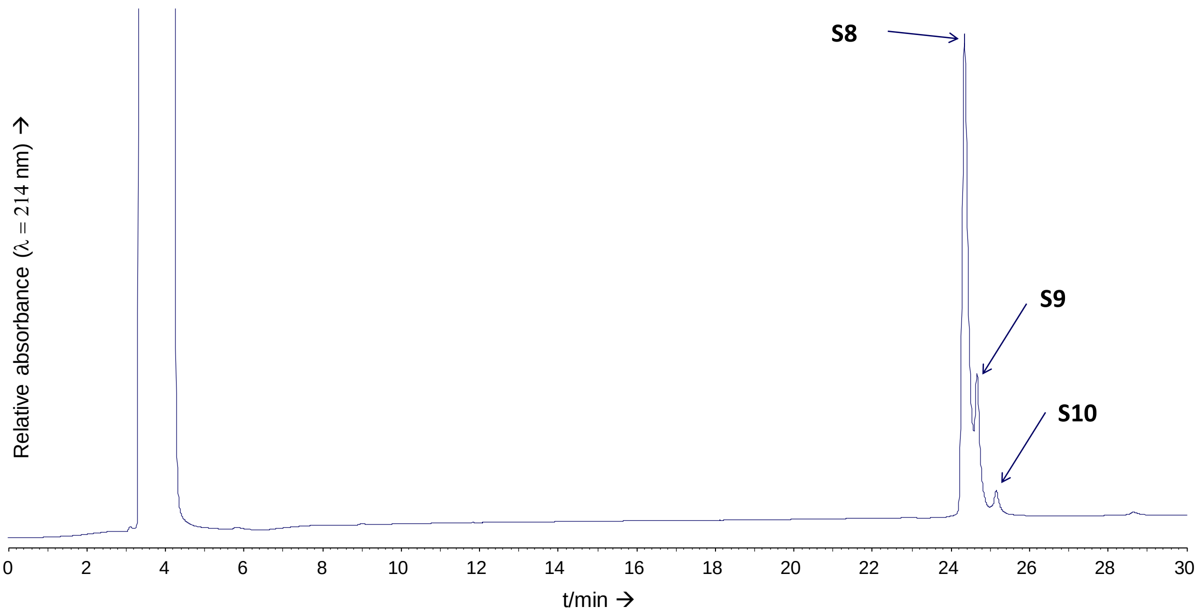

Supplementary figure S15 - Analytical HPLC profile of reaction mixture after 120 min incubation in 1 M aqueous hydrazine. Column: Nucleosil®; gradient: from 25% to 55% B over 30 min.

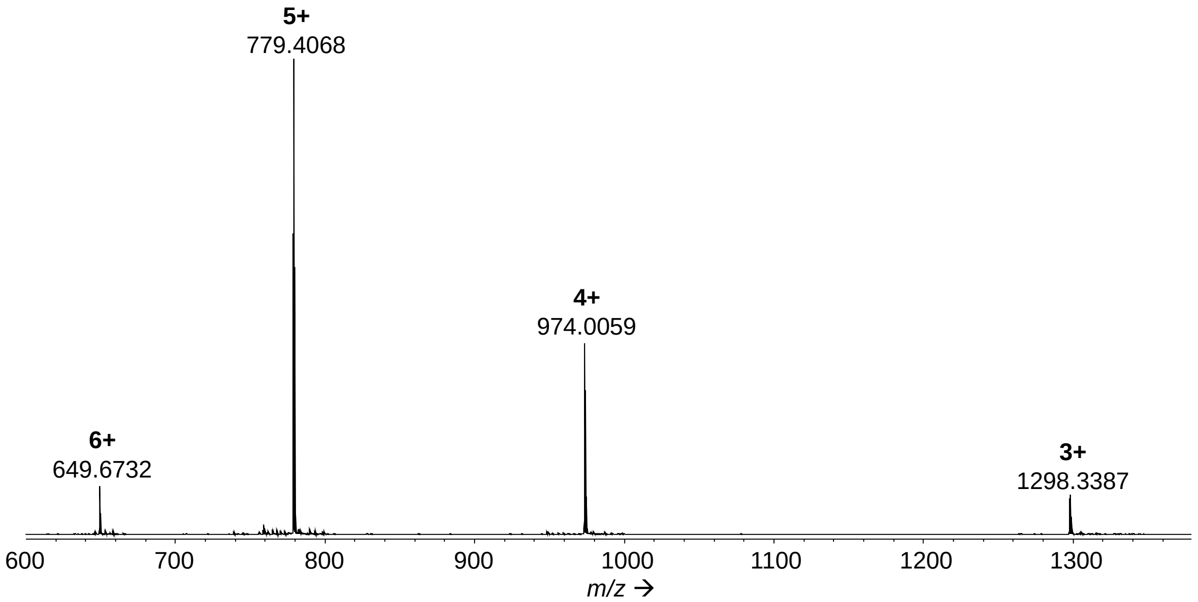

Supplementary figure S16 - ESI-HRMS mass spectrum of peptide **S8**

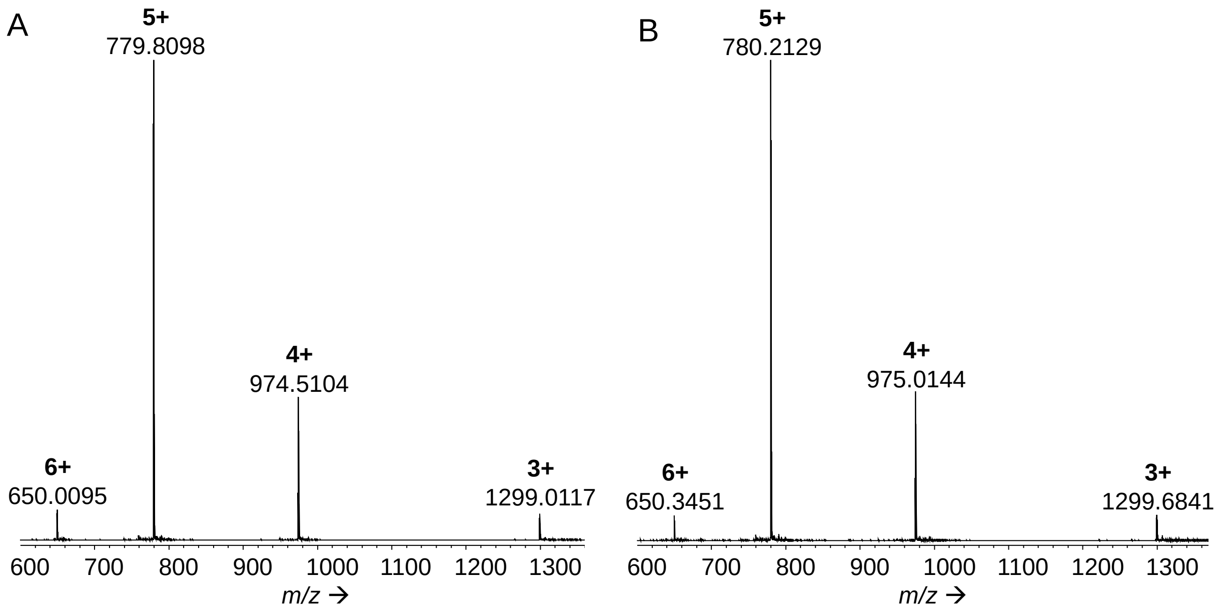

Supplementary figure S17 - A) ESI-HRMS mass spectrum of peptide **S9** B) ESI-HRMS mass spectrum of peptide **S10**

D.2 Case study of the reduction of a model TIPS-alkyne with aqueous hydrazine and hydroxylamine

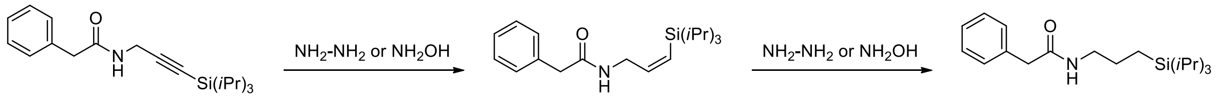

S11 **S12** **S13**

Supplementary scheme S13 - Reduction of a model TIPS-alkyne with aqueous hydrazine and hydroxylamine

2-Phenyl-*N*-[3-(triisopropylsilyl)prop-2-ynyl] acetamide **S11** (CAS N°: 1189342-28-6)[8] was subjected to a set of aqueous conditions to evaluate the kinetics of the reduction of the alkyne moiety by hydrazine and hydroxylamine. **S11** was directly dissolved in the different aqueous mixtures (1 mM final concentration) and stirred under an open air atmosphere at room temperature. The reaction was followed by analytical HPLC (column: Chromolith®; gradient: from 25% to 55% B over 5 min). The amount of the products **S12** and **S13** resulting from the reduction of **S11** was measured by integration of the HPLC peaks at λ = 254 nm.

Addition of sodium ascorbate prevented the reduction of TIPS-alkyne. We hypothesize that AscNa helps avoiding the air oxidation of hydrazine into diimide.

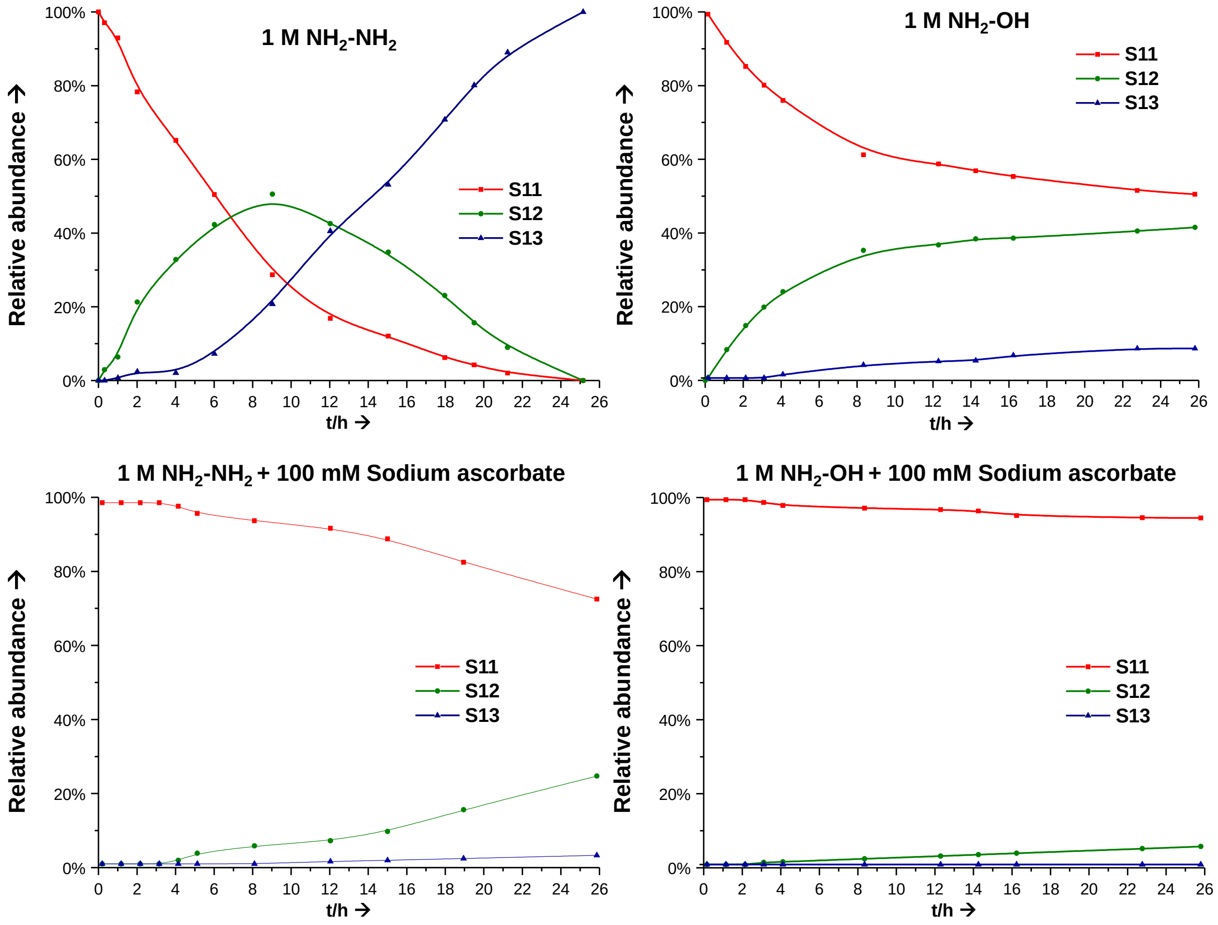

Supplementary figure S18 - Reduction of **S11** under a set of aqueous conditions

[8] I. E. Valverde, A. F. Delmas, V. Aucagne, *Tetrahedron* **2009**, *65*, 7597–7602.

2-Phenyl-*N*-[3-(triisopropylsilyl)prop-2-ynyl] acetamide **S11** (33 mg, 100 µmol) was dissolved in 1 M hydrazine aqueous solution (100 mL). The solution was stirred under an open air atmosphere at room temperature. After 6 h and 26 h of reaction time, 20 mL of the reaction mixture was acidified with fuming HCl (37%) and extracted with CH_2Cl_2 (4 x 10 mL). The combined organic layers were dried over $MgSO_4$, filtrated, concentrated under reduced pressure and analyzed with NMR.

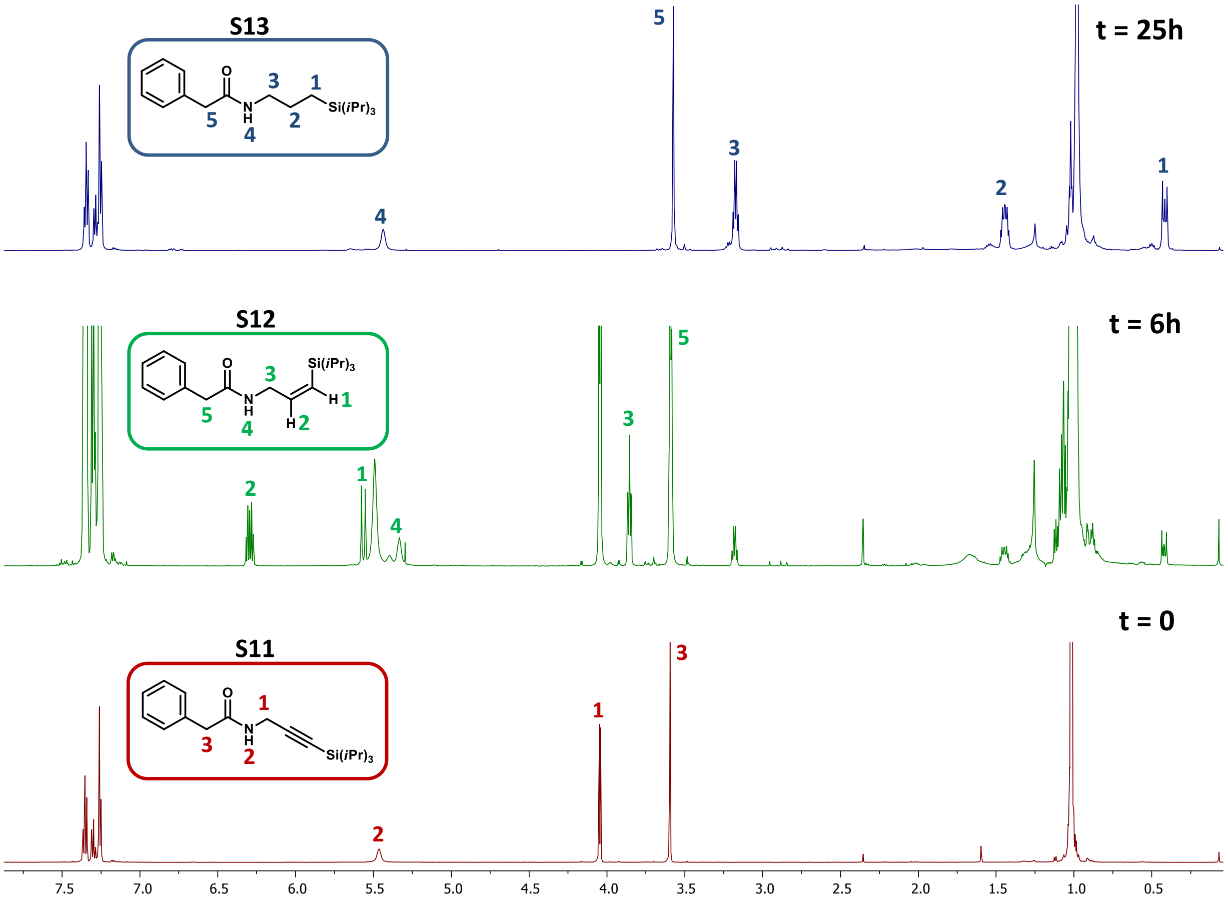

<u>**Supplementary figure S19**</u> - Copy of the ^{1}H NMR spectrum of pure **S11** and **S13**, and **S12** in mixture

<u>Selected data for S12 (not isolated from the mixture):</u>

^{1}H NMR (600 MHz, $CDCl_3$) δ = 6.22 (dt, *J* = 14.2, 7.0 Hz, 1H, H2), 5.49 (dt, *J* = 14.4, 1.4 Hz, 1H,H1), 5.26 (bm, 1H, H4), 3.79 (ddd, *J* = 7.0, 5.4, 1.4 Hz, 2H, H3).

ESI-HRMS (*m/z*): $[M+H]^+$ calcd for $C_{20}H_{34}NOSi$: 332.2404, found: 332.2406.

<u>S13:</u>

^{1}H NMR (600 MHz, $CDCl_3$) δ = 7.40 – 7.31 (m, 2H), 7.31 – 7.21 (m, 3H), 5.51 – 5.34 (m, 1H), 3.57 (s, 2H), 3.17 (dt, *J* = 6.5 Hz, 2H), 1.49 – 1.39 (m, 2H), 0.98 (s, 21H), 0.47 – 0.37 (m, 2H).

^{13}C NMR (151 MHz, $CDCl_3$) δ = 170.9, 135.1, 129.5, 129.1, 127.4, 44.0, 43.0, 24.4, 18.8, 10.8, 6.2.

ESI-HRMS (*m/z*): $[M+H]^+$ calcd for $C_{20}H_{36}NOSi$: 334.2560, found: 334.2563.

D.3 Case study of the reduction of a model terminal alkyne with aqueous hydrazine and hydroxylamine

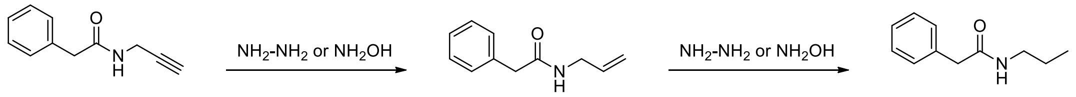

S14 **S15** **S16**

Supplementary scheme S14 -Reduction of a model terminal alkyne with aqueous hydrazine and hydroxylamine

N-Propargyl-2-phenylacetamide **S14** (CAS N°: 174271-37-5) was subjected to a set of aqueous conditions to evaluate the kinetics of the reduction of the alkyne moiety by hydrazine and hydroxylamine. **S14** was directly dissolved in the different aqueous mixtures (1 mM final concentration) and stirred under an open air atmosphere at room temperature. The reaction was followed by analytical HPLC (column: Chromolith®; gradient: from 20% to 50% B over 5 min). The amount of the products **S15** and **S16** resulting from the reduction of **S14** was measured by integration of the HPLC peaks at $\lambda = 254$ nm.

S15: CAS Registry Number: 30160-48-6

S16: CAS Registry Number: 64075-36-1

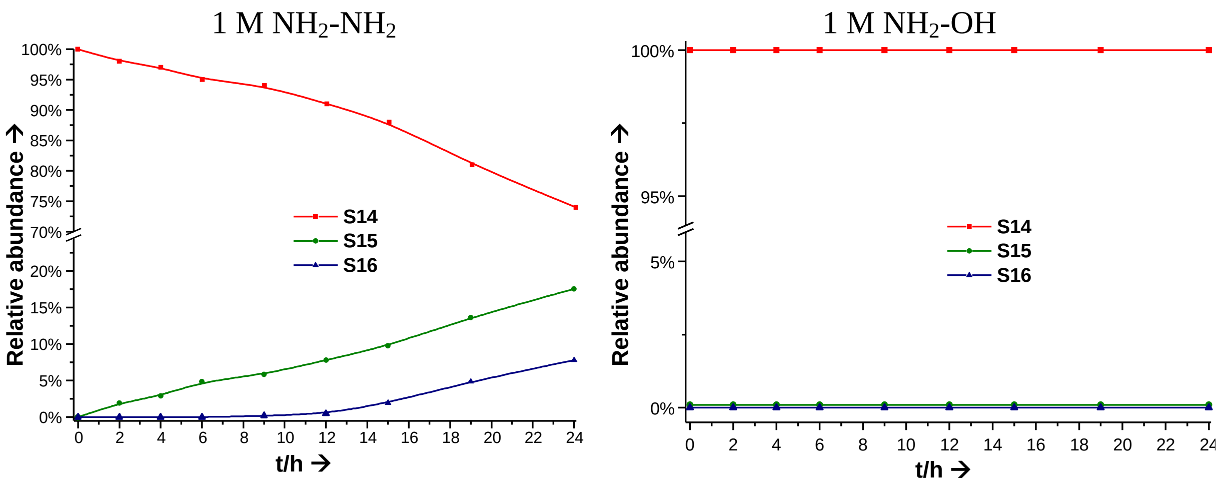

Supplementary figure S20 - Reduction of **S15** under a set of aqueous conditions

N-Propargyl-2-phenylacetamide **S15** (17 mg, 100 µmol) was dissolved in 1 M hydrazine aqueous solution (100 mL). The solution was stirred for 48 h under an open air atmosphere at room temperature. The reaction mixture was acidified with fuming HCl (37%) and extracted with CH_2Cl_2 (4 x 10 mL). The combined organic layers were dried over $MgSO_4$, filtrated, concentrated under reduced pressure and analyzed by NMR.

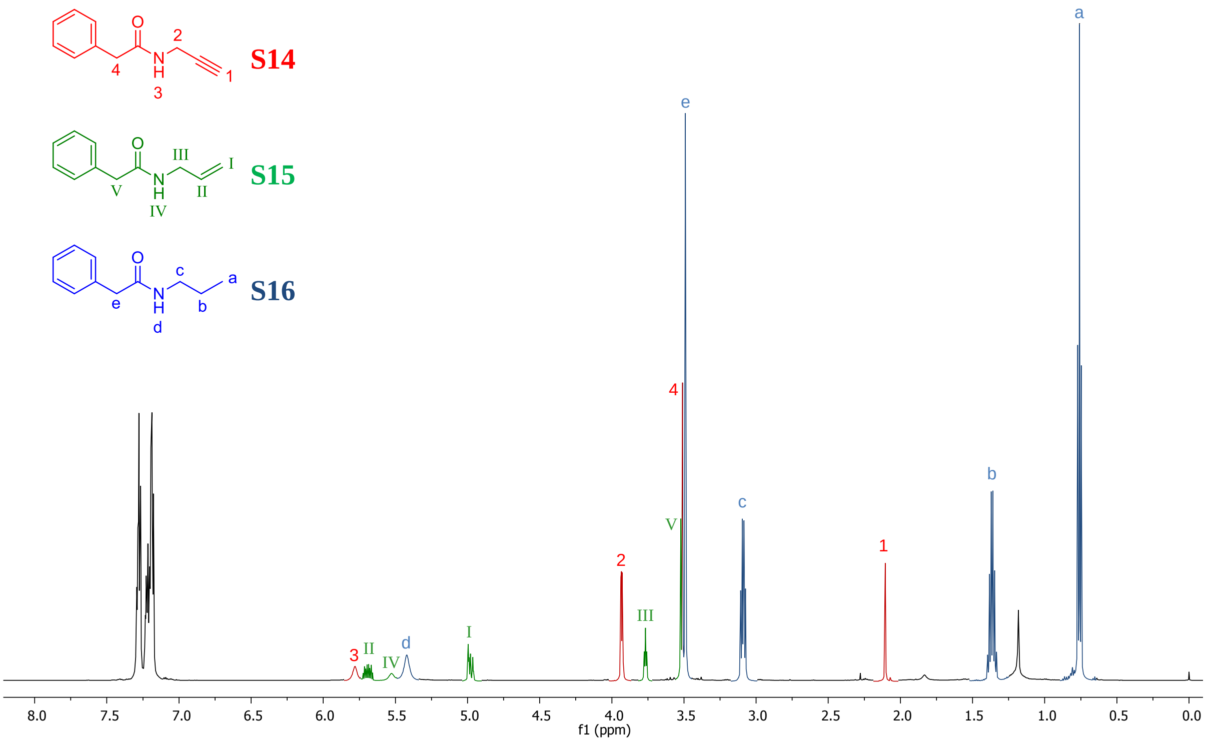

Supplementary figure S21 - Copy of the ^{1}H NMR spectrum (600 MHz, $CDCl_3$) of **S14** and **S15** and **S16** in mixture

Selected data for **S14** (not isolated from the mixture):
^{1}H NMR (600 MHz, $CDCl_3$) δ = 3.93 (dd, *J* = 5.3, 2.6 Hz, 2H), 3.51 (s, 2H), 2.11 (t, *J* = 2.6 Hz, 1H).

Selected data for **S15** (not isolated from the mixture):
^{1}H NMR (600 MHz, $CDCl_3$) δ = 5.69 (ddt, *J* = 17.1, 10.7, 5.5 Hz, 1H), 5.53 (br, 1H), 5.03 – 4.93 (br, 2H), 3.77 (br, 2H), 3.52 (s, 2H).

Selected data for **S16** (not isolated from the mixture):
^{1}H NMR (600 MHz, $CDCl_3$) δ = 5.42 (br, 1H), 3.49 (s, 2H), 3.09 (dt, *J* = 7.4, 5.6 Hz, 2H), 1.36 (dt, *J* = 7.4, 7.4 Hz, 2H), 0.76 (t, *J* = 7.4 Hz, 3H).

D.4 Kinetic studies of the Dtpp linker cleavage under optimized conditions

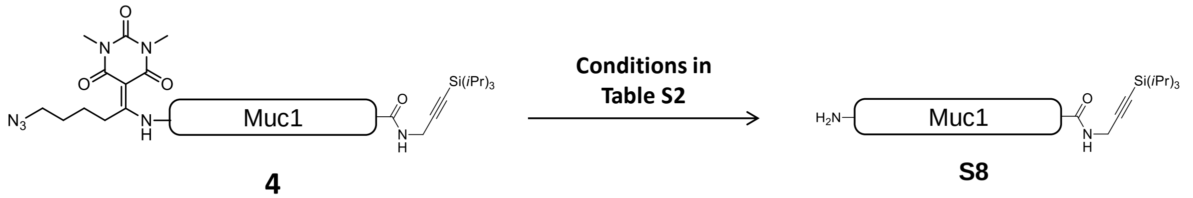

Supplementary scheme S15 - Cleavage of the Dtpp linker of peptide **4**

Peptide **4** was subjected to a set of conditions to evaluate the liability of the Dtpp linker in solution (1 mM final peptide concentration). The cleavage of the linker was followed by analytical HPLC (column: Nucleosil®, gradient: from 25% to 55% B over 30 min). The amount of free amine **S13** resulting from the cleavage of the linker was measured by integration of the HPLC peaks at λ = 214 nm.

	Conditions	pH	$t_{1/2}$
A	1 M NH_2-NH_2 + 100 mM AscNa	9.5	~ 1 min
B	1 M NH_2-NH_2 + 100 mM AscNa + 100 mM HEPES	7.5	~ 3 min
C	1 M NH_2-OH + 100 mM AscNa	8.5	~ 7 min
D	1 M NH_2-OH + 100 mM AscNa + 100 mM HEPES	7.5	~ 18 min
E	1 M NH_2-OH + 100 mM AscNa + 100 mM HEPES	7.0	~ 82 min

Supplementary table S3 – Approximate half-times for the complete cleavage of Dtpp linker by a set of aqueous conditions

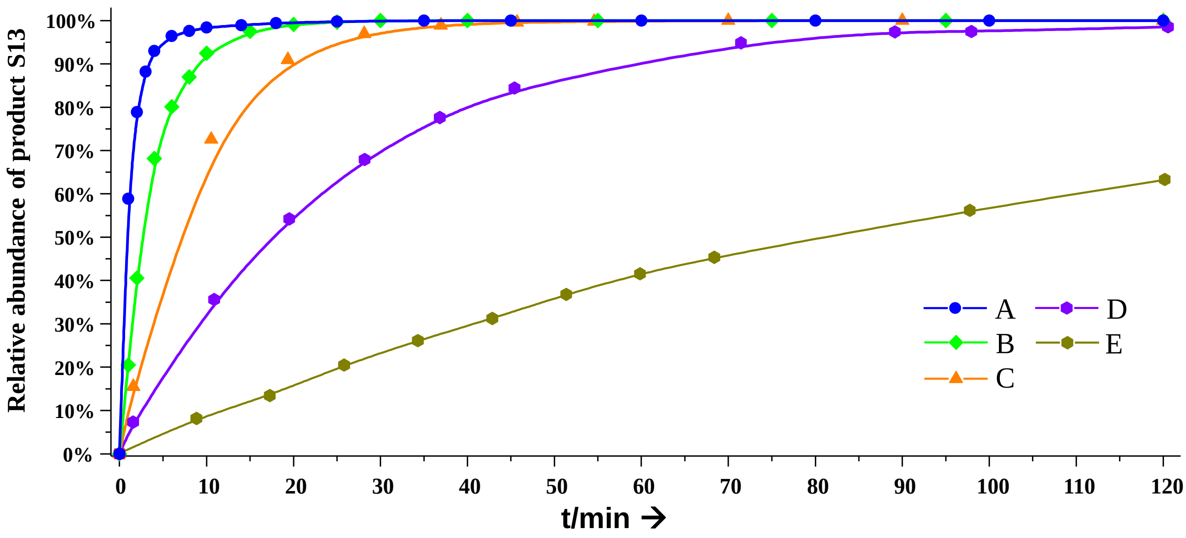

Supplementary figure S22 – Kinetics of the Dtpp linker cleavage in solution: treatment of **4** by a set of conditions

E Synthesis of 8 by successive deprotection of 5

E.1 Synthesis of protected glycopeptide 5

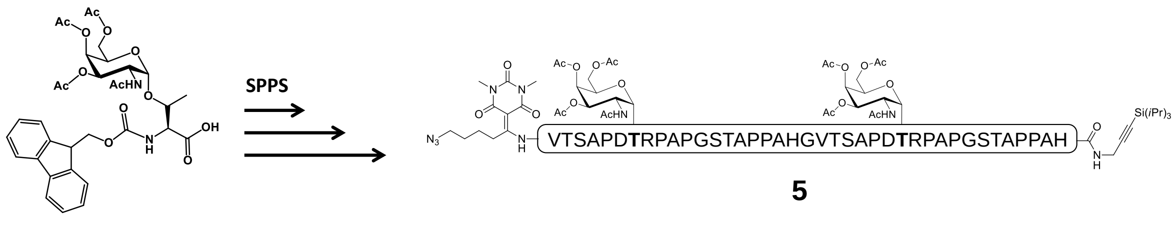

Supplementary scheme S16 - Solid supported synthesis of protected glycopeptide **5**

The backbone amide linker 4-(4-formyl-3,5-dimethoxyphenoxy)butyric acid, (67 mg, 0.25 mmol, 2.5 equiv.) was loaded on Rink amide ChemMatrix® resin (192 mg, 0.52 mmol/g, 0.1 mmol) using HATU (95 mg, 0.25 mmol, 2.5 equiv.) and *i*Pr_2NEt (87 µL, 0.5 mmol, 5 equiv.) in DMF for 2 h at room temperature. The resin was then thoroughly washed with DMF. In case of incomplete reaction (positive Kaiser's test), this protocol was repeated once.
3-(triisopropylsilyl)prop-2-yn-1-amine (106 mg, 0.5 mmol, 5 equiv.) and $NaBH_3CN$ (32 mg, 0.5 mmol, 5 equiv.) dissolved in 5 mL of DMF/MeOH/AcOH (7:2:1) were added to the resin (1 equiv., swelled in DMF prior to the reaction), and the resulting suspension was mixed overnight at 50 °C with N_2 bubbling.
The resin was then thoroughly washed with DMF and CH_2Cl_2. Fmoc-His(Trt)-OH (620 mg, 1 mmol, 10 equiv.) was coupled onto the resulting resin with HATU (380 mg, 1 mmol, 10 equiv.) and *i*Pr_2NEt (348 µL, 2 mmol, 20 equiv.) in DMF overnight at room temperature.
The elongation of the peptide was performed by standard automated solid phase synthesis (p S4) up to ^{2}Thr. Prolines 4, 5, 24 and 25 were coupled twice following the automated program for double couplings. Glycosylated amino acid were introduced at position 7 and 27 using Fmoc-Thr(Ac_3-α-D-GalNAc)-OH (135 mg, 0.2 mmol, 2 equiv.) with HATU (76 mg, 0.2 mmol, 2 equiv.) HOAt (27 mg, 0.2 mmol, 2 equiv.) and 2,4,6-trimethylpyridine (27 µL, 2 mmol, 2 equiv.) in DMF for 2 h at room temperature.[9] A 65% elongation yield was obtained, determined by the titration of the first and last (^{2}Thr) Fmoc group deprotection (UV, 301 nm).
Peptide resin (25 µmol, 1 equiv., swelled in DMF prior to the reaction) was further washed with CH_2Cl_2, and treated with TFA/*i*Pr_3SiH/1,3 dimethoxybenzene (90:5:5) for 2 h. Peptide **5** was precipitated by dilution with ice-cold diethyl ether, recovered by centrifugation, washed 3 times with diethyl ether then dried under reduced pressure. The crude mixture was purified by semi-preparative HPLC and lyophilized to yield **5** as a white fluffy powder (30 mg, 5.7 µmol, 23%* isolated yield based on the original resin loading).

*: estimated considering a MW = 5267 g/mol taking into account 4 trifluoroacetate counter-anions, as predicted from the sequence: 2Arg, 2His = +4 overall charge. Water content is not taken into account.

ESI-HRMS (*m/z*): $[M+H]^+$ calcd for $C_{209}H_{324}N_{57}O_{72}Si$: 4812.3213, found: 4812.3197.

[9] Ludek, K. Adams, J. J. Barchi, J. C. Gildersleeve, *J. Am. Chem. Soc.* **2012**, *134*, 6316–6325

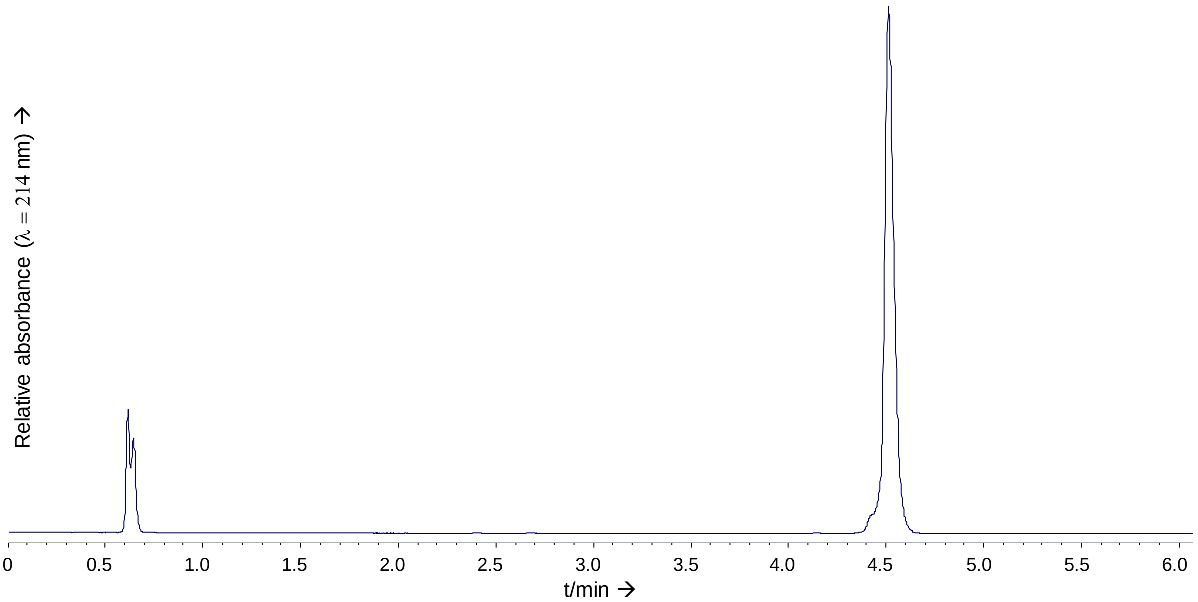

Supplementary figure S23 - Analytical HPLC profile of purified peptide **5**. Column: Chromolith®; gradient: from 5% to 65% B over 5 min

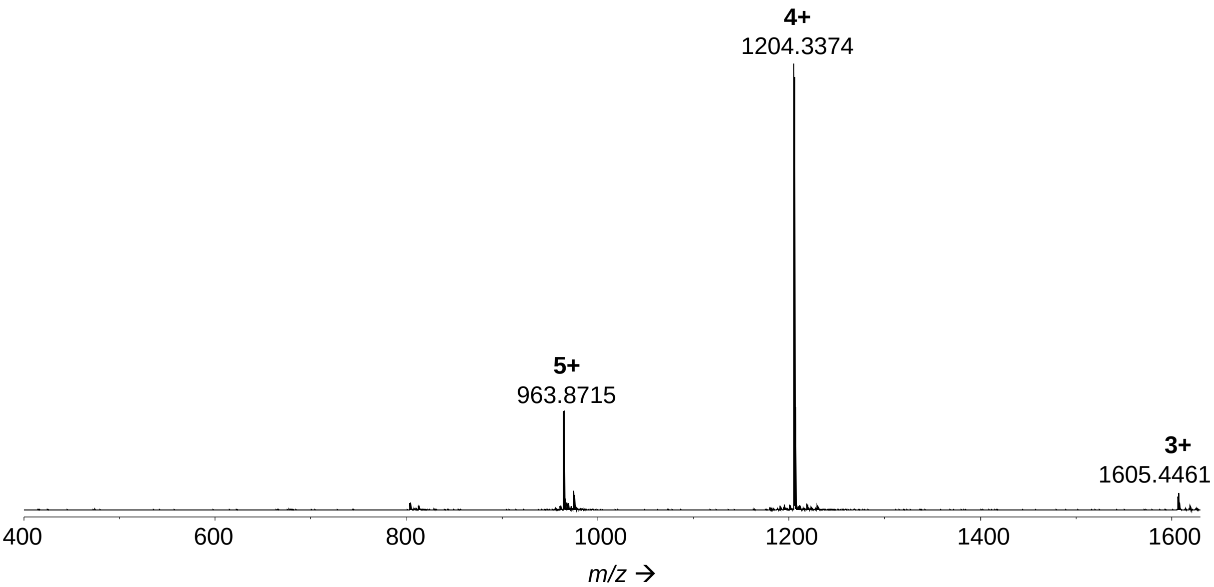

Supplementary figure S24 - ESI-HRMS mass spectrum of peptide **5**

E.2 Selective deprotection of the *O*-acetyl group of 5

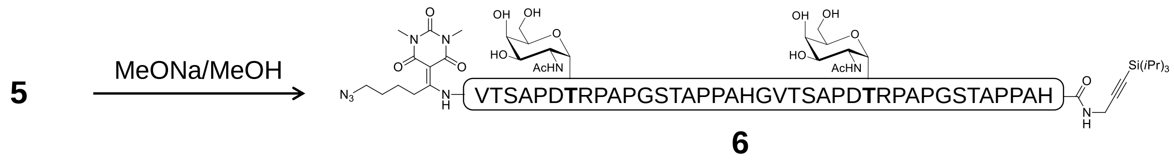

Supplementary scheme S17 - Selective deprotection of the O-acetyl group of **5**

Purified glycopeptide **5** (2 mmol) was dissolved in anhydrous methanol (2 mL) under an argon atmosphere. Freshly prepared 1% MeONa/MeOH solution was added dropwise at 0 °C until pH 10 was reached when spotting an aliquot of the solution on a wet pH indicator paper. After 1 h stirring at room temperature and confirmation of the completion of the reaction by RP-HPLC analysis, the reaction mixture was neutralized with acetic acid (10 µL). Volatiles were removed under reduced pressure and the residue was dissolved in mQ water (1 mL). Lyophilization yielded glycopeptide **6** as a white fluffy powder.

ESI-HRMS (*m/z*): $[M+H]^+$ calcd for $C_{197}H_{312}N_{57}O_{66}Si$: 4560.2579, found: 4560.2563.

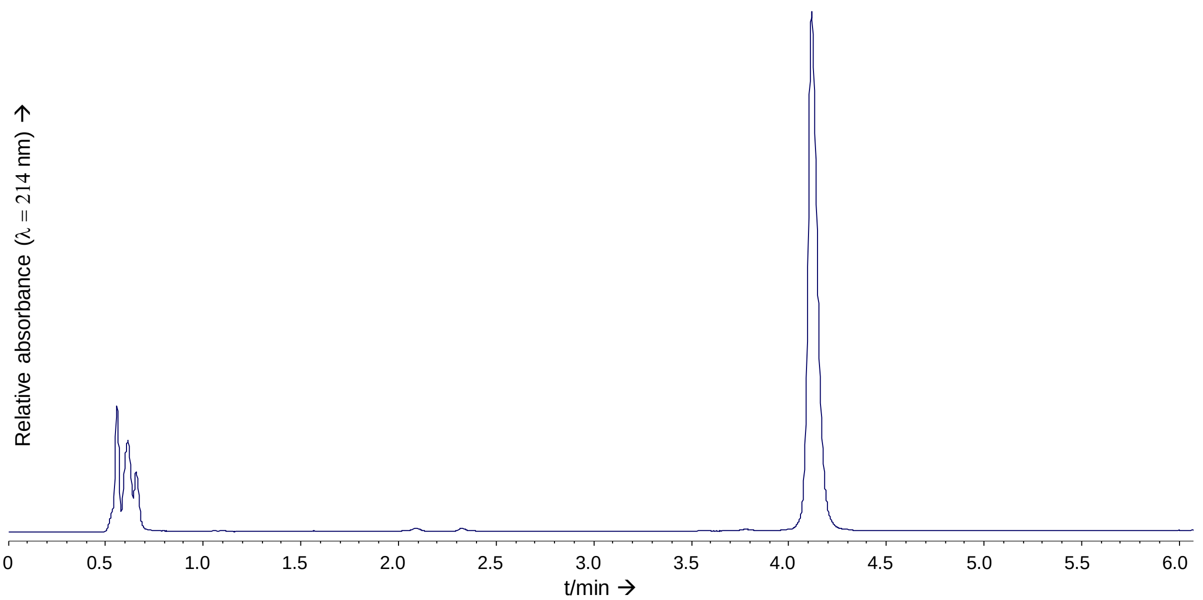

<u>Supplementary figure S25</u> - Analytical HPLC profile of crude peptide **6.** Column: Chromolith®; gradient: from 5% to 65% B over 5 min

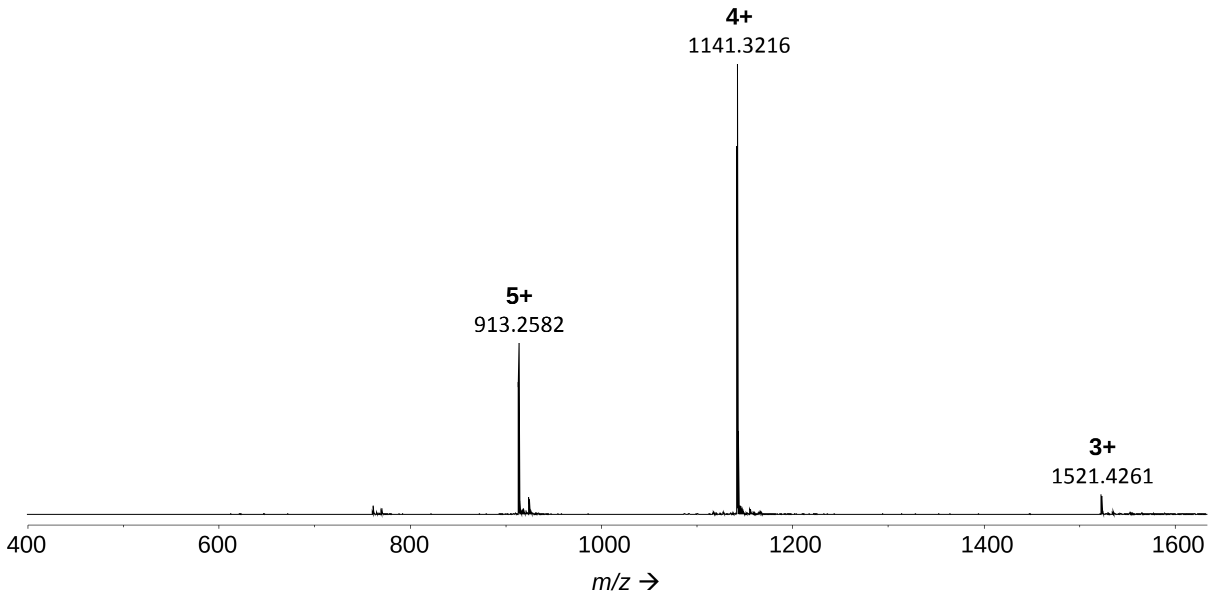

<u>Supplementary figure S26</u> - ESI-HRMS mass spectrum of peptide **6**

E.3 Selective deprotection of the TIPS group of 6

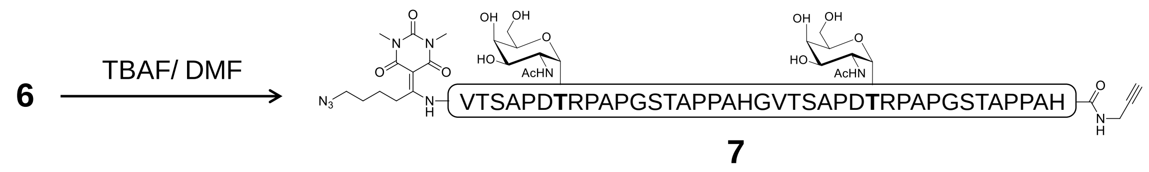

Supplementary scheme S18 - Selective deprotection of the TIPS group of **6**

Crude lyophilized glycopeptide **6** (100 nmol) was dissolved in anhydrous DMF (50 µL) under an argon atmosphere. 50 µL of a 200 mM tetrabutylammonium fluoride trihydrate in anhydrous DMF was added to the mixture. After 1 h stirring at room temperature and confirmation of the completion of the reaction by RP-HPLC, the reaction mixture was neutralized with acetic acid (10 µL) and diluted with water (2 mL). DMF and salts were removed using standard hydrophobic SPE cartridge. Lyophilization yielded glycopeptide **7** as a white fluffy powder.

ESI-HRMS (*m/z*): $[M+H]^+$ calcd for $C_{188}H_{292}N_{57}O_{66}$: 4404.1245, found: 4403.1363.

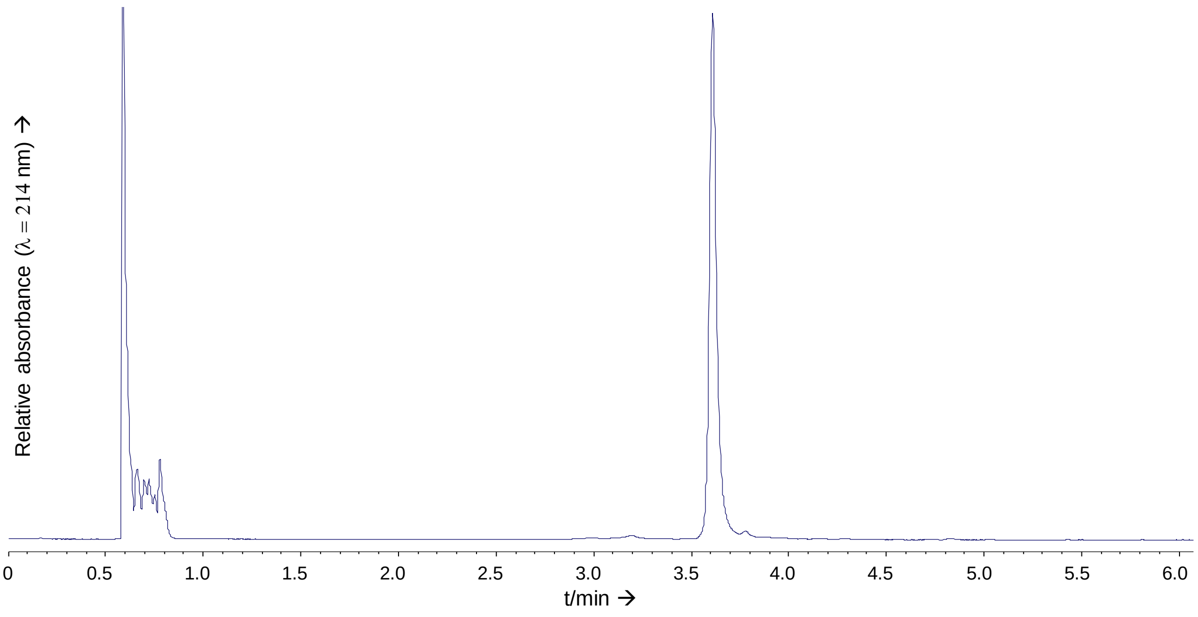

Supplementary figure S27 - Analytical HPLC profile of crude peptide **7**. Column: Chromolith®; gradient: from 5% to 65% B over 5 min.

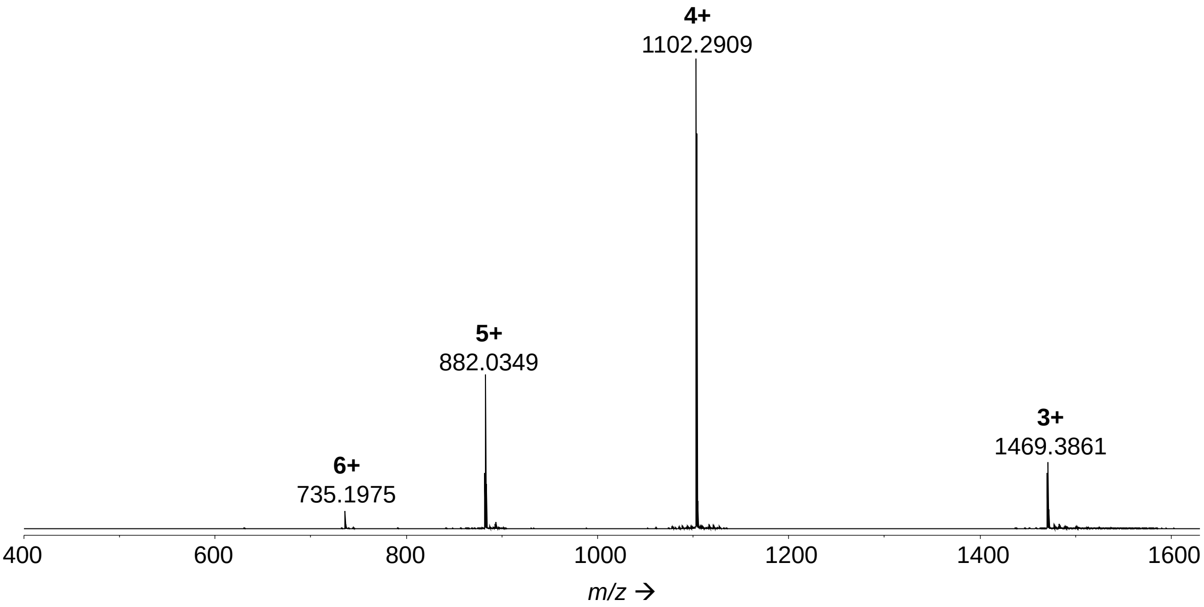

Supplementary figure S28 - ESI-HRMS mass spectrum of peptide **7**

E.4 Selective cleavage of the Dtpp linker

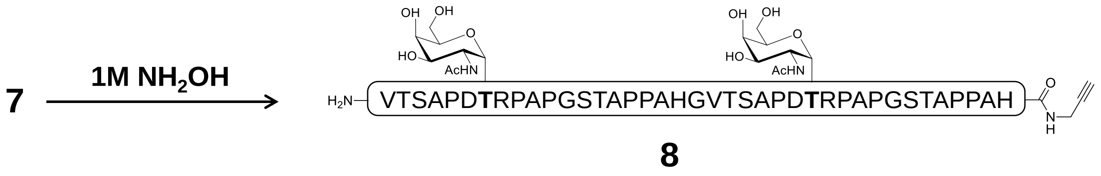

Supplementary scheme S19 - Selective cleavage of the Dtpp linker of **7**

Crude lyophilized glycopeptide **7** (100 nmol) was dissolved in water (50 µL) and a 2M hydroxylamine solution (50 µL) was added to the mixture. After 2 h stirring at room temperature and confirmation of the completion of the reaction by RP-HPLC, the reaction mixture was neutralized with acetic acid (20 µL) and diluted with water (2 mL). Salts and hydroxylamine were removed using standard hydrophobic SPE cartridge. Lyophilization yielded glycopeptide **8** as a white fluffy powder.

ESI-HRMS (*m/z*): $[M+H]^+$ calcd for $C_{177}H_{279}N_{52}O_{63}$: 4141.0227, found: 4141.0252.

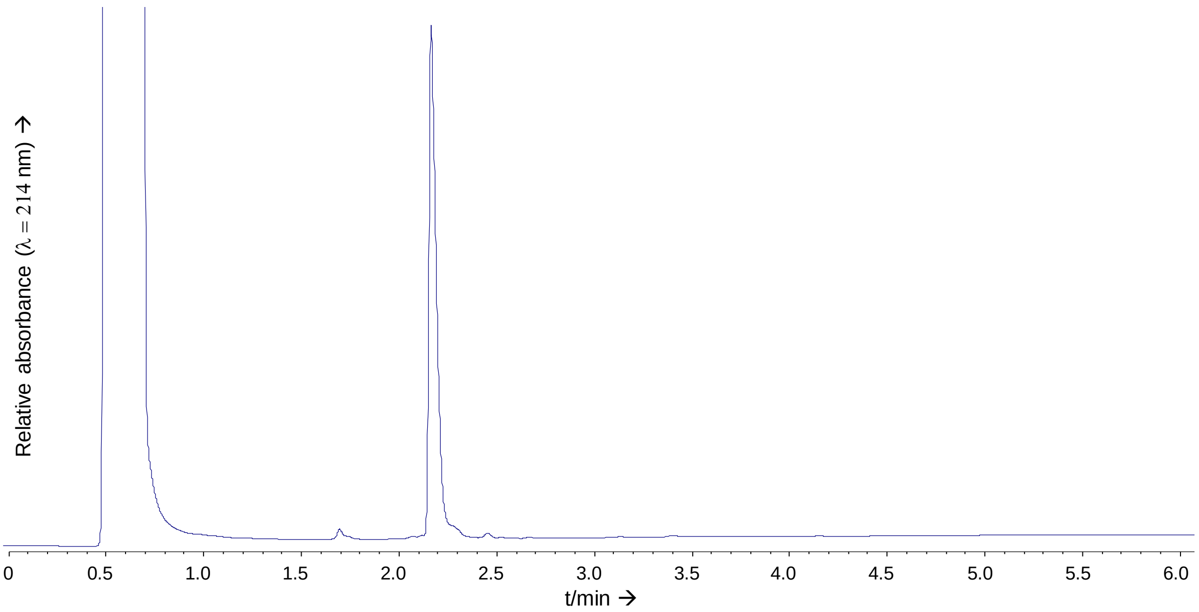

Supplementary figure S29 - Analytical HPLC profile of crude glycopeptide **8**. Column: Chromolith®; gradient: from 5% to 65% B over 5 min.

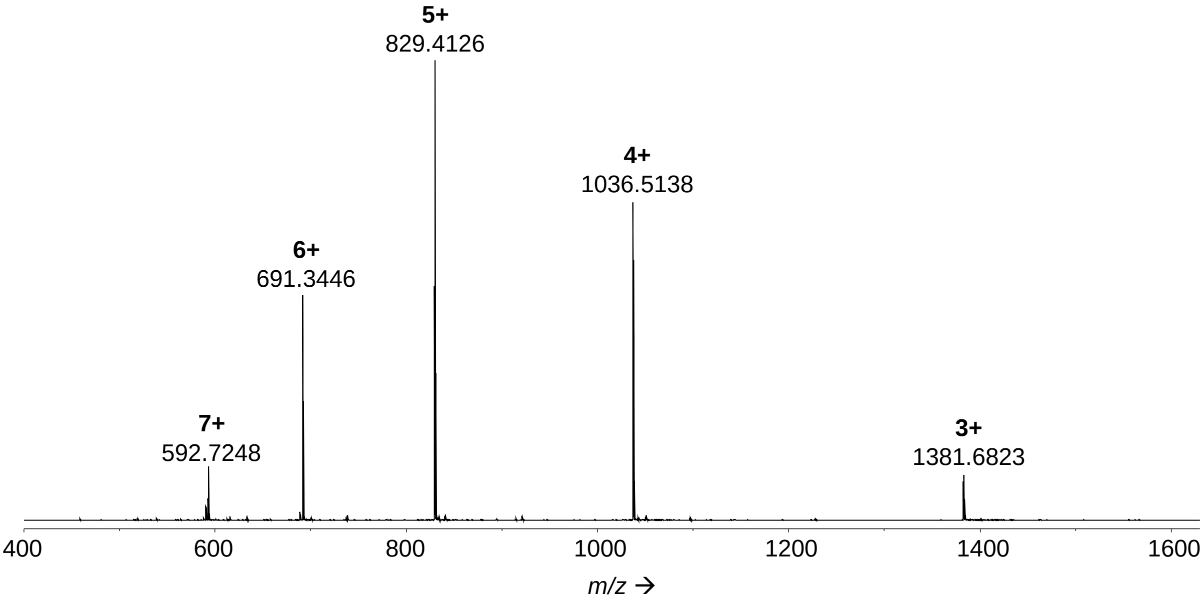

Supplementary figure S30 - ESI-HRMS mass spectrum of glycopeptide **8**

E.5 Stability of glycopeptide 8

- **Stability at pH11.7**

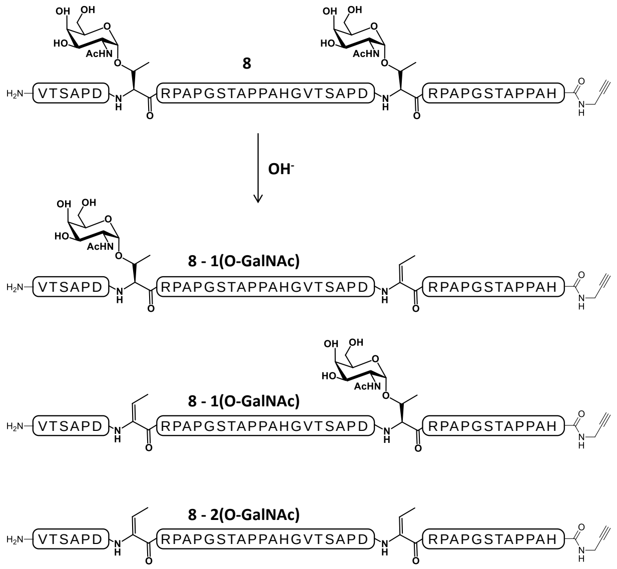

Supplementary scheme S20 – sugar β-elimination under aqueous alkaline condition

Purified glycopeptide **8** (100 nmol) was dissolved in water (50 µL) and 50 µL CAPS buffer (200 mM, pH11.7) was added. After 16 h stirring at room temperature, the reaction mixture was analyzed by LC/HRMS.

Glycopeptide **8 - 1(*O*-GalNAc):**
ESI-HRMS (*m/z*): $[M+H]^+$ calcd for $C_{169}H_{264}N_{51}O_{57}$: 3919.9327, found: 3919.9384.

Glycopeptide **8 - 2(*O*-GalNAc):**
ESI-HRMS (*m/z*): $[M+H]^+$ calcd for $C_{161}H_{249}N_{50}O_{51}$: 3698.8428, found: 3698.8492.

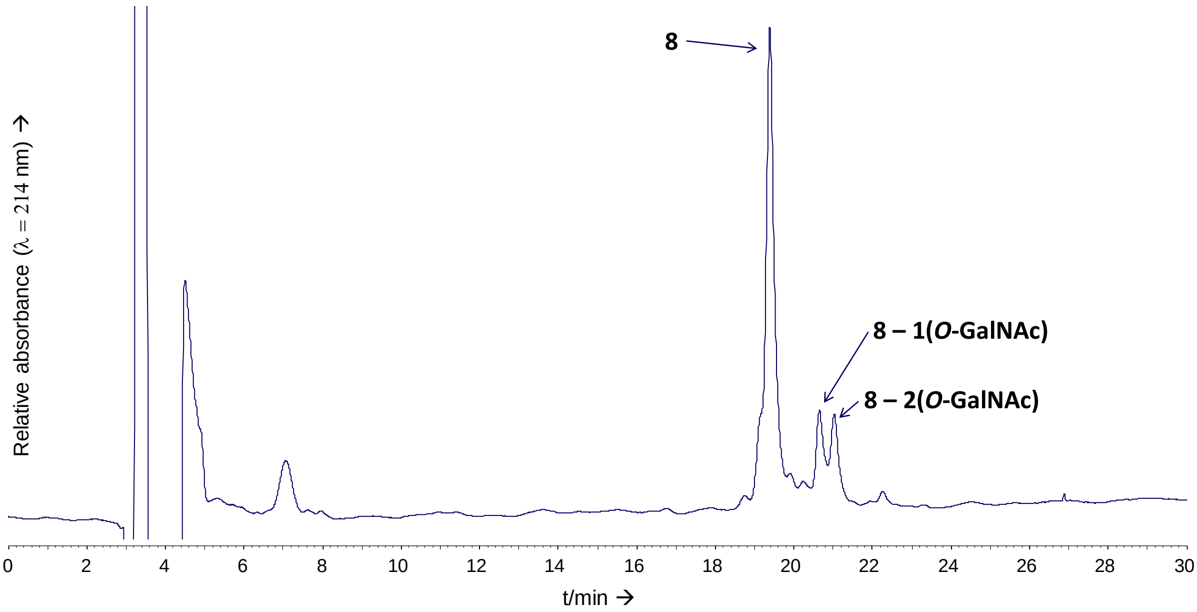

Supplementary figure S31 - Analytical HPLC profile of the reaction mixture after treatment of glycopeptide **8** with 50 mM CAPS buffer pH 11.7 for 16 h at room temperature. Column: Nucleosil®; gradient: from 10% to 25% B over 30 min.

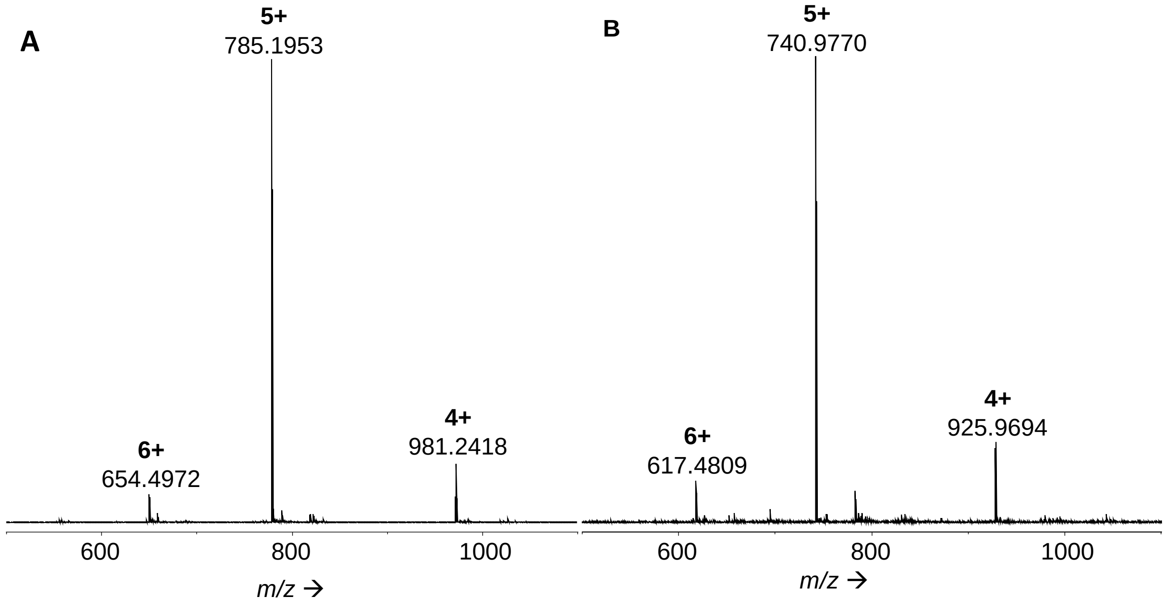

Supplementary figure S32 - A) ESI-HRMS mass spectrum of glycopeptide **8 - 1(O-GalNAc)** B) ESI-HRMS mass spectrum of peptide **8 - 2(O-GalNAc)**

- **Stability in 1 M aqueous NH_2OH**

Purified glycopeptide **8** (100 nmol) was dissolved in water (50 µL) and 50 µL of a 2M aqueous hydroxylamine solution was added. After 24 h stirring at room temperature, the reaction mixture was analyzed by LC/HRMS.

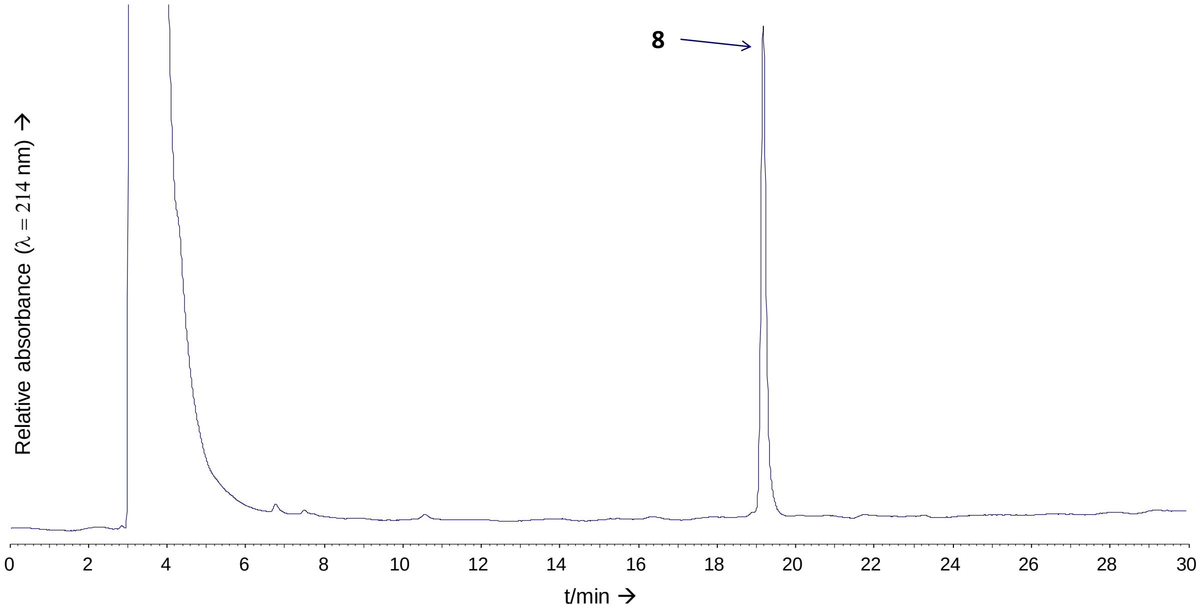

Supplementary figure S33 - Analytical HPLC profile of the reaction mixture after treatment of glycopeptide **8** with 1 M aqueous hydroxylamine solution for 24 h at room temperature. Column: Nucleosil®; gradient: from 10% to 25% B over 30 min.

F Studies for improvement of solid supported enzymatic glycosylation

F.1 Preparation of the alkyne-functionalized solid support

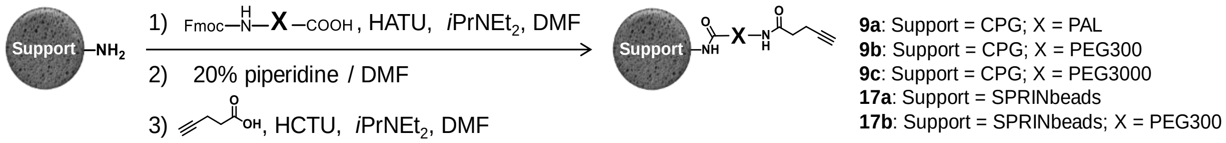

Supplementary scheme S21 - Preparation of the alkyne-functionalized capture resins **9a-c** and **17a-b**

Controlled Pore Glass (aminopropyl CPG beads TRISOPERL®) or polymethacrylate resin (SPRINBeads AH130)[10] was introduced in a syringe equipped with a polypropylene frit and a teflon stopcock and washed successively with DMF (3x), 10% *i*Pr_2NEt in CH_2Cl_2 (3x), DMF (3x) and peptide synthesis-grade DMF (3x). Then, Fmoc-spacer-OH (2 equiv., see Table S3 Fmoc-NH-X-COOH,) and HATU (2 equiv.) dissolved in a minimum amount of DMF, were transferred by suction followed by *i*Pr_2NEt (4 equiv.). The resin suspension was mixed by rotation for 16 h. In case of incomplete reaction (positive Kaiser's test), this protocol was repeated once followed by capping with acetic anhydride. The Fmoc group was removed by a treatment by 20% piperidine in DMF (3 x 3min), followed by thorough wash with DMF. Finally, pentynoic acid was coupled using HCTU (10 equiv.) and *i*Pr_2NEt (20 equiv.) in DMF to give the respective alkyne-containing solid support.

	Support	Spacer (Fmoc-NH-X-COOH)	Final Loading
**9a**	CPG	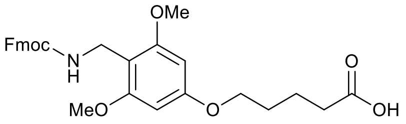 Fmoc-PAL-OH	145 µmol/g
**9b**	CPG	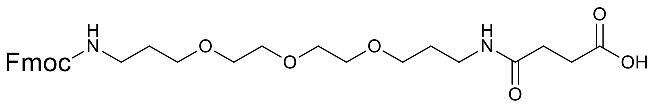 Fmoc-TTDS-OH (172089-14-4, ~ 300 Da)	120 µmol/g
**9c**	CPG	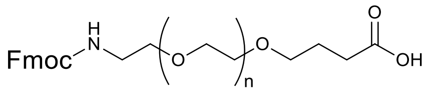 Fmoc-NH-PEG-COOH (3000 Da polydisperse)	20 µmol/g
**S17a**	SPRINbeads	No linker	60 µmol/g
**S17b**	SPRINbeads	Fmoc-TTDS-OH	60 µmol/g

Supplementary table S4 – Alkyne-functionalized solid support

[10] a) A. Basso, P. Braiuca, L. De Martin, C. Ebert, L. Gardossi, P. Linda, S. Verdelli, A. Tam, *Chem. Eur. J.* **2004**, *10*, 1007–1013. b) L. Sinigoi, P. Bravin, C. Ebert, N. D'Amelio, L. Vaccari, L. Ciccarelli, S. Cantone, A. Basso, L. Gardossi, *J. Comb. Chem.* **2009**, *11*, 835–845.

F.2 Typical procedure for the release of the peptide

The peptide was released by cleaving the Dtpp linker through treatment with 1 M NH_2-OH aqueous solution (1:10 *v*/*v* swollen resin/total volume), for 60 min at room temperature (addition of 100 mM AscNa is required for TIPS-protected alkynes).[11] This protocol was repeated once and the solid support was finally washed with de-ionized water. Released peptide was isolated from the mixture using standard procedures such as hydrophobic SPE cartridge.

[11] Note that contrary to TIPS-protected alkynes, terminal alkynes were much less sensitive to reduction during Dtpp cleavage, and did not require addition of ascorbate

F.3 Typical procedure for the grafting of peptide 4 on an alkyne-functionalized resin through CuAAC

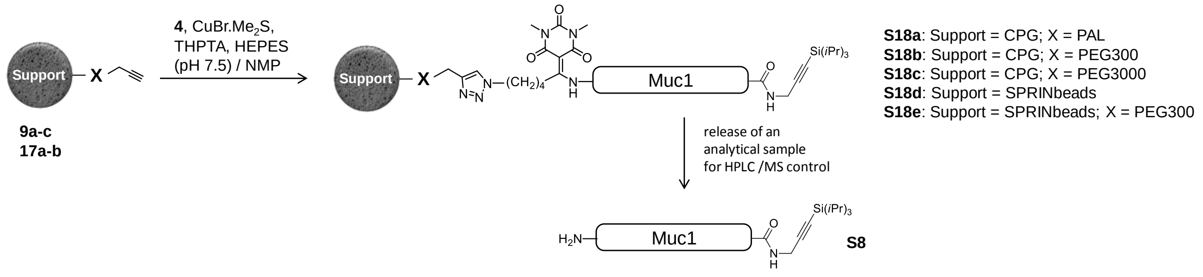

Supplementary scheme S22 - Grafting of peptide **4** on alkyne functionalized resin

A solution of the azide containing peptide (0.5 µmol) dissolved in 400 µL of a 1:1 mixture of NMP and 200 mM HEPES buffer pH 7.5 was added to a 2 mL microcentrifuge tube containing the alkyne functionalized solid support. To the suspension were subsequently added a 1 M aqueous aminoguanidine solution (15 µL, 30 equiv.), a 1 M aqueous *tert*-butanol solution (15 µL, 30 equiv.). The tube was sealed with a rubber septum and the resulting mixture was further deoxygenated through several successive vacuum (15 mbar) / argon cycles. Then, a mixture containing copper(I) bromide-dimethyl sulfide complex (30 equiv.) and THPTA (40 equiv.) dissolved under argon in 50 µL of deoxygenated NMP was added, the resulting suspension was further deoxygenated and was stirred for 5 h at 37 °C. The supernatant was analyzed by HPLC to check the total consumption of the azidopeptide. Capping of remaining solid-supported terminal alkyne was performed by adding 2-(2-azido-ethoxy)-ethanol (100 equiv.). The mixture was stirred an additional hour at 37 °C. The resin was then extensively washed with NMP, de-ionized water, then repeatedly treated (3x) with a pH 7 aqueous buffer containing 6 M guanidinium chloride, 0.1 M EDTA and 0.1 M sodium dihydrogenophosphate for a few minutes then drained and finally extensively washed with de-ionized water.

A few beads of the resulting solid supported peptide were cleaved using the general procedure (p S53) to give crude peptide **S8** which was analyzed by RP-HPLC and HRMS.

ESI-HRMS (*m/z*): $[M+H]^+$ calcd for $C_{170}H_{273}N_{50}O_{53}Si$: 3890.9973, found: 3890.9997.

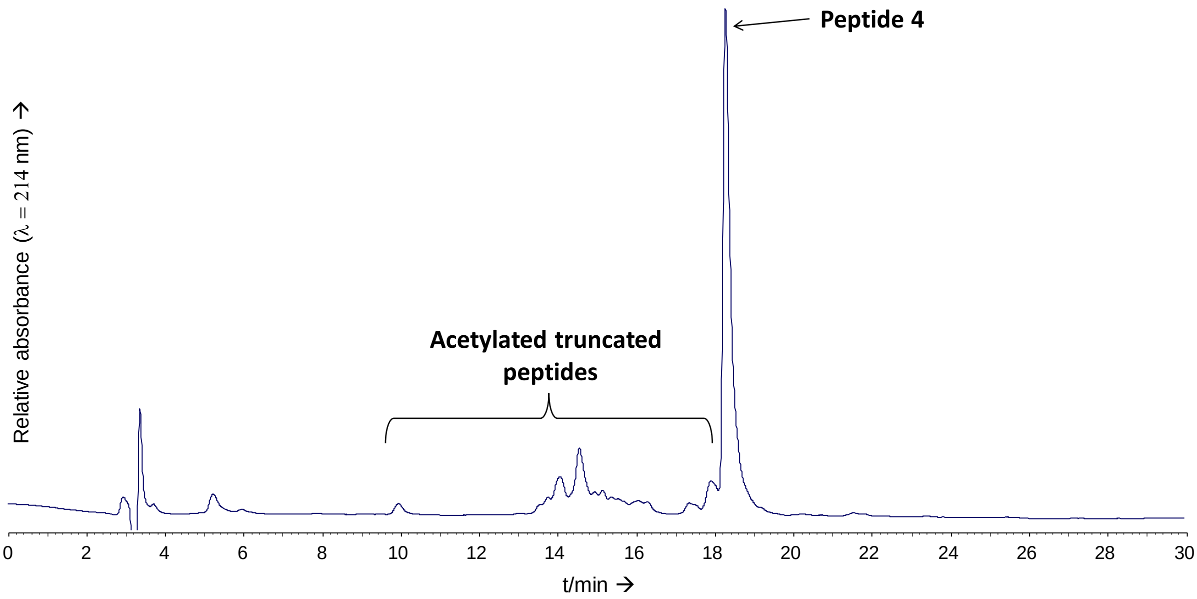

Supplementary figure S34 - Analytical HPLC profile of crude peptide **4**. Column: Nucleosil®; gradient: from 30% to 60% B over 30 min.

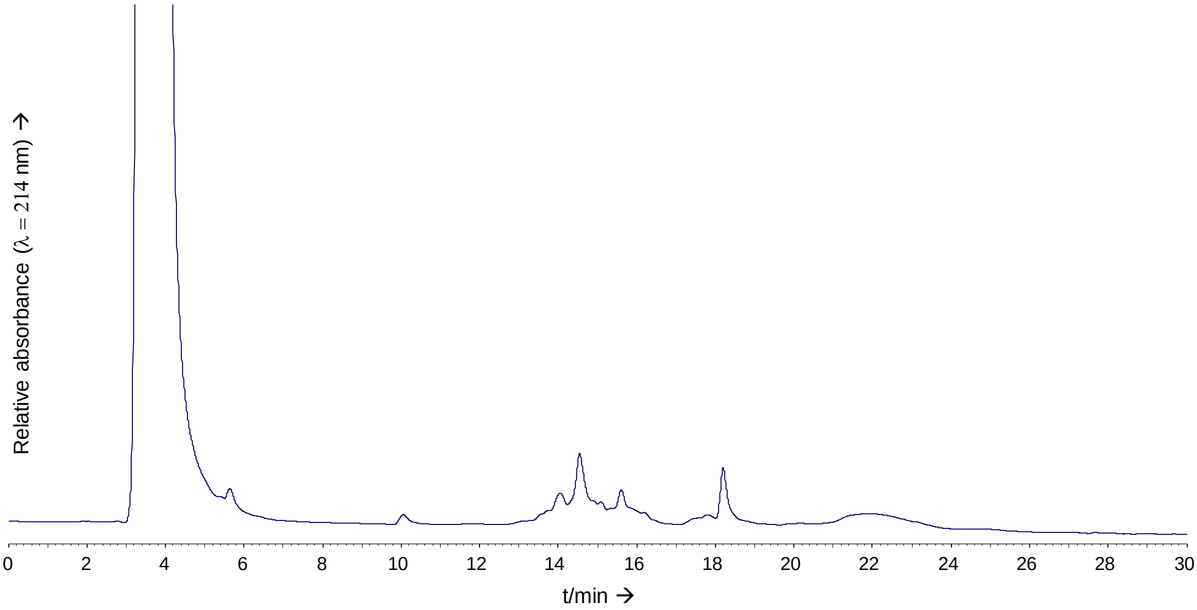

Supplementary figure S35 - Analytical HPLC profile of the crude cycloaddition mixture after 5 hour showing the total consumption of peptide **4** leaving unreacted the acetylated truncated peptides coproducts. Column: Nucleosil®; gradient: from 30% to 60% B over 30 min.

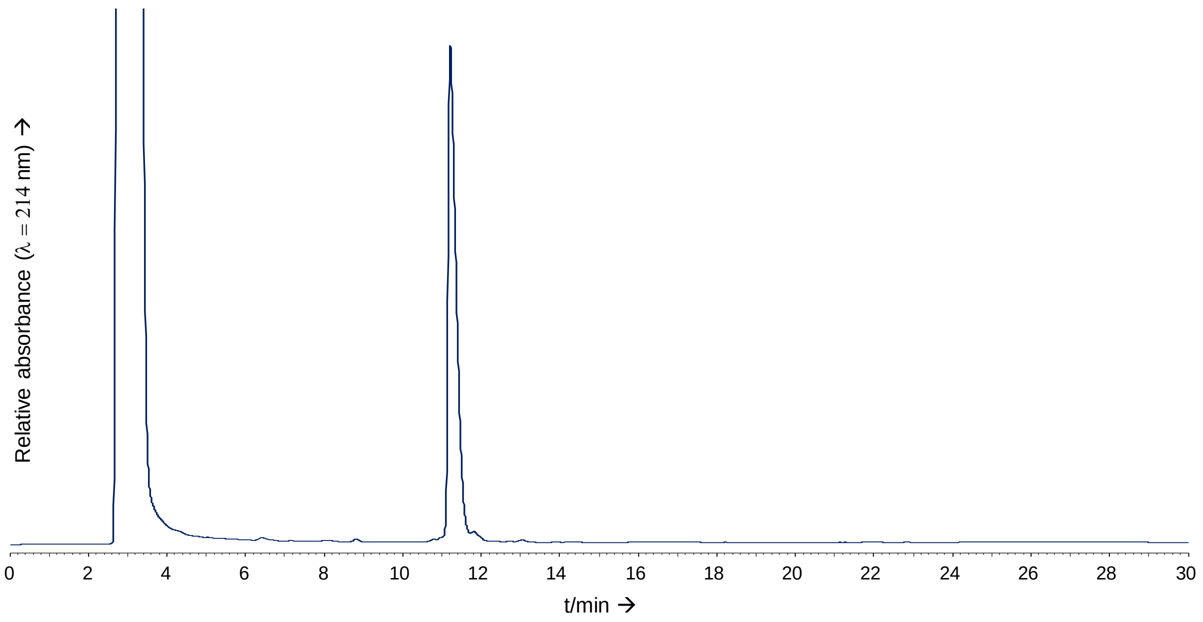

Supplementary figure S36 - Analytical HPLC profile of crude peptide **S8** after treatment of the peptide support with typical procedure described above (p S53). Column: Nucleosil®; gradient: from 30% to 60% B over 30 min.

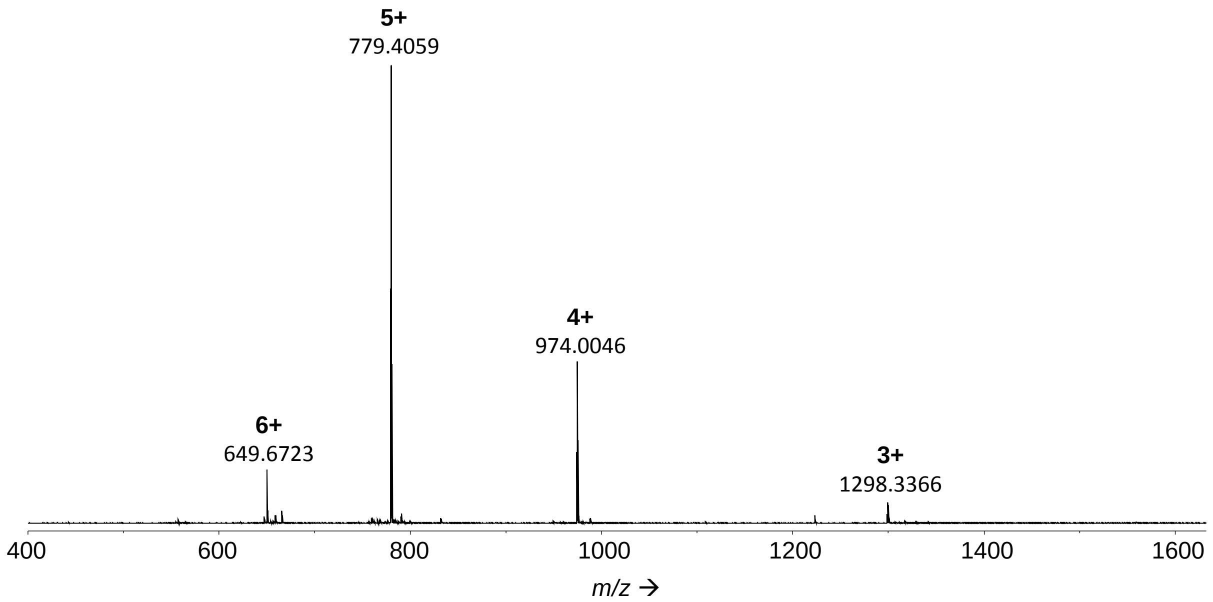

Supplementary figure S37 - ESI-HRMS mass spectrum of peptide **S8**

F.4 Solid-supported deprotection of the TIPS group

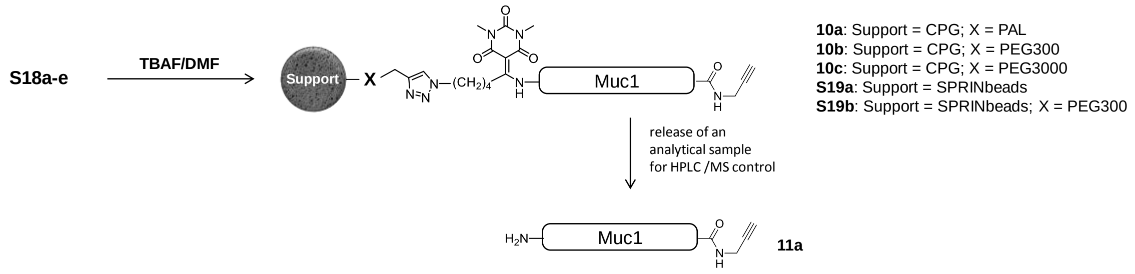

Supplementary scheme S23 – Solid supported deprotection of the TIPS group

The solid supported peptide was introduced in a syringe fitted with a polypropylene frit and a teflon stopcock and was washed with anhydrous DMF (4x). A mixture of TBAF (100 equiv.) dissolved in anhydrous DMF (final concentration 100 mM) was added and the reaction mixture was stirred by syringe rotation for 15 min at room temperature. This protocol was repeated once and the resin was then extensively washed with DMF, a 0.1% aqueous TFA solution and finally de-ionized water.

A few beads of the resulting solid supported peptide were cleaved to give crude peptide **11a** which was analyzed by RP-HPLC and HRMS.

ESI-HRMS (*m/z*): $[M+H]^+$ calcd for $C_{161}H_{253}N_{50}O_{53}$: 3734.8639, found: 3734.8459.

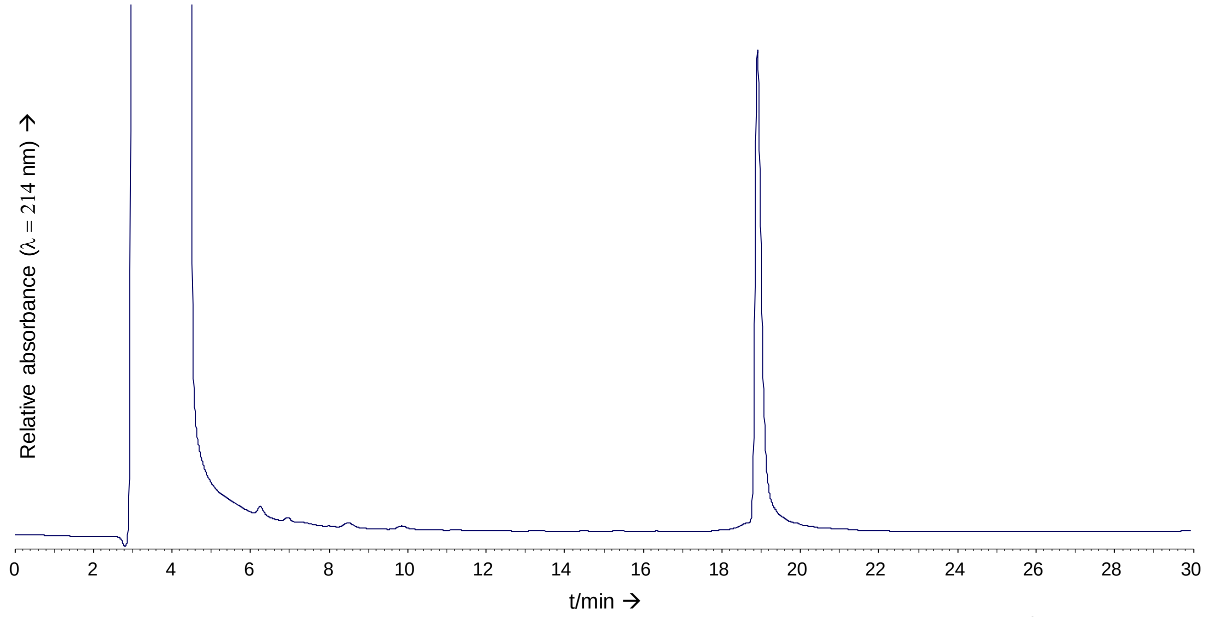

<u>Supplementary figure S38</u> - Analytical HPLC profile of crude peptide **11a**. Column: Nucleosil®; gradient: from 10% to 25% B over 30 min.

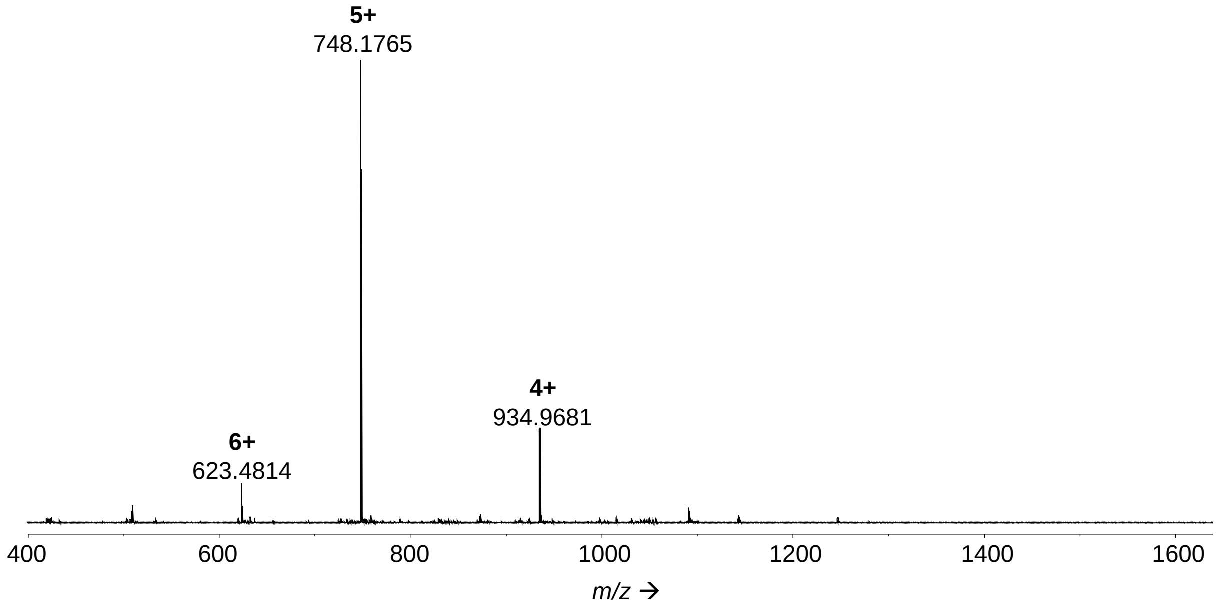

<u>Supplementary figure S39</u> - ESI-HRMS mass spectrum of peptide **11a**

F.5 Quantification of the yield of the catch-and-release process yield

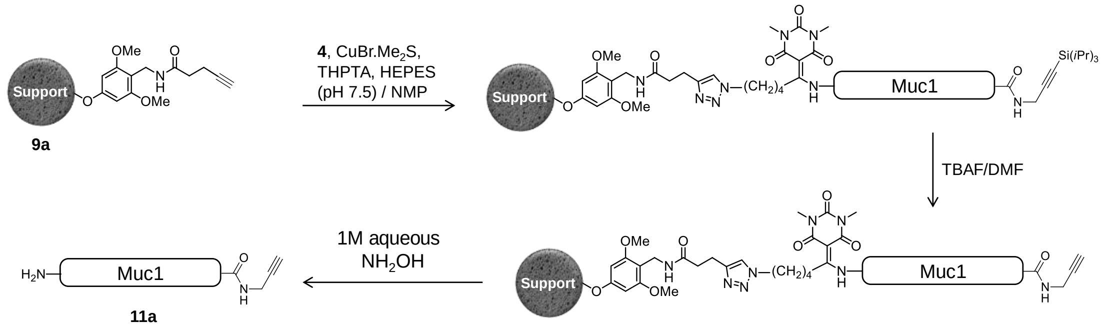

Supplementary scheme S24 – Grafting, alkyne deprotection and releasing of peptide **4** on alkyne functionalized solid support **9a**

Purified azido peptide **4** (5.01 mg, 1.09 µmol[a]) was grafted on alkyne support **9a** (100 mg) following the typical procedure (p S54). Then, solid-supported deprotection of the TIPS group was performed following the typical procedure (p S57). The whole resulting solid supported peptide were cleaved using the general procedure (p S53). A third cleavage with 1M aqueous NH_2OH followed by a TFA cleavage was performed but no trace of peptide was observed by HPLC analysis. Released peptide was isolated from the mixture using a hydrophobic SPE cartridge to give pure peptide **11a** as a white fluffy powder (4.14 mg, 0.99 µmol, 91% [b]).

[a]: estimated considering a MW = 4611 g/mol taking into account 4 trifluoroacetate counter-anions, as predicted from the sequence: 2Arg, 2His = +4 overall charge. Water content is not taken into account.

[b]: estimated considering a MW = 4192 g/mol taking into account 4 trifluoroacetate counter-anions, as predicted from the sequence: 2Arg, 2His = +4 overall charge. Water content is not taken into account.

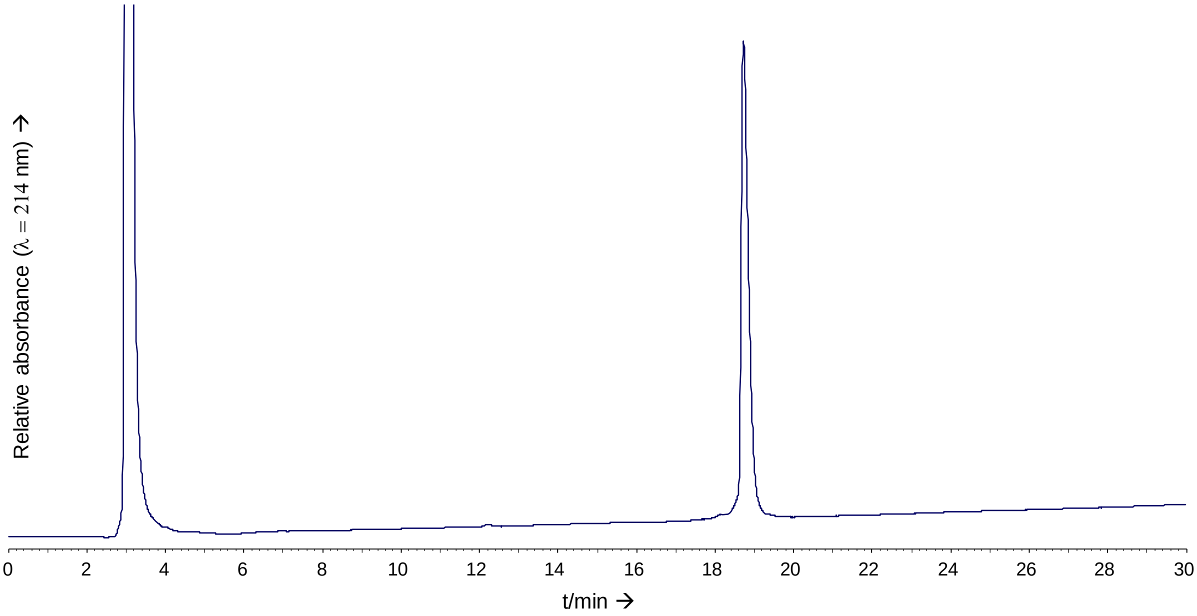

Supplementary figure S40 - Analytical HPLC profile of crude peptide **11a**. Column: Nucleosil®; gradient: from 30% to 60% B over 30 min.

F.6 Enzymatic glycosylation

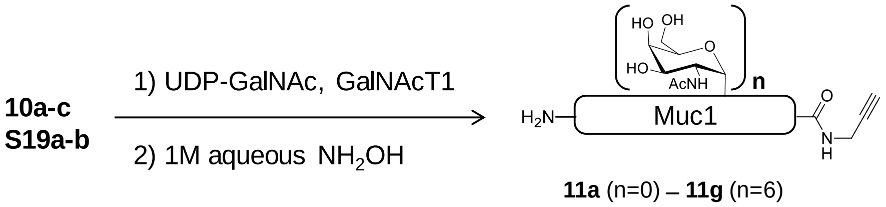

Supplementary scheme S25 - Enzymatic glycosylation and release of solid supported peptide

Solid supported peptide (20 nmol) was introduced in a 2 mL microcentrifuge tube and washed with de-ionized water (2 x 500 µL). To the solid was added 100 µL of an aqueous solution containing recombinant α-*N*-acetylgalactosaminyl transferase T1[12] (GalNAc-T1, 6 mU), MES (50 mM, pH 6.5), $MnCl_2$ (15 mM), Bovine Serum albumin (BSA, 100 ng), DTT (1 mM) and UDP-GalNAc (2mM). The resulting suspension was mixed at 37 °C for 18 h under moderate agitation. Beads were then washed with de-ionized water (2 x 500 µL), in order to remove the UDP formed during the transfer reaction since it inhibits GalNAc-T1 activity. Solid supported peptide was then incubated under the same conditions as above for another 18 h and the resin was washed with de-ionized water.

The resulting solid supported peptide was released under typical procedure (p S53) to give crude peptide which was analyzed by RP-HPLC and HRMS.

Glycopeptide	Number of GalNAc (n)	Formula	$[M+H]^+$ calcd	$[M+H]^+$ found
**11g**	6	$C_{209}H_{331}N_{56}O_{83}$	4953.3402	4953.3472
**11f**	5	$C_{201}H_{318}N_{55}O_{78}$	4750.2608	4750.2671
**11e**	4	$C_{193}H_{305}N_{54}O_{73}$	4547.1814	4547.1833
**11d**	3	$C_{185}H_{292}N_{53}O_{68}$	4344.1020	4344.1183
**11c**	2	$C_{177}H_{279}N_{52}O_{63}$	4141.0227	4141.0334
**11b**	1	$C_{169}H_{266}N_{51}O_{58}$	3937.9433	3937.9235
**11a**	0	$C_{161}H_{253}N_{50}O_{53}$	3734.8639	3734.8459

Supplementary table S5 - ESI-HRMS analysis of **11a-g**

[12] T. Freire, R. Lo-Man, F. Piller, V. Piller, C. Leclerc, S. Bay, *Glycobiology* **2006**, *16*, 390–401.

					GalNAc units added per peptide [a]							
Entry	Solid supported peptide	Support	Loading (µmol/g)	Spacer	7 **(11h)**	6 **(11g)**	5 **(11f)**	4 **(11e)**	3 **(11d)**	2 **(11c)**	1 **(11b)**	0 **(11a)**
1	**10a**	CPG	20	PAL	-[b]	-	-	2%	41%	55%	2%	-
2	**10a**	CPG	10	PAL	-	-	-	22%	58%	20%	-	-
3	**10b**	CPG	20	PEG 300	-	2%	9%	33%	36%	17%	3%	-
4	**10b**	CPG	10	PEG 300	-	8%	19%	47%	18%	8%	-	-
5	**10c**	CPG	20	PEG 3000	-	37%	50%	9%	4%	-	-	-
6	**10c**	CPG	10	PEG 3000	-	40%	48%	8%	4%	-	-	-
7	**S19a**	SPRINbeads	20	-	-	5%	12%	27%	26%	21%	3%	6%
8	**S19a**	SPRINbeads	10	-	-	7%	15%	34%	20%	16%	3%	5%
9	**S19b**	SPRINbeads	10	PEG 300	-	3%	13%	29%	26%	21%	2%	6%

[a] Estimated by integration of the corresponding LC-MS peaks at λ = 214 nm. The differences in molar absorption coefficient due to the added GalNAc moieties are not taken into account, thus overestimating a little the glycosylated compounds. [b] An hyphen indicates that the eventually detected amount of the corresponding product is below 2%.

Supplementary table S6 - Optimization of the solid support for enzymatic glycosylation.

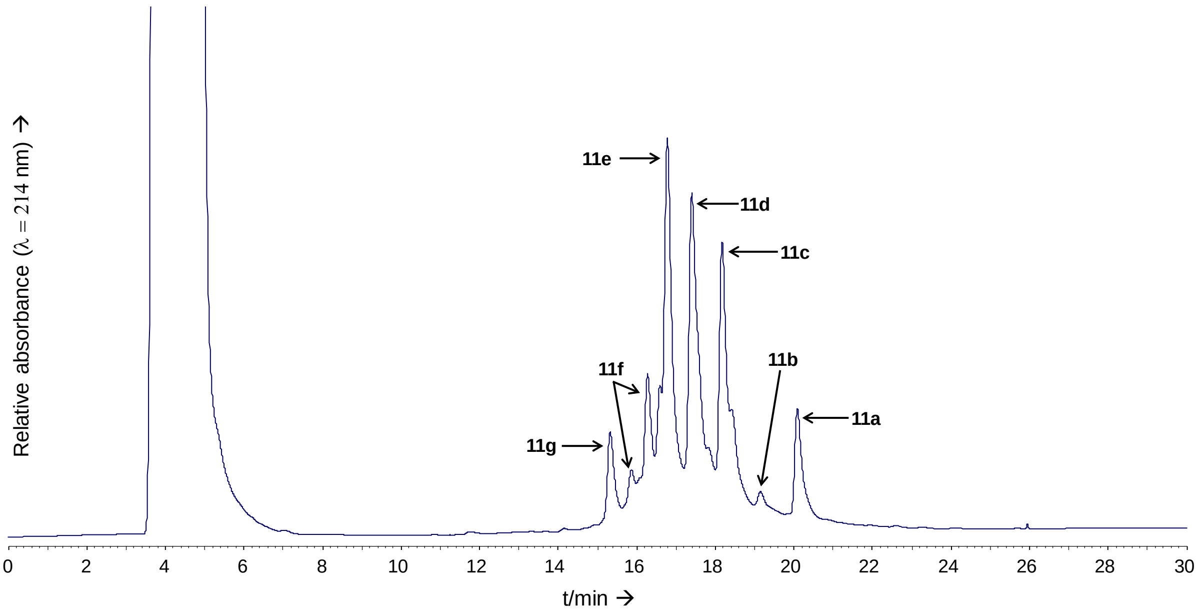

Supplementary figure S41 – Typical analytical HPLC profile of glycopeptides obtained after release from solid support **S19a** (corresponding to entry 8 of Table S5). Column: Nucleosil®; gradient: from 10% to 25% B over 30 min.

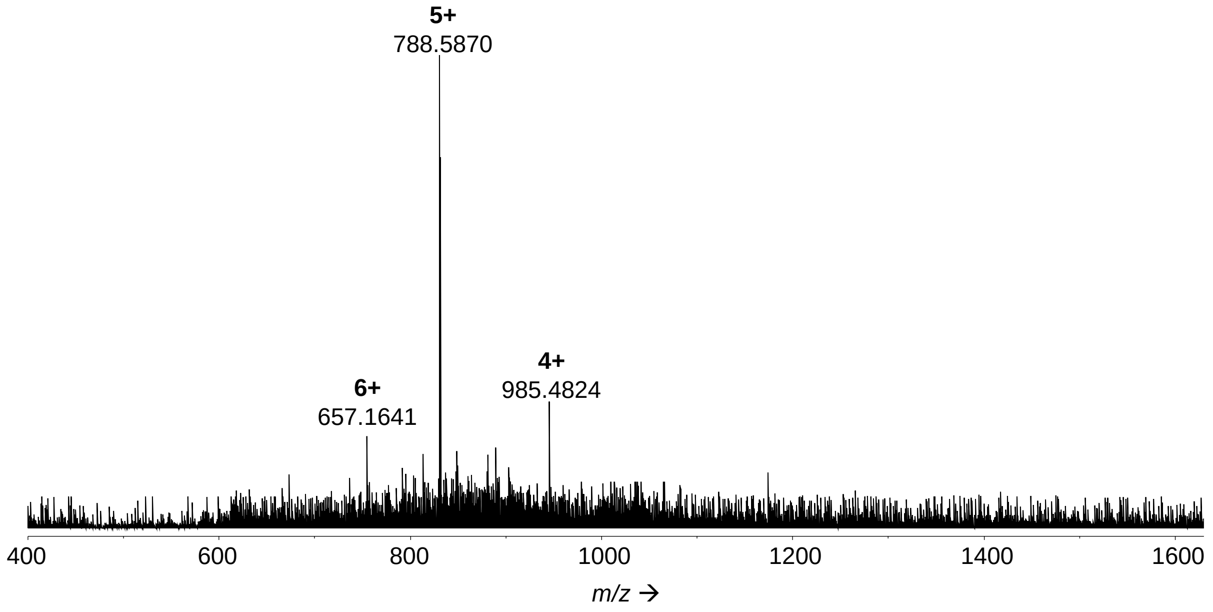

Supplementary figure S42 - ESI-HRMS mass spectrum of glycopeptide **11b** (1 *O*-GalNAc)

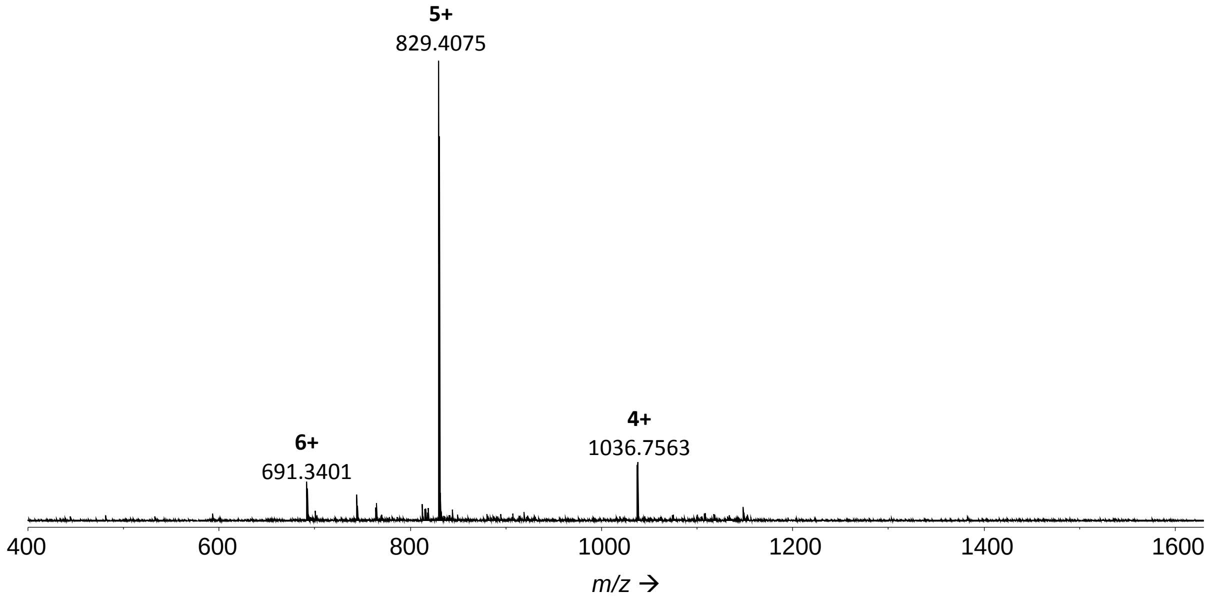

Supplementary figure S43 - ESI-HRMS mass spectrum of glycopeptide **11c** (2 *O*-GalNAc)

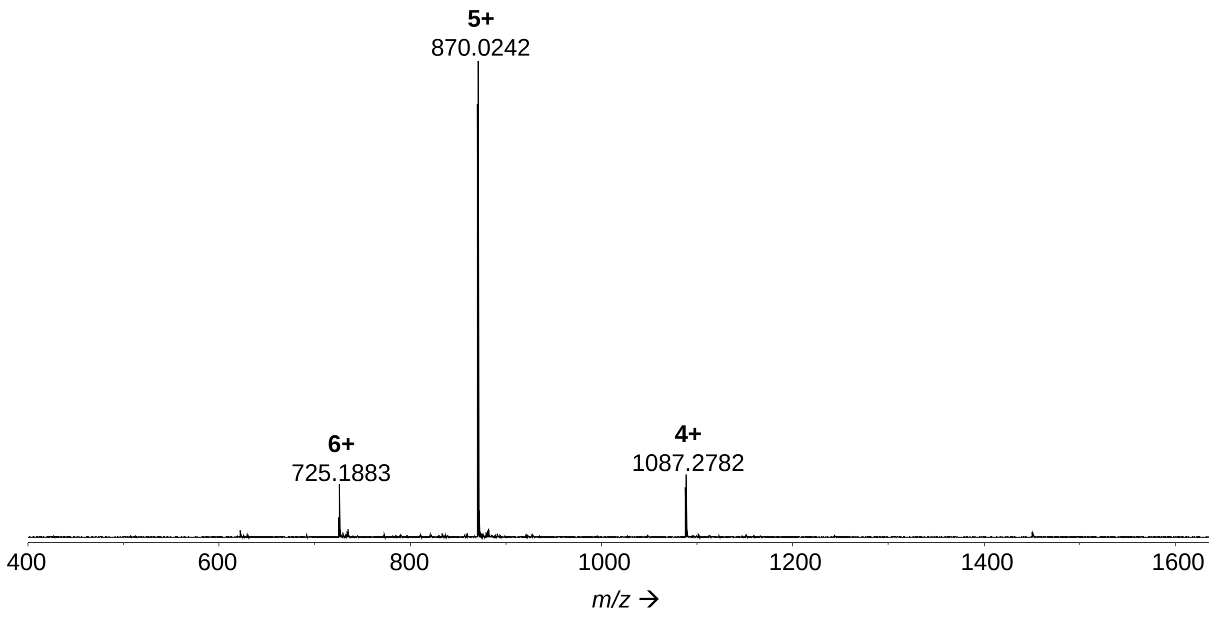

Supplementary figure S44 - ESI-HRMS mass spectrum of glycopeptide **11d** (3 *O*-GalNAc)

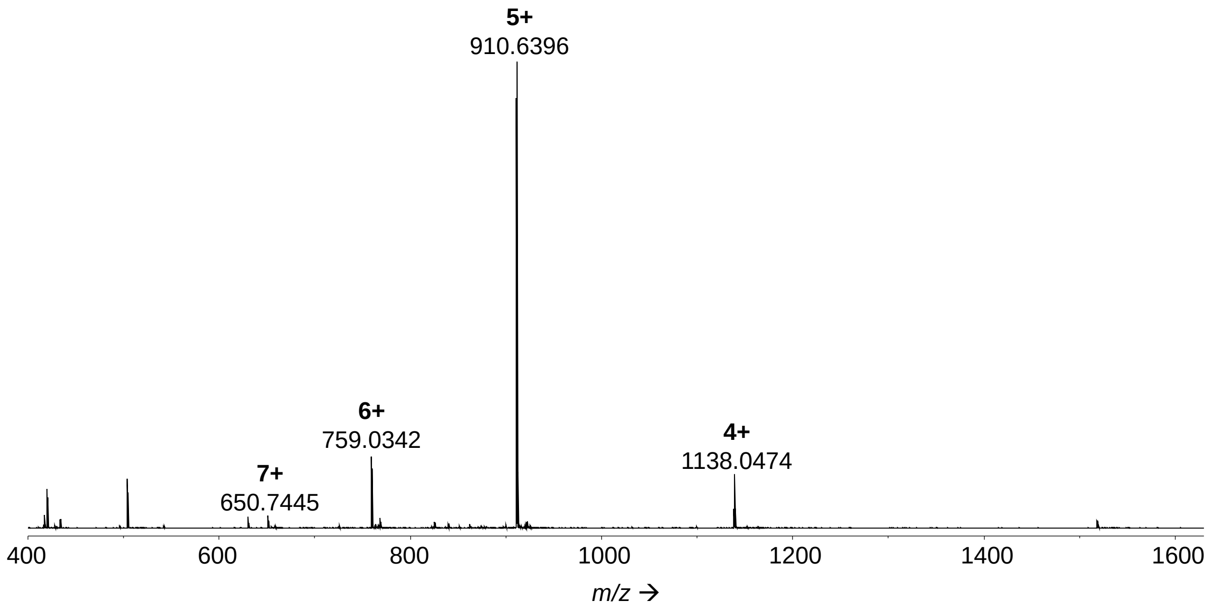

Supplementary figure S45 - ESI-HRMS mass spectrum of glycopeptide **11e** (4 *O*-GalNAc)

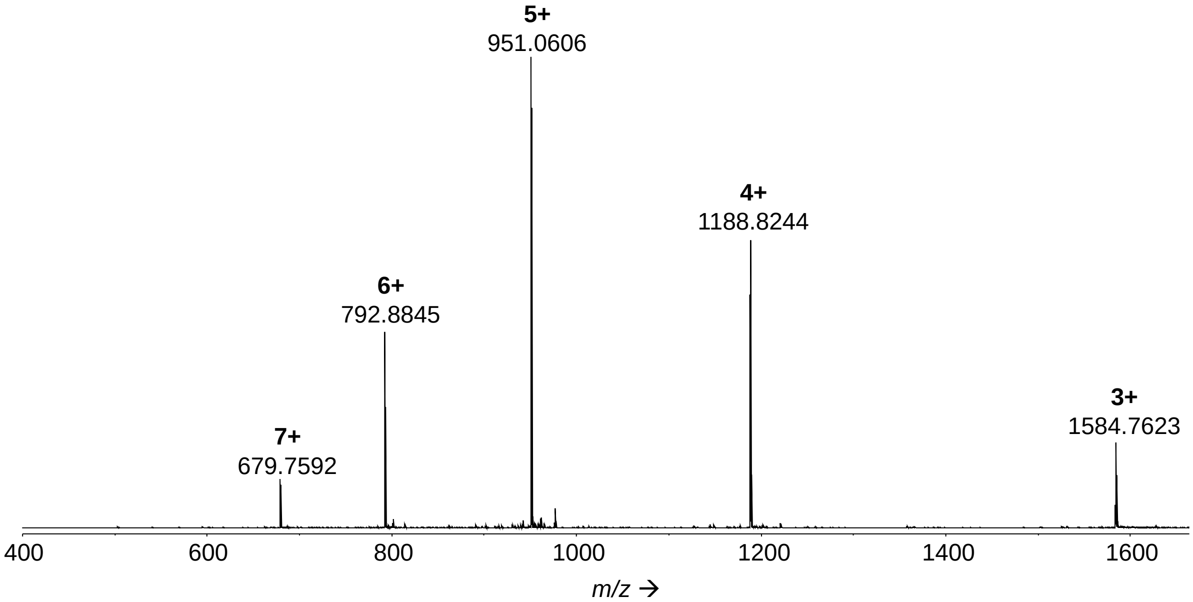

Supplementary figure S46 - ESI-HRMS mass spectrum of glycopeptide **11f** (5 *O*-GalNAc)

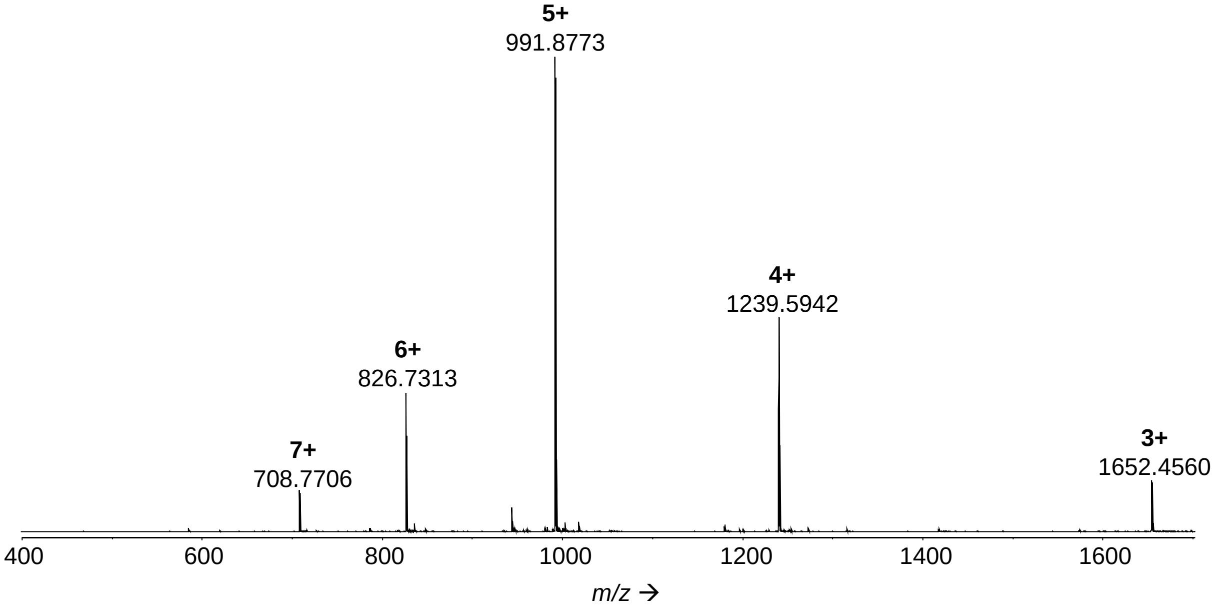

Supplementary figure S47 - ESI-HRMS mass spectrum of glycopeptide **11g** (6 *O*-GalNAc)

F.7 Enzymatic glycosylation in solution

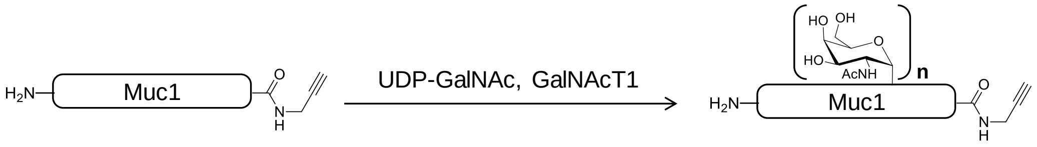

11a **11a** (n=0) – **11g** (n=6)

Supplementary scheme S26 – Enzymatic glycosylation of peptide Muc1 **11a** in solution

In a 2 mL microcentrifuge tube containing peptide **11a** (20 nmol) was added 100 µL of an aqueous solution containing GalNAc-T1 (6 mU), MES (50 mM, pH 6.5), $MnCl_2$ (15 mM), Bovine Serum albumin (BSA, 100 µg), DTT (1 mM) and UDP-GalNAc (2mM). The resulting suspension was mixed at 37 °C for 18 h under moderate agitation. UDP formed during GalNAc transfer was eliminated by addition of calf intestinal alkaline phosphatase (1U). Then 100 µL of fresh GalNAc-T1 enzyme mixture were added for another 18 h period. Resulting glycopeptides were isolated from the mixture using standard hydrophobic SPE cartridge and was analyzed by RP-HPLC and HRMS.

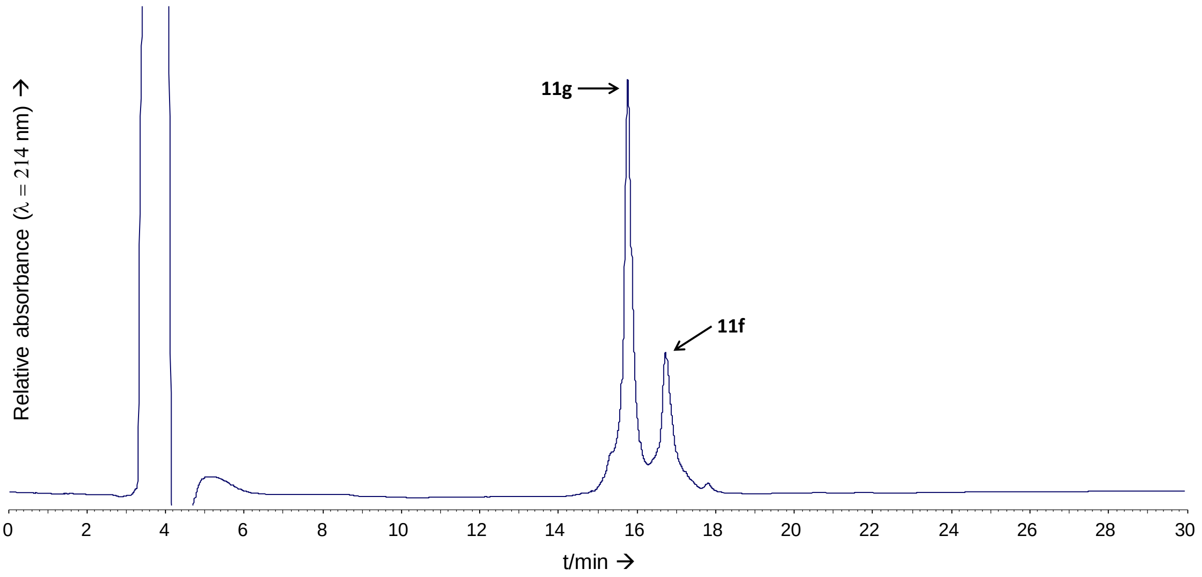

Supplementary figure S48 - Analytical HPLC profile of glycopeptides obtained after enzymatic glycosylation of peptide **11a** in solution. Column: Nucleosil®; gradient: from 10% to 25% B over 30 min.

F.8 Kinetics of enzymatic glycosylation

Kinetics experiments were performed with solid supported peptide **10c**. Conditions used for kinetics experiments on beads were identical to those described above, and reactions were stopped at different time points by washing the beads with water. Addition of freshly prepared enzyme and substrate solution was performed after 1 h, 3 h, 6 h, 12 h, 24 h, 48 h and 96 h while beads were left under moderate agitation at 37 °C for the indicated time.

			GalNAc units added per peptide [a]							
Entry	Reaction duration	Addition of Enzyme	7 (**11h**)	6 (**11g**)	5 (**11f**)	4 (**11e**)	3 (**11d**)	2 (**11c**)	1 (**11b**)	0 (**11a**)
1	1	1	-[b]	-	-	3%	9%	38.0%	16%	34%
2	3	2	-	-	5%	14%	20%	46%	7%	7%
3	6	3	-	2%	12%	22%	25%	34%	3%	2%
4	12	4	-	9%	29%	26%	21%	15%	-	-
5	24	5	-	25%	45%	16%	11%	3%	-	-
6	48	6	-	45%	46%	6%	3%	-	-	-
7	96	7	-	61%	39%	-	-	-	-	-

[a] Estimated by integration of the corresponding LC-MS peaks at λ = 214 nm. The differences in molar absorption coefficient due to the added GalNAc moieties are not taken into account, thus overestimating a little the glycosylated compounds. [b] An hyphen indicates that the eventually detected amount of the corresponding product is below 2%.

Supplementary table S7 - Kinetics of enzymatic glycosylation

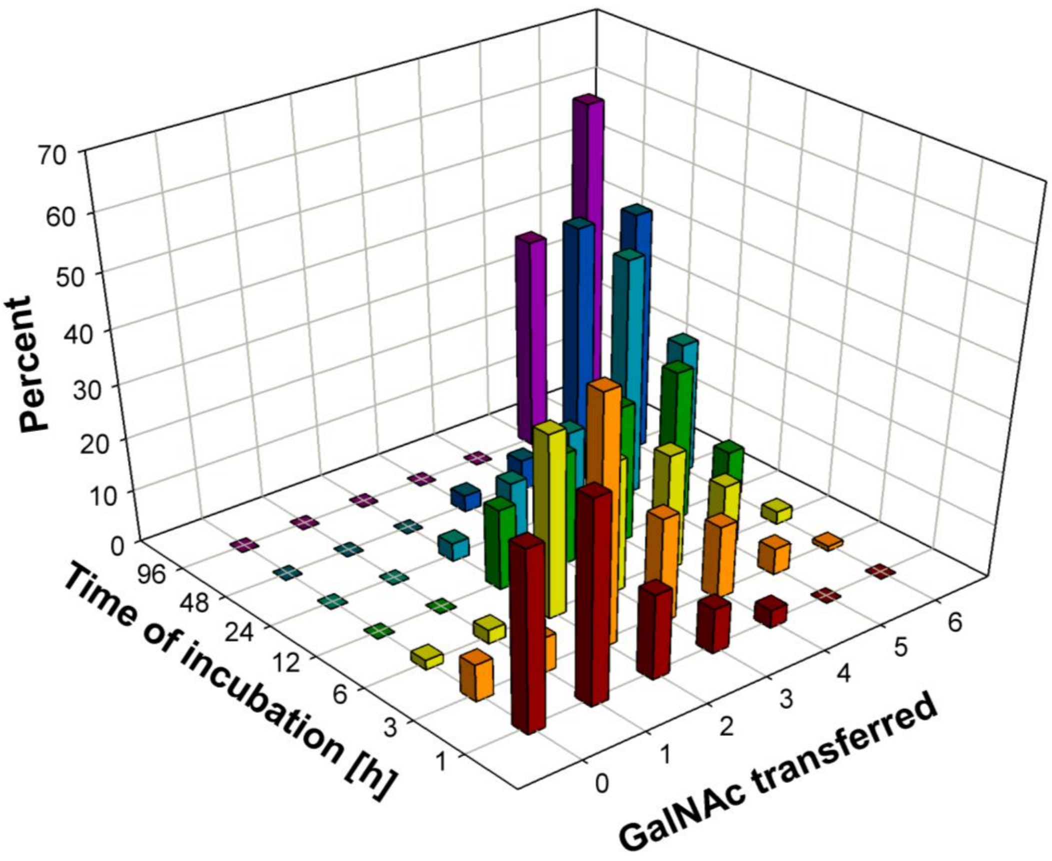

Supplementary figure S49 - Kinetics of enzymatic glycosylation

F.9 Optimization of reaction conditions for enzymatic glycosylation

Attempts realized in order to improve the glycosylation score by addition of 0.1% (*v*/*v*) of Triton™-X100 (Sigma–Aldrich) to the enzymatic mixture, showed no significative change in GalNAc incorporation.

			GalNAc units added per peptide [a]							
Entry	Reaction duration	Addition of Triton™	7 (**11h**)	6 (**11g**)	5 (**11f**)	4 (**11e**)	3 (**11d**)	2 (**11c**)	1 (**11b**)	0 (**11a**)
1	1×24 h	-	-[b]	23%	43%	17%	12%	4%	-	-
2	1×24 h	Yes	-	26%	48%	16%	7%	3%	-	-
3	2×24 h	-	-	36%	48%	10%	5%	-	-	-
4	2×24 h	Yes	-	43%	48%	6%	3%	-	-	-
5	2×48 h	-	-	61%	39%	-	-	-	-	-
6	2×48 h	Yes	-	64%	36%	-	-	-	-	-
7	2×36 h	-	-	59%	41%	-	-	-	-	-
8	4×18 h	-	-	56%	44%	-	-	-	-	-
9	1×144 h	-	-	59%	41%	-	-	-	-	-

[a] Estimated by integration of the corresponding LC-MS peaks at λ = 214 nm. The differences in molar absorption coefficient due to the added GalNAc moieties are not taken into account, thus overestimating a little the glycosylated compounds. [b] An hyphen indicates that the eventually detected amount of the corresponding product is below 2%.

Supplementary table S8 - Optimization of reaction conditions for enzymatic glycosylation

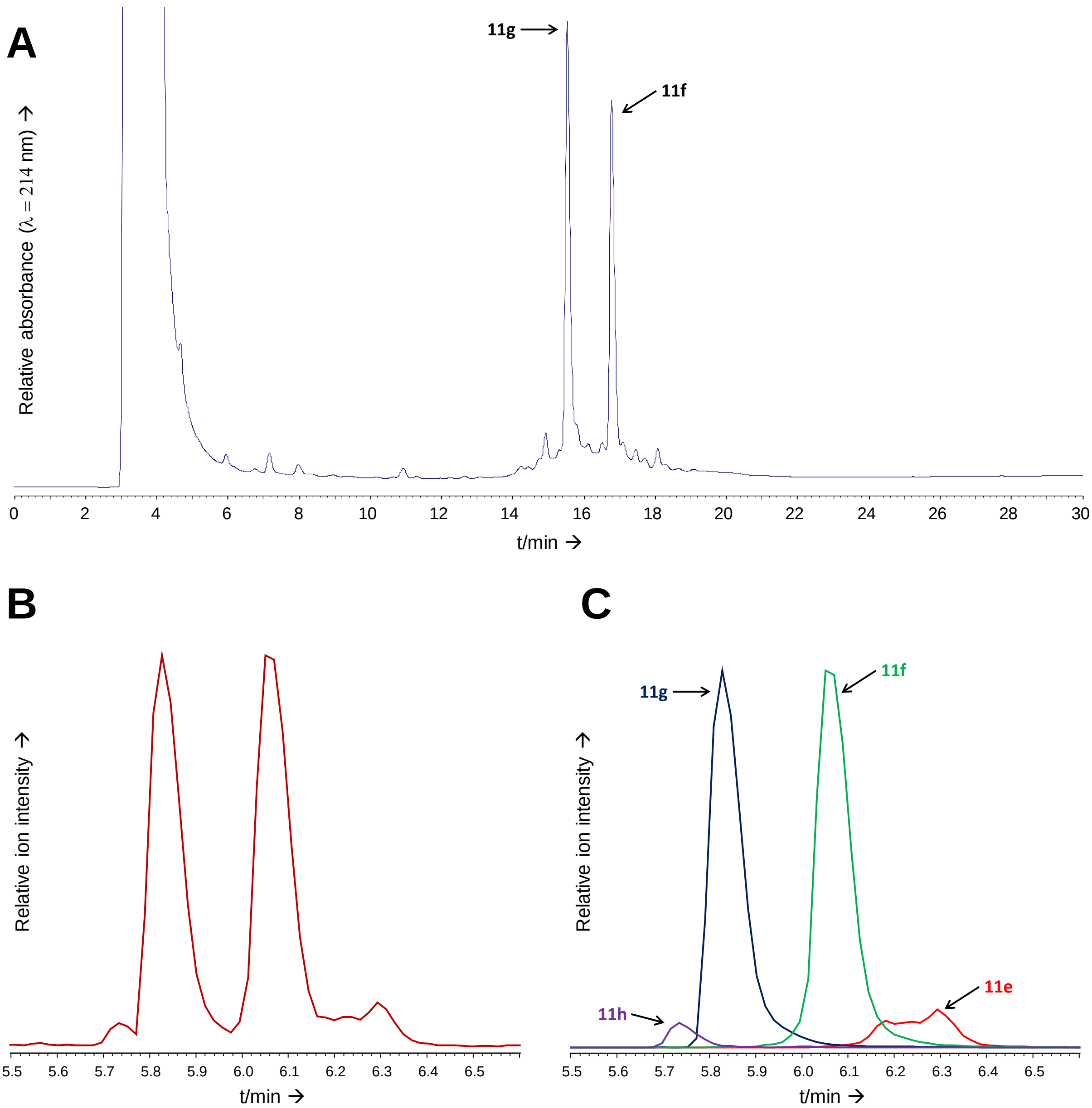

Supplementary figure S50 – **A**) Analytical HPLC profile of glycopeptides obtained after release from solid support **10c** (corresponding to entry 5 of Table S7). Column: Nucleosil®; gradient: from 10% to 25% B over 30 min. **B**) Total-ion chromatogram **C**) Extracted-ion chromatogram of compound **11h**, **11g**, **11f** and **11e**. Column: Zorbax 300; gradient: from 3% to 50% B over 20 min

Compound	GalNAc units added per peptide	Formula	$[M+H]^+$ calcd	$[M+H]^+$ found	Relative abundance[a]
**11h**	7	$C_{217}H_{344}N_{57}O_{88}$	5156.4195	5156.4212	1.9%
**11g**	6	$C_{209}H_{331}N_{56}O_{83}$	4953.3402	4953.3472	48.3%
**11f**	5	$C_{201}H_{318}N_{55}O_{78}$	4750.2608	4750.2671	42.5%
**11e**	4	$C_{193}H_{305}N_{54}O_{73}$	4547.1814	4547.1833	7.3%

[a] Estimated by peak area integration on extracted-ion chromatograms; not a rigorous quantification, due to probable differences in ionization of the compounds.

F.10 Glycosylation site identification

After release from solid-support, glycopeptide **11g** was purified by semi-preparative HPLC and lyophilized. *O*-glycosylation sites of the glycopeptides were identified by electron transfer dissociation (ETD) ESI-MS/MS analysis by a mass spectrometry as described above.

Product ions	*m/z* expected	*m/z* observed
z_2+1	248.127	248.146
c_2	421.229	421.26
y_4	458.251	458.375
c_3	508.261	508.339
z_5+1	513.269	513.344
c_5	676.351	676.398
c_6	791.378	791.466
$(z_{12}+1)^{+2}$	793.42	793.465
z_6	817.396	817.481
$(z_{13}+1)^{+2}$	843.928	844.011
c_7	892.426	892.489
$(z_{14}+1)^{+2}$	901.412	901.527
c_{14}^{+2}	982.978	983.056
$(z_{16}+1)^{+2}$	985.459	985.553
M^{+5}	991.668	992.068
$(z_{17}+1)^{+2}$	1028.933	1029.062
z_7+1	1107.508	1107.584
c_{17}^{+2}	1115.498	1115.616
c_9	1145.58	1145.686
c_{18}^{+2}	1151.028	1151.097
z_8+1	1164.529	1164.57
c_{29}^{+3}	1208.232	1208.311
c_{19}^{+2}	1219.542	1219.644
$(z_{39}+1)^{+4}$	1235.034	1235.122
M+4	1239.335	1239.425
c_{20}^{+2}	1248.14	1248.136
$(z_{20}+1)^{+2}$	1259.05	1259.178
$(z_{30}+1)^{+3}$	1270.597	1270.617
c_{32}^{+3}	1283.261	1238.349
c_{21}^{+2}	1297.65	1297.655
c_{11}	1313.67	1313.726
z_{21}+1	1327.68	1327.685
z_{10}+1	1332.619	1332.662

Product ions	*m/z* expected	*m/z* observed
$(z_{22}+1)^{+2}$	1363.16	1363.166
c_{12}	1370.691	1370.745
c_{33}^{+3}	1379.969	1379.976
c_{22}^{+2}	1449.697	1449.704
c_{34}^{+3}	1481.284	1481.331
c_{23}^{+2}	1493.205	1493.197
$(z_{25}+1)^{+2}$	1495.724	1495.714
$(z_{37}+1)^{+3}$	1512.047	1511.975
c_{37}^{+3}	1569.672	1569.743
c_{25}^{+2}	1577.263	1577.257
c_{38}^{+3}	1593.352	1593.399
$(z_{38}+1)^{+3}$	1613.422	1613.515
$(z_{39}+1)^{+3}$	1646.376	1646.451
M^{+3}	1651.78	1651.91
$(z_{27}+1)^{+2}$	1792.833	1792.836
c_{29}^{+2}	1811.842	1811.851
$(z_{30}+1)^{+2}$	1905.391	1905.411
c_{32}^{+2}	1924.391	1924.394
c_{14}	1964.929	1964.949
z_{16}+1	1969.938	1969.91
z_{17}+1	2056.97	2056.858
c_{34}^{+2}	2221.542	2221.429
$(z_{37}+1)^{+2}$	2267.566	2267.492
c_{18}	2301.109	2301.048
c_{37}^{+2}	2354.364	2354.487
c_{38}^{+2}	2389.6325	2389.519
$(z_{39}+1)^{+2}$	2469.16	2469.054
M^{+2}	2477.166	2477.244
z_{20}+1	2517.187	2517.093
c_{22}	2898.385	2898.387
c_{23}	2985.417	2985.394
z_{25}+1	2990.425	2990.423

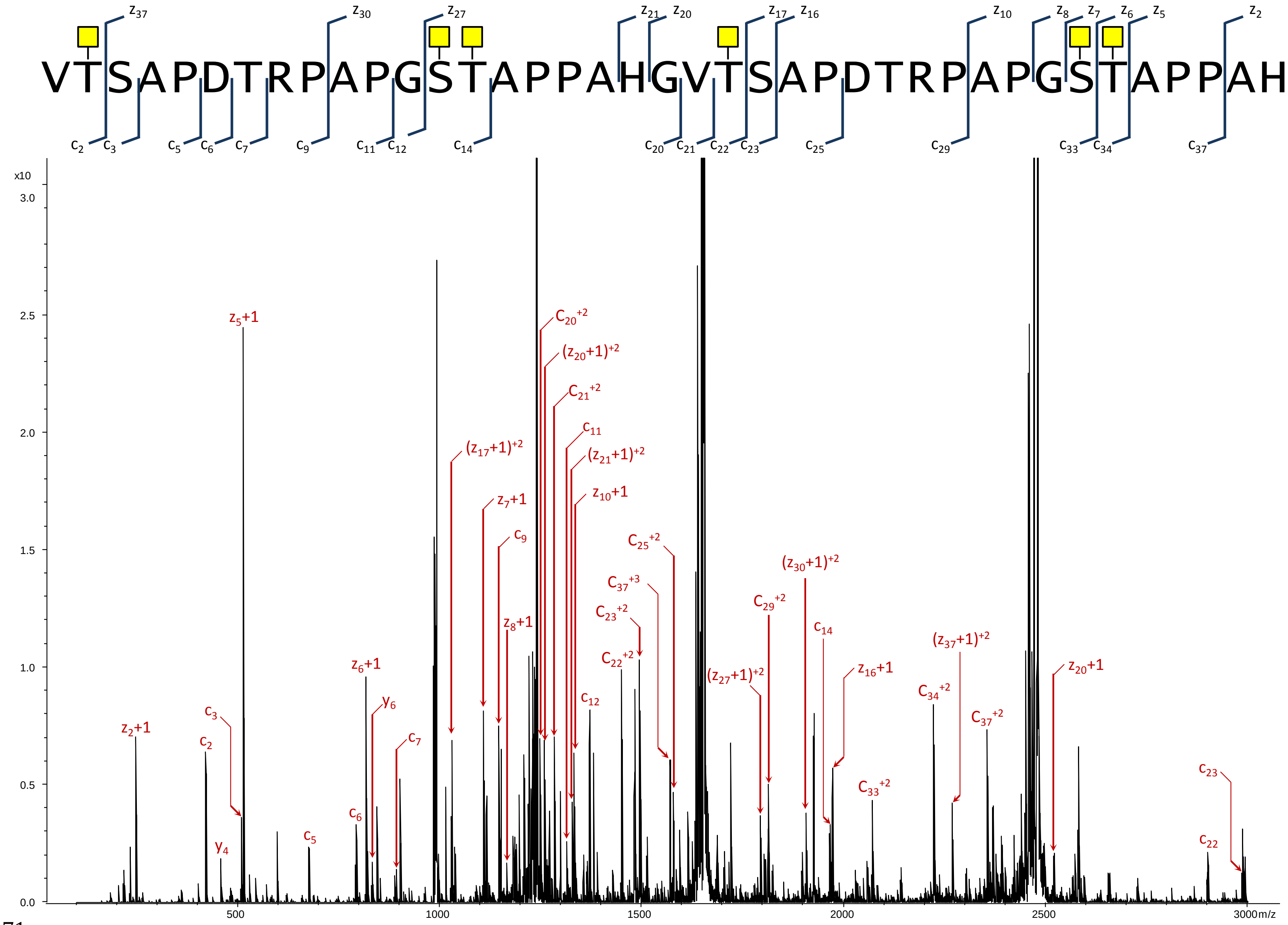

VTSAPDTRPAPGSTAPPAHGVTSAPDTRPAPGSTAPPAH
z37 z30 z27 z21 z20 z17 z16 z10 z8 z7 z6 z5 z2
c2 c3 c5 c6 c7 c9 c11 c12 c14 c20 c21 c22 c23 c25 c29 c33 c34 c37
x10
3.0
2.5
2.0
1.5
1.0
0.5
0.0
500
1000
1500
2000
2500
3000m/z
z2+1
c2
c3
y4
z5+1
c5
c6
z6+1
y6
c7
(z17+1)+2
z7+1
c9
z8+1
C20+2
(z20+1)+2
C21+2
c11
(z21+1)+2
z10+1
c12
C25+2
C37+3
C23+2
C22+2
(z27+1)+2
C29+2
(z30+1)+2
c14
z16+1
C33+2
C34+2
(z37+1)+2
C37+2
z20+1
c23
c22

S71

G Solid phase enzymatic glycosylation of chemically-glycosylated peptide 6

G.1 Grafting of peptide 6 on alkyne functionalized solid support 9c

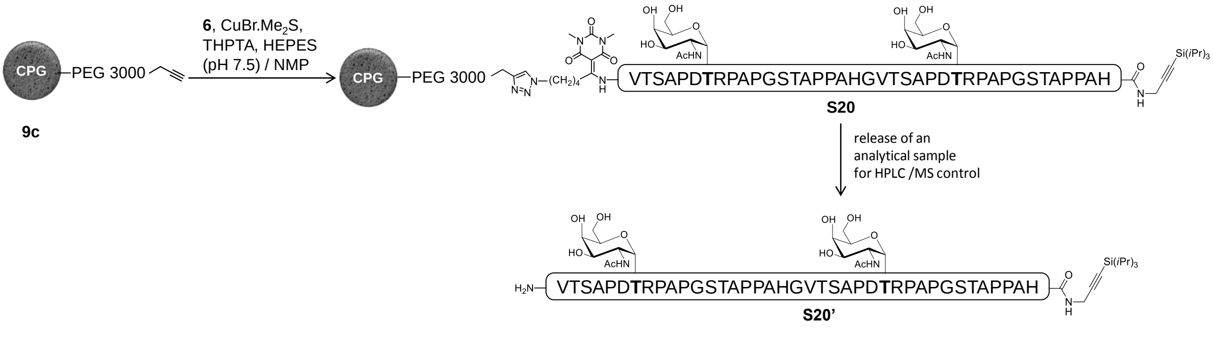

Supplementary scheme S27 - Grafting of peptide **6** on alkyne functionalized solid support **9c**

A solution of the azide-containing glycopeptide **6** (1 µmol) dissolved in 800 µL of a 1:1 mixture of NMP and 200 mM HEPES buffer pH 7.5 was added to a 2 mL microcentrifuge tube containing the alkyne functionalized solid support **9c** (100 mg). To the suspension were subsequently added a 1 M aqueous aminoguanidine solution (30 µL, 30 equiv.), a 1 M aqueous *tert*-butanol solution (30 µL, 30 equiv.). The tube was sealed with a rubber septum and the resulting mixture was further deoxygenated through several successive vacuum (15 mbar) / argon cycles. Then, a mixture containing copper(I) bromide-dimethyl sulfide complex (30 equiv.) and THPTA (40 equiv.) dissolved under argon in 50 µL of deoxygenated NMP was added, the resulting suspension was further deoxygenated and was stirred for 5 h at 37 °C. The supernatant was analyzed by HPLC to check the total consumption of the azidopeptide. Capping of remaining solid-supported terminal alkyne was performed by adding 2-(2-azido-ethoxy)-ethanol (100 equiv.). The mixture was stirred an additional hour at 37 °C. The resin was then extensively washed with NMP, de-ionized water, then repeatedly treated (3x) with a pH 7 aqueous buffer containing 6 M guanidinium chloride, 0.1 M EDTA and 0.1 M sodium dihydrogenophosphate for a few minutes then drained and finally extensively washed with de-ionized water.

A few beads of the resulting solid supported peptide were cleaved to give crude glycopeptide **S20'** which was analyzed by RP-HPLC and HRMS.

ESI-HRMS (*m/z*): $[M+H]^+$ calcd for $C_{186}H_{299}N_{52}O_{63}Si$: 4297.1561, found: 4297.1514.

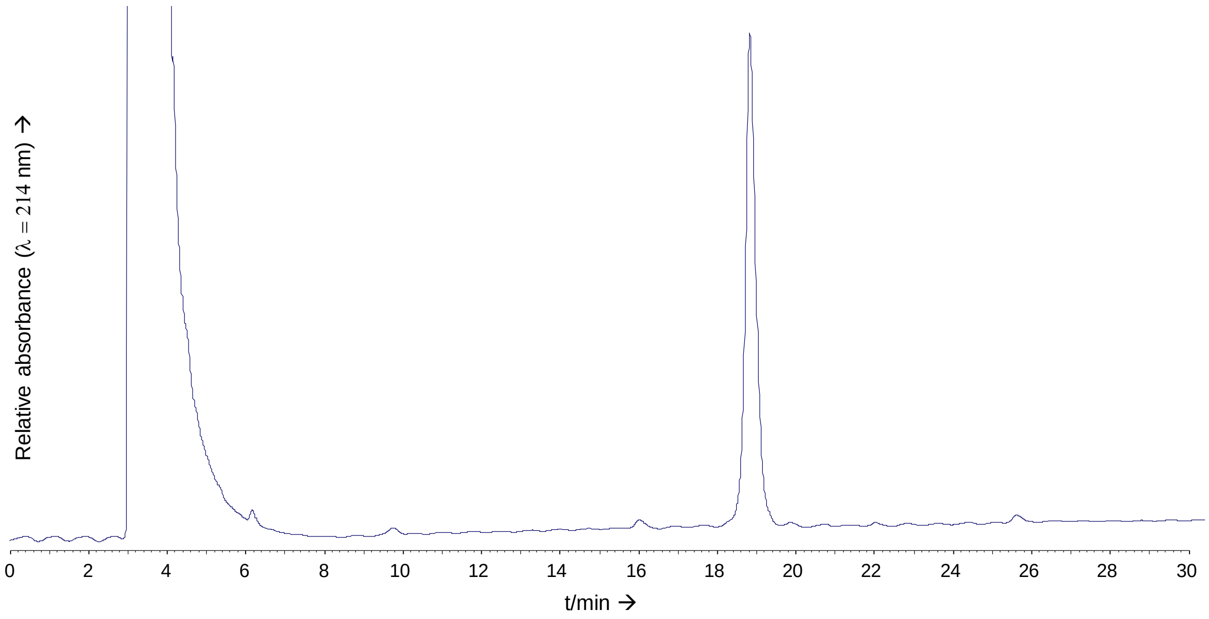

<u>Supplementary figure S51</u> - Analytical HPLC profile of crude glycopeptide **S20’** after treatment of the peptide support with typical procedure described above. Column: Nucleosil®; gradient: from 30% to 60% B over 30 min.

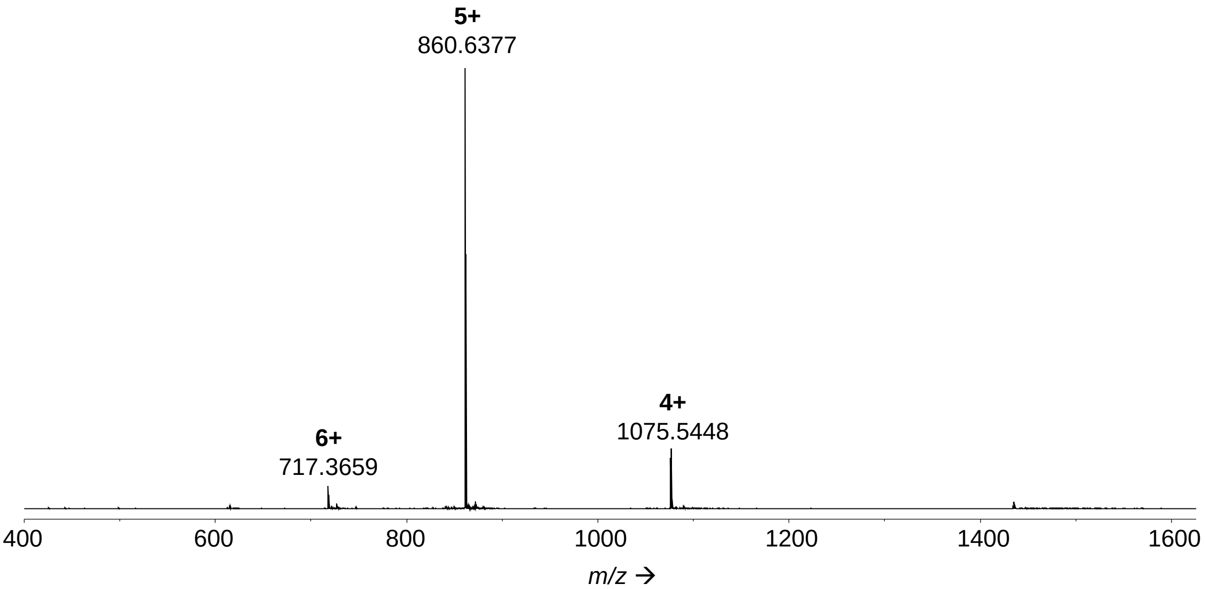

<u>Supplementary figure S52</u> - ESI-HRMS mass spectrum of glycopeptide **S20’**

G.2 Solid-supported deprotection of the TIPS group of 15

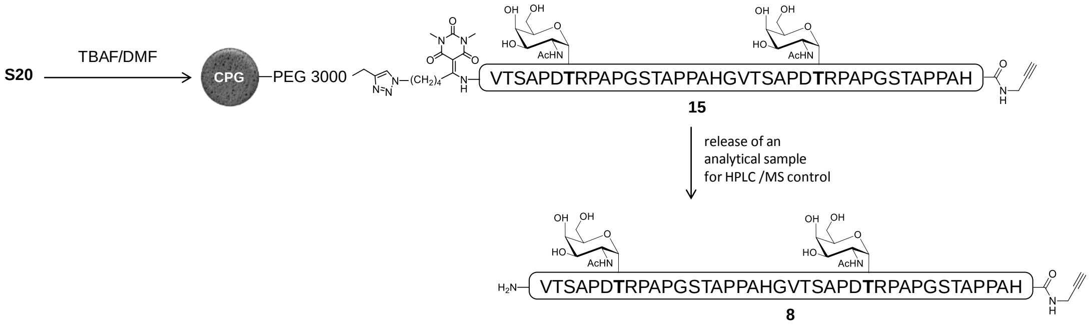

Supplementary scheme S28 - Solid-supported deprotection of the TIPS group of **15**

The solid supported glycopeptide **S20** was introduced in a syringe fitted with a polypropylene frit and a teflon stopcock and was washed with anhydrous DMF (4x). A mixture of TBAF (100 equiv.) dissolved in anhydrous DMF (final concentration 100 mM) was added and the reaction mixture was stirred by syringe rotation for 15 min at room temperature. This protocol was repeated once and the resin was then extensively washed with DMF, a 0.1% aqueous TFA solution and finally de-ionized water to give the solid supported peptide **15**.
A few beads of the resulting solid supported peptide were cleaved to give crude glycopeptide **8** which was analyzed by RP-HPLC and HRMS.

ESI-HRMS (*m/z*): $[M+H]^+$ calcd for $C_{177}H_{279}N_{52}O_{63}$: 4141.0227, found: 4141.0252.

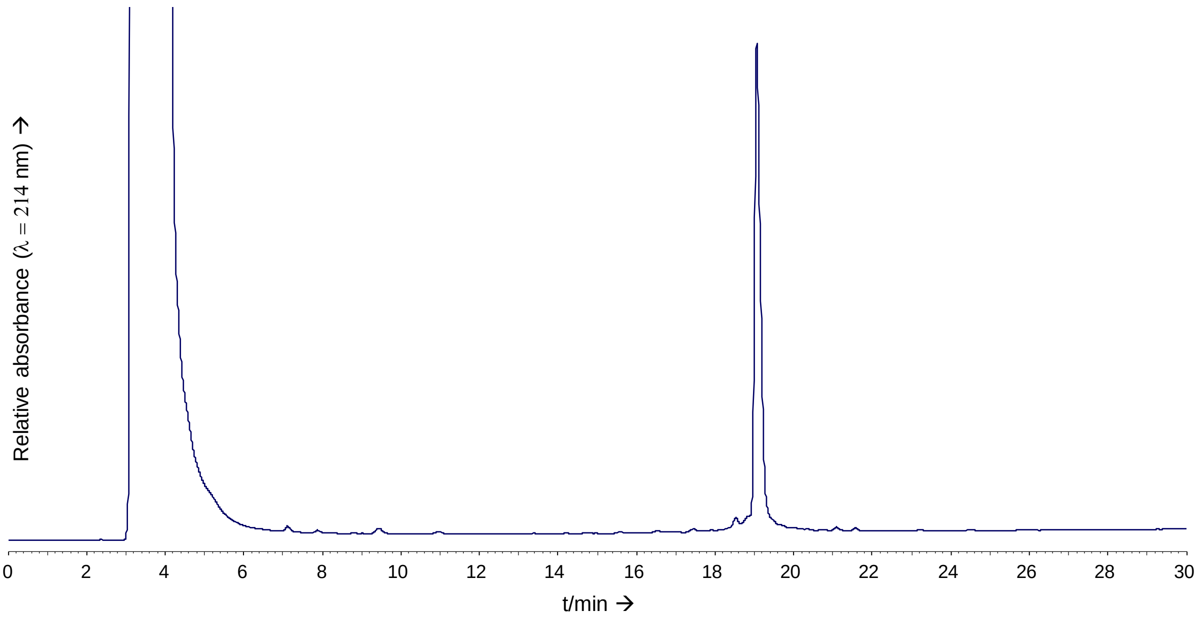

<u>Supplementary figure S53</u> - Analytical HPLC profile of crude glycopeptide **8** released from solid supported peptide **15**. Column: Nucleosil®; gradient: from 10% to 25% B over 30 min.

G.3 Enzymatic glycosylation of 15 with GalNAc-T1

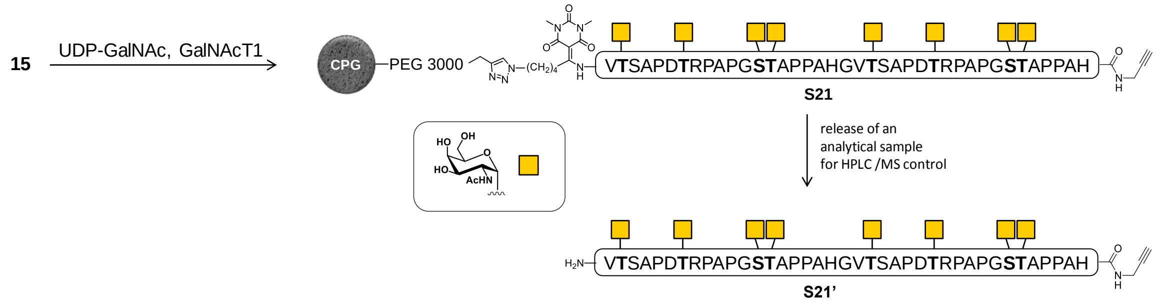

Supplementary scheme S 29 - Enzymatic glycosylation of **15** with GalNAcT1

Solid supported glycopeptide **15** (20 nmol) was introduced in a 2 mL microcentrifuge tube and washed with de-ionized water (2 x 500 µL). To the solid was added 100 µL of an aqueous solution containing GalNAc-T1 (6 mU), MES (50 mM, pH 6.5), $MnCl_2$ (15 mM), BSA (100 ng), DTT (1 mM) and UDP-GalNAc (2 mM). The resulting suspension was mixed at 37 °C for 48 h under moderate agitation. Beads were then washed with de-ionized water (2 x 500 µL), in order to remove the UDP formed during the transfer reaction since it inhibits GalNAc-T1 activity. Solid supported glycopeptide was then incubated under the same conditions as above for another 48 h and the resin was washed with de-ionized water.
The resulting solid supported glycopeptide was released under typical procedure (p S53) to give crude glycopeptide **S21'**. Glycopeptide was purified by semi-preparative HPLC and lyophilized to yield glycopeptide **S21'** as a white fluffy powder (Yield: 48.3%; estimated by integration of the HPLC peak at λ = 214 nm)
ESI-HRMS (*m/z*): $[M+H]^+$ calcd for $C_{225}H_{357}N_{58}O_{93}$: 5359.4989, found: 5359.5029.

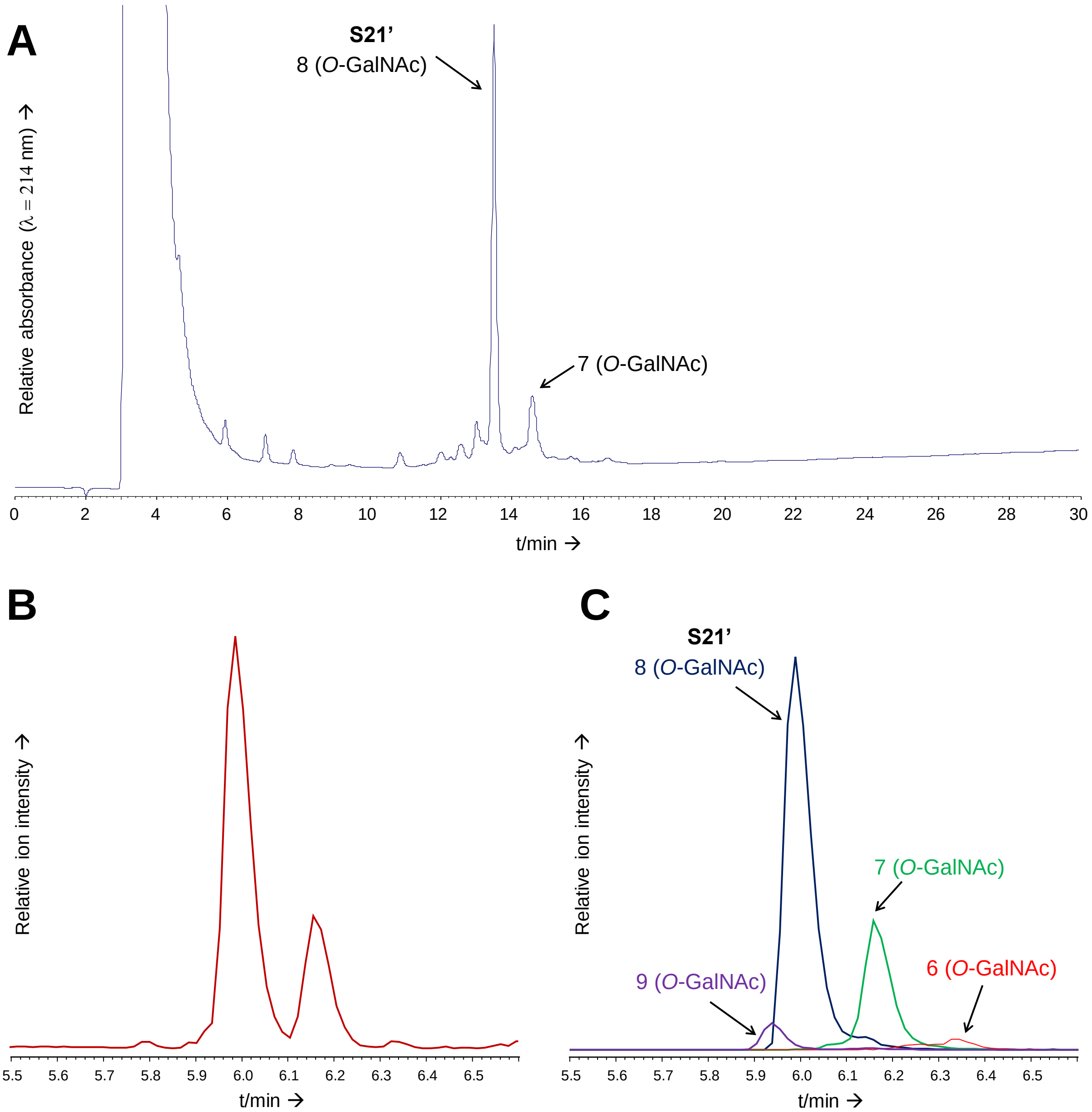

Supplementary figure S54 - Analytical HPLC profile of crude mixture containing glycopeptide **S21’**. Column: Nucleosil®; gradient: from 10% to 25% B over 30 min. **B**) Total-ion chromatogram **C**) Extracted-ion chromatogram of glycopeptides containing 9, 8, 7 and 6 *O*-GalNAc, respectively. Column: Zorbax 300; gradient: from 3% to 50% B over 20 min

- ESI-HRMS analysis of crude mixture containing glycopeptide **S21’**

GalNAc units added per peptide	Formula	$[M+H]^+$ calcd	$[M+H]^+$ found	Relative abundance[a]
9	$C_{233}H_{370}N_{59}O_{98}$	5562.5782	5562.5812	3.9%
8	$C_{225}H_{357}N_{58}O_{93}$	5359.4989	5359.5029	74.4%
7	$C_{217}H_{344}N_{57}O_{88}$	5156.4195	5156.4229	21.3%
6	$C_{209}H_{331}N_{56}O_{83}$	4953.3401	4953.3354	0.4%

[a] Estimated by peak area integration on extracted-ion chromatograms; not a rigorous quantification, due to probable differences in ionization of the compounds.

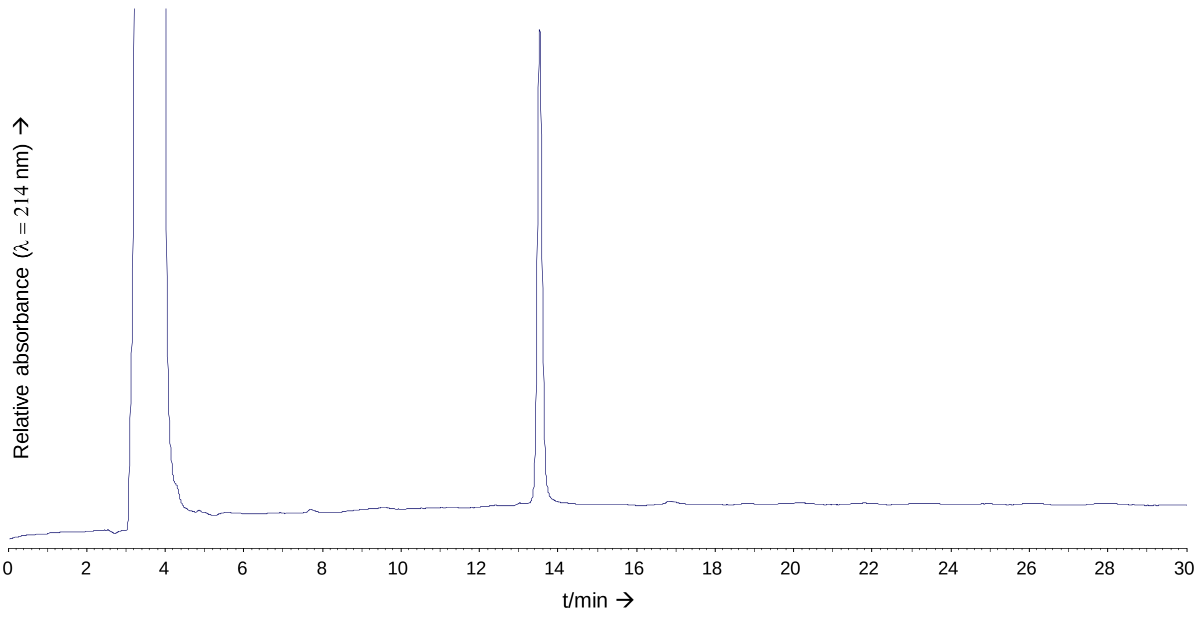

<u>Supplementary figure S55</u> - Analytical HPLC profile of purified glycopeptide **S21'**. Column: Nucleosil®; gradient: from 10% to 25% B over 30 min.

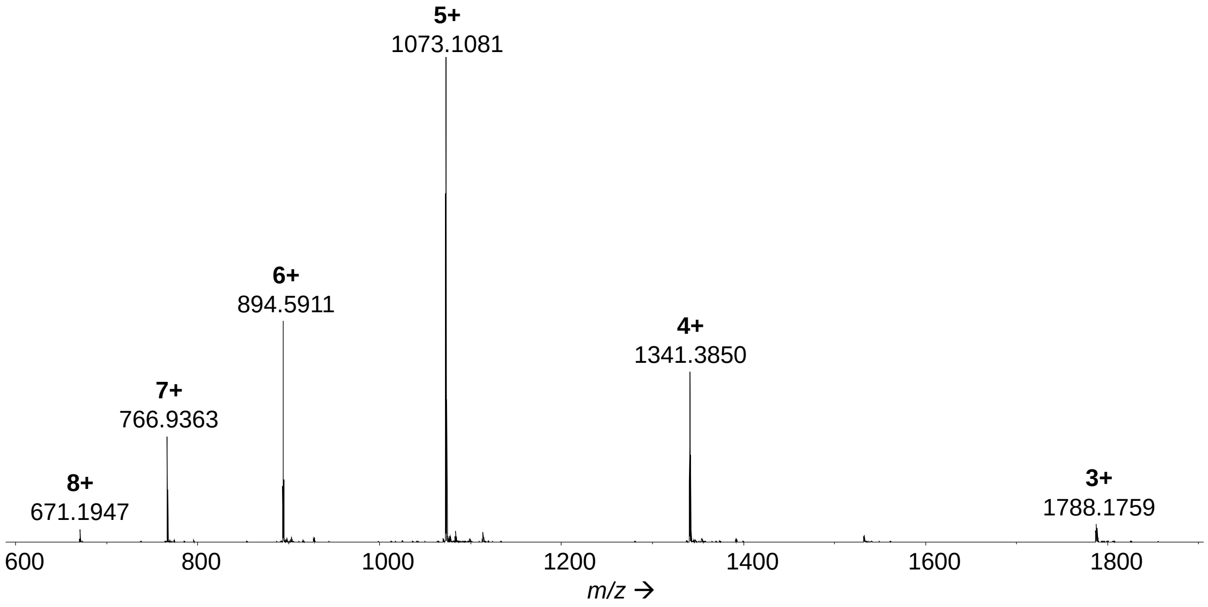

<u>Supplementary figure S56</u> - ESI-HRMS mass spectrum of glycopeptide **S21'**

G.4 Enzymatic sialylation of 15 with ST6GalNAc I

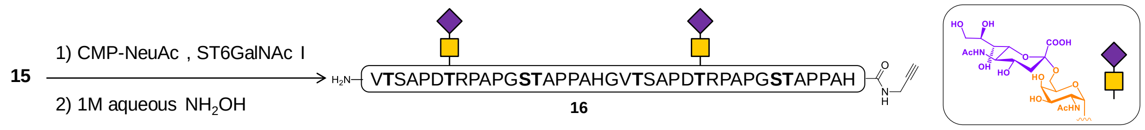

Supplementary scheme S30 - Enzymatic sialylation of **15** with ST6GalNAc I

The catalytic domain of human ST6GalNAc I [13] was expressed in Sf9 insect cells transduced by a recombinant baculovirus and secreted into the medium and was a kind gift of A. Harduin-Lepers. The medium was concentrated 25-fold and the enzymatic activity kept at -20 °C in 30% (*v*/*v*) aqueous glycerol.

Solid supported glycopeptide **15** (20 nmol) was introduced in a 2 mL microcentrifuge tube and washed with de-ionized water (2 x 500 µL). To the solid was added 100 µL of an aqueous solution containing recombinant ST6GalNAc I (8 mU),[14] MES (50 mM, pH 6.8), Triton-X100 (0.8% *v*/*v*), BSA (50 µg), DTT (1 mM) and CMP-NeuAc (4 mM).

The resulting suspension was mixed at 37 °C for 24 h under moderate agitation. Beads were washed with de-ionized water (2 x 500 µL) and were then incubated for another 24 h with a fresh enzyme and substrate mixture. Beads were extensively washed with de-ionized water.

The resulting solid supported glycopeptide was released under typical procedure (p S53) to give a mixture of disialylated glycopeptide **16** (65%) and monosialylated glycopeptide **S22** (30%). **No trace of degradation of the sialyl moiety was observed, even under much prolonged cleavage reaction**. The disialylated glycopeptide **16** was purified by semi-preparative HPLC and lyophilized to yield glycopeptide **16** as a white fluffy powder (Yield: 38.1%; estimated by integration of the HPLC peak at λ = 214 nm).

Disialylated glycopeptide **16**

ESI-HRMS (*m/z*): $[M+H]^+$ calcd for $C_{199}H_{313}N_{54}O_{79}$: 4723.2135, found: 4723.2131.

Monosialylated glycopeptide **S22**

ESI-HRMS (*m/z*): $[M+H]^+$ calcd for $C_{188}H_{296}N_{53}O_{71}$: 4432.1181, found: 4432.1214.

---

[13] S. Julien, E. Adriaenssens, K. Ottenberg, A. Furlan, G. Courtand, A.-S. Vercoutter-Edouart, F.-G. Hanisch, P. Delannoy, X. L. Bourhis, *Glycobiology* **2006**, *16*, 54–64.

[14] One unit of enzyme is defined as the amount of enzyme that transfers 1 µmol of Neu5Ac from CMP-Neu5Ac in 1 minute using the standard reaction mixture -as decsribed in the assay method- but with asialofetuin as acceptor substrate.

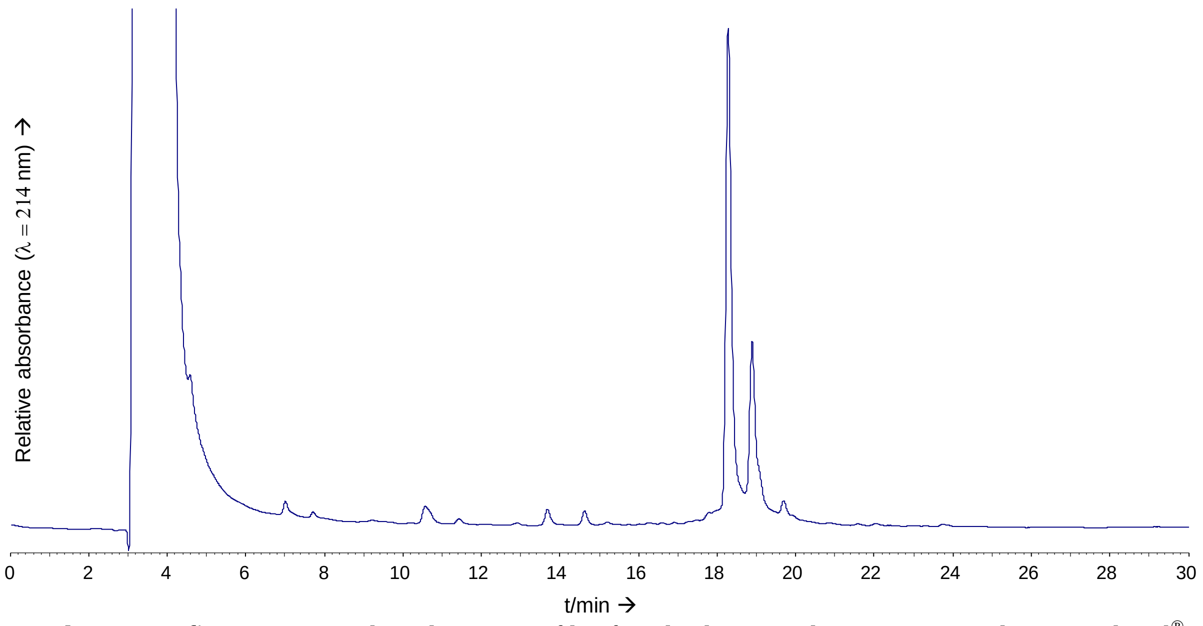

Supplementary figure S57 - Analytical HPLC profile of crude glycopeptides **16** mixture Column: Nucleosil®; gradient: from 10% to 25% B over 30 min.

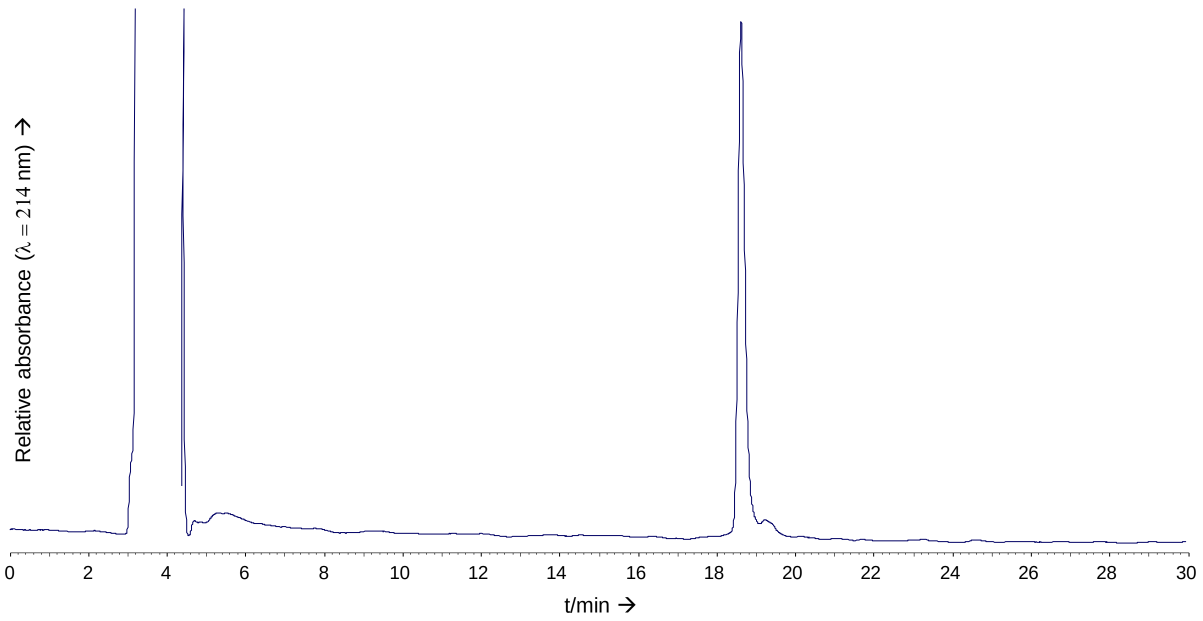

Supplementary figure S58 - Analytical HPLC profile of purified disialylated glycopeptide **16**. Column: Nucleosil®; gradient: from 10% to 25% B over 30 min.

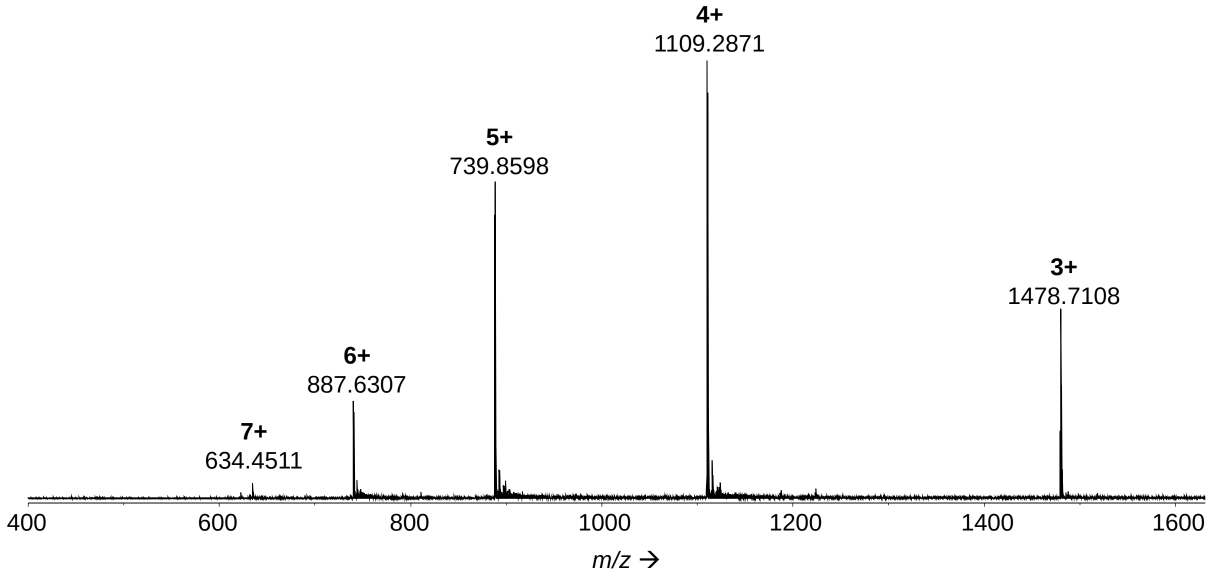

Supplementary figure S59 - ESI-HRMS mass spectrum of disialylated glycopeptide **16**

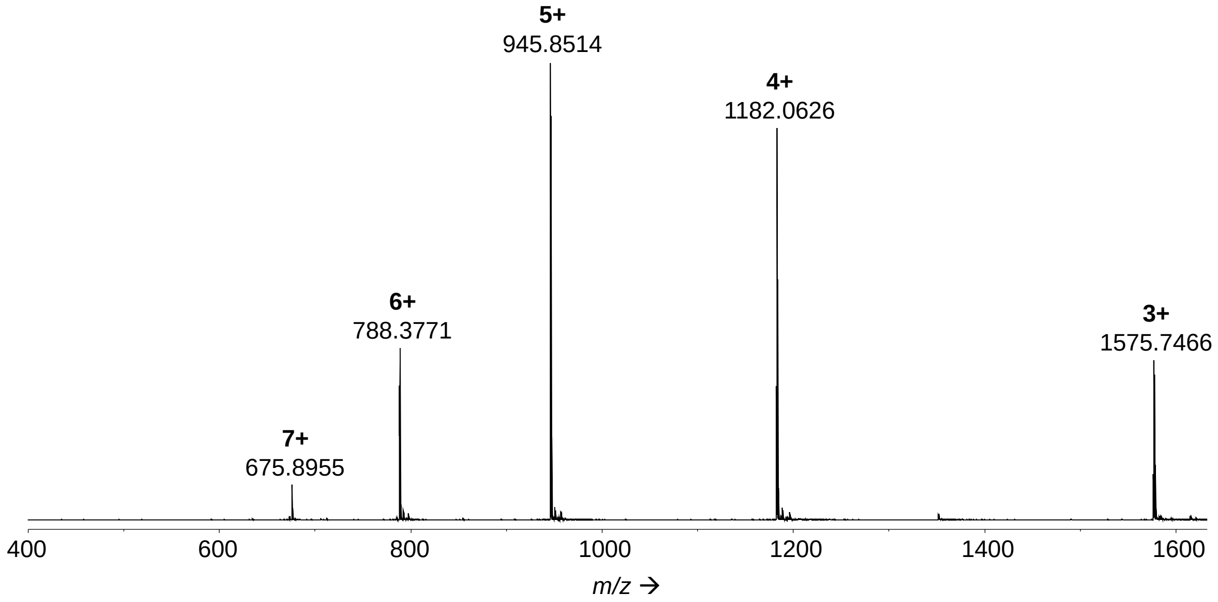

Supplementary figure S60 - ESI-HRMS mass spectrum of monosialylated glycopeptide **S22**

H Chemical ligation to introduce a biotin probe

H.1 Introduction of a biotin probe on solid supported peptide 10c through CuAAc

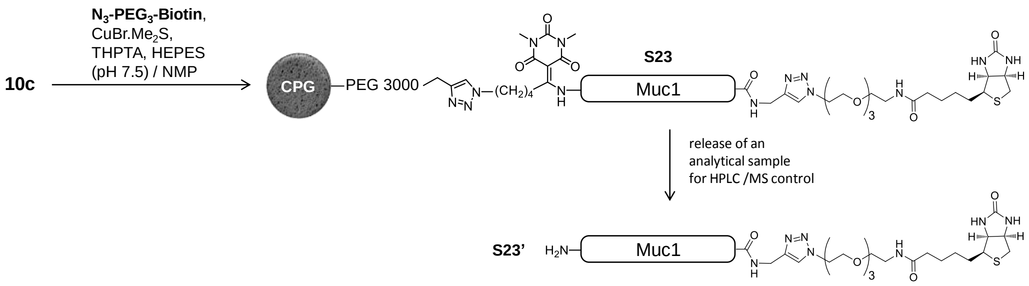

Supplementary scheme S 31 - Introduction of a biotin probe on solid supported peptide **10c**

Solid supported peptide **10c** (100 nmol) was introduced in a 2 mL microcentrifuge tube and washed with de-ionized water (2 x 500 µL). A solution of N_3-PEG$_3$-biotin (450 µg, 1 µmol, 10 equiv) dissolved in 100 µL of a 1:1 mixture of NMP and 200 mM HEPES buffer pH 7.5 was then added. To the suspension were subsequently added a 1 M aqueous aminoguanidine solution (3 µL, 30 equiv.), a 1 M aqueous *tert*-butanol solution (3 µL, 30 equiv.). The tube was sealed with a rubber septum and the resulting mixture was further deoxygenated through several successive vacuum (15 mbar) / argon cycles. Then, a mixture containing copper(I) bromide-dimethyl sulfide complex (30 equiv.) and THPTA (40 equiv.) dissolved under argon in 10 µL of deoxygenated NMP was added, the resulting suspension was further deoxygenated and was stirred for 5 h at 37 °C. The resin was then extensively washed with NMP, de-ionized water, then repeatedly treated (3x) with a pH 7 aqueous buffer containing 6 M guanidinium chloride, 0.1 M EDTA and 0.1 M sodium dihydrogenophosphate for a few minutes then drained and finally extensively washed with de-ionized water.

A few beads of the resulting solid supported peptide were cleaved to give crude peptide **S23'** which was analyzed by RP-HPLC and HRMS.

ESI-HRMS (*m/z*): $[M+H]^+$ calcd for $C_{179}H_{285}N_{56}O_{58}S$: 4179.0794, found: 4179.0760.

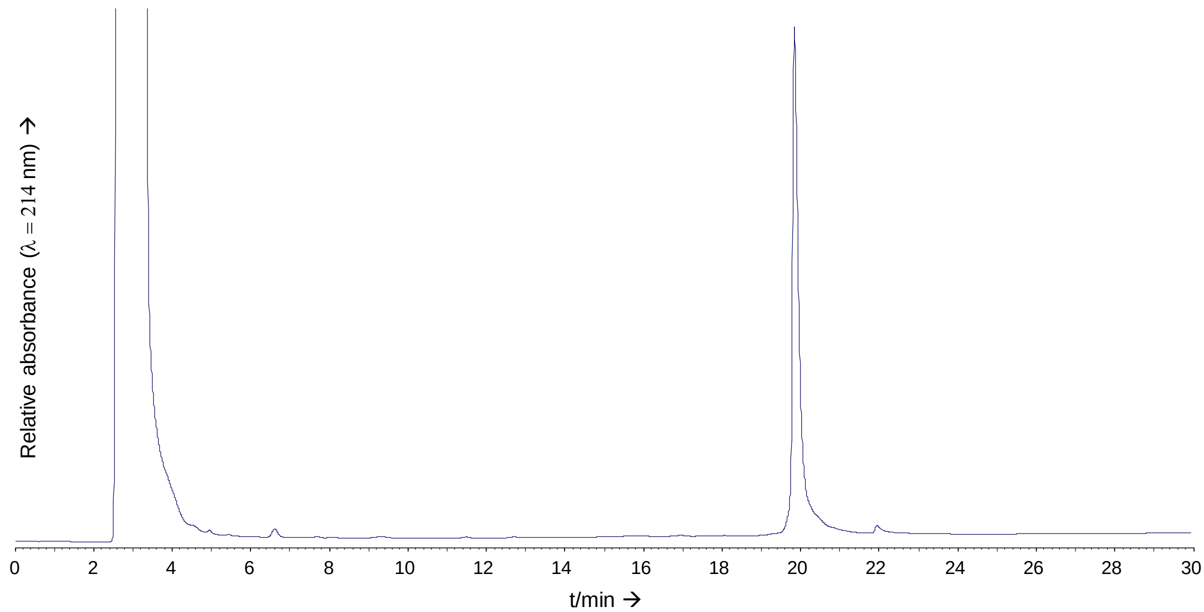

Supplementary figure S61 - Analytical HPLC profile of crude peptide **S23'**. Column: Nucleosil®; gradient: from 10% to 25% B over 30 min.

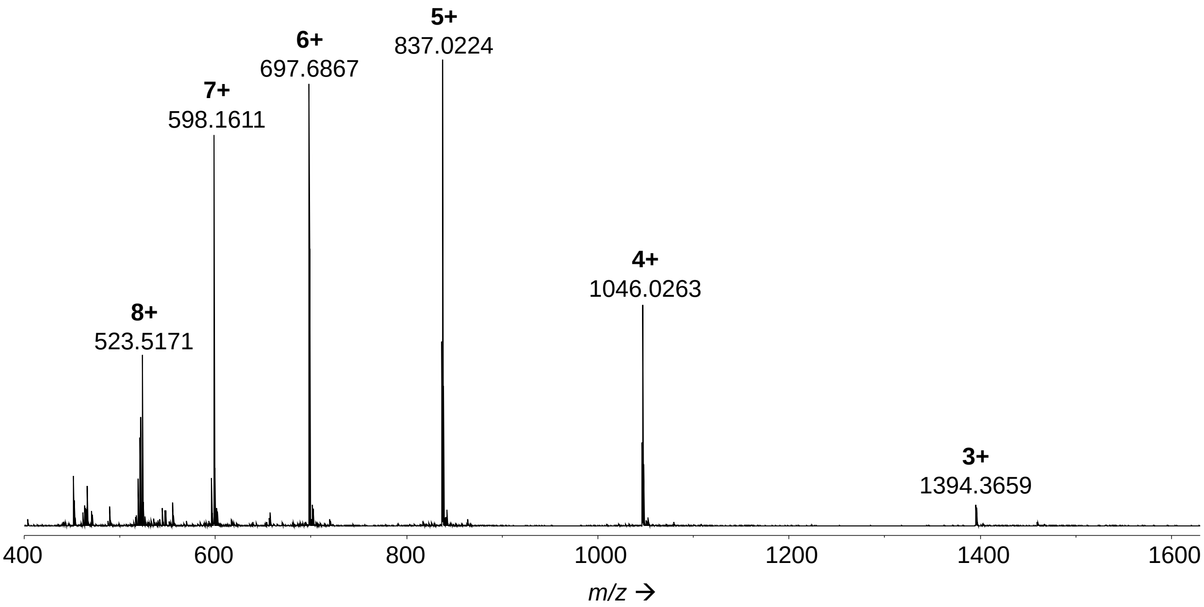

Supplementary figure S62 - ESI-HRMS mass spectrum of peptide **S23'**

H.2 Introduction of a biotin probe on solid supported glycopeptide S21 through CuAAc

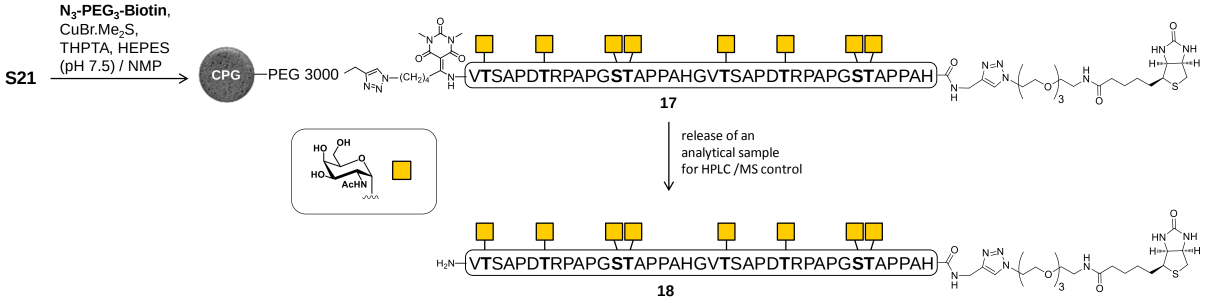

Supplementary scheme S32 – Introduction of biotin probe on solid supported peptide **S21**

Solid supported glycopeptide **S21** (20 nmol) was introduced in a 2 mL microcentrifuge tube and washed with de-ionized water (2 x 500 µL). A solution of N_3-PEG$_3$-Biotin (450 µg, 1 µmol, 50 equiv) dissolved in 100 µL of a 1:1 mixture of NMP and 200 mM HEPES buffer pH 7.5 was then added. To the suspension were subsequently added a 1 M aqueous aminoguanidine solution (3 µL, 150 equiv.), a 1 M aqueous *tert*-butanol solution (3 µL, 150 equiv.). The tube was sealed with a rubber septum and the resulting mixture was further deoxygenated through several successive vacuum (15 mbar) / argon cycles. Then, a mixture containing copper(I) bromide-dimethyl sulfide complex (30 equiv.) and THPTA (40 equiv.) dissolved under argon in 10 µL of deoxygenated NMP was added, the resulting suspension was further deoxygenated and was stirred for 2 h at 37 °C. The resin was then extensively washed with NMP, de-ionized water, then repeatedly treated (3x) with a pH 7 aqueous buffer containing 6 M guanidinium chloride, 0.1 M EDTA and 0.1 M sodium dihydrogenophosphate for a few minutes then drained and finally extensively washed with de-ionized water.

A few beads of the resulting solid supported peptide were cleaved to give crude glycopeptide **18** which was analyzed by RP-HPLC and HRMS.

ESI-HRMS (*m/z*): $[M+H]^+$ calcd for $C_{243}H_{389}N_{64}O_{98}S$: 5803.7144, found: 5803.7232.

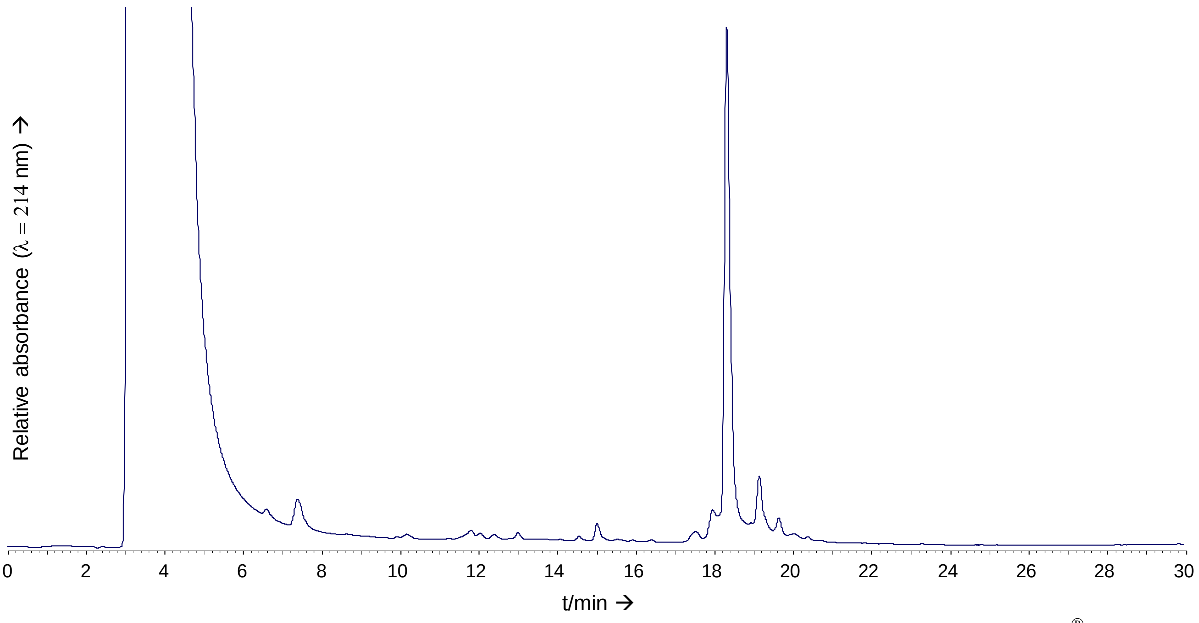

Supplementary figure S63 - Analytical HPLC profile of crude glycopeptide **18.** Column: Nucleosil®; gradient: from 10% to 25% B over 30 min.

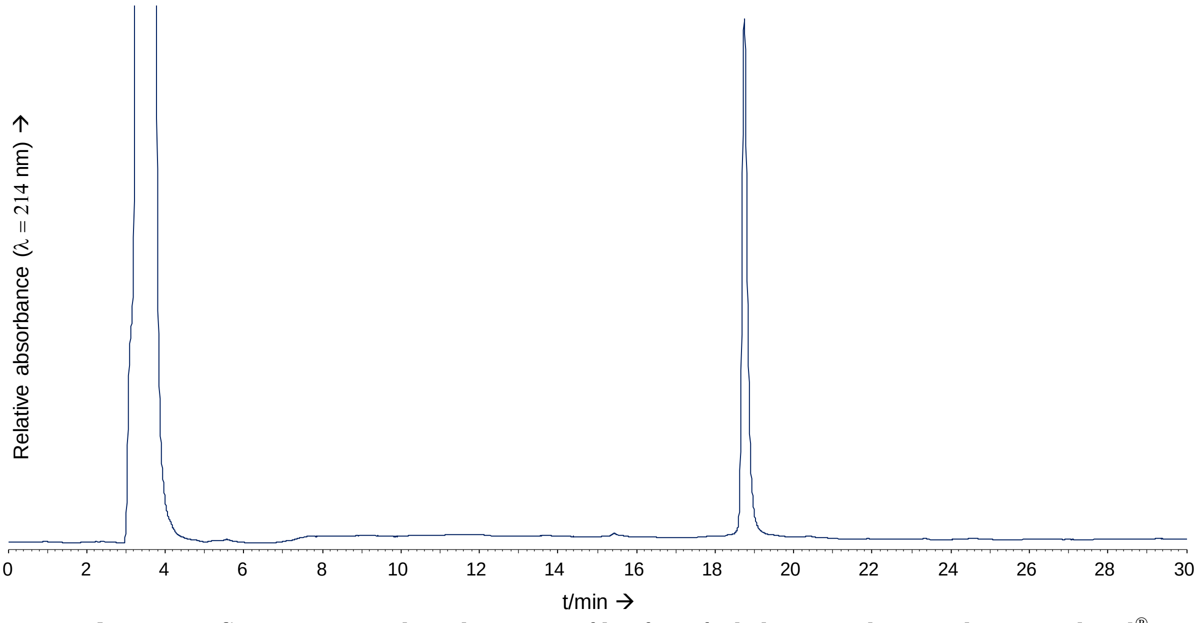

Supplementary figure S64 - Analytical HPLC profile of purified glycopeptide **18.** Column: Nucleosil®; gradient: from 10% to 25% B over 30 min.

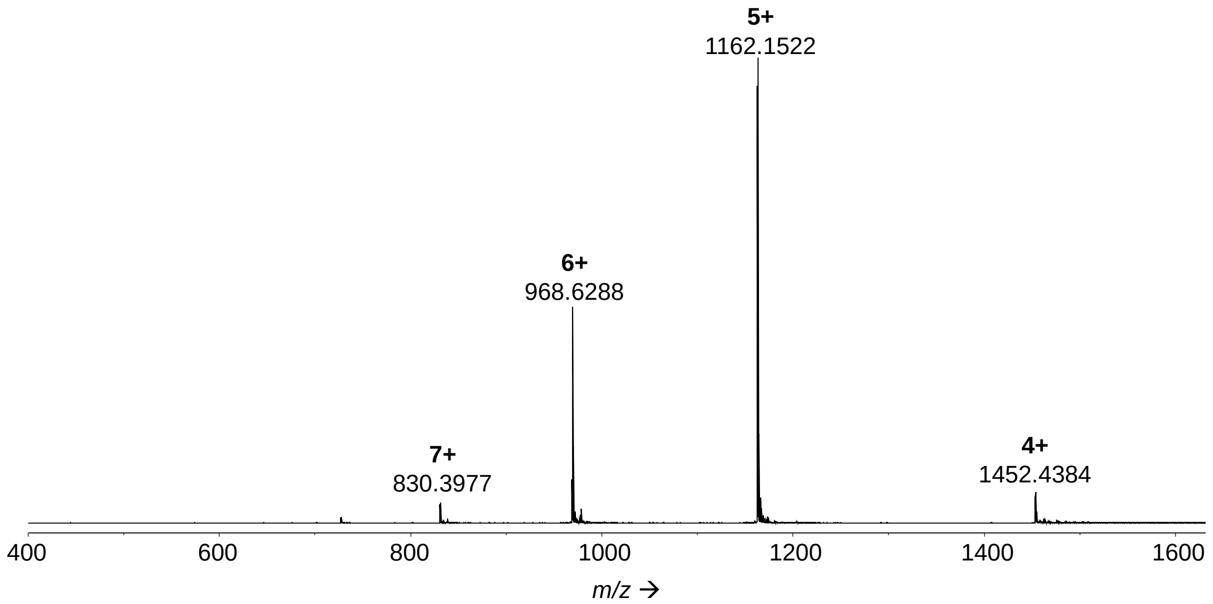

<u>Supplementary figure S65</u> - ESI-HRMS mass spectrum of glycopeptide **18**

I Fluorescence labeling of CPG supported peptides

All the differently substituted beads (**9c**, **10c**, **S23**, **S21** and **17**) were washed with water (2x), PBS containing BSA (2 mg/mL). Substituted beads were subsequently incubated with labelling agent at room temperature, with moderate agitation. At the end of the incubation periods, all beads were washed in PBS, mounted on glass slides in a 1:1 mixture of PBS/mounting medium (Vectashield, Vector Laboratories, Burlingame, CA, USA) and observed with a Zeiss Axiovert 200 M microscope coupled with a Zeiss LSM 510 scanning device (Carl Zeiss Co. Ltd., Jena, Germany). Mouse anti human MUC1 antibodies (clones C595 and CB2, Serotec, Cergy, France) were used together at 0.5 µg/mL in PBS-BSA. After 45 min incubation followed by 3 washes in PBS, a secondary antibody (FITC-conjugated goat anti mouse IgG, Jackson ImmunoResearch, West Grove, PA, USA) diluted 7 µg/mL in PBS-BSA was added to the beads for 45 min. Incubations with the GalNAc-specific *Vica villosa* lectin (VVL) were routinely carried out for 45 min in PBS-BSA with 20 µg/mL biotin-labeled lectin or FITC-conjugated lectin (Vector Laboratories). The beads were washed in PBS and for the biotin-VVL treatment, further incubated with streptavidin Alexa-fluor 568 (Molecular Probes, Eugene, OR, USA) at 4 µg/mL in PBS-BSA for 45 min. Biotin-substituted beads were incubated with streptavidin Alexa-fluor 568 (Molecular Probes) at 4 µg/mL in PBS-BSA for 45 min. Whenever they were doubly labeled, the beads were incubated in the same conditions as above first with the antibodies and then with the couple biotin-VVL / streptavidin-Alexa-fluor 568, or first with FITC-VVL and then streptavidin-Alexa-fluor 568, or with the antibodies and then streptavidin Alexa-fluor 568. For an extensive labeling of the beads, FITC- VVL was used at 2 mg/mL for 18 h, followed by washes in PBS (6x).

			Solid supported peptide				
Entry		Labeling	None **9c**	Muc1 **10c**	Muc1-biotin **S23**	Muc1 (8GalNAc) **S21**	Muc1 (8GalNAc)-biotin **17**
1	A	Anti-MUC Ab + FITC-anti-mouse Ab	**Neg**[1]	**Pos**[3]	**Pos**	**Pos**	**Pos**
2	B	VVL-biotin + streptavidin-Alexa 568	n.d.[2]	**Neg**	n.d.	**Pos**[4]	n.d.
3		A + B	**Neg**	n.d.	n.d.	**Pos**[5]	n.d.
4		FITC-anti –mouse Ab	**Neg**	**Neg**	**Neg**	**Neg**	**Neg**
5	C	VVL-FITC	n.d.	n.d.	n.d.	n.d.	**Pos**
6	D	Streptavidin-Alexa 568	**Neg**	n.d.	**Pos**	**Neg**	n.d.
7		C + D	**Neg**	**Neg**	n.d.	n.d.	**Pos**
8		A + D	n.d.	n.d.	**Pos**	n.d.	n.d.

[1]no fluorescent labeling, [2] not determined, [3] positive FITC labeling, [4] positive Alexa-fluor 568 labeling, [5] positive double labeling.

Supplementary table S9 –Fluorescence labeling of GPC supported peptides

Supplementary figure S66 - Visualization of CPG beads after labeling with fluorescent probes

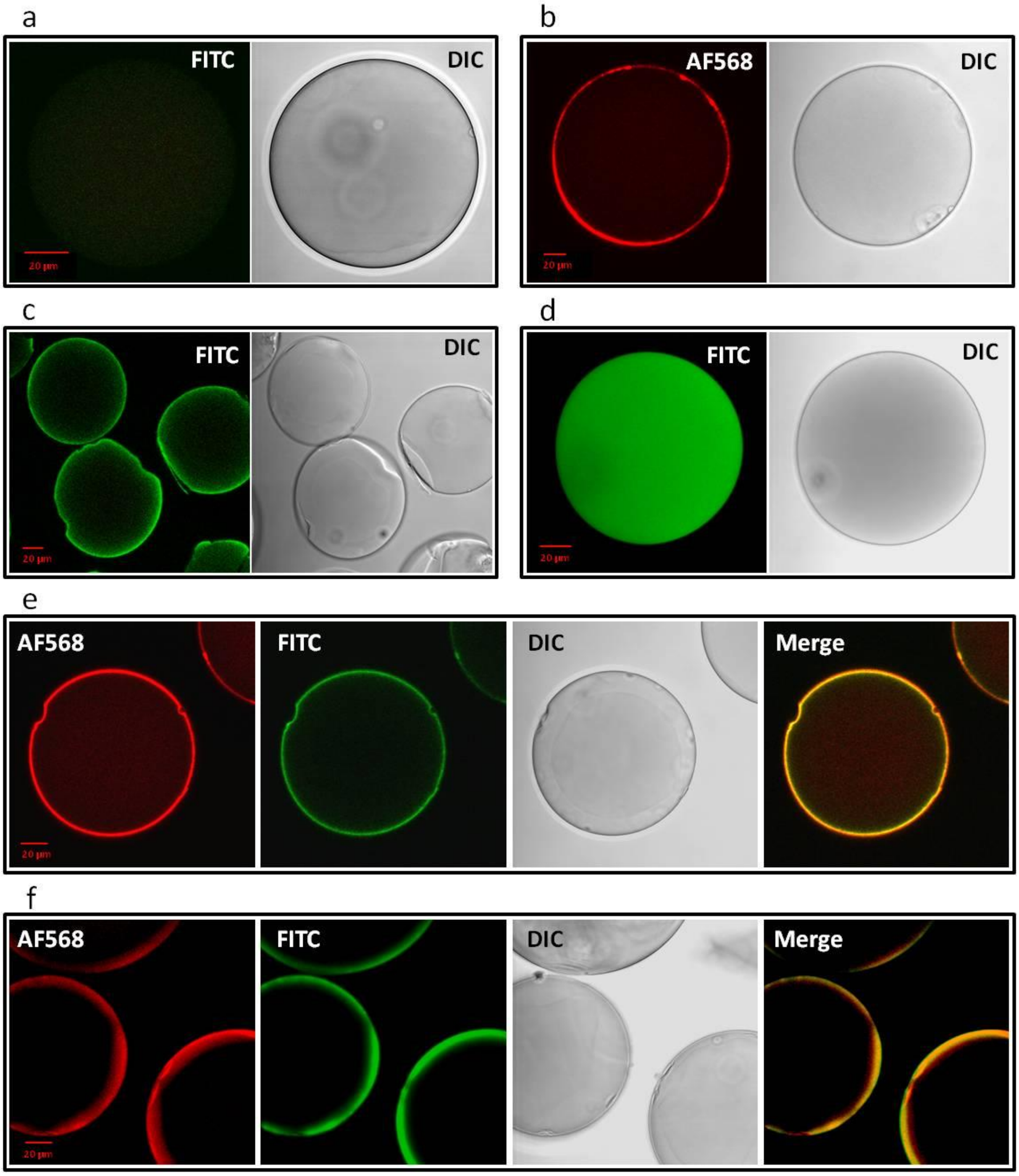

a) Muc1(8GalNAc)-biotin **17** beads after incubation with the FITC–labeled anti-mouse secondary antibody. b) Muc1(8GalNAc) **S21** beads incubated shortly and with low concentration of the biotinylated lectin VVL, and then with streptavidin–Alexa fluor 568. c) Muc1(8GalNAc)-biotin **17** beads after incubation with anti-MUC1 antibodies followed by FITC-labeled secondary antibody. d) Muc1(8GalNAc)-biotin **17** beads after a long incubation with a high concentration of FITC-labeled lectin VVL. e) Muc1-biotin **S23** beads incubated first with streptavidin–Alexa fluor 568 and then with anti–MUC1 antibodies followed by an FITC –labeled secondary antibody. f) Muc1(8GalNAc) **S21** beads incubated first with FITC-VVL and then streptavidin-Alexa-fluor 568. All beads were observed by confocal microscopy. The red bar represents 20 µm; DIC: phase contrast; AF568: Alexa-fluor 568.

J Demonstration that presence of a triazole as amide surrogate does not alter enzymatic glycosylation efficiency

J.1 Synthesis of 20mer Muc1 peptide S24 containing N_3-Dtpp

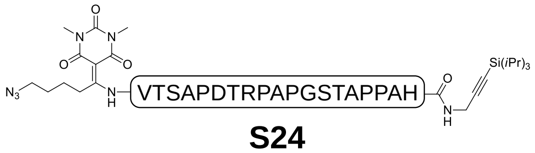

S24

The backbone amide linker 4-(4-formyl-3,5-dimethoxyphenoxy)butyric acid, (34 mg, 125 µmol, 2.5 equiv.) was loaded on Rink amide ChemMatrix® resin (96 mg, 0.52 mmol/g, 50 µmol) using HATU (47 mg, 125 µmol, 2.5 equiv.) and *i*Pr_2NEt (43 µL, 250 µmol, 5 equiv.) in DMF for 2 h at room temperature. The resin was then thoroughly washed with DMF. In case of incomplete reaction (positive Kaiser's test), was repeated once.

3-(Triisopropylsilyl)prop-2-yn-1-amine (53 mg, 250 µmol, 5 equiv.) and $NaBH_3CN$ (16 mg, 250 µmol, 5 equiv.) dissolved in 5 mL of DMF/MeOH/AcOH (7:2:1) were added to the resin (1 equiv., swelled in DMF prior to the reaction), and the resulting suspension was mixed overnight at 50 °C with N_2 bubbling.

The resin was then thoroughly washed with DMF and CH_2Cl_2. Fmoc-His(Trt)-OH (310 mg, 0.5 mmol, 10 equiv.) was coupled onto the resulting resin with HATU (190 mg, 0.5 mmol, 10 equiv.) and *i*Pr_2NEt (175 µL, 1 mmol, 20 equiv.) in DMF overnight at room temperature.

The elongation of the peptide was performed by standard automated solid phase synthesis (p S4) up to ^{2}Thr. Prolines 4 and 5 were coupled twice following the automated program for double couplings. A 85% elongation yield was obtained, determined by the titration of the first (BAL) and last (^{2}Thr) Fmoc group deprotection (UV, 301 nm).

At this point, the resin was divided in two equal portions, for the production of both **S24** and **S25**.

Compound **S6** (2 equiv., dissolved in DMF), HATU (2 equiv.) and *i*Pr_2NEt (3 equiv.) were added to the peptide resin (25 µmol, 1 equiv., swelled in DMF prior to the reaction), and the resulting suspension was stirred overnight at room temperature. The resin was thoroughly washed with DMF. The resin was further washed with CH_2Cl_2, and treated with TFA/*i*Pr_3SiH/1,3 dimethoxybenzene (90:5:5) for 2 h. Peptide **S24** was precipitated by dilution with ice-cold diethyl ether, recovered by centrifugation, washed 3 times with diethyl ether, dried under reduced pressure and lyophilized to yield crude peptide **S24** as a white fluffy powder (42.7 mg, 17 µmol, 68%* isolated yield based on the original resin loading).

This peptide was used without any further purification.

*: estimated considering a MW = 2514 g/mol taking into account 4 trifluoroacetate counter-anions, as predicted from the sequence: 1Arg, 1His = +2 overall charge. Water content is not taken into account.

ESI-HRMS (*m/z*): $[M+H]^+$ calcd for $C_{101}H_{161}N_{30}O_{29}Si$: 2286.1815, found: 2286.1843

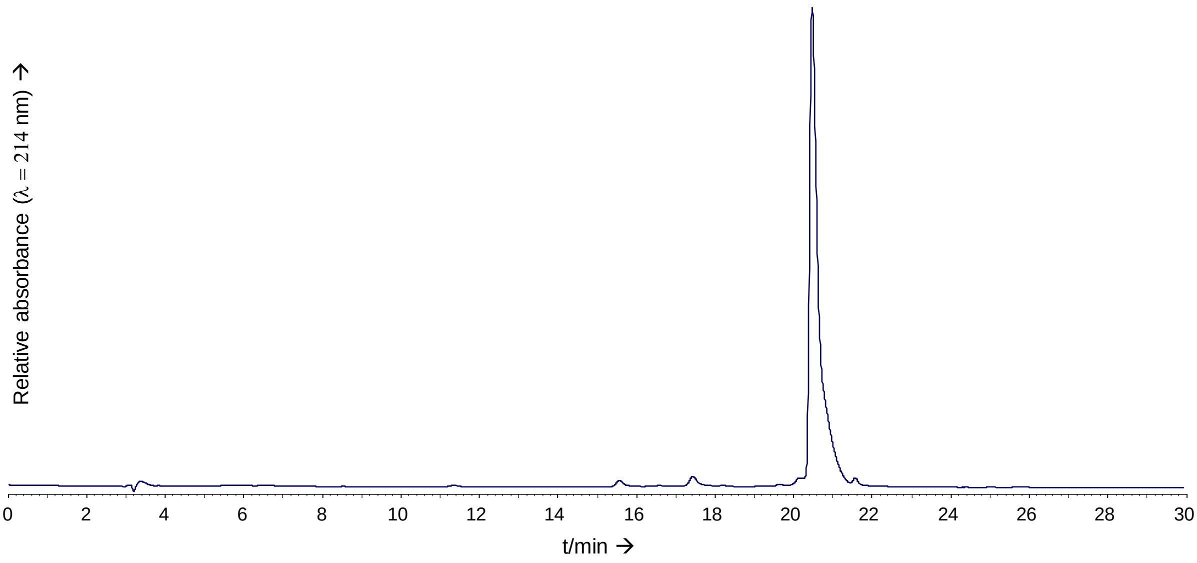

<u>Supplementary figure S67</u> - Analytical HPLC profile of crude peptide **S24**. Column: Nucleosil®; gradient: from 30% to 60% B over 30 min.

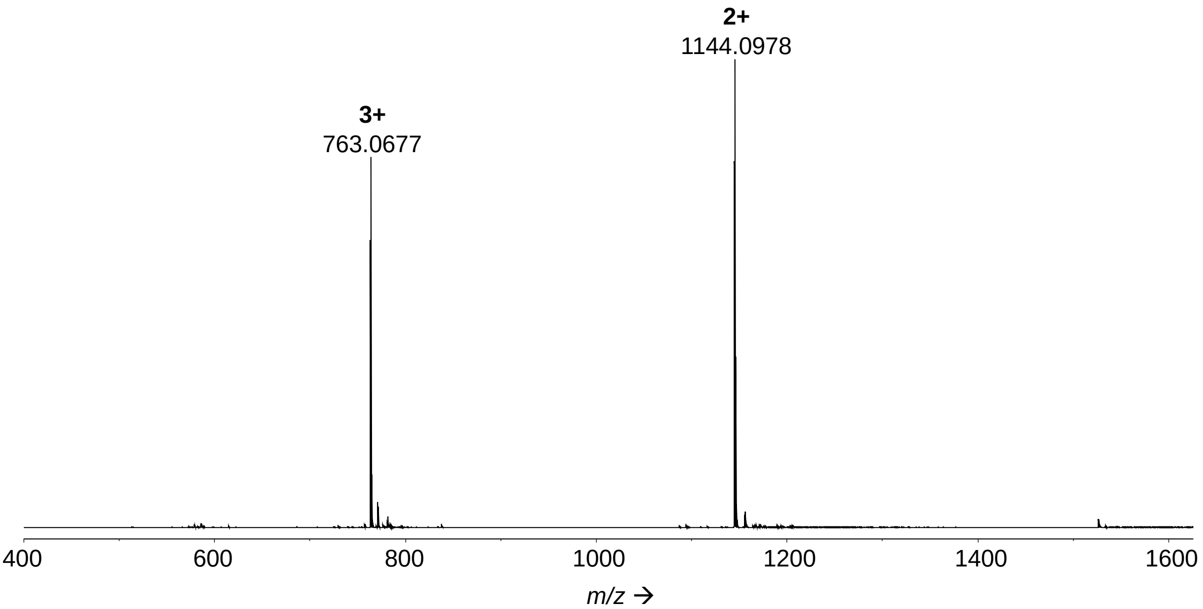

<u>Supplementary figure S68</u> - ESI-HRMS mass spectrum of crude peptide **S24**

J.2 Synthesis of 20mer Muc1 azido peptide S25

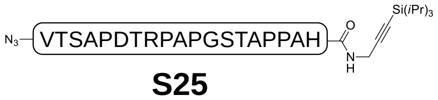

S25

The solid phase elongation of **S25** up to [2]Thr is described in the synthesis of **S24** (pS82). N_3Val-OH (7 mg, 50 µmol, 2 equiv., dissolved in DMF), HATU (19 mg, 50 µmol, 2 equiv.) and *i*Pr_2NEt (13 µL, 75 µmol, 3 equiv.,) were dissolved in DMF. The mixture was then added to the peptide resin (25 µmol, 1 equiv., swelled in DMF prior to the reaction), and the resulting suspension was stirred by syringe rotation for 2 h at room temperature. The resin was then thoroughly washed with DMF. The resin was further washed with CH_2Cl_2, and treated with TFA/*i*Pr_3SiH/1,3 dimethoxybenzene (90:5:5) for 2 h. Peptide **S25** was precipitated by dilution with ice-cold diethyl ether, recovered by centrifugation, washed 3 times with diethyl ether, dried under reduced pressure and lyophilized to yield crude peptide **S25** as a white fluffy powder (39 mg, 17 µmol, 69%* isolated yield based on the original resin loading).

This peptide was used without any further purification.

*: estimated considering a MW = 2277 g/mol taking into account 4 trifluoroacetate counter-anions, as predicted from the sequence: 1Arg, 1His = +2 overall charge. Water content is not taken into account.

ESI-HRMS (*m/z*): $[M+H]^+$ calcd for $C_{90}H_{146}N_{27}O_{26}Si$: 2049.0702, found: 2049.0716

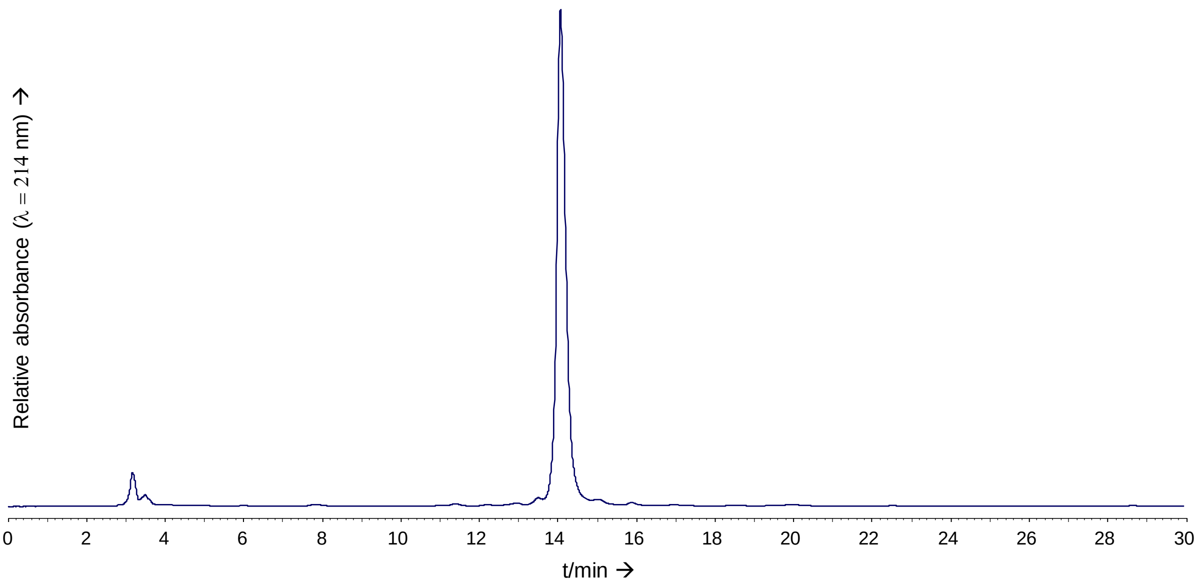

Supplementary figure S69 - Analytical HPLC profile of crude peptide **S25**. Column: Nucleosil®; gradient: from 30% to 60% B over 30 min.

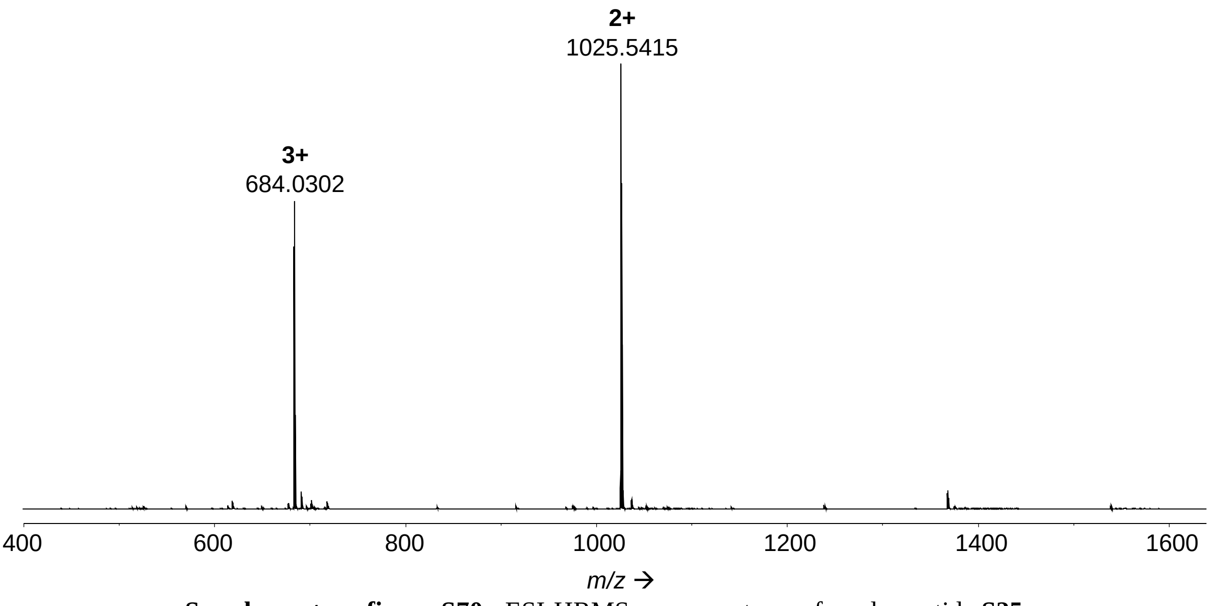

Supplementary figure S70 - ESI-HRMS mass spectrum of crude peptide **S25**

J.3 Grafting of peptide S24 on alkyne functionalized solid support 9c

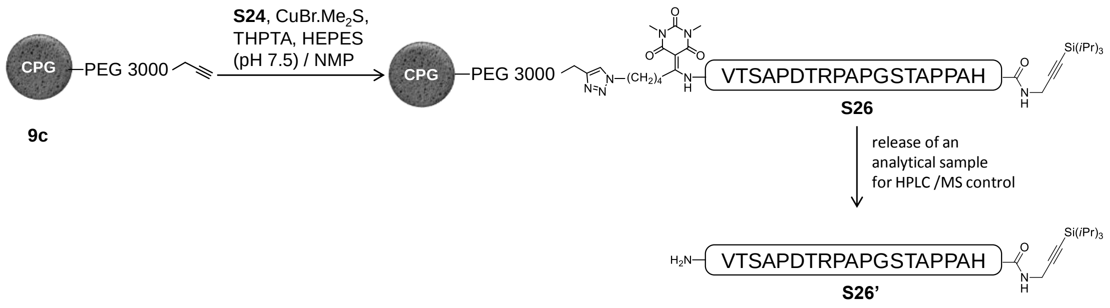

Supplementary scheme S33 - Grafting of peptide **S24** on alkyne functionalized solid support **9c**

Azido peptide **S24** (2.5 mg, 1 µmol) was grafted on alkyne resin **9c** (100 mg) following the typical procedure (p S50).

A few beads of the resulting solid supported peptide were cleaved using the general procedure (p S53) to give crude peptide **S26'** which was analyzed by RP-HPLC and HRMS.

ESI-HRMS (*m/z*): $[M+H]^+$ calcd for $C_{90}H_{148}N_{25}O_{26}Si$: 2023.0797, found: 2023.0794

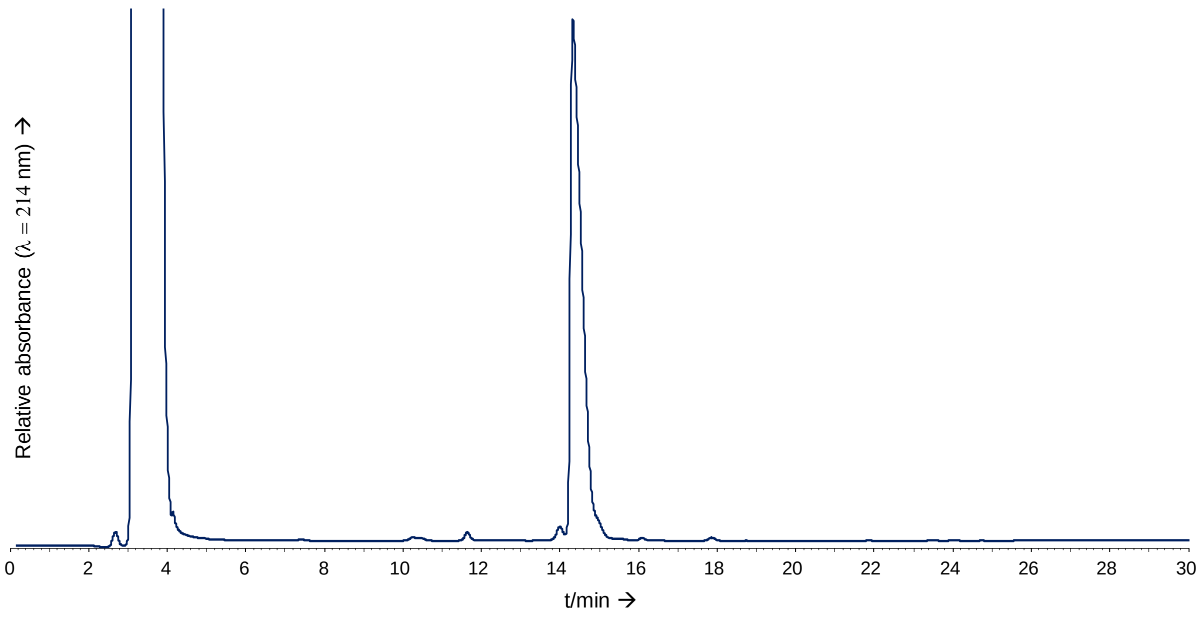

<u>Supplementary figure S71</u> - Analytical HPLC profile of crude peptide **S26'**. Column: Nucleosil®; gradient: from 30% to 60% B over 30 min.

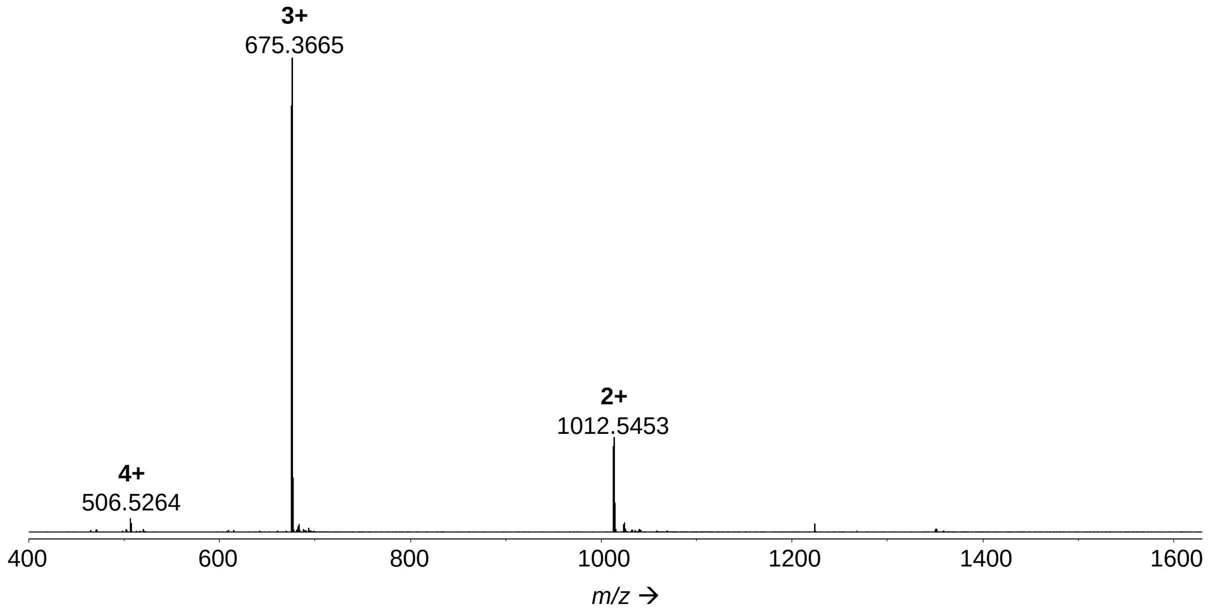

<u>Supplementary figure S72</u> - ESI-HRMS mass spectrum of crude peptide **S26'**

J.4 Solid-supported deprotection of the TIPS group of S26

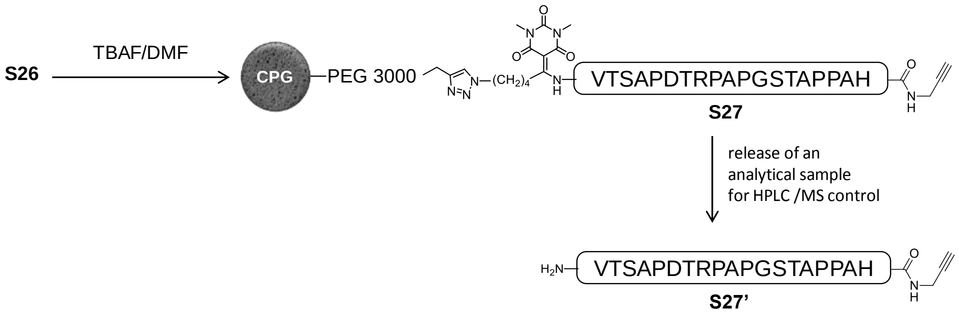

Supplementary scheme S34 - Solid-supported deprotection of the TIPS group of **S26**

The solid supported peptide **S26** was introduced in a syringe fitted with a polypropylene frit and a teflon stopcock and was washed with anhydrous DMF (4x). A mixture of TBAF (100 equiv.) dissolved in anhydrous DMF (final concentration 100 mM) was added and the reaction mixture was stirred by syringe rotation for 15 min at room temperature. This protocol was repeated once and the resin was then extensively washed with DMF, a 0.1% aqueous TFA solution and finally de-ionized water to give the solid supported peptide **S27**.
A few beads of the resulting solid supported peptide were cleaved using the general procedure (p S53) to give crude peptide **S27’** which was analyzed by RP-HPLC and HRMS.

ESI-HRMS (*m/z*): $[M+H]^+$ calcd for $C_{81}H_{128}N_{25}O_{26}$: 1866.9462, found: 1866.9468.

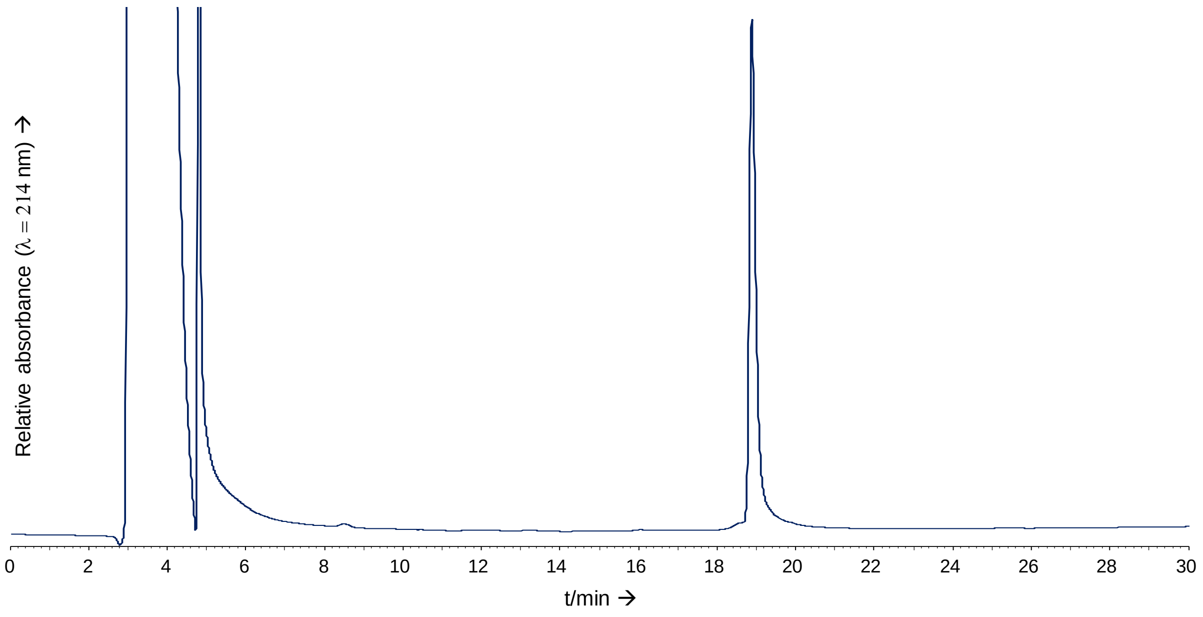

<u>Supplementary figure S73</u> - Analytical HPLC profile of crude peptide **S27'**. Column: Nucleosil®; gradient: from 10% to 25% B over 30 min.

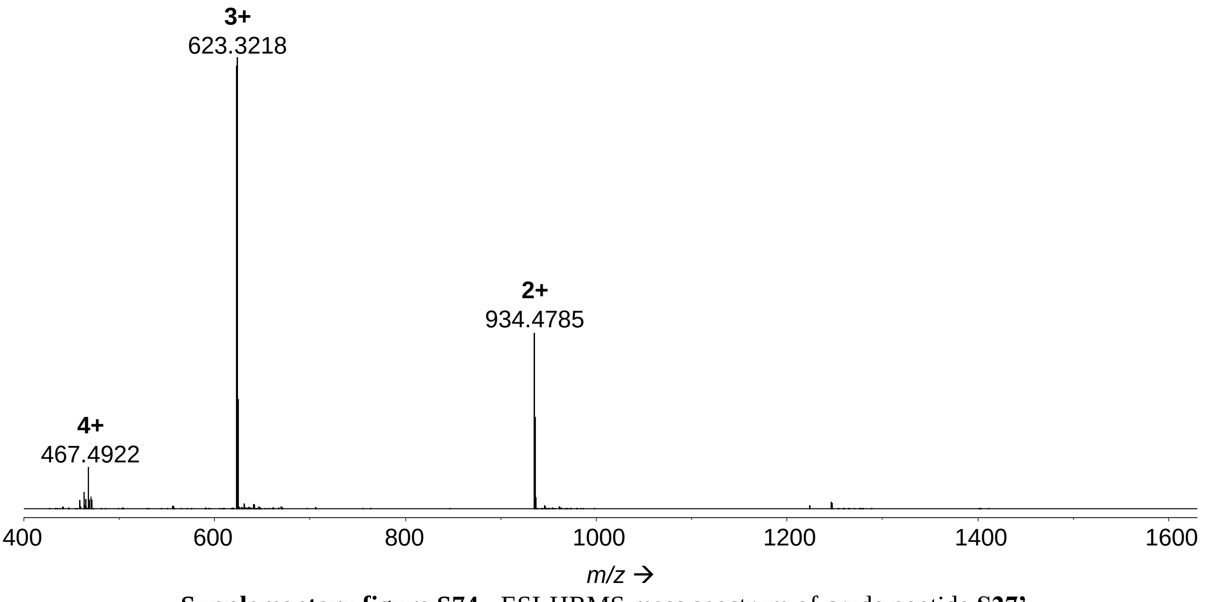

<u>Supplementary figure S74</u> - ESI-HRMS mass spectrum of crude peptide **S27'**

J.5 Introduction of azido peptide S25 on solid supported peptide S27 through peptidomimetic triazole ligations

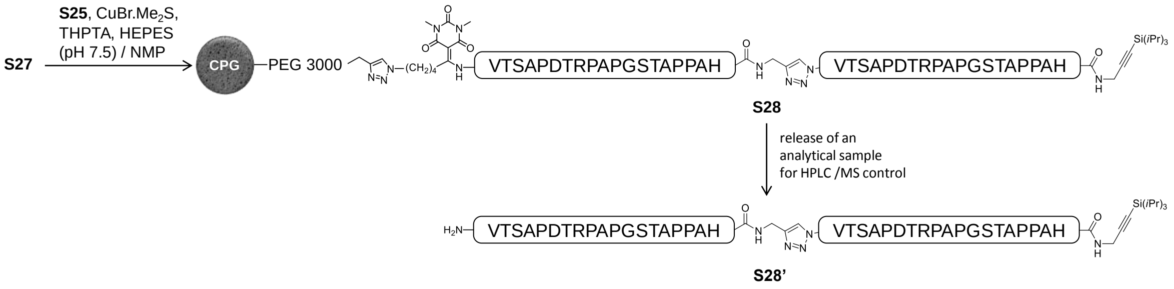

Supplementary scheme S35 - Grafting of peptide **S25** on alkyne peptide resin **S27**

Azido peptide **S25** (3.4 mg, 1.5 µmol, 1.5 equiv.) was grafted on peptide resin **S27** (1 µmol) following the typical procedure (p S50)

A few beads of the resulting solid supported peptide were cleaved using the general procedure (p S53) to give crude peptide **S28'** which was analyzed by RP-HPLC and HRMS.

ESI-HRMS (*m/z*): $[M+H]^+$ calcd for $C_{171}H_{273}N_{52}O_{52}Si$: 3915.0086, found: 3915.0055

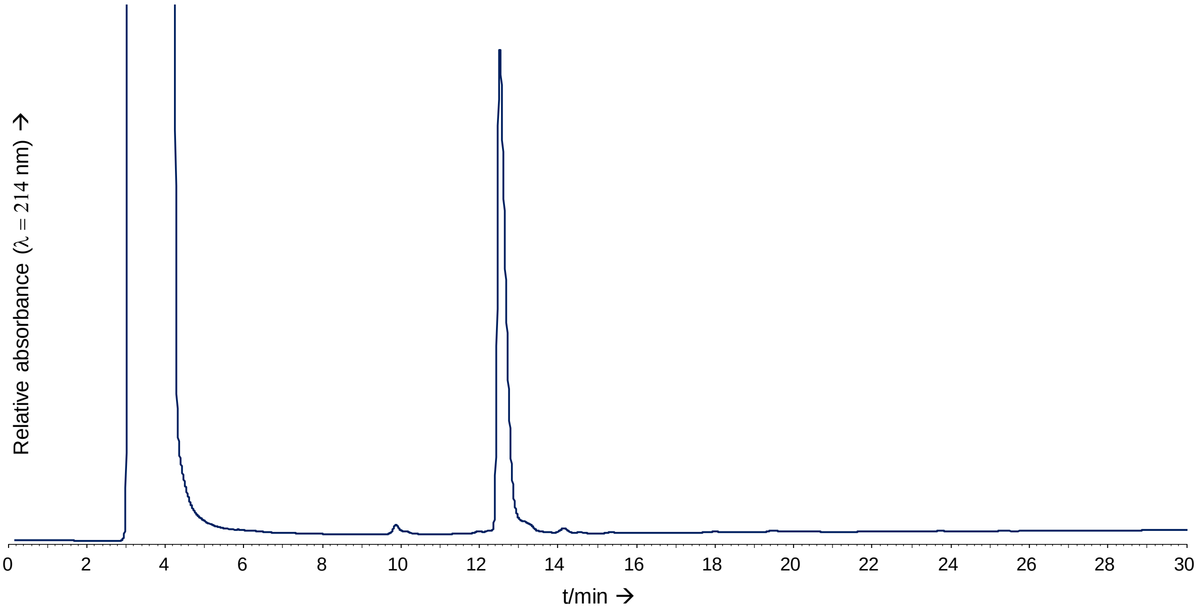

Supplementary figure S75 - Analytical HPLC profile of crude peptide **S28'**. Column: Nucleosil®; gradient: from 30% to 60% B over 30 min.

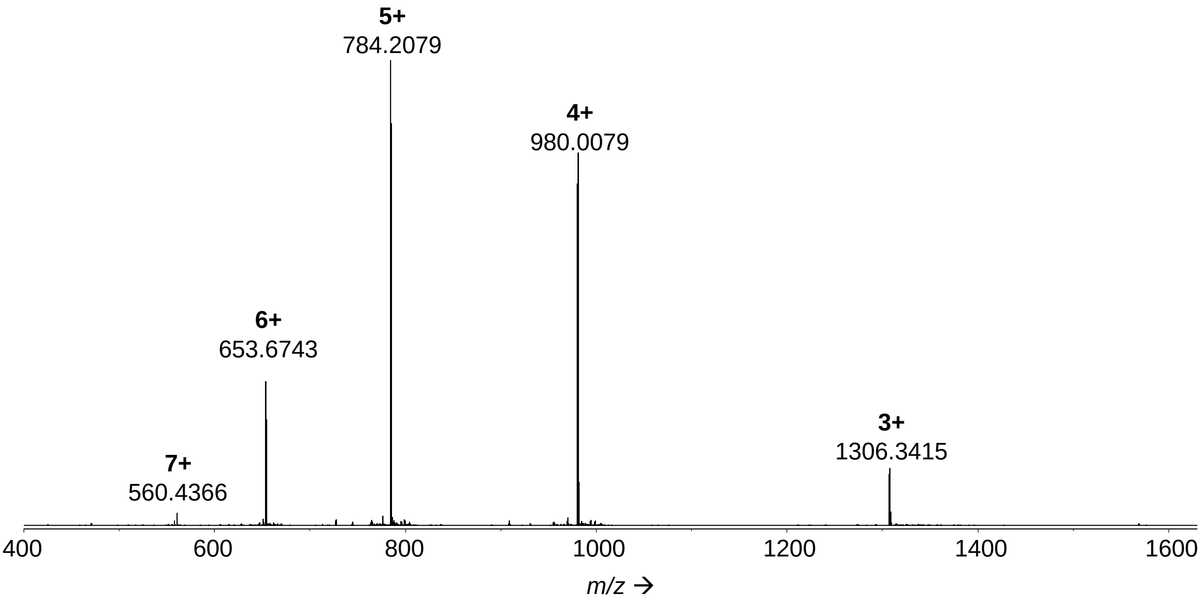

Supplementary figure S76 - ESI-HRMS mass spectrum of peptide crude **S28'**

J.6 Solid-supported deprotection of the TIPS group of S28

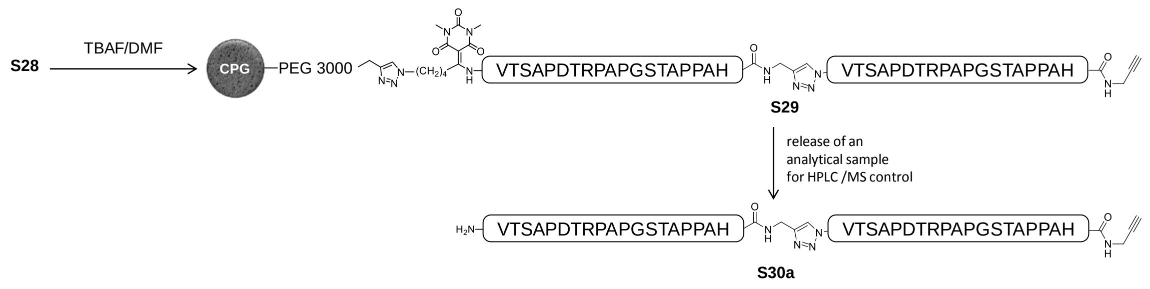

Supplementary scheme S36 - Solid-supported deprotection of the TIPS group of **S28**

The solid supported peptide **S28** was introduced in a syringe fitted with a polypropylene frit and a teflon stopcock and was washed with anhydrous DMF (4x). A mixture of TBAF (100 equiv.) dissolved in anhydrous DMF (final concentration 100 mM) was added and the reaction mixture was stirred by syringe rotation for 15 min at room temperature. This protocol was repeated once and the resin was then extensively washed with DMF, a 0.1% aqueous TFA solution and finally de-ionized water to give the solid supported peptide **S29**.
A few beads of the resulting solid supported peptide were cleaved using the general procedure (p S53) to give crude peptide **S30a** which was analyzed by RP-HPLC and HRMS.

ESI-HRMS (*m/z*): $[M+H]^+$ calcd for $C_{162}H_{253}N_{52}O_{52}$: 3758.8752, found: 3758.8737.

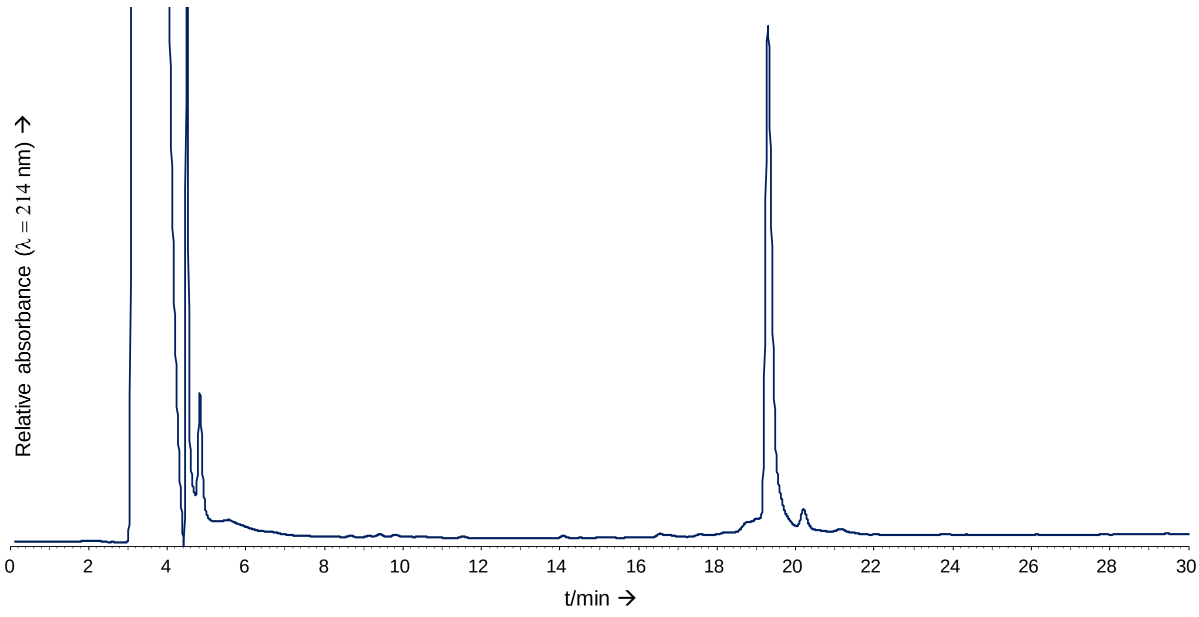

Supplementary figure S77 - Analytical HPLC profile of crude peptide **S30a**. Column: Nucleosil®; gradient: from 10% to 25% B over 30 min.

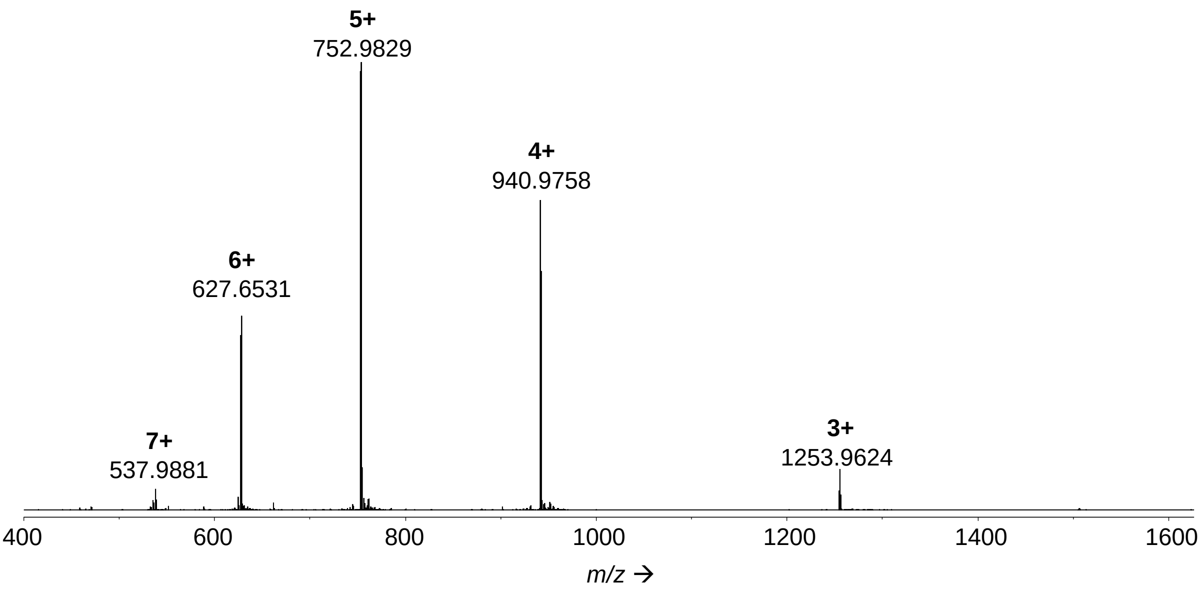

Supplementary figure S78 - ESI-HRMS mass spectrum of peptide **S30a**

J.7 Solid supported enzymatic glycosylation of S29

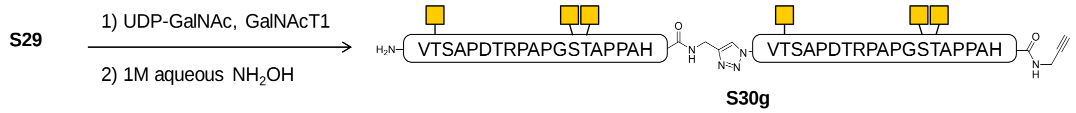

<u>**Supplementary scheme S37**</u> - Enzymatic glycosylation and release of solid supported peptide **S29**

Solid supported glycopeptide **S29** (20 nmol) was glycosylated under rigorously identical conditions as used for peptide **10c** not containing any triazole as amide bond surrogate. It was introduced in a 2 mL microcentrifuge tube and washed with de-ionized water (2 x 500 µL). To the solid was added 100 µL of an aqueous solution containing GalNAc-T1 (6 mU), MES (50 mM, pH 6.5), $MnCl_2$ (15 mM), BSA (100 ng), DTT (1 mM) and UDP-GalNAc (2 equiv. per glycosylation sites). The resulting suspension was mixed at 37 °C for 48 h under moderate agitation. Beads were then washed with de-ionized water (2 x 500 µL), in order to remove the UDP formed during the transfer reaction since it inhibits GalNAc-T1 activity. Solid supported glycopeptide was then incubated under the same conditions as above for another 48 h and the resin was washed with de-ionized water.

The resulting solid supported peptide was released under typical procedure (p S53) to give crude mixture containing glycopeptides **S30g** and **S30f** which were analyzed by RP-HPLC and HRMS.

No difference in glycosylation rate could be observed between 10c and S29, demonstrating that presence of a triazole did not alter the enzymatic glycosylation efficiency.

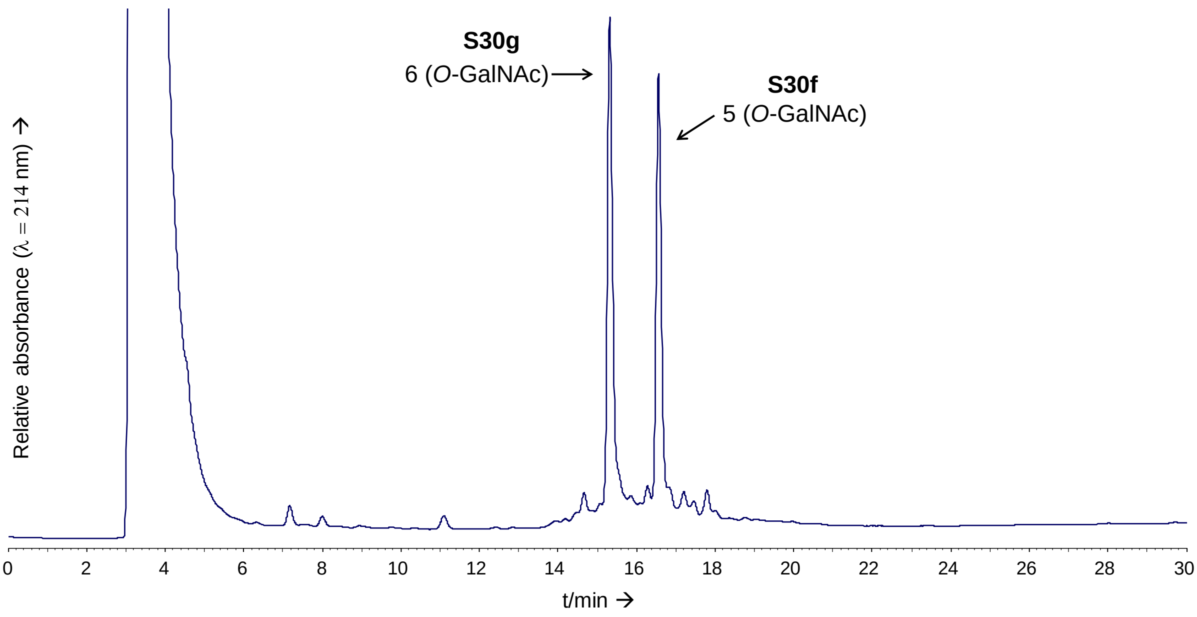

Supplementary figure S79 - Analytical HPLC profile of crude mixture containing glycopeptide **S30g**. Column: Nucleosil®; gradient: from 10% to 25% B over 30 min.

- ESI-HRMS analysis of crude mixture containing glycopeptide **S30g**

Glycopeptide	Number of GalNAc	Formula	$[M+H]^+$ calcd	$[M+H]^+$ found
**S30g**	6	$C_{210}H_{331}N_{58}O_{82}$	4977.3514	4977.3504
**S30f**	5	$C_{202}H_{318}N_{57}O_{77}$	4774.2720	4774.2714

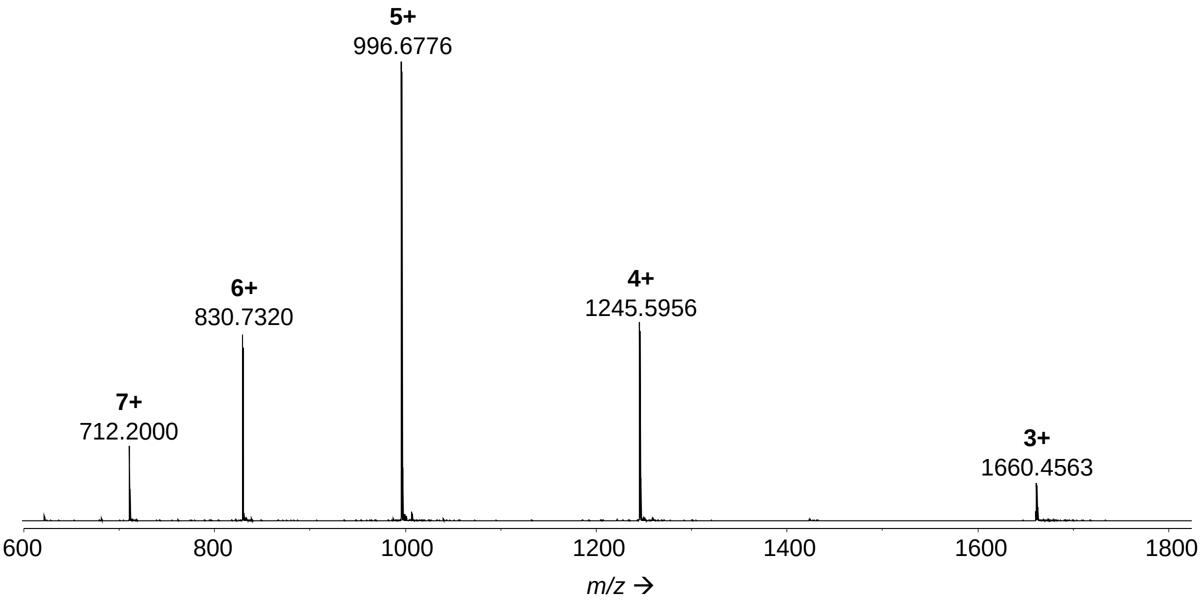

Supplementary figure S80 - ESI-HRMS mass spectrum of peptide **S30g**

- Comparison of glycosylation of **S29** (A) and **10c** (B) under identical condition

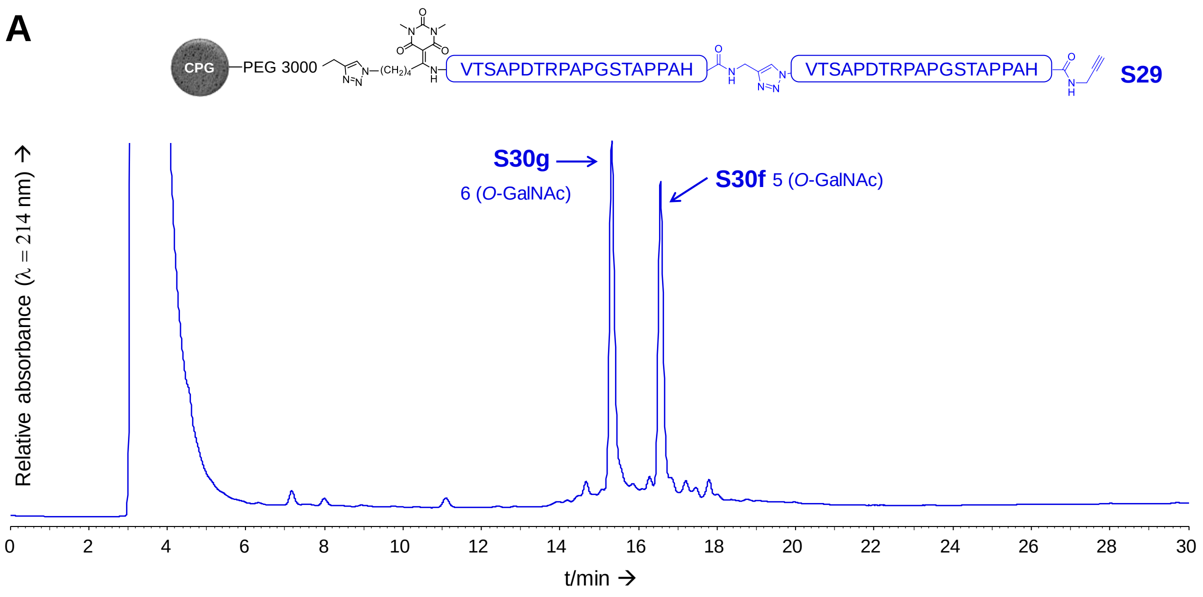

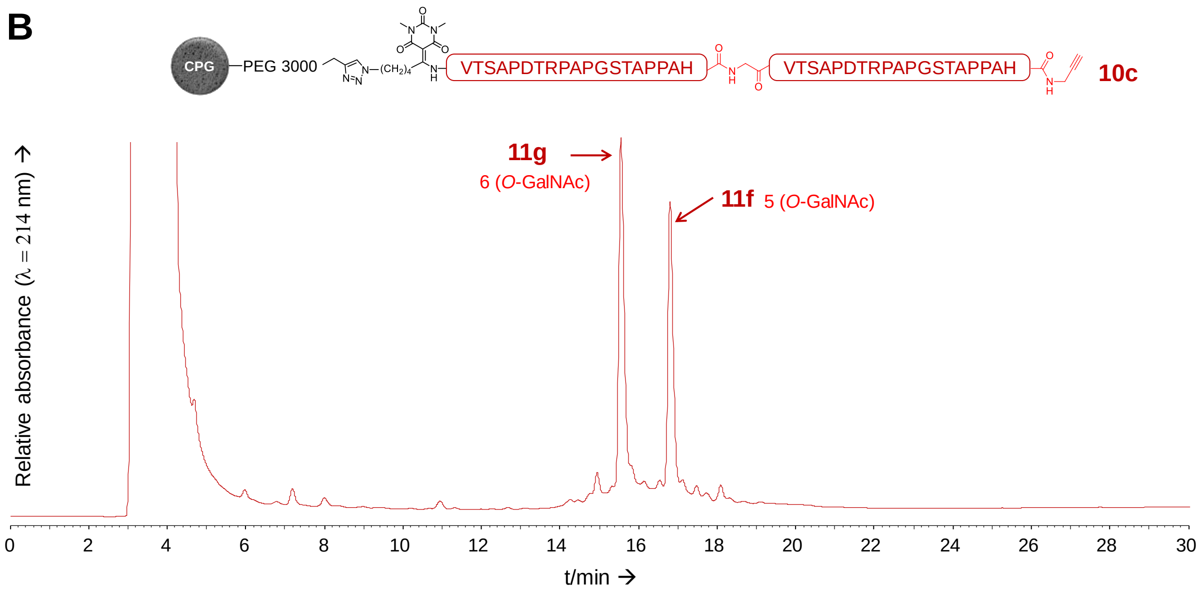

K Successive solid phase peptidomimetic triazole ligations

K.1 Synthesis of peptide azido alkyne Muc1 fragment 19

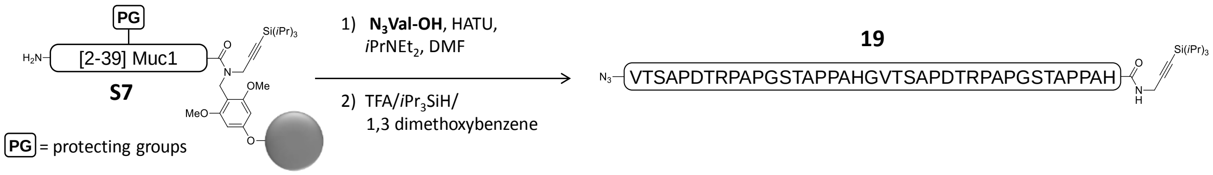

Supplementary scheme S38 - Solid-phase synthesis of peptide **19**

N_3Val-OH (7 mg, 50 µmol, 2 equiv., dissolved in DMF), HATU (19 mg, 50 µmol, 2 equiv.) and *i*Pr_2NEt (13 µL, 75 µmol, 3 equiv.,) were dissolved in DMF. The mixture was then added to the peptide resin **S7** (25 µmol, 1 equiv., swelled in DMF prior to the reaction), and the resulting suspension was stirred by syringe rotation for 2 h at room temperature. The resin was then thoroughly washed with DMF. The resin was further washed with CH_2Cl_2, and treated with TFA/*i*Pr_3SiH/1,3 dimethoxybenzene (90:5:5) for 2 h. Peptide **19** was precipitated by dilution with ice-cold diethyl ether, recovered by centrifugation, washed 3 times with diethyl ether, dried under reduced pressure and lyophilized to yield crude peptide **19** as a white fluffy powder (50.3 mg, 11.5 µmol, 46%* isolated yield based on the original resin loading).

This peptide was used without any further purification.

*: estimated considering a MW = 4375 g/mol taking into account 4 trifluoroacetate counter-anions, as predicted from the sequence: 2Arg, 2His = +4 overall charge. Water content is not taken into account.

ESI-HRMS (*m/z*): $[M+H]^+$ calcd for $C_{170}H_{271}N_{52}O_{53}Si$: 3916.9878, found: 3916.9771

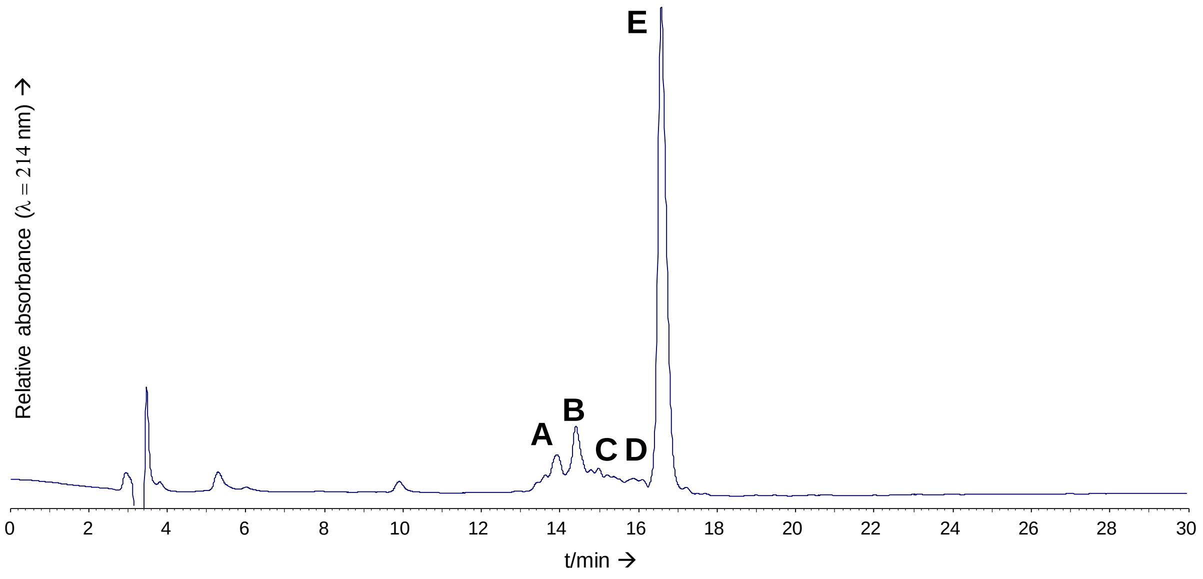

<u>Supplementary figure S81</u> - Analytical HPLC profile of crude peptide **19**. Column: Nucleosil®; gradient: from 30% to 60% B over 30 min.

Peak	Found *m/z*	Attributed to	Calculated $[M+H]^+$
A	3574.8192	Ac-[5-39]	3574.8227 ($C_{157}H_{249}N_{46}O_{48}Si$)
	3362.7356	Ac-[7-39]	3362.7430 ($C_{148}H_{237}N_{44}O_{44}Si$)
B	3732.8789	Ac-[3-39]	3732.8918 ($C_{163}H_{259}N_{48}O_{51}Si$)
	3261.6876	Ac-[8-39]	3261.6953 ($C_{144}H_{230}N_{43}O_{42}Si$)
	3645.8594	Ac-[4-39]	3645.8598 ($C_{160}H_{254}N_{47}O_{49}Si$)
C	2840.4448	Ac-[12-39]	2840.4516 ($C_{125}H_{199}N_{36}O_{38}Si$)
	2783.4244	Ac-[13-39]	2783.4301 ($C_{123}H_{196}N_{35}O_{37}Si$)
	2937.4944	Ac-[11-39]	2937.5043 ($C_{130}H_{206}N_{37}O_{39}Si$)
	3105.5868	Ac-[9-39]	3105.5942 ($C_{138}H_{218}N_{39}O_{41}Si$)
	2696.3931	Ac-[14-39]	2696.3981 ($C_{120}H_{191}N_{34}O_{35}Si$)
	3008.5329	Ac-[10-39]	3008.5414 ($C_{133}H_{211}N_{38}O_{40}Si$)
D	1966.0157	Ac-[22-39]	1966.0218 ($C_{87}H_{141}N_{24}O_{26}Si$)
	1864.9684	Ac-[23-39]	1864.9742 ($C_{83}H_{134}N_{23}O_{24}Si$)
	2122.1073	Ac-[20-39]	2122.1117 ($C_{94}H_{153}N_{26}O_{28}Si$)
	1494.8202	Ac-[27-39]	1494.8253 ($C_{68}H_{112}N_{19}O_{17}Si$)
	1609.8478	Ac-[26-39]	1609.8523 ($C_{72}H_{117}N_{20}O_{20}Si$)
	1393.7730	Ac-[28-39]	1393.7776 ($C_{64}H_{105}N_{18}O_{15}Si$)
	1777.9367	Ac-[24-39]	1777.9421 ($C_{80}H_{129}N_{22}O_{22}Si$)
	1706.8987	Ac-[25-39]	1706.9050 ($C_{77}H_{124}N_{21}O_{21}Si$)
E	3916.9771	**19**	3916.9878 ($C_{170}H_{271}N_{52}O_{53}Si$)
	1237.6726	Ac-[29-39]	1237.6765 ($C_{58}H_{93}N_{14}O_{14}Si$)
	1140.6208	Ac-[30-39]	1140.6238 ($C_{53}H_{86}N_{13}O_{13}Si$)
	915.5092	Ac-[33-39]	915.5124 ($C_{43}H_{71}N_{10}O_{10}Si$)

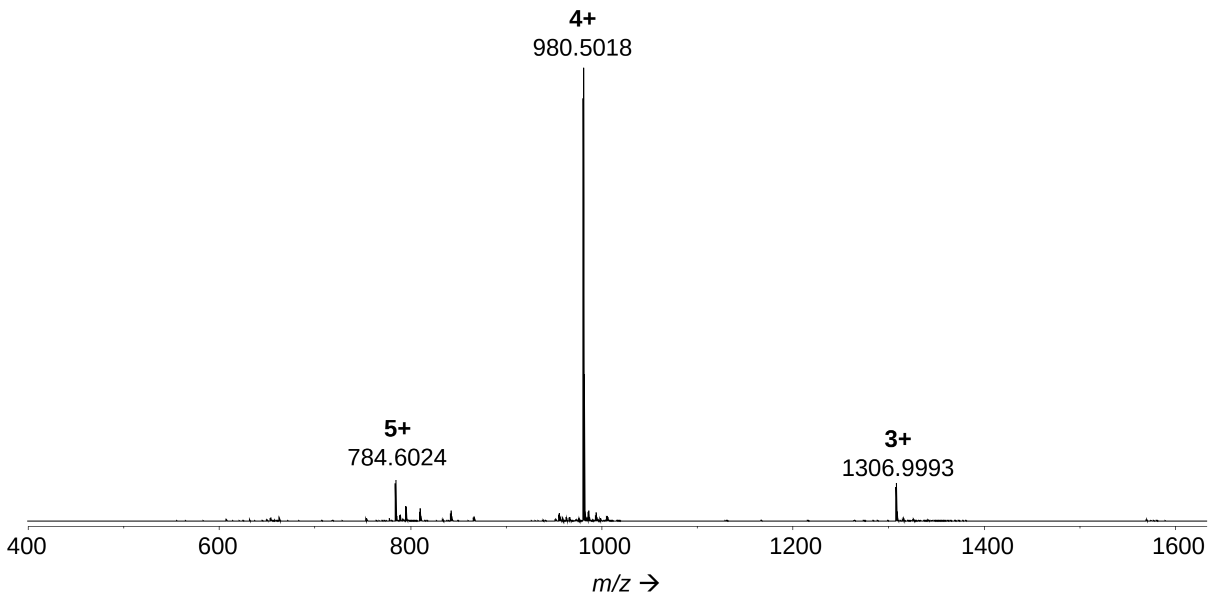

<u>Supplementary figure S82</u> - ESI-HRMS mass spectrum of peptide **19**

K.2 Typical procedures for multiple successive solid phase peptidomimetic triazole ligations

i) Typical procedure for solid-supported peptidomimetic triazole ligations:

Solid supported alkyne peptide (1 µmol) was introduced in a 2 mL microcentrifuge tube and washed with de-ionized water (2 x 500 µL). A solution of crude **19** (6.6 mg, 1.5 µmol, 1.5 equiv) dissolved in 100 µL of a 1:1 mixture of NMP and 200 mM HEPES buffer pH 7.5 was then added. To the suspension were subsequently added a 1 M aqueous aminoguanidine solution (3 µL, 30 equiv.) and a 1 M aqueous *tert*-butanol solution (3 µL, 30 equiv.). The tube was sealed with a rubber septum and the resulting mixture was further deoxygenated through several successive vacuum (15 mbar) / argon cycles. Then, a mixture containing copper(I) bromide-dimethyl sulfide complex (30 equiv.) and THPTA (40 equiv.) dissolved under argon in 10 µL of deoxygenated NMP was added, the resulting suspension was further deoxygenated and was stirred for 5 h at 37 °C. The resin was then extensively washed with NMP, de-ionized water, then repeatedly treated (3x) with a pH 7 aqueous buffer containing 6 M guanidinium chloride, 0.1 M EDTA and 0.1 M sodium dihydrogenophosphate for a few minutes then drained and finally extensively washed with de-ionized water.

ii) Typical procedure for solid-supported deprotection of the TIPS group:

The solid supported TIPS-alkyne peptide was introduced in a syringe fitted with a polypropylene frit and a teflon stopcock and was washed with anhydrous DMF (4x). A mixture of TBAF (100 equiv.) and phenol[15] (110 equiv.) dissolved in anhydrous DMF (final TBAF concentration: 100 mM) was added and the reaction mixture was stirred by syringe rotation for 2 h at room temperature. This protocol was repeated once and the resin was then extensively washed with DMF, a 0.1% aqueous TFA solution and finally de-ionized water to give the solid supported alkyne peptide.

[15] Phenol was introduced to reduce the basic condition of the reaction. Better results in term of purity were observed with an equimolar addition of phenol during TIPS deprotection

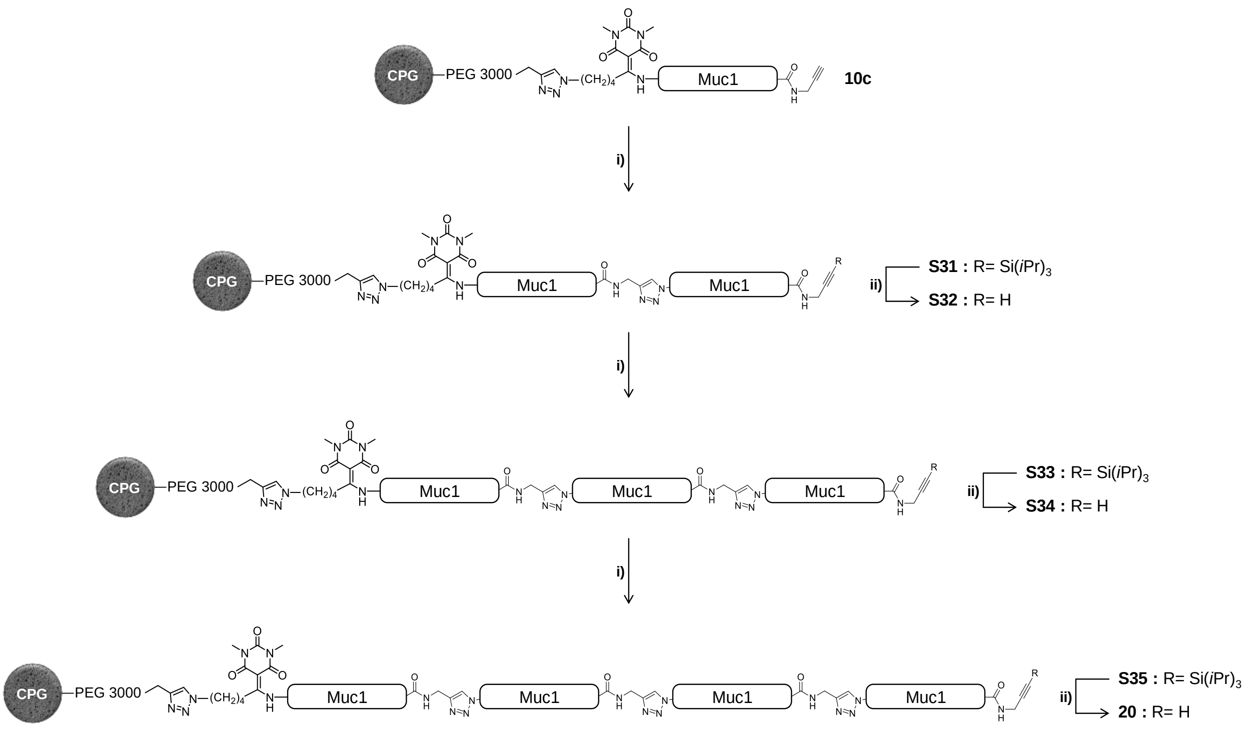

Supplementary scheme S39 - Introduction of a biotin probe on solid supported peptide **10c**

The N-to-C elongation of **10c** (1 µmol) through multiple solid phase PTLs was performed by repeating three times a succession of **i)** solid-supported peptidomimetic triazole ligations with azido alkyne **19** and **ii)** solid-supported deprotection of the TIPS group.

After each step, a few beads of the resulting solid supported peptide were cleaved using the general procedure (p S53) and the released peptide was analyzed by RP-HPLC and HRMS.

- Analysis of **S31’**

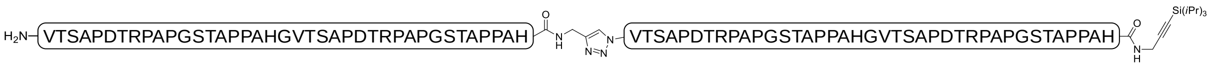

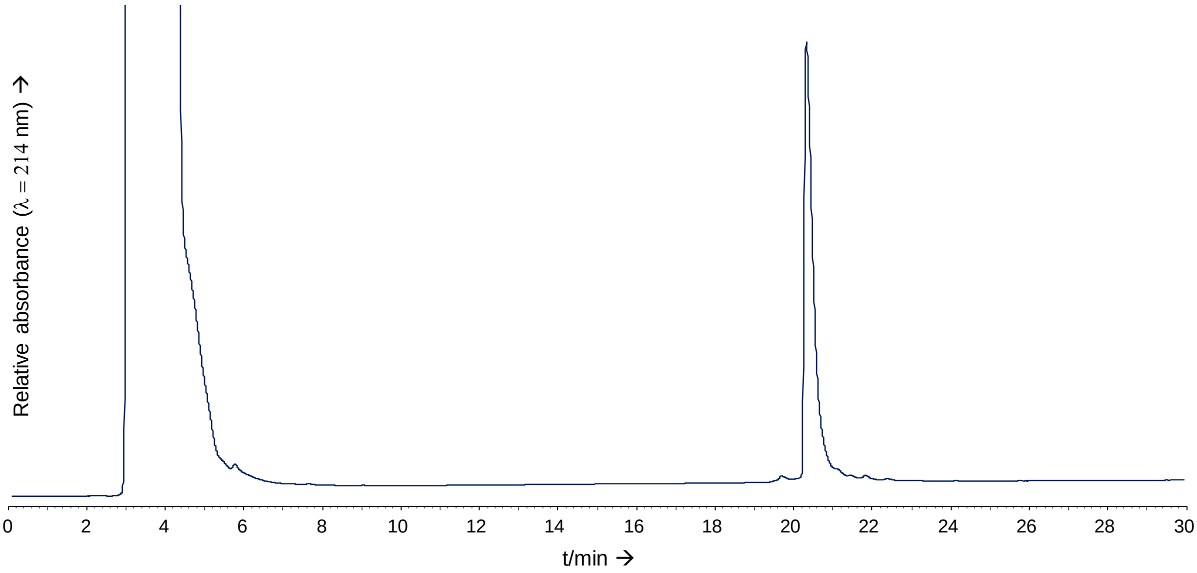

Supplementary figure S83 - Analytical HPLC profile of crude peptide **S31’** released from solid supported peptide **S31**. Column: Nucleosil®; gradient: from 30% to 60% B over 30 min at 60° C.

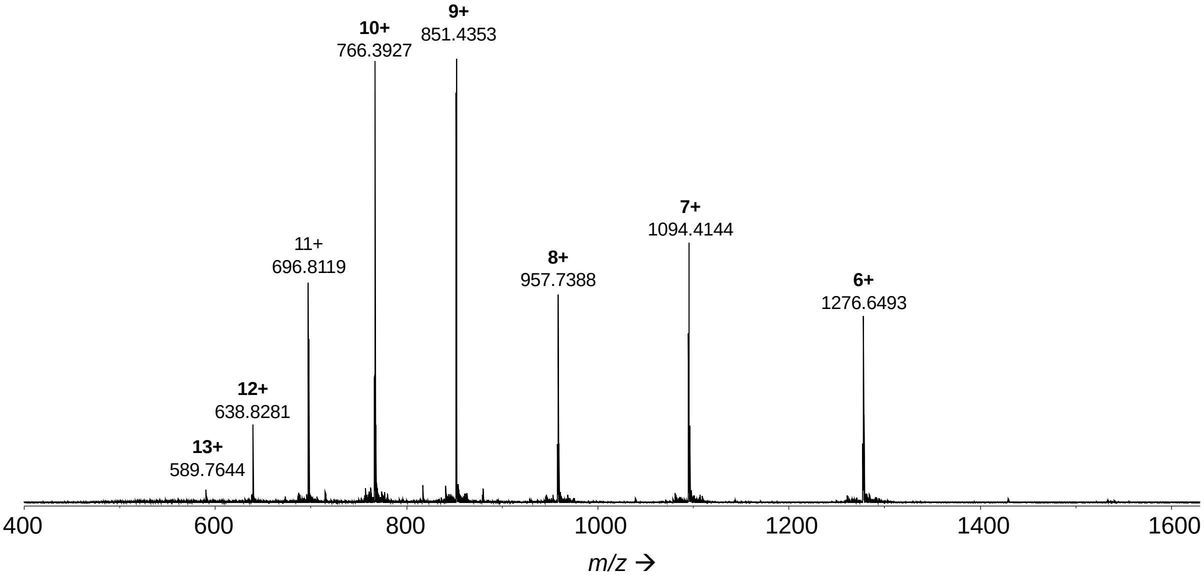

Supplementary figure S84 - ESI-HRMS mass spectrum of peptide **S31’**

ESI-HRMS (*m/z*): $[M+H]^+$ calcd for $C_{331}H_{523}N_{102}O_{106}Si$: 7650.8439, found: 7650.8464

- Analysis of **S32’**

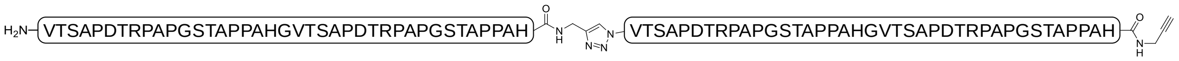

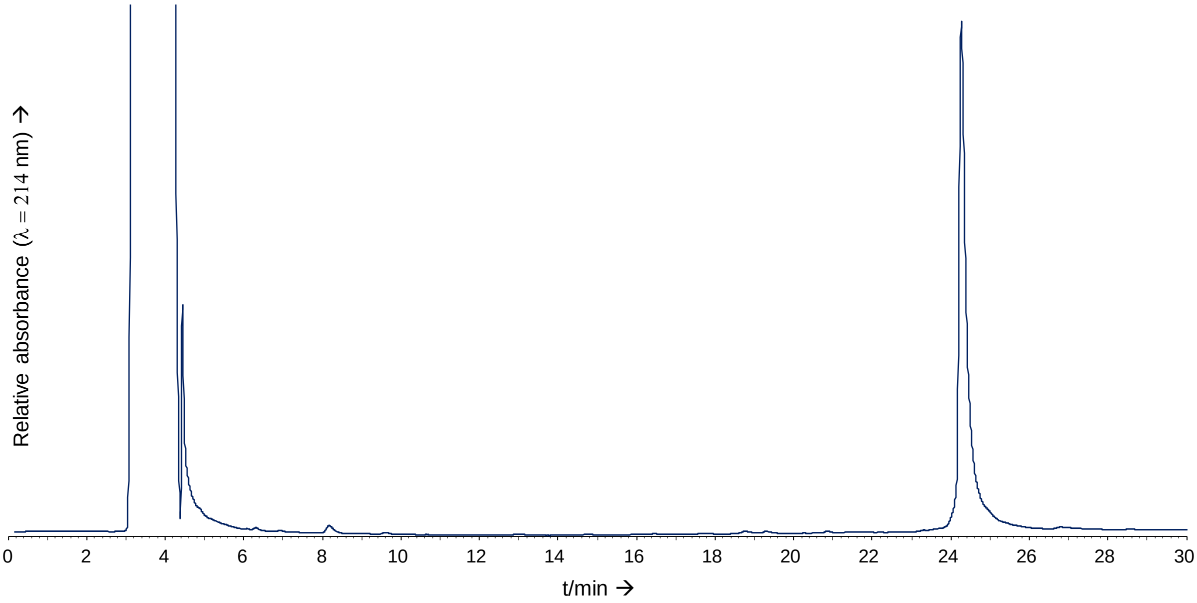

<u>Supplementary figure S85</u> - Analytical HPLC profile of crude peptide **S32’** released from solid supported peptide **S32**. Column: Nucleosil®; gradient: from 10% to 25% B over 30 min at 60° C.

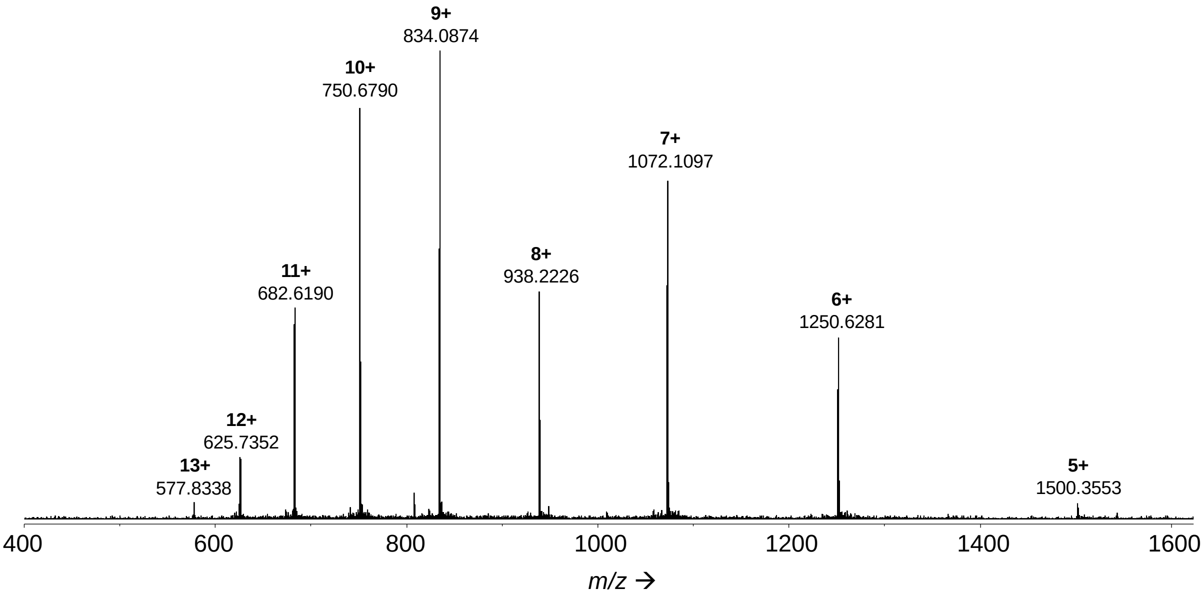

<u>Supplementary figure S86</u> - ESI-HRMS mass spectrum of peptide **S32’**

ESI-HRMS (*m/z*): $[M+H]^+$ calcd for $C_{322}H_{503}N_{102}O_{106}$: 7494.710513, found: 7494.7133

- Analysis of **S33'**

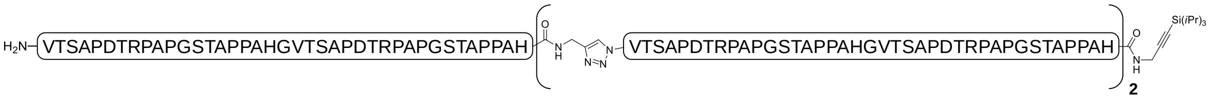

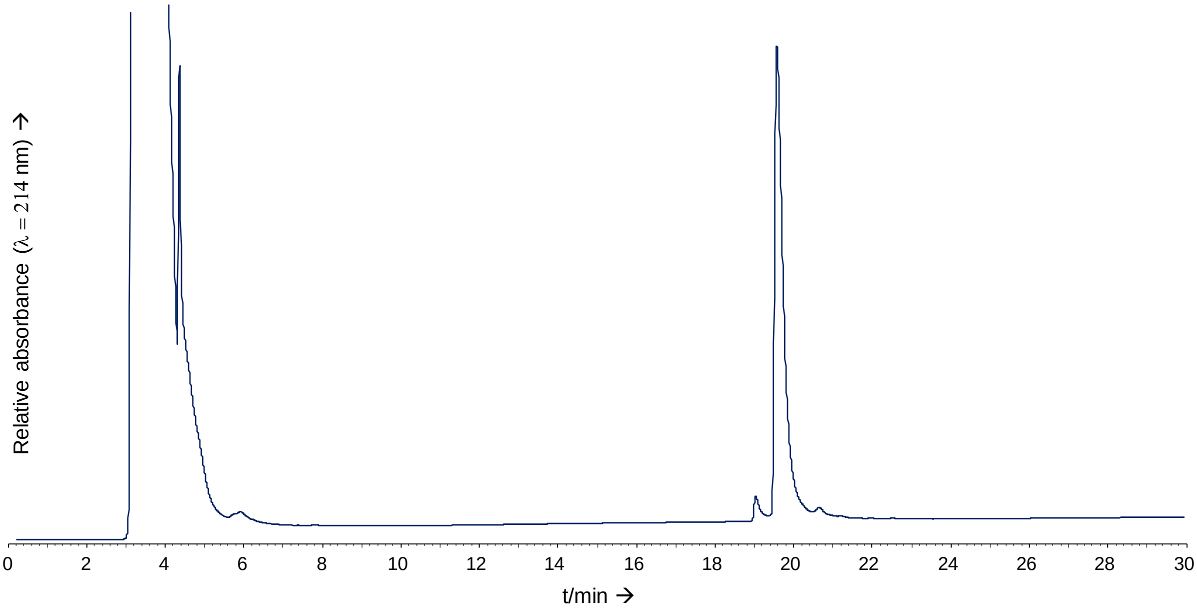

Supplementary figure S87 - Analytical HPLC profile of crude peptide **S33'** released from solid supported peptide **S33**. Column: Nucleosil®; gradient: from 30% to 60% B over 30 min at 60° C.

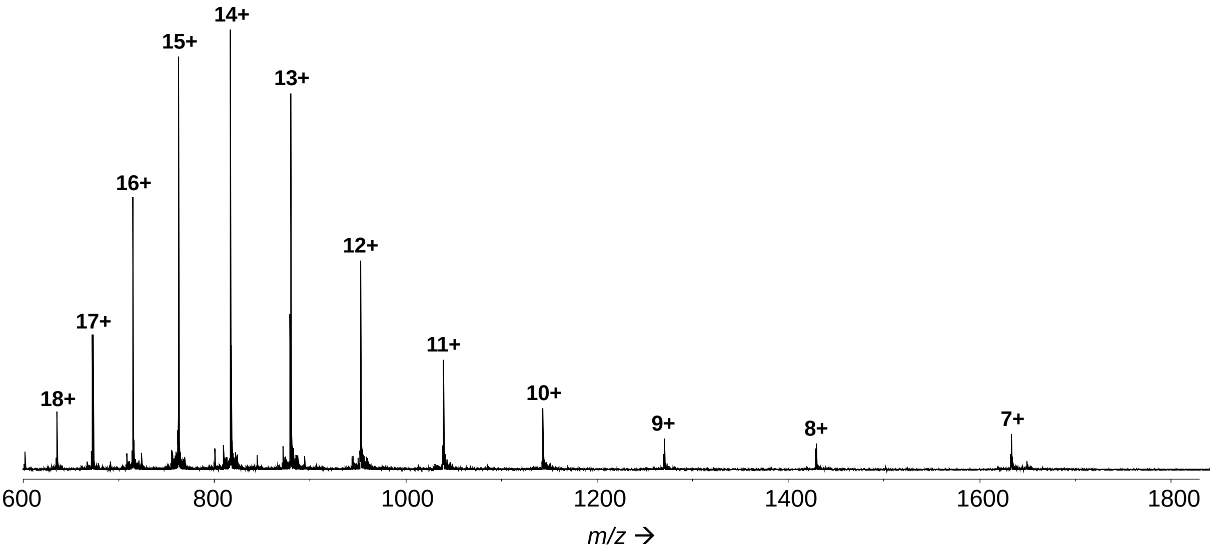

Supplementary figure S88 - ESI-HRMS mass spectrum of peptide **S33'**

$[M+18H]^{18+}$:626.5403, $[M+17H]^{17+}$:663.3361, $[M+16H]^{16+}$: 704.7945, $[M+15H]^{15+}$: 751.5786, $[M+14H]^{14+}$: 805.2638, $[M+13H]^{13+}$: 867.1291, $[M+12H]^{12+}$: 939.3058, $[M+11H]^{11+}$: 1024.6057, $[M+10H]^{10+}$: 1127.1676, $[M+9H]^{9+}$: 1252.0694, $[M+8H]^{8+}$: 1408.4567, $[M+7H]^{7+}$:1609.6606

ESI-HRMS (*m/z*): $[M+H]^+$ calcd for $C_{492}H_{773}N_{154}O_{159}Si$: 11410.6905, found: 114010.6995

- Analysis of **S34'**

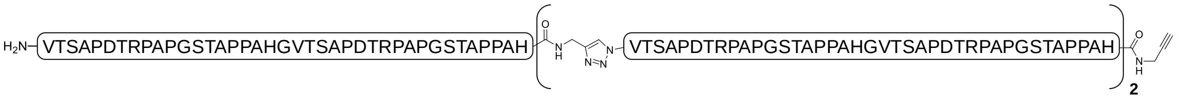

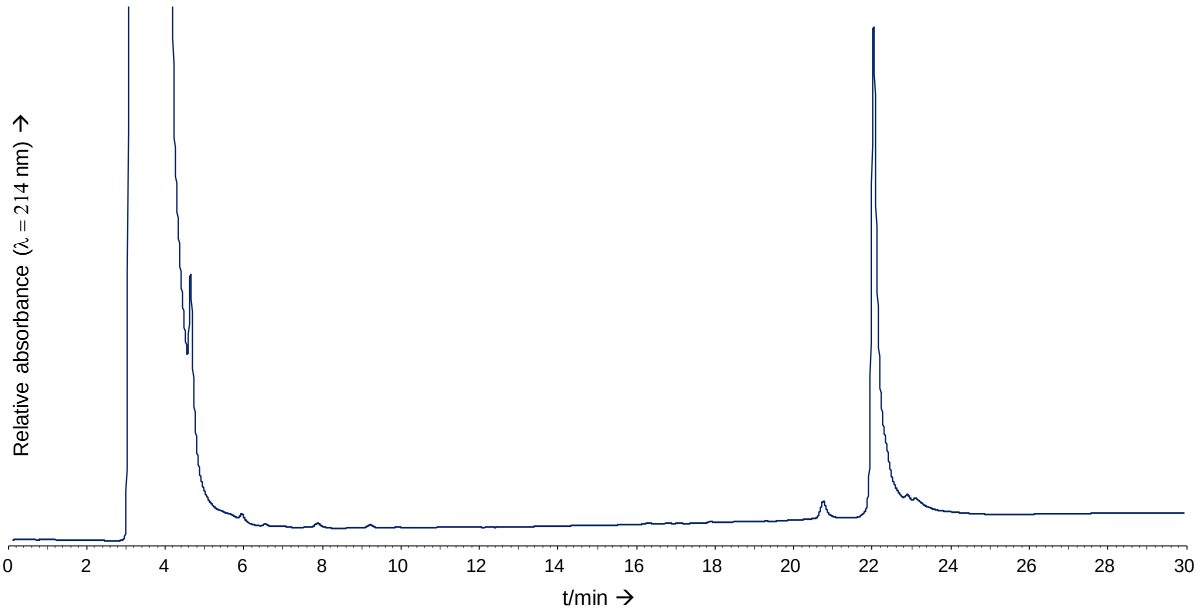

<u>Supplementary figure S89</u> - Analytical HPLC profile of crude peptide **S34'** released from solid supported peptide **S34**. Column: Nucleosil®; gradient: from 10% to 25% B over 30 min at 60° C.

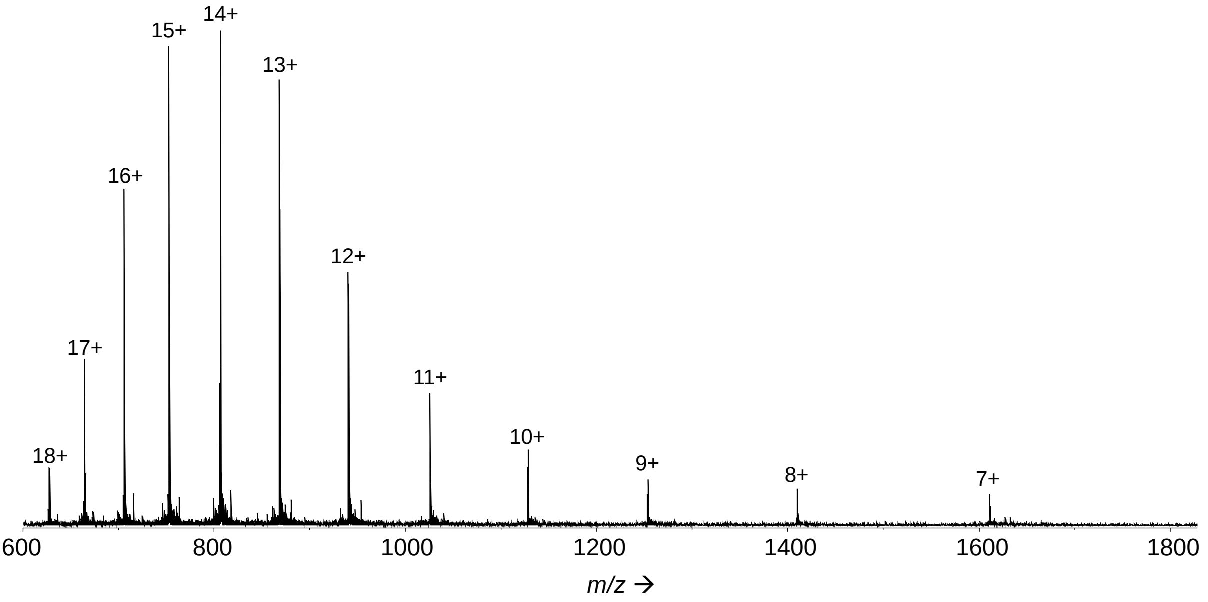

<u>Supplementary figure S90</u> - ESI-HRMS mass spectrum of peptide **S34**

$[M+18H]^{18+}$: 626.5403, $[M+17H]^{17+}$: 663.3361, $[M+16H]^{16+}$: 704.7945, $[M+18H]^{15+}$: 751.5786, $[M+14H]^{14+}$: 805.2638, $[M+13H]^{13+}$: 867.1291, $[M+12H]^{12+}$: 939.3058, $[M+11H]^{11+}$: 1024.6057, $[M+10H]^{10+}$:1127.1676, $[M+9H]^{9+}$:1252.0694, $[M+8H]^{8+}$:1408.4567, $[M+7H]^{7+}$:1609.6606

ESI-HRMS (*m/z*): $[M+H]^+$ calcd for $C_{483}H_{753}N_{154}O_{159}$: 11254.5571, found: 11254.5726

- Analysis of **S35’**

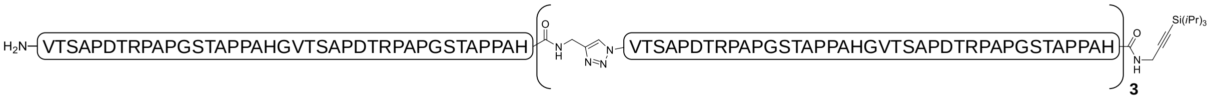

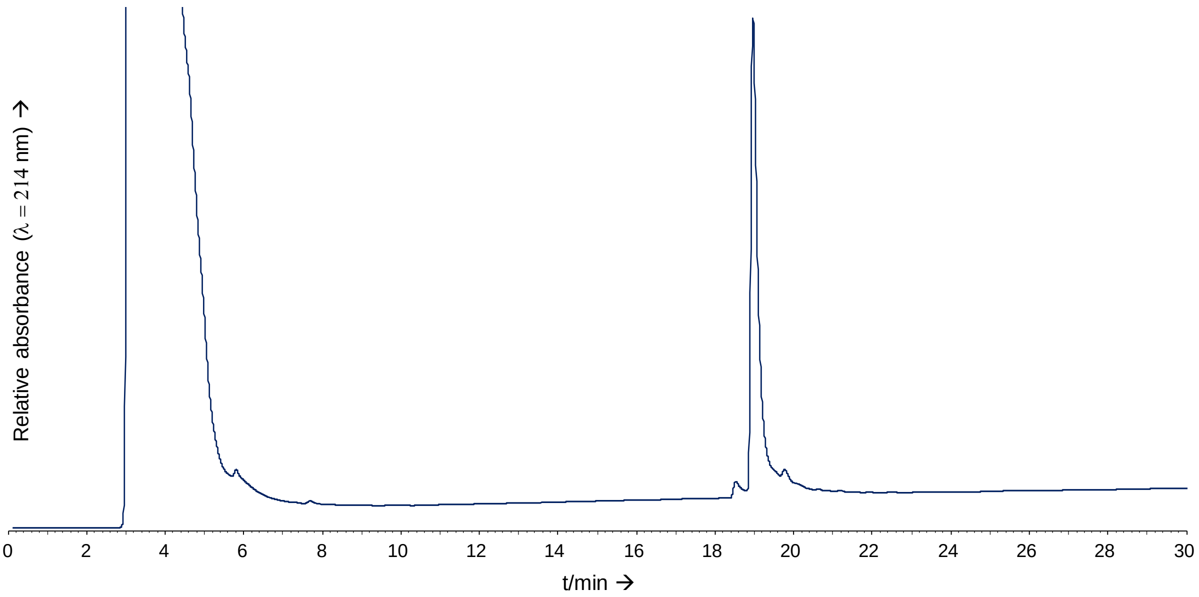

Supplementary figure S74 - Analytical HPLC profile of crude peptide **S35’** released from solid supported peptide **S35**. Column: Nucleosil®; gradient: from 30% to 60% B over 30 min at 60° C

.

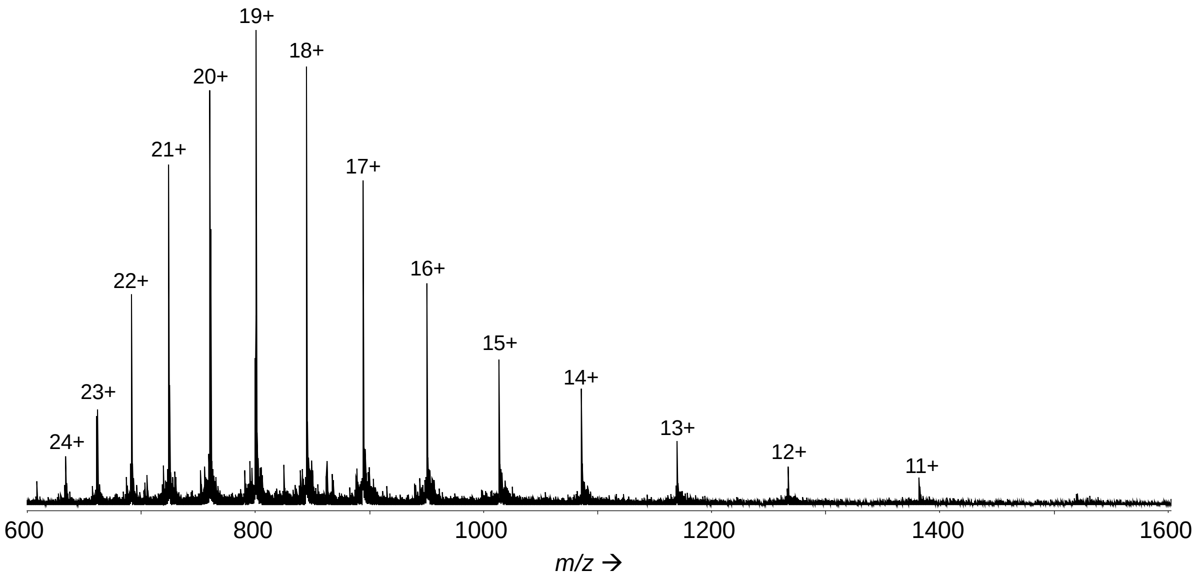

Supplementary figure S91 - ESI-HRMS mass spectrum of peptide **S35’**

$[M+24H]^{24+}$:633.4070, $[M+23H]^{23+}$: 660.9009, $[M+22H]^{22+}$: 690.8977, $[M+21H]^{21+}$: 723.7966, $[M+20H]^{20+}$: 759.9360, $[M+19H]^{19+}$: 799.8281,$[M+18H]^{18+}$: 844.2625,$[M+17H]^{17+}$: 893.7464, $[M+16H]^{16+}$: 949.6047, $[M+15H]^{15+}$: 1012.7790, $[M+14H]^{14+}$: 1085.1189, $[M+13H]^{13+}$: 1168.3550, $[M+12H]^{12+}$: 1265.8080, $[M+11H]^{11+}$:1380.8706

ESI-HRMS (*m/z*): $[M+H]^+$ calcd for $C_{653}H_{1023}N_{206}O_{212}Si$: 15170.5371, found: 15170.5610

- Analysis of **21**

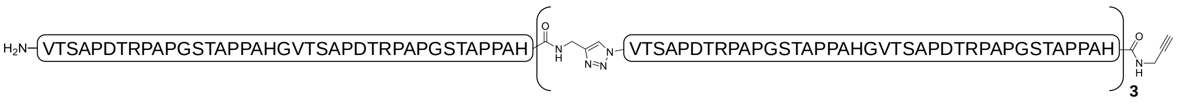

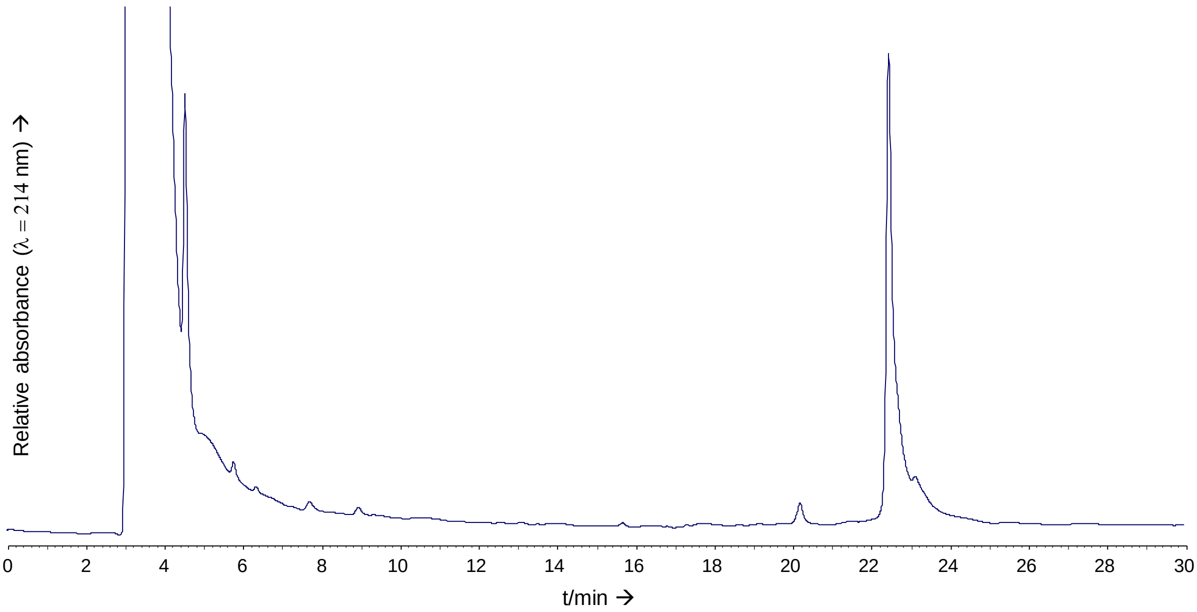

Supplementary figure S92 - Analytical HPLC profile of crude peptide **21** released from solid supported peptide **20**. Column: Nucleosil®; gradient: from 15% to 25% B over 30 min at 60° C.

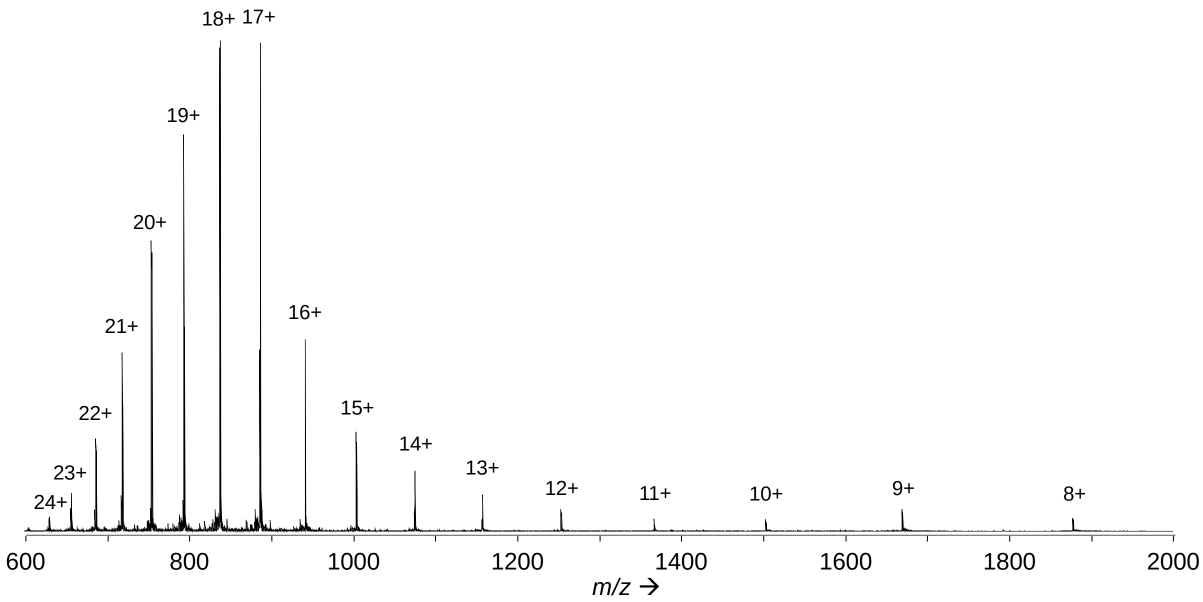

Supplementary figure S93 - ESI-HRMS mass spectrum of peptide **21**

$[M+24H]^{24+}$:, $[M+23H]^{23+}$: 654.1563, $[M+22H]^{22+}$: 683.7546 , $[M+21H]^{21+}$: 716.3613 , $[M+20H]^{20+}$: 752.0792, $[M+19H]^{19+}$: 791.6090,$[M+18H]^{18+}$: 835.5864,$[M+17H]^{17+}$: 884.6209, $[M+16H]^{16+}$: 939.8461, $[M+15H]^{15+}$: 1002.4357, $[M+14H]^{14+}$: 1073.9662, $[M+13H]^{13+}$: 1156.5776, $[M+12H]^{12+}$: 1252.7928, $[M+11H]^{11+}$:1366.5934, $[M+10H]^{10+}$: 1503.1474, $[M+9H]^{9+}$: 1670.1626, $[M+8H]^{8+}$: 1878.8129

ESI-HRMS (*m/z*): $[M+H]^+$ calcd for $C_{644}H_{1003}N_{206}O_{212}$: 15014.4036, found: 15014.4085

L Subsequent solid supported enzymatic glycosylation

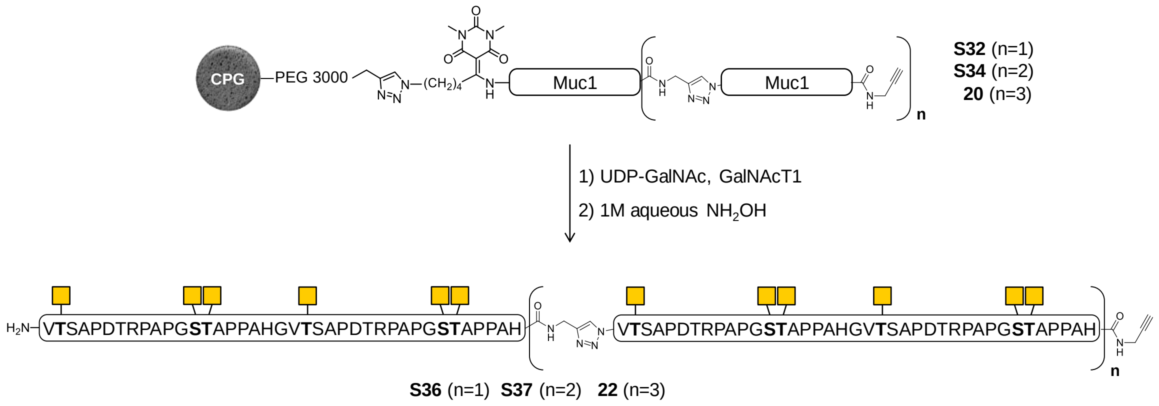

Supplementary scheme S40 - Enzymatic glycosylation and release of solid supported peptide

Solid supported glycopeptides **S32**, **S34** and **20** (20 nmol) were introduced in a 2 mL microcentrifuge tube and washed with de-ionized water (2 x 500 µL). To the solid was added 100 µL of an aqueous solution containing GalNAc-T1 (6 mU), MES (50 mM, pH 6.5), $MnCl_2$ (15 mM), BSA (100 ng), DTT (1 mM) and UDP-GalNAc (2 equiv. per glycosylation sites). The resulting suspension was mixed at 37 °C for 48 h under moderate agitation. Beads were then washed with de-ionized water (2 x 500 µL), in order to remove the UDP formed during the transfer reaction since it inhibits GalNAc-T1 activity. Solid supported glycopeptide was then incubated under the same conditions as above for another 48 h and the resin was washed with de-ionized water.

The resulting solid supported glycopeptides were released under typical procedure (p S53) to give glycopeptide mixture which was purified by semi-preparative HPLC and lyophilized to yield pure homogeneous glycopeptide containing 3 *O*-GalNAc per Muc1 tandem repeat.

- Analysis of **S36**

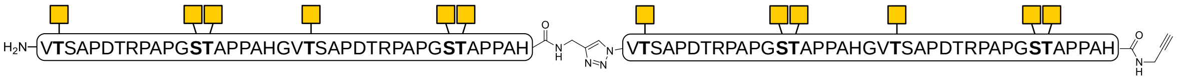

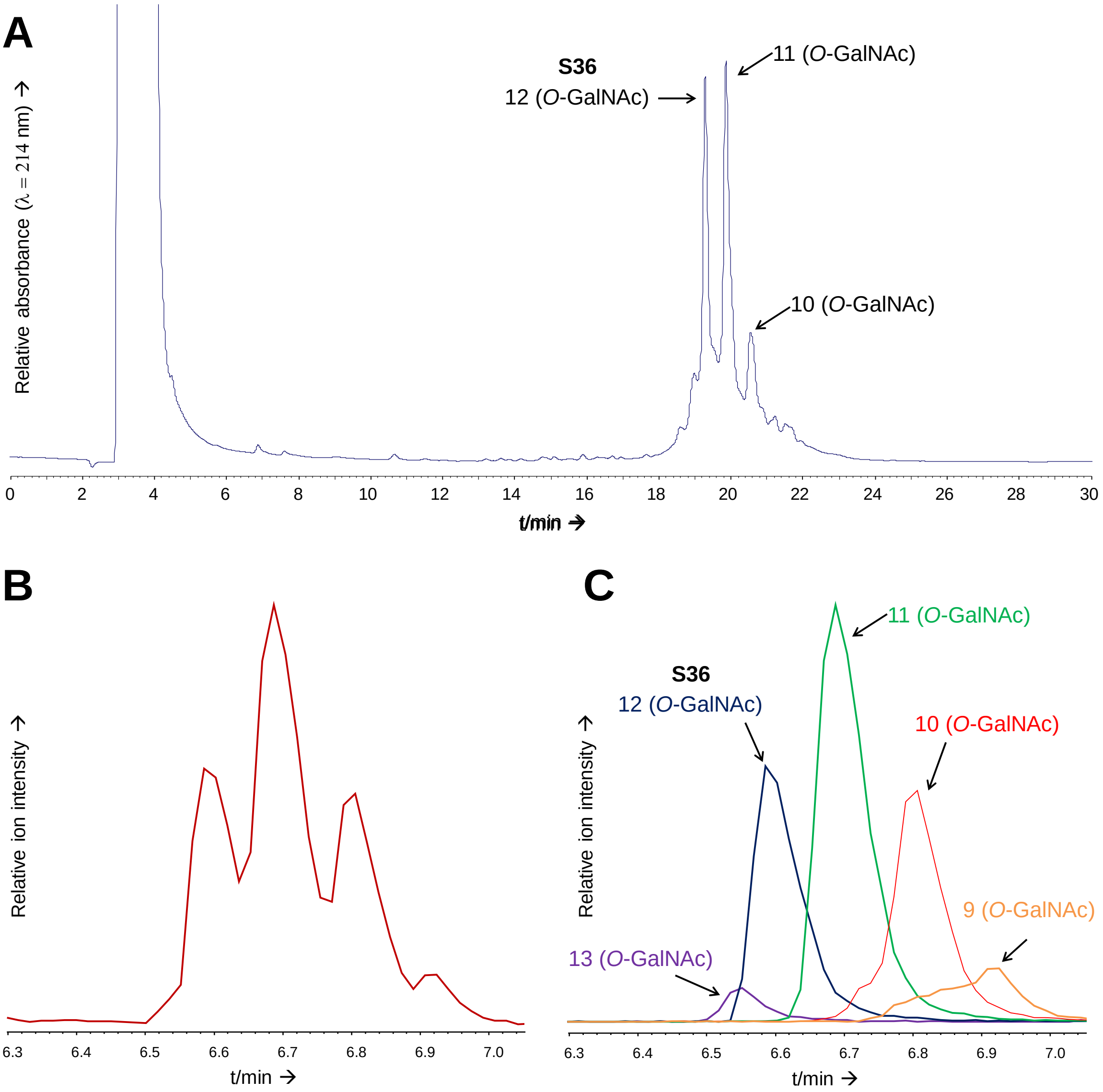

Supplementary figure S94 - Analytical HPLC profile of crude mixture containing glycopeptide S36. Column: Nucleosil®; gradient: from 10% to 25% B over 30 min at 60° C. **B)** Total-ion chromatogram **C)** Extracted-ion chromatogram of glycopeptides containing 13, 12, 11, 10 and 9 *O*-GalNAc, respectively. Column: Zorbax 300; gradient: from 3% to 50% B over 20 min

- ESI-HRMS analysis of crude mixture containing glycopeptide **S36**

GalNAc units added per peptide	Formula	$[M+H]^+$ calcd	$[M+H]^+$ found	Relative abundance[a]
13	$C_{426}H_{672}N_{115}O_{171}$	10134.7423	10134.7502	2.6%
12	$C_{418}H_{659}N_{114}O_{166}$	9931.6630	9931.6671	23.8%
11	$C_{410}H_{646}N_{113}O_{161}$	9728.5836	9728.6042	40.7%
10	$C_{402}H_{633}N_{112}O_{156}$	9525.5043	9525.5626	24.2%
9	$C_{394}H_{620}N_{111}O_{151}$	9322.4248	9322.4363	8.7%

[a] Estimated by peak area integration on extracted-ion chromatograms; not a rigorous quantification, due to probable differences in ionization of the compounds.

- RP-HPLC purification of **S36**

Yield: 14.8%; estimated by integration of the HPLC peak at λ = 214 nm.

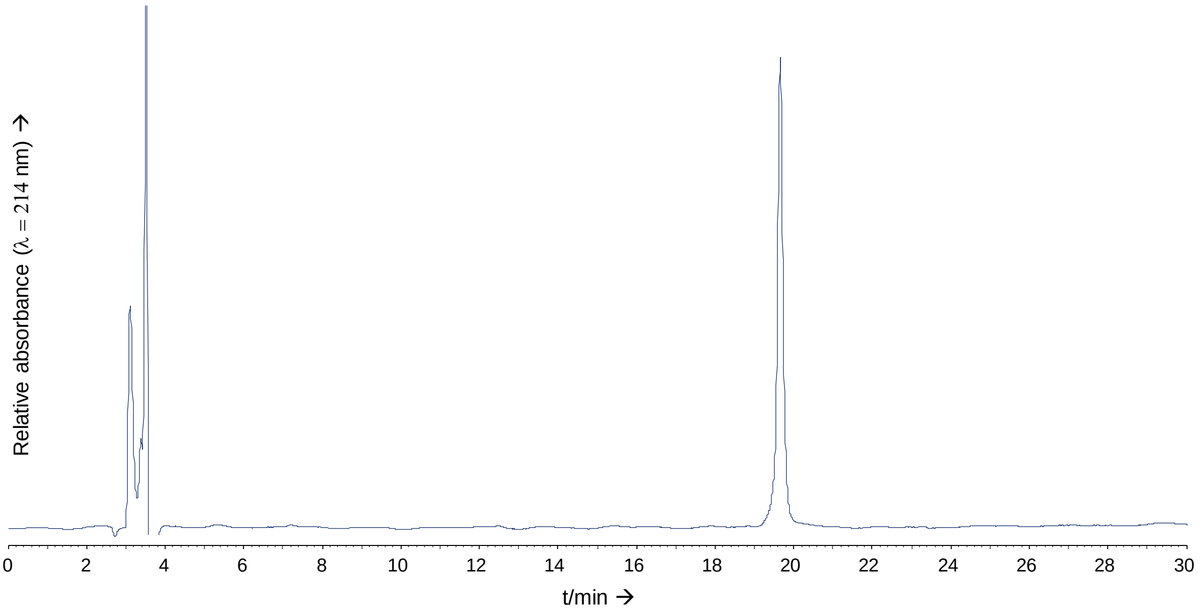

Supplementary figure S95 - Analytical HPLC profile of purified glycopeptide **S36**. Column: Nucleosil®; gradient: from 10% to 25% B over 30 min at 60° C.

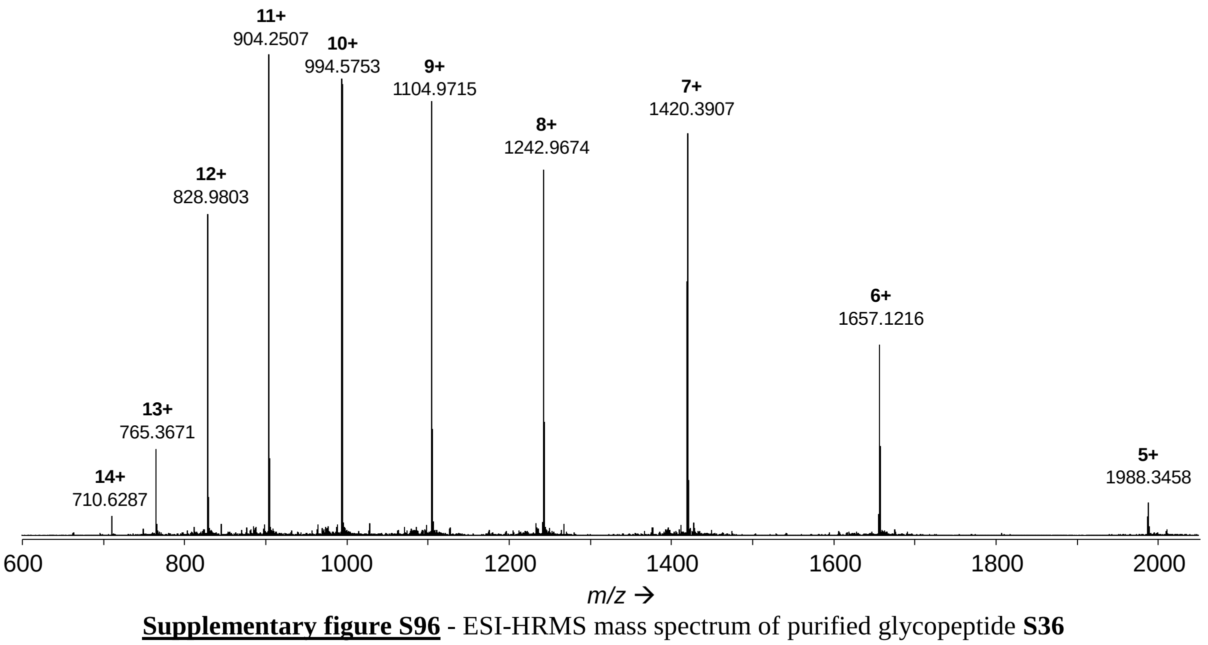

Supplementary figure S96 - ESI-HRMS mass spectrum of purified glycopeptide **S36**

ESI-HRMS (*m/z*): $[M+H]^+$ calcd for $C_{418}H_{659}N_{114}O_{166}$: 9931.6630, found: 9931.6671

- Analysis of **S37**

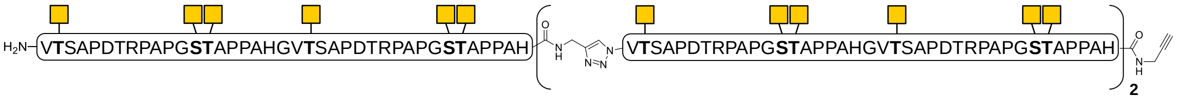

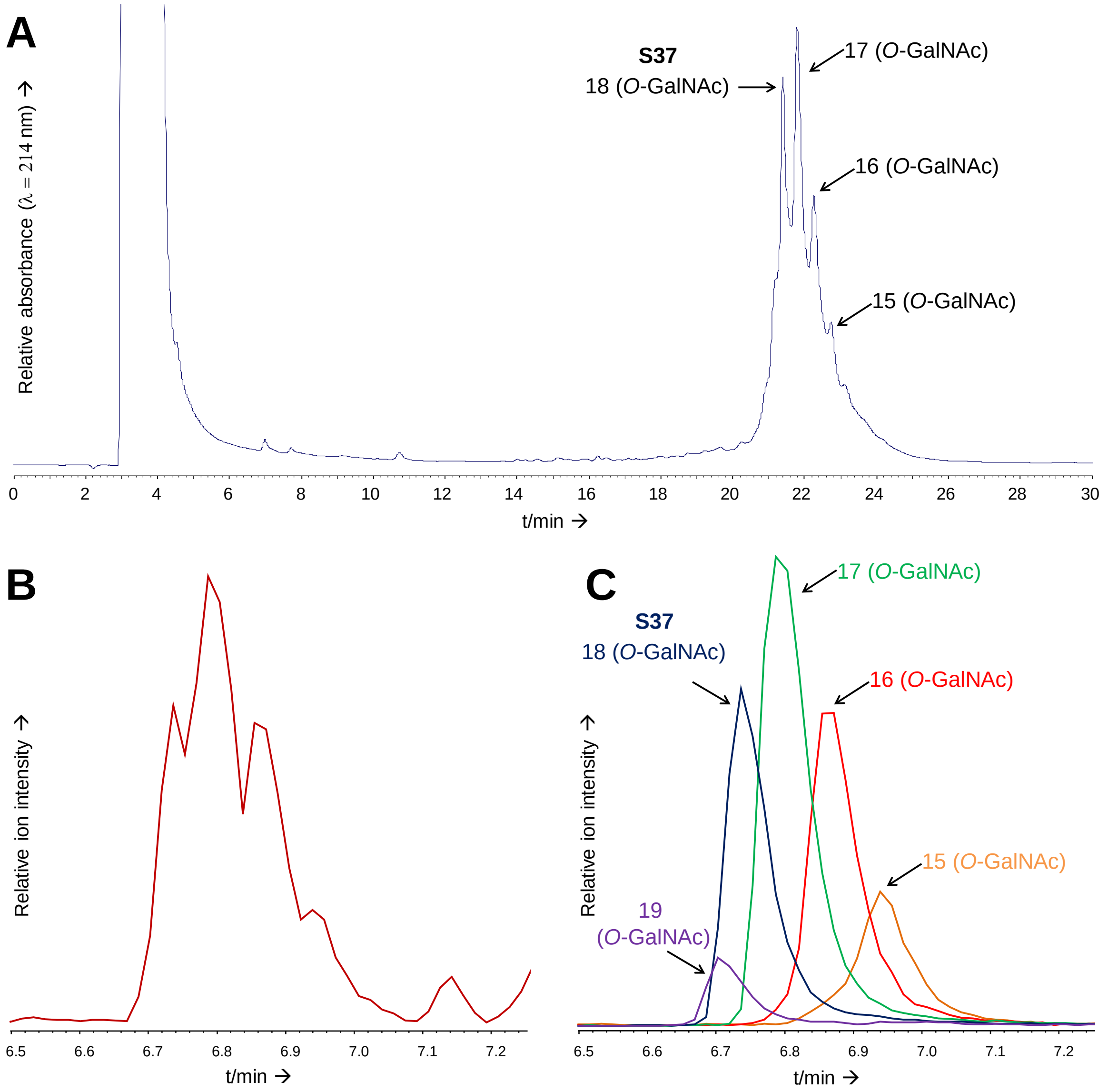

Supplementary figure S97 - Analytical HPLC profile of crude mixture containing glycopeptide S37. Column: Nucleosil®; gradient: from 10% to 25% B over 30 min at 60° C. B) Total-ion chromatogram C) Extracted-ion chromatogram of glycopeptides containing 19, 18, 17,16 and 15 *O*-GalNAc, respectively. Column: Zorbax 300; gradient: from 3% to 50% B over 20 min

- ESI-HRMS analysis of crude mixture containing glycopeptide **S37**

GalNAc units added per peptide	Formula	Average mass $[M+H]^+$ calcd	Average mass $[M+H]^+$ found	Relative abundance[a]
19	$C_{635}H_{1000}N_{173}O_{254}$	15121.70	15121.74	4.2%
18	$C_{627}H_{987}N_{172}O_{249}$	14918.548	14918.590	26.6%
17	$C_{619}H_{974}N_{171}O_{244}$	14715.356	14715.411	39.6%
16	$C_{611}H_{961}N_{170}O_{239}$	14512.163	14512.214	27.5%
15	$C_{603}H_{948}N_{169}O_{234}$	14308.971	14308.868	2.1%

[a] Estimated by peak area integration on extracted-ion chromatograms; not a rigorous quantification, due to probable differences in ionization of the compounds.

- RP-HPLC purification of **S37**

Yield: 9.8%; estimated by integration of the HPLC peak at λ = 214 nm.

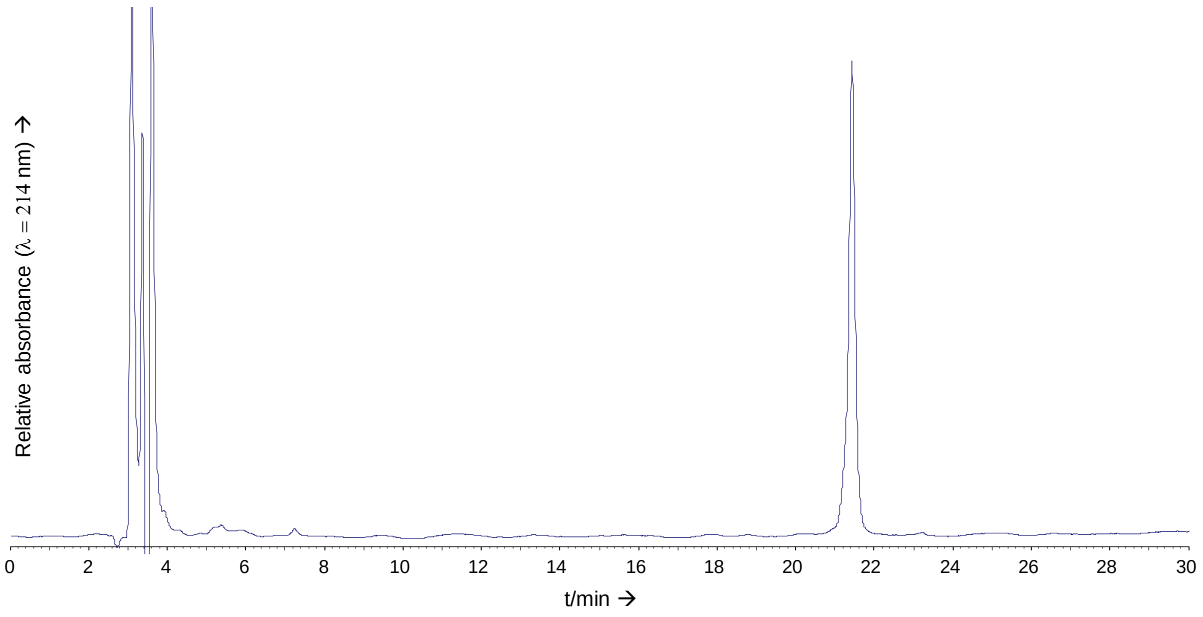

Supplementary figure S98 - ** Analytical HPLC profile of purified glycopeptide **S37. Column: Nucleosil®; gradient: from 10% to 25% B over 30 min at 60° C.

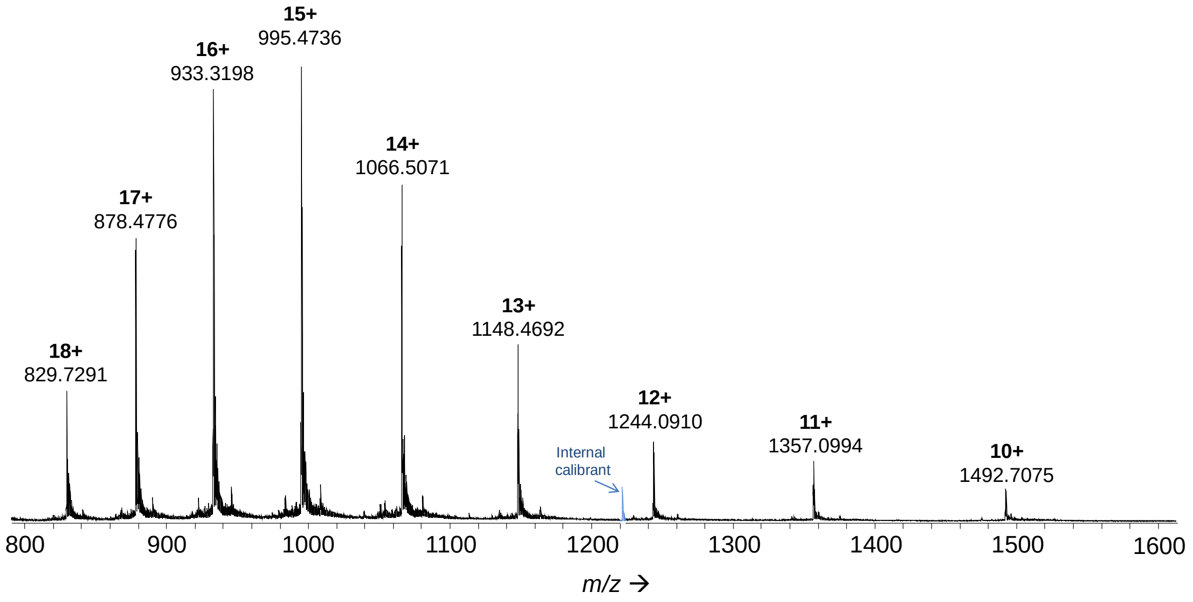

Supplementary figure S99 - ESI-HRMS mass spectrum of peptide **S37**

ESI-HRMS (*m/z*): $[M+H]^+$ average mass calcd for : $C_{627}H_{987}N_{172}O_{249}$: 14918.548 , found: 14918.590

Internal calibrant: $C_{24}H_{19}F_{36}N_3O_6P_3$ (*m/z* 1221.990623)

- Analysis of **22**

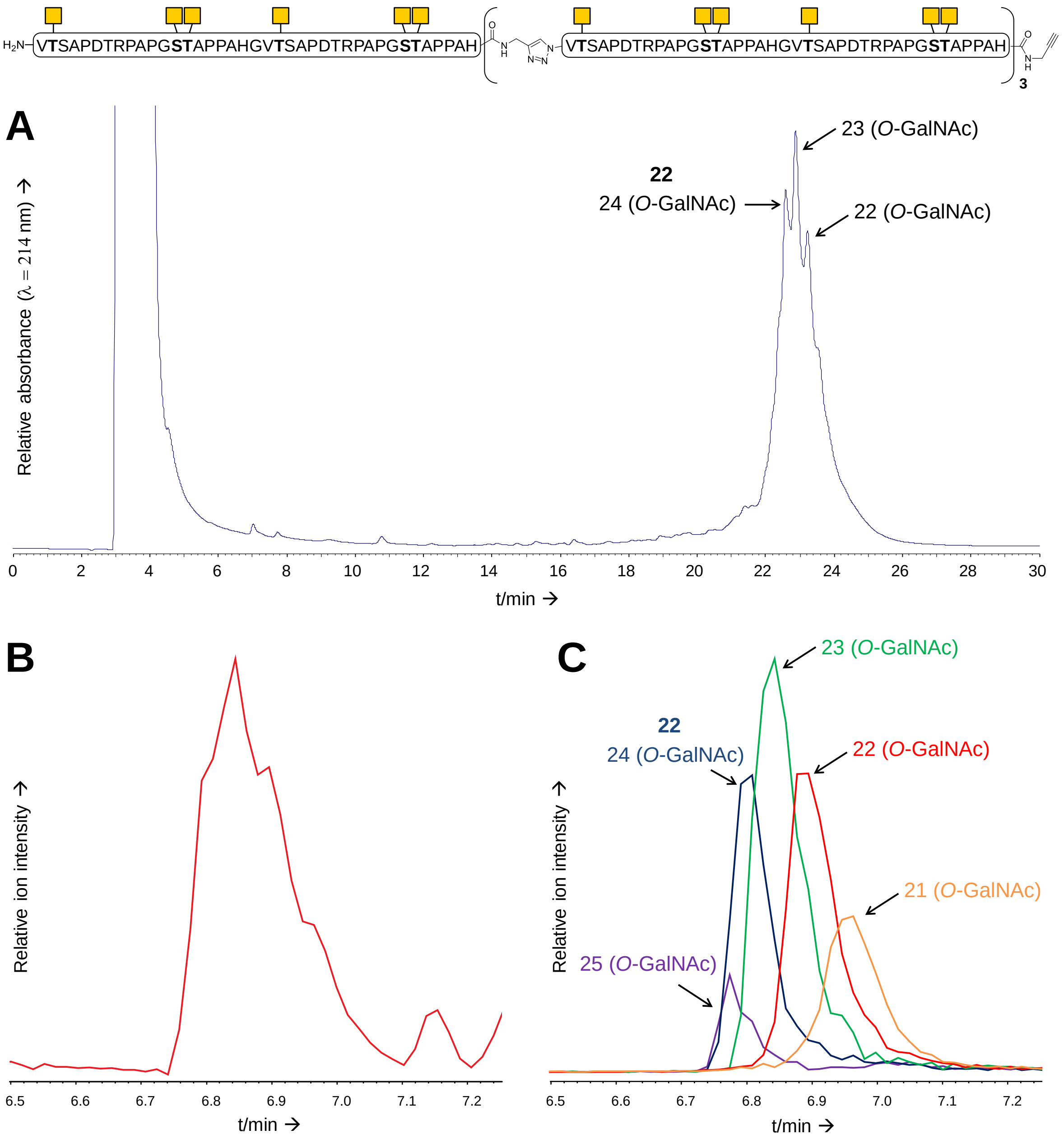

Supplementary figure S100 - Analytical HPLC profile of crude mixture containing glycopeptide **22**. Column: Nucleosil®; gradient: from 10% to 25% B over 30 min at 60° C. B) Total-ion chromatogram C) Extracted-ion chromatogram of glycopeptides containing 25, 24, 23, 22 and 21 *O*-GalNAc, respectively. Column: Zorbax 300; gradient: from 3% to 50% B over 20 min

- ESI-HRMS analysis of crude mixture containing glycopeptide **22**

GalNAc units added per peptide	Formula	Average mass $[M+H]^+$ calcd	Average mass $[M+H]^+$ found	Relative abundance[a]
25	$C_{844}H_{1327}N_{231}O_{337}$	20101.86	20102.10	4.2%
24	$C_{836}H_{1314}N_{230}O_{332}$	19898.72	19898.37	22.3%
23	$C_{828}H_{1301}N_{229}O_{327}$	19695.52	19695.05	33.4%
22	$C_{820}H_{1288}N_{228}O_{322}$	19492.33	19492.64	24.9%
21	$C_{812}H_{1275}N_{227}O_{317}$	19289.09	19289.66	15.2%

[a] Estimated by peak area integration on extracted-ion chromatograms; not a rigorous quantification, due to probable differences in ionization of the compounds.

- RP-HPLC purification of **22**

Yield: 5.9%; estimated by integration of the HPLC peak at λ = 214 nm.

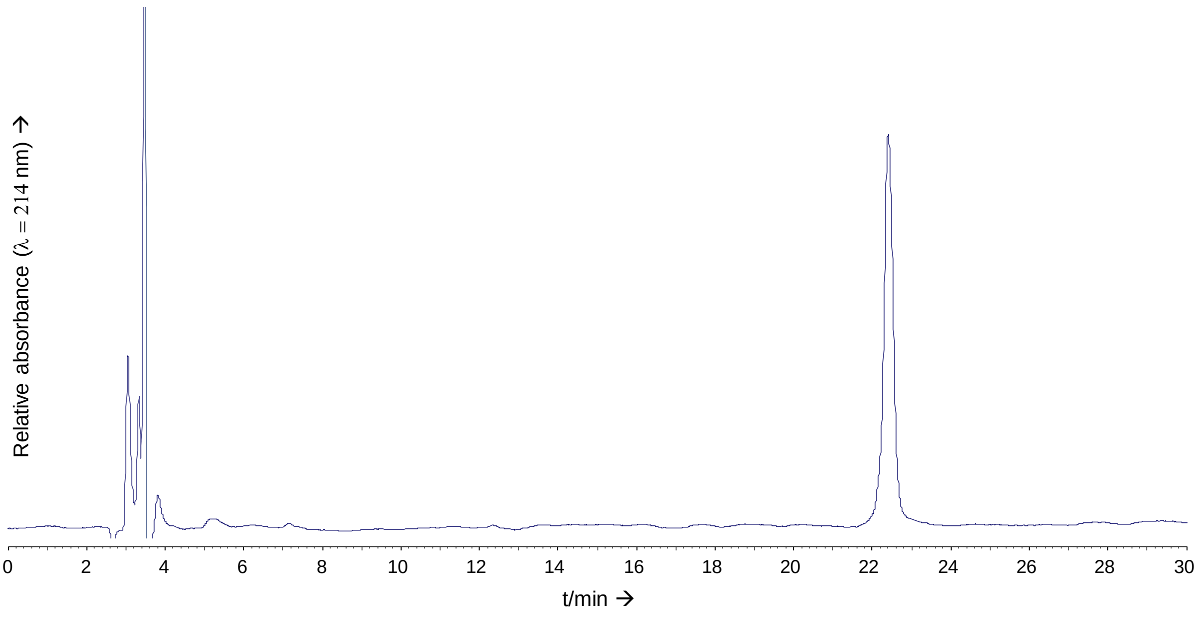

Supplementary figure S101 - Analytical HPLC profile of purified glycopeptide **22**. Column: Nucleosil®; gradient: from 10% to 25% B over 30 min at 60° C.

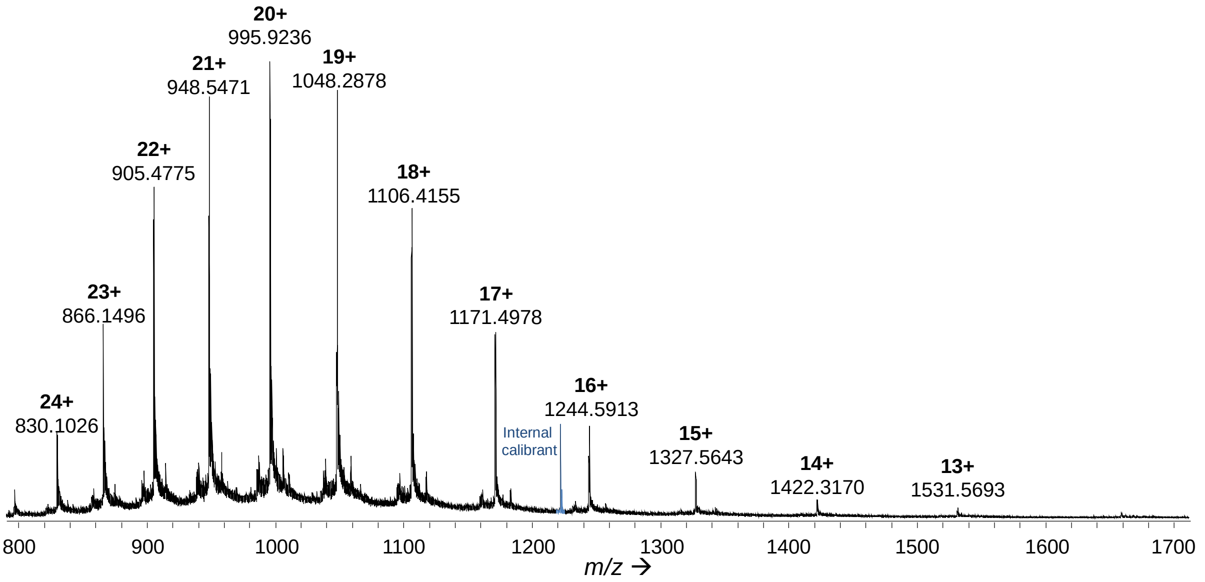

Supplementary figure S102 - ESI-HRMS mass spectrum of peptide **22**

ESI-HRMS (*m/z*): $[M+H]^+$ average mass calcd for : $C_{836}H_{1314}N_{230}O_{332}$: 19898.72, found: 19898.37

Internal calibrant: $C_{24}H_{19}F_{36}N_3O_6P_3$ (*m/z* 1221.990623)
